# Duplication, Loss, and Feeding Ecology of Lysozyme Gene Copy Number in Insects

**DOI:** 10.1093/gbe/evag265

**Published:** 2026-10-22

**Authors:** Héctor Antônio Assunção Romão, Carolina Kurotusch Canettieri, Thalison Rodrigues Moreira, Karoliny Zarreta Santos Freire, Jaqueline Magalhães Pereira, José Alexandre Felizola Diniz-Filho, Rhewter Nunes, Adriana Rios Lopes, Walter R Terra, Clayton Luiz Borges, Renata de Oliveira Dias

**Affiliations:** Instituto de Ciências Biológicas, Universidade Federal de Goiás (UFG), Goiânia, GO, Brasil; Instituto de Ciências Biológicas, Universidade Federal de Goiás (UFG), Goiânia, GO, Brasil; Instituto de Ciências Biológicas, Universidade Federal de Goiás (UFG), Goiânia, GO, Brasil; Escola de Agronomia (EA), Universidade Federal de Goiás (UFG), Goiânia, GO, Brasil; Escola de Agronomia (EA), Universidade Federal de Goiás (UFG), Goiânia, GO, Brasil; Instituto de Ciências Biológicas, Universidade Federal de Goiás (UFG), Goiânia, GO, Brasil; Instituto Acadêmico de Ciências da Saúde e Biológicas (IACSB), Universidade Estadual de Goiás (UEG)—Campus Oeste—UnU de Iporá, Iporá, GO, Brasil; Laboratório de Bioquímica e Biofísica, Instituto Butantan, São Paulo, SP, Brasil; Instituto de Química (IQ), Universidade de São Paulo (USP), São Paulo, SP, Brasil; Instituto de Ciências Biológicas, Universidade Federal de Goiás (UFG), Goiânia, GO, Brasil; Instituto de Ciências Biológicas, Universidade Federal de Goiás (UFG), Goiânia, GO, Brasil

**Keywords:** antimicrobial peptide, comparative genomics, feeding ecology, gene duplication and loss, muramidase

## Abstract

Lysozymes are ubiquitous enzymes traditionally associated with immune defense via bacterial cell wall degradation. In some insect lineages, particularly Diptera with bacteria-rich diets, these enzymes have been secondarily recruited for digestion, but how widespread this shift is across other insect groups remains unclear. Here, we investigate the evolutionary dynamics and functional roles of C-type and I-type lysozymes across 275 insect genomes, inferring patterns of gene duplication and loss and testing associations between lysozyme copy number and dietary habits. Phylogenetic Generalized Least Squares method revealed few significant associations: a weak link between larval detritivory and C-type copy number, and a more consistent one between an omnivorous diet and I-type copy number across life stages. Duplication analysis revealed multiple independent expansions of C-type lysozymes within Brachycera. In Muscoidea, C-type lysozymes were highly expressed in the *Musca domestica* larval midgut, consistent with digestion, whereas in Blattodea, moderate C-type copy numbers and salivary gland-biased expression in *Periplaneta americana* adults instead suggest a primarily antiseptic role. I-type lysozymes lack the catalytic residues required for canonical muramidase activity, yet remain conserved throughout Insecta, including in lineages that lost C-type lysozymes, suggesting alternative, non-canonical functions. Together, these findings are more consistent with a predominantly lineage-specific pattern of gene family evolution, largely uncoupled from a single ecological driver, than with a broad, diet-driven adaptive signal. Digestive recruitment of C-type lysozymes may extend beyond the well-characterized cases of *Musca domestica* and *Drosophila melanogaster* to additional expanded lineages across Brachycera, although direct functional evidence remains lacking.

Significance Lysozymes are best known as bactericidal immune enzymes, but some insects have repurposed them for digestion in bacteria-rich diets, raising the question of how common and predictable such functional shifts are across such a large and ecologically diverse group. By comparing lysozyme gene copy number, sequence features, and tissue expression across 275 insect genomes spanning most major orders, we found that gene duplication and loss occur largely independently of diet, with only isolated exceptions, suggesting that most of this gene family's diversity reflects neutral evolutionary turnover rather than direct dietary adaptation. This reframes insect lysozyme evolution as primarily governed by lineage history rather than feeding ecology, and offers a genome-scale reference point for identifying the rare cases, such as certain fly lineages, where digestive recruitment has plausibly occurred.

## Introduction

Lysozymes (EC 3.2.1.17) are hydrolytic enzymes that cleave the β-(1,4)-glycosidic bond between N-acetylmuramic acid and N-acetylglucosamine in peptidoglycan, displaying muramidase activity. In certain occasions, they can also cleave the β-(1,4)-glycosidic linkages between N-acetyl-D-glucosamine residues in chitodextrins, acting as chitinases. Lysozymes are present in a wide range of organisms, and so far, these enzymes have been classified into six categories: Ch (Chalaropsis), present in fungi, metazoans, bacteria, and viruses; T4, harbored by bacteriophages; bacterial, expressed by prokaryotes and commonly denominated autolysins; and G (goose), C (chicken) and I (invertebrate), all these found in animals, of which insects retain only C-type and I-type forms (Jollès and Jollès 1984; Callewaert and Michiels, 2010). Despite the similarities between their tertiary structures, they share little linear sequence identity, and each type has its own biochemical characteristics, cleaving bonds more or less fast and varying in muramidase/chitinase activity ratio. In general, in peptidoglycan catalysis, two acidic residues are involved: a nucleophilic aspartate and a proton-donor glutamate. This dyad is found at least in C, I, G, and phage types, and experimental results have proved them to be active in most lysozyme copies (Callewaert and Michiels, 2010). Beyond their canonical muramidase activity, insect C-type lysozymes have also been reported to display antifungal activity against yeasts through a muramidase-independent mechanism (Sowa-Jasiłek et al. 2016) and were induced upon infection with entomopathogenic fungi lineages (Ramirez et al. 2023), further broadening the immune functions attributed to this lysozyme type beyond bacteriolysis and digestion.

The evolution of lysozymes is a successful case of co-option through all life kingdoms. As peptidoglycan and chitodextrins are components of bacterial and fungal cell walls, these enzymes are primarily involved in immune defense against pathogens, promoting their lysis (Riera Romo et al. 2016). However, it has been shown that they also act in cell wall remodeling in bacterial cell expansion/division, in progeny release in the bacteriophage life cycle, and in nutrient acquisition in microbe-rich diets (Jollès and Jollès 1984; López et al. 1997; Hermoso et al. 2003; Korczynska et al. 2010). The participation of lysozyme also extends beyond pathogen control to other biological processes, including development, where it contributes to microbiota remodeling during metamorphosis, as well as digestion (Díaz-Garrido et al. 2026). This wide diversification is related to gene duplication followed by neo-functionalization of new copies, accompanied by regulatory shifts affecting time and local expression (Prager 1996; Long et al. 2003; Zhang 2003; Callewaert and Michiels 2010; Van Herreweghe and Michiels 2012).

One notable example that illustrates this is the recruitment of C-type lysozymes for digestive roles. Their bacteriolytic activity in the alimentary tract was first detected in the bovine stomach by Dobson et al. (1984). Studying the sequences of these lysozymes, Jollès and Jollès (1984) found remarking molecular adaptations to higher resistance to peptidases and to functionality at a low pH in comparison to canonically immune-related lysozymes. Later on, similar cases were also reported for other foregut-fermenting vertebrates, such as the leaf-eating monkey, the hoatzin, and rabbits (Cámara and Prieur 1984; Stewart et al. 1987; Kornegay et al. 1994; Prager 1996). Further investigations lead to the discovery of digestive lysozymes in invertebrates as well. Extensive studies were carried on the brachyceran dipterans *Drosophila melanogaster* (Daffre et al. 1994; Regel et al. 1998) and *Musca domestica* (Lemos and Terra 1991; Cançado et al. 2008; Terra et al. 2019), corroborating the previous finding on molecular adaptations to an acidic digestive environment and shedding light on the evolutionary dynamics of lysozymes. Moreover, experiments with mosquitoes, termites, and bivalve mollusks showed that these enzymes can also act in neutral to alkaline alimentary tracts (see further in Callewaert and Michiels 2010). In the latter, it is noteworthy that the lysozymes that have undergone those adaptations are I-type ones, which are primarily related to immune defense.

Thus far, lysozymes have proved to be highly adaptative in a wide range of organisms with different life-strategy backgrounds. Importantly, digestion and immunity are not independent axes along which lysozyme function can be classified: the gut lumen is itself a major component of the immune barrier, in constant contact with ingested microbes (Dillon and Dillon 2004), and the recruitment of lysozymes for digestion has been proposed as an evolutionary extension of their ancestral immune role rather than a distinct function (Terra et al. 2019). A lysozyme acting on ingested bacteria can therefore simultaneously supply nutrients from microbial biomass and limit bacterial translocation across the gut epithelium. Digestive recruitment should thus be understood as one specific outcome along a continuum of gut-associated antibacterial activity, rather than as a function distinct from immunity. Nevertheless, the extent to which the digestive recruitment has occurred concurrently with the immune function outside well-characterized model systems remains unclear. Besides, the evolutionary implications of co-existence of two lysozyme types in insects have been poorly explored.

In this sense, insects are a timely study object to unravel evolutionary aspects of lysozymes in a large-scale approach. From occupying terrestrial to aquatic and aerial environments, this most successful metazoan group exhibits extraordinarily different lifestyles. Thus, ecological exposure to microbes varies widely and is shaped by multiple interacting factors, including feeding habits, developmental stage, habitat, and social or symbiotic behaviors (Dillon and Dillon 2004; Calderón-Cortés et al. 2012; Crava et al. 2015). Detritivory, scavenging, hematophagy, and sap-feeding expose insects to distinct microbial communities, while some behavioral and ecological patterns, such as social hygiene or reliance on obligate symbionts, may reduce the need for inducible immune defenses (Ursic Bedoya et al. 2005; Cremer et al 2007; Gerardo et al. 2010; Nayduch and Burrus 2017).

Furthermore, previous studies showed that C- and I-types lysozymes are ubiquitously distributed across Insecta phylogeny, even in taxa lacking antimicrobial peptides (AMPs), implying that this enzyme was among the earliest evolutionary adaptations to cope with early immunological challenges (Zhou et al. 2024). What is intriguing about this is that both immune and digestive functions are so far performed only by C-type lysozymes, and not by the I-type as in other invertebrates as nematodes and mollusks, lineages that diverged from arthropods approximately 610 to 730 million years ago (Kumar et al. 2022), despite the retention of I-type lysozymes across this deep divergence (Callewaert and Michiels 2010). In insects, I-type lysozymes appear to have lost their catalytic dyad, and their biological role is still not well understood.

In this study, we aim to explore how ecological and physiological selective pressures underlie the evolutionary dynamics of the lysozyme gene family across Insecta. Specifically, we hypothesized that lysozymes in detritivorous insects may have undergone a similar recruitment for digestive function, while recognizing that such recruitment builds upon, rather than replaces, their ancestral role in gut immunity. Among the different insect feeding strategies, we focus on detritivory because decaying organic matter typically harbors the densest and most consistent bacterial loads among insect food sources, providing the closest ecological parallel to the bacteria-rich digestive precedent already established for Diptera (Lemos and Terra 1991; Regel et al. 1998), and thus offering the strongest a priori expectation for detecting digestive recruitment of lysozyme outside these model species. To test it, we formulated three main questions: (i) Do detritivorous insects harbor a greater number of lysozyme gene copies? (ii) Have insect lysozymes undergone structural or functional adaptations to suit digestive environments? and, (iii) Does lysozyme expression vary according to feeding habits, and is this expression concentrated in gut regions with intense digestive activity, similarly to *D. melanogaster* and *M. domestica*? To address these questions, we: (i) quantified C-type and I-type lysozyme gene copies across the Insecta phylogeny, aiming to detect expansion events associated with ecological traits; (ii) tested the correlation between lysozyme copy number and feeding habits; (iii) described the composition and number of cleavage sites in insect lysozymes targeted by the major digestive peptidases in insect midguts; and (iv) investigated lysozyme expression profile along the digestive tract of five model insects representing distinct orders and ecological niches: *Musca domestica* (Diptera, detritivorous/saprophagous larvae), *Rhodnius prolixus* (Hemiptera, obligate haematophagy), *Tenebrio molitor* (Coleoptera, generalist grain scavenger), *Spodoptera frugiperda* (Lepidoptera, phytophagous), and *Periplaneta americana* (Blattodea, omnivorous scavenger).

## Results

### Evolutionary Analyses of the C-type Lysozyme Gene Family in Insects

All duplication and loss event counts reported below were inferred in this study through gene tree-species tree reconciliation (GeneRax; see Methodology section) and do not represent previously published estimates. The outgroup taxa included here (three crustaceans and the collembolan *Folsomia candida*) were used to root and polarize the phylogeny, following standard practice in gene family reconciliation analyses. Because these four taxa differ substantially from one another in C-type lysozyme copy number, however, we treat the reconstructed ancestral states at the Hexapoda and Insecta nodes as poorly constrained and do not report a specific ancestral copy number for these deepest nodes. Our analysis of gene family dynamics instead focuses on the four holometabolan orders represented by at least 27 species each (Diptera, *n* = 92; Hymenoptera, *n* = 77; Lepidoptera, *n* = 44; Coleoptera, *n* = 27) and on the backbone nodes connecting them (Neoptera, Paraneoptera + Holometabola, Holometabola, Aparaglossata, and Lepidoptera + Diptera). For all remaining lineages, represented by one to five species per order or, in the case of Hemiptera, by 19 species spread across three suborders of a highly diverse order, we report only observed copy numbers. In total, we sampled 275 insect species spanning 13 orders. A highly dynamic pattern of gene copy expansion and contraction is evident across these insect lineages, as described in Fig. 1 (see Tables S1 and S2 for details).

**Fig. 1.**
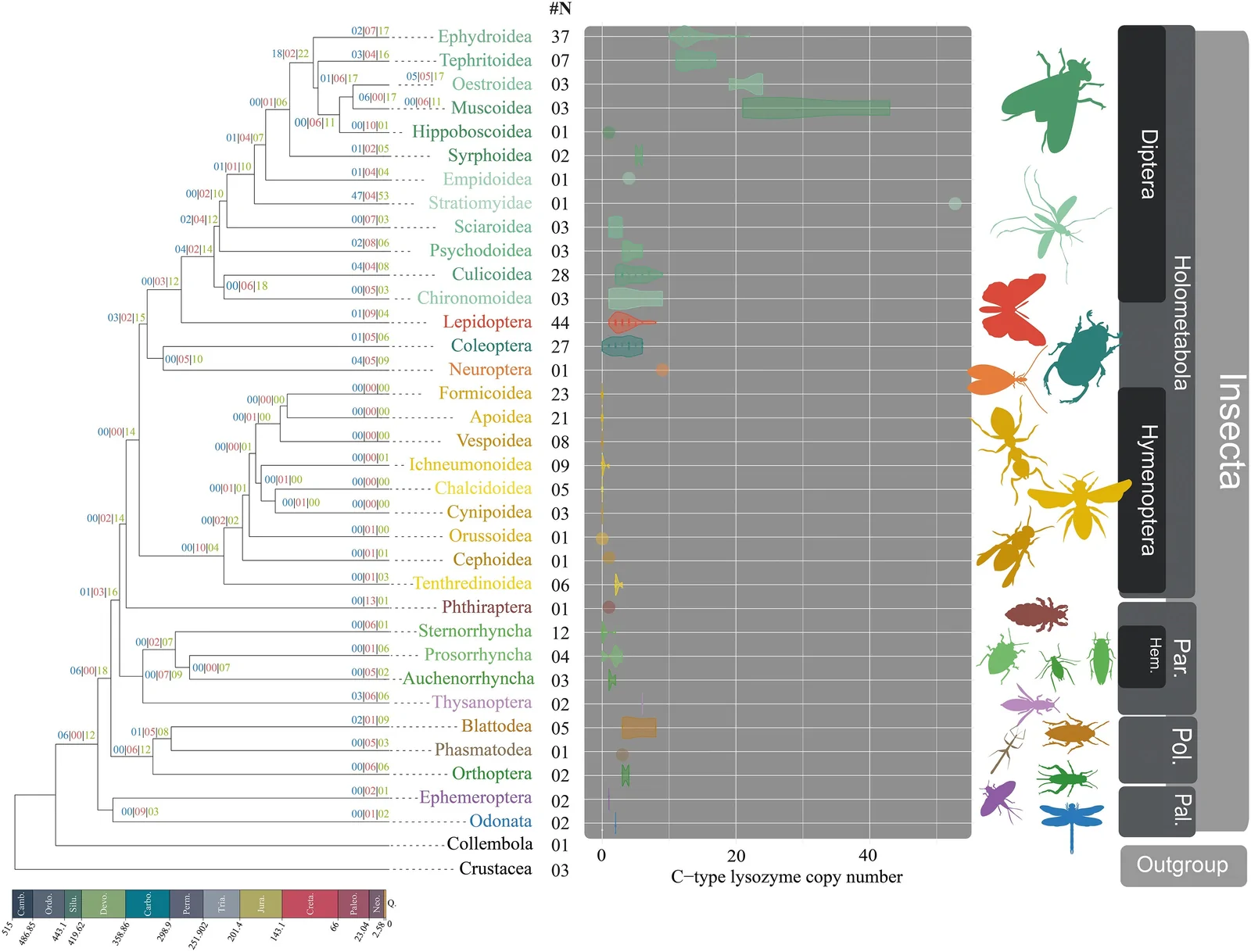
Time-calibrated phylogeny and copy number evolution of C-type lysozyme genes across Insecta. Left, dated phylogeny of the 275 insect species (plus the outgroups Collembola and Crustacea) analyzed in this study, with geological periods indicated below. The time scale represents the geological time periods: Camb., Cambrian; Ordo., Ordovician; Silur., Silurian; Neo., Neogene; Q., Quaternary. Numbers at internal nodes show the inferred number of gene duplication (blue), loss (red), and ancestral copy number (green) events for each lineage; #N indicates the number of species sampled per group. Duplication, loss, and ancestral copy number estimates for sparsely sampled clades (Palaeoptera, Polyneoptera, Paraneoptera, Phthiraptera, and Neuroptera) should be interpreted with caution given the limited taxon sampling in these lineages (see main text for details). Because Diptera, Hymenoptera, and Hemiptera showed the greatest diversification within the group, lineages are shown at the family level for Diptera and Hymenoptera and at the infraorder level for Hemiptera, while other orders are shown as single units. Right, distribution of C-type lysozyme gene copy number across species within each lineage, colored by taxonomic group as depicted by the silhouettes at right; higher-level classification is shown on the far right (Hem., Hemiptera; Par., Paraneoptera; Pol., Polyneoptera; Pal., Palaeoptera). Dots representing lineages with a single sampled species were enlarged for visualization purposes.

Among the Palaeoptera (Odonata + Ephemeroptera) taxa sampled here, the two Odonata species examined (*Hetaerina americana*, Calopterygidae and *Ischnura elegans*, Coenagrionidae) each had two gene copies, while the two Ephemeroptera species examined (*Cloeon dipterum* and *Neocloeon triangulifer*, both from the Baetidae family) each with a single gene copy. Given that only two species represent each of these orders in our dataset, we do not attempt to reconstruct ancestral copy numbers for this clade and report only the observed values.

The Neoptera ancestor, which gave rise to the vast majority of sampled species across Polyneoptera, Paraneoptera, and Holometabola, is inferred to have had 18 C-type lysozyme copies. From this ancestral state, copy number varied extensively among its constituent superorders through lineage-specific expansions and contractions, as described below.

Within Polyneoptera (Orthoptera + Phasmatodea + Blattodea, among others), taxon sampling is limited (one to five species per order), so we report only the observed copy numbers rather than reconstructed ancestral states for this clade. Orthoptera species had three (*Anabrus simplex*, Tettigoniidae) and four (*Gryllus longicercus*, Gryllidae) gene copies. The single samples Phasmatodea species (*Bacillus rossius redtenbacheri*, Bacillidae) exhibited three copies. Within Blattodea, copy number ranged from three to eight across the five species sampled: the cockroaches *Blattella germanica* (Blattellidae) and *Periplaneta americana* (Blattidae) had five and eight copies, respectively, while the termites *Zootermopsis nevadensis* (Termopsidae) and *Coptotermes formosanus* (Rhinotermitidae) displayed three copies, and *Cryptotermes secundus* (Kalotermitidae) had eight, which is a notable expansion related to its termite relatives.

Following the divergence of Polyneoptera, the last common ancestor of Paraneoptera (Hemiptera + Thysanoptera + Psocodea) and Holometabola (Hymenoptera, Neuroptera, Coleoptera, Lepidoptera, Diptera, and allies) is inferred to have had 16 C-type lysozyme gene copies, consistent with the elevated ancestral estimate for Neoptera as a whole (18 copies) and the subsequently high copy numbers observed within Holometabola.

Paraneoptera, represented in our work by Hemiptera and Thysanoptera, is another clade with sparse taxon sampling relative to its diversity, so we again report only observed copy numbers. In Thysanoptera, both sampled species (*Thrips palmi* and *Frankliniella occidentalis*, both from the Thripidae family) showed six copies. Within Hemiptera (*n* = 19), copy number varies markedly among suborders: in Sternorrhyncha (*n* = 12), the gene was found only in *Bemisia tabaci* (Aleyrodoidea) (two copies), while all other sampled Sternorrhyncha species (Aphidomorpha and Coccoidea) have lost it entirely; in Auchenorrhyncha (*n* = 03), the single Cicadomorpha species (*Homalodisca vitripennis*, Cicadellidae) exhibited two copies, while the two sampled Fulgoromorpha species (*Nilaparvata lugens* and *Laodelphax striatellus*, both Delphacidae) had only one gene copy; and in Heteroptera, copy number ranged from complete loss (*Halyomorpha halys*, Pentatomidae) to three copies (*Rhodnius prolixus*, Reduviidae) and two copies each in the Cimicoidea species (*n* = 02) *Cimex lectularius* (Cimicidae) and *Apolygus lucorum* (Miridae).

For Phthiraptera, the single sampled species, *Pediculus humanus corporis* (Pediculidae), had only one C-type lysozyme copy. We do not attempt to place this observation within an ancestral-state framework: Phthiraptera is represented by a single species here, and its phylogenetic position relative to Holometabola remains contested (Friedemann et al. 2014; Misof et al. 2014), which would itself change any inferred ancestral count.

Regarding Holometabola (or Endopterygota), represented in this study by the orders Hymenoptera (*n* = 77), Neuroptera (*n* = 01), Coleoptera (*n* = 27), Lepidoptera (*n* = 44), and Diptera (*n* = 92), the ancestor of this clade is inferred to have had 14 C-type lysozyme gene copies, comparable to the ancestral estimate for the preceding Paraneoptera + Holometabola node (16 copies). Within Hymenoptera, this lineage underwent 10 gene losses in its ancestral state, followed by lineage-specific loss events across Orussidae (*n* = 01) and Proctotrupomorpha (*n* = 08), and a complete loss in Ichneumonoidea (*n* = 09) + Aculeata (*n* = 52). These cumulative losses ultimately led to the complete absence of C-type lysozyme copies in all Hymenoptera lineages, with the sole exceptions of Tenthredinoidea and Cephoidea, which harbored an average of two C-type lysozyme copies, and *Chelonus insularis* (Ichneumonoidea: Braconidae), with one copy.

After the divergence of Hymenoptera, the Aparaglossata ancestor is inferred to have had 15 copies, following three duplications and two losses. A subsequent reduction to 12 copies is inferred for the ancestor of Lepidoptera + Diptera, both among the most densely sampled orders in our dataset. The corresponding estimate for the Coleoptera + Neuroptera ancestor (ten copies, following five losses) should be interpreted with more caution, as Neuroptera is represented here by a single species.

The single sampled Neuroptera species, *Chrysoperla carnea* (Chrysopidae), had nine C-type lysozyme copies. Within Coleoptera, the evolutionary history of C-type lysozymes exhibits substantial variation. The Coleoptera ancestor underwent five losses, reducing the ancestral gene repertoire to six copies. Throughout the phylogeny, gene copy number patterns were highly variable, with copy numbers ranging from six to one copy across multiple lineages, reflecting additional lineage- and species-specific loss events, including complete gene loss in *Ignelater luminosus* (Elateridae) and *Diorhabda carinulata* (Chrysomelidae).

Following the divergence from Diptera, a significant number of losses (nine) were predicted in the last common ancestor of Lepidoptera, leaving only four gene copies. After this, a dynamic evolutionary trajectory was shaped by lineage- and species-specific events. These processes resulted in relatively low copy numbers of C-type lysozyme across most Lepidoptera lineages. However, this pattern probably has arisen through convergent evolution, as the gene loss events might have occurred independently along the branches. A notable example is found in Nymphalidae (*n* = 11), where, despite multiple species-specific gene losses, all sampled members had two copies. Notable exceptions to the general trend were observed in specific lineages with lineage-specific duplications, such as those that produced the eight gene copies in *Parnassius apollo* (Papilionidae).

Diptera is the most well-sampled order in this study, represented by 92 species across eight families. The common ancestor of Diptera is inferred to have arisen from 14 gene copies (following four duplications and two losses), initiating a highly dynamic evolutionary trajectory within the group. A general pattern is observed, with relatively low copy numbers of C-type lysozyme in Nematocera (*n* = 37), resulting from convergent lineage-specific losses in Culicomorpha (*n* = 31), Psychodidae (*n* = 03), and Sciaroidea (*n* = 03), whereas Brachycera (*n* = 55) species had high copy numbers. A particularly striking case is *Hermetia illucens* (Brachycera: Stratiomyomorpha), which underwent 47 duplications and four losses, resulting in a remarkable 53 copies of C-type lysozyme, the most extensive repertoire identified in this study. Another highly eventful node is found within Muscoidea, where each sampled species experienced extensive species-specific duplications, harboring numerous copies. The number of copies in this family ranged from 21 in *Musca vetustissima* (nine duplications, five losses) to 43 in *Musca domestica* (29 duplications, three losses).

### Evolution of the I-type Lysozyme Gene Family

The evolutionary dynamics of gene duplication and loss events were also assessed for I-type lysozymes (Fig. 2, see Tables S3 and S4 for details). As with C-type lysozymes, we restrict ancestral-state inferences to the four densely sampled holometabolan orders and the backbone nodes connecting them, and report observed values for all other clades.

**Fig. 2.**
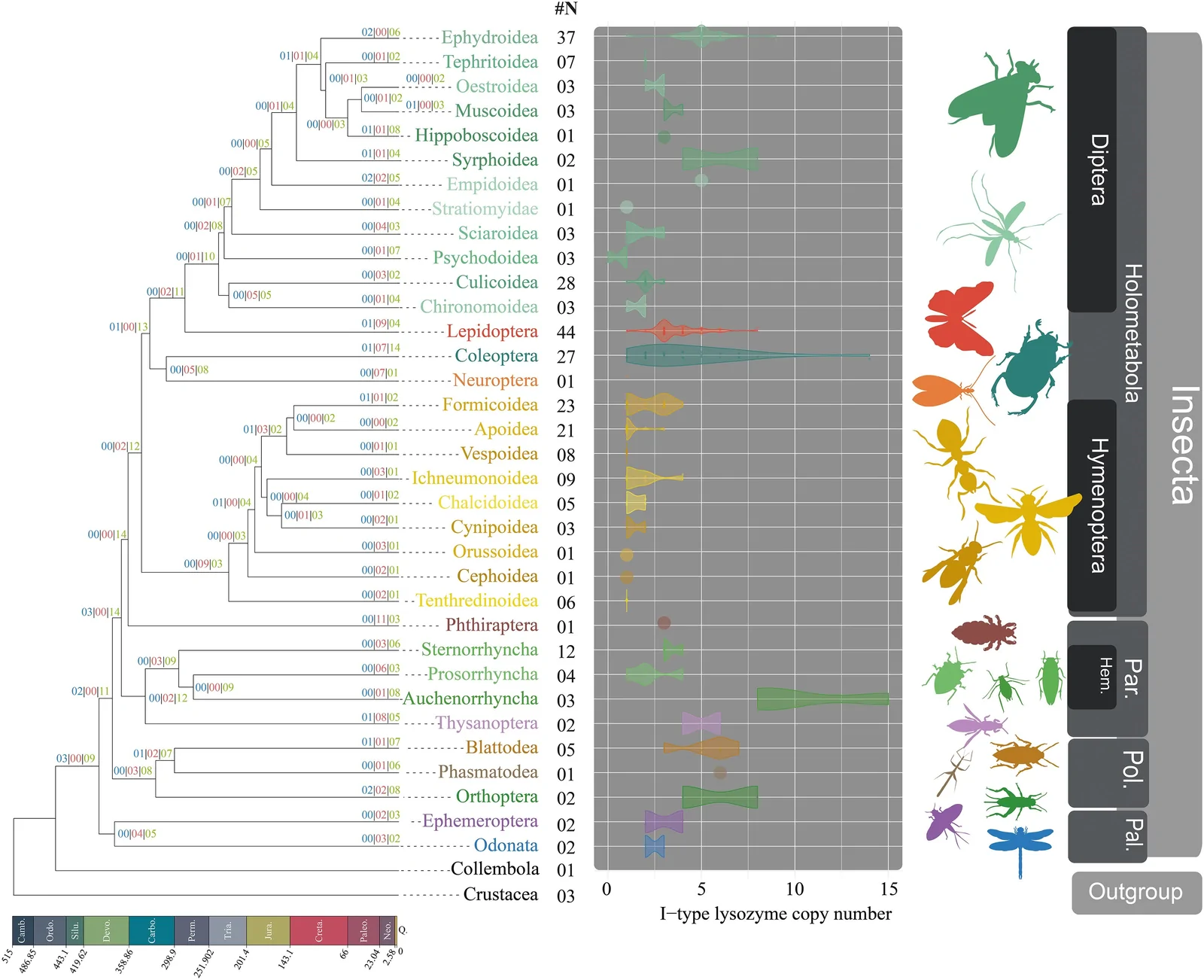
Time-calibrated phylogeny and copy number evolution of I-type lysozyme genes across Insecta. Left, dated phylogeny of the 275 insect species (plus the outgroups Collembola and Crustacea) analyzed in this study, with geological periods indicated below. The time scale represents the geological time periods: Camb., Cambrian; Ordo., Ordovician; Silur., Silurian; Neo., Neogene; Q., Quaternary. Numbers at internal nodes show the inferred number of gene duplication (blue), loss (red), and ancestral copy number (green) events for each lineage; #N indicates the number of species sampled per group. Duplication, loss, and ancestral copy number estimates for sparsely sampled clades (Palaeoptera, Polyneoptera, Paraneoptera, Phthiraptera, and Neuroptera) should be interpreted with caution given the limited taxon sampling in these lineages (see main text for details). Because Diptera, Hymenoptera, and Hemiptera showed the greatest diversification within the group, lineages are shown at the family level for Diptera and Hymenoptera and at the infraorder level for Hemiptera, while other orders are shown as single units. Right, distribution of I-type lysozyme gene copy number across species within each lineage, colored by taxonomic group as depicted by the silhouettes at right; higher-level classification is shown on the far right (Hem., Hemiptera; Par., Paraneoptera; Pol., Polyneoptera; Pal., Palaeoptera). Dots representing lineages with a single sampled species were enlarged for visualization purposes.

Among the Palaeoptera taxa sampled here, the two Odonata species examined exhibited three and two gene copies, respectively, while the two Ephemeroptera species examined had four and two copies. The Neoptera ancestor, in contrast, is inferred to have had 11 I-type copies, broadly consistent with the elevated ancestral estimate found for C-type lysozymes at this same node. Within the sparsely sampled Polyneoptera clade, I-type copy number varied considerably among species: four and eight copies in the two Orthoptera species, six copies in the single Phasmatodea species, five and seven copies in the two cockroach species, and three, six, and six copies in the three termite species.

Within Paraneoptera, Hemiptera stands out, particularly the Auchenorrhyncha group, which possesses the highest number of I-type lysozymes among the species sampled here. *Homalodisca vitripennis* showed eight copies, while the two Fulgoromorpha species *Laodelphax striatellus* and *Nilaparvata lugens*, exhibited nine and 15 copies, respectively. This makes *N. lugens* the insect species with the highest number of I-type lysozyme gene copies identified in this study. In contrast, the sampled Heteroptera species had between one and four gene copies each, and the sampled Stenorrhyncha species had three or four copies each.

Within Holometabola, unlike C-type lysozymes, all hymenopteran species had at least one copy of the I-type lysozyme gene. Following their divergence from a common ancestor, which is inferred to have possessed three copies of this gene (following nine losses), several lineage-specific losses and duplications occurred, resulting in one to four copies of I-type lysozyme genes.

Coleoptera is inferred to have begun with 14 gene copies in its last common ancestor (following seven duplications and one loss) and showed a highly variable number of copies among the species analyzed in our work. The group underwent several lineage- and species-specific duplications throughout the clade, as well as multiple lineage-specific losses in Curculionoidea (*n* = 05) and Chrysomelidae (*n* = 06). Notably, Coleoptera also harbors the species with the second-highest number of I-type lysozyme genes, *Zophobas morio* (Tenebrionidae), which has 14 copies, a number higher than its closest relative in our analysis, *Tenebrio molitor*, from the same family, which also has a considerable number of I-type gene copies (ten).

The order Lepidoptera exhibited a relatively stable composition, with most species harboring three to eight copies of I-type lysozymes, not far from the number of copies inferred for their last common ancestor, which possessed four copies derived from eight losses and one duplication.

The evolutionary dynamics of I-type lysozyme gene copy number in Diptera reveal distinct patterns across different lineages. The Culicomorpha infraorder exhibits a conserved profile, with most species having around two copies. Similarly, reduced copy numbers are observed in Psychodomorpha and in Tephritoidea (Acalyptratae), where most species had two copies of the I-type lysozyme gene. In contrast, other Acalyptratae lineages, represented by Ephydroidea (Drosophilidae), display a more complex pattern characterized by a greater number of I-type copies. This expansion traces back to an ancestral state of six gene copies (two duplications) in their last common ancestor, followed by lineage- and species-specific expansions and contractions. A notable exception within this group is *Drosophila arizonae*, which appears to have only one I-type copy. Notably, the flies with the highest numbers of C-type lysozyme copies, *M. domestica* (43) and *H. illuscens* (53), have only four and one copy of the gene coding for I-type lysozyme, respectively.

### I-type Sequences Profile

Docking simulations were performed for the most highly expressed I-type lysozyme sequence identified in each of the five RNA-seq model species (Methods 2.5), together with the *Meretrix lusoria* lysozyme structure (PDB ID: 3AB6), used as a positive control due to its well-characterized binding interface (Kuwano et al. 2013), to assess whether these active-site-deficient insect sequences retained potential binding interactions with the NAG3 substrate. All five insect sequences exhibited favorable binding energies for NAG3, as indicated by HADDOCK scores ranging from −63.6 to −57.2 in the top-ranked clusters, close to the −68.3 score observed for the *Meretrix* structure, suggesting that substrate affinity may be partially conserved despite the loss of catalytic residues (Table S5).

Furthermore, most of the sequences analyzed by AMP Scanner were classified as potential antimicrobial peptides (Tables S6 and S7). For the C-type sequences, 155 out of 1,326 (11.68%) were not predicted as AMPs. Among the I-type sequences, only 10 out of 876 (1.14%) were not predicted as AMPs.

### Phylogenetic Signal

The best-fitting evolutionary models, according to the AICc criterion, were the Ornstein-Uhlenbeck (OU1) model for I-type lysozymes and the Brownian Motion with variable rates (BMS) model for C-type lysozymes, suggesting the presence of an optimal state governing I-type lysozyme copy-number evolution and heterogeneous rates of diversification among C-type lysozymes across insect lineages (Table S8). Phylogenetic signal analyses further supported these differences, revealing stronger phylogenetic structuring for C-type lysozymes than for I-type lysozymes (Table S8). The correlograms based on cophenetic distance showed significant positive autocorrelation at short to intermediate distances for both lysozyme types, followed by an overall decline in Moran's I estimates with increasing distance, reaching significantly negative values at a distance of 800. For I-type lysozymes, a local peak was observed at a cophenetic distance of 600, coinciding with the split between Nematocera and Brachycera, which may point to a localized increase in trait similarity around this divergence event (Fig. S1).

To investigate potential associations between insect feeding habits and lysozyme gene copy number, Phylogenetic Generalized Least Squares (PGLS) regressions were performed separately for C-type and I-type lysozymes, accounting for phylogenetic non-independence among species according to the evolutionary models with best fit (Table S9).

Overall, few statistically significant relationships were detected. However, the analyses revealed some associations between diet and lysozyme copy number. A detritivorous diet showed a weak, marginally significant positive correlation with C-type lysozyme copy number in larvae (coefficient = 0.6731; *P* = 0.047), which was not replicated in adults (*P* = 0.081). An omnivorous diet showed a more consistent positive association with I-type lysozyme copy number, significant in both adults (coefficient = 0.273; *P* = 0.012) and larvae (coefficient = 0.277; *P* = 0.011). No other dietary categories were consistently associated with variation in lysozyme copy number for either lysozyme type.

Finally, to assess whether dietary shifts were associated with sequence-level adaptations, we tested, we quantified, for every lysozyme sequence in the filtered dataset, the number of predicted cleavage sites for the digestive peptidases chymotrypsin, papain, pepsin, and trypsin, and tested whether the per-species average number of cleavage sites (reflecting susceptibility to digestion by the host's own proteases) varied with feeding habit using PGLS models. No significant associations were detected for any of the evaluated peptidases (chymotrypsin, papain, pepsin, or trypsin), indicating that variation in cleavage-site number is not explained by insect feeding strategies (Table S10).

### Gene Expression Profiles Across Digestive Tissues

To evaluate whether expression patterns are consistent with a digestive role, we quantified *in silico* expression (transcripts per million [TPM]) of C-type and I-type lysozyme genes from published RNA-seq datasets from our group (Dias et al. 2019) across digestive and carcass tissues of five model species.

In *M. domestica*, the highest lysozyme expression levels were observed in the anterior sections of the larvae's midgut, particularly in segments AM2, MM1, and AM1, respectively (Table S11). Notably, two C-type lysozyme genes (XM_059127591.1 and XM_059127592.1) together accounted for nearly one-third of all lysozyme transcripts detected in this species. These genes exhibited identical TPM values, likely due to their recent duplication and complete sequence identity, which leads to multi-mapping of reads. As a result, it is not possible to determine which of the two genes is more highly expressed. It is also noteworthy that the well-characterized lysozyme 1 of *M. domestica* (NP_001295901.1) (Cançado et al. 2008) showed high expression in AM2, MM1, and AM1, respectively, although it was not among the most abundantly expressed lysozyme genes in our dataset. In contrast, the I-type lysozyme exhibited its highest expression in the carcass.

Similarly, in *Rhodnius prolixus* adults (Table S12) and *Tenebrio molitor* larvae (Table S13), AM showed the most significant expression of C-type lysozymes. However, the I-type lysozymes in both species were predominantly expressed in the carcass.

In *Periplaneta americana* adults, lysozyme expression was most abundant in the salivary glands, followed by the carcass and gastric cecum (Table S14). Nevertheless, some gene copies exhibited distinct patterns: XP_069673831.1 showed elevated expression in the midgut, and XP_069700793.1 showed elevated expression in the hindgut. Additionally, I-type lysozymes showed higher collective expression in the foregut region.

For *Spodoptera frugiperda*, the carcass was not sampled. However, within the midgut regions, C-type lysozyme expression was higher in the posterior segment, whereas I-type lysozymes were more expressed in the anterior portion (Table S15).

## Discussion

### Evolutionary Dynamics in the Number of Copies of Genes Coding C-type Lysozymes

Reconciliation analyses indicate that C-type lysozyme genes were already established early in insect evolution, present even in the most early-diverging lineages sampled here (Palaeoptera), suggesting that these enzymes played an important functional role from the earliest stages of insect diversification. The ancestral insect is thought to have occupied a terrestrial habitat and fed on a generalist diet, mainly decaying organic matter associated with foliage (Calderón-Cortés et al. 2012; Engel 2015), an ecological context consistent with regular exposure to microbe-rich substrates and with reliance on an inducible antibacterial response in which lysozyme plays a role (Marra et al. 2021). This is consistent with a long-term association between lysozyme and the insect immune system, among its more ancestral components in Hexapoda; we do not, however, attempt to pinpoint a precise ancestral copy number for this deep node, given the limited and heterogeneous outgroup sampling discussed in the Results.

Interestingly, low lysozyme gene copy numbers are observed in lineages with alternative immune strategies. In Palaeoptera (eg dragonflies and mayflies), the comparatively low C-type lysozyme gene copy number observed may be consistent with reduced reliance on inducible antibacterial defenses, potentially related to predatory or filter-feeding habits and life stages with little or no feeding, which limit direct exposure to microbe-rich substrates, as well as to alternative physical and chemical antimicrobial strategies documented in Odonata, such as bactericidal nanostructures on dragonfly wings (Bandara et al. 2017; Combey et al. 2025). Given that this clade is represented by only four species in our dataset, these interpretations remain speculative and would benefit from broader taxonomic sampling.

The evolutionary patterns observed in Polyneoptera appear relatively stable in some orders: the two Orthoptera species sampled had similar copy numbers (three and four). Phasmatodea is represented by a single species in our dataset, precluding within-order comparison. Nonetheless, lineage-specific deviations become apparent at deeper nodes of the tree, particularly within Blattodea.

Within Blattodea, elevated C-type lysozyme copy numbers are observed in the cockroaches *B. germanica* and *P. americana* (five and eight copies, respectively), as well as in the termite *C. secundus* (eight copies), which is markedly higher than in the other sampled termites, *Z. nevadensis* and *C. formosanus* (three copies each). The two cockroach species are synanthropic insects that are frequently exposed to diverse bacterial communities through their scavenging in human-associated environments, which are known to harbor a broad range of microbial strains (Kakooza et al. 2025). In this context, the observed expansion of lysozyme gene copies may be consistent with increased microbial exposure, although a direct adaptive link cannot be established. Supporting this interpretation, *P. americana* has been reported to possess an immune repertoire enriched in antimicrobial peptides with broad antibacterial activity (Kim et al. 2016). Moreover, extracts derived from *P. americana* have been shown to exhibit antimicrobial effects in applied contexts, such as improving animal growth performance and antioxidant capacity, without synthetic additives (Liu et al. 2025).

In contrast, the expansion of C-type lysozyme copies in *C. secundus* may be associated with nutritional constraints rather than solely immune-related pressures. Termites feed primarily on cellulose-rich substrates that are extremely poor in nitrogen, requiring alternative strategies for nitrogen acquisition. In this species, nitrogen can be obtained through the digestion of microbial biomass transferred via proctodeal trophallaxis (Brune 2014). Additionally, the one-piece nesting behavior of *C. secundus*, which restricts feeding to a single log and limits dietary diversity (Monroy Kuhn et al. 2019), may further increase reliance on internal nutrient recycling. Under this scenario, an elevated number of lysozyme copies could facilitate microbial turnover and nutrient recycling within the colony, although this hypothesis remains to be directly tested.

Remarkable evolutionary patterns within Paraneoptera are concentrated in the Aphidomorpha clade, where all sampled species have lost C-type lysozyme entirely. This loss may be associated with the highly specialized phloem-sap diet of aphids, their firm reliance on symbiotic bacteria for immune defense, and adaptive life-history strategies such as terminal reproduction, in which individuals prioritize reproductive output over survival when facing immune challenges, investing in offspring production rather than in energetically costly immune responses (Gerardo et al. 2010).

In contrast, the relatively high C-type lysozyme copy number observed in Thripinae (six copies in both sampled species, *T. palmi* and *F. occidentalis*) may be linked to immune functions distinct from antibacterial defense. In this group, lysozyme transcripts are upregulated during the early stages of infection by Tomato spotted wilt virus in *Frankliniella* species, suggesting a role in constitutive or rapidly inducible antiviral responses (Shrestha et al. 2017). Consistent with this hypothesis, antiviral activity of C-type lysozymes has been reported in vertebrates and Lepidoptera, where these enzymes can inhibit viral replication through hydrolytic mechanisms (Chen et al. 2018). Taken together, these observations suggest that the relatively high copy number in Thripinae may contribute to enhanced antiviral defense, although functional validation in Paraneoptera remains necessary, and only two species were sampled for this subfamily.

Phthiraptera is represented by a single species in our dataset, *Pediculus humanus corporis*, which had a single C-type lysozyme copy, among the lowest counts observed in species in which the gene was detected. We discuss it here, before Holometabola, without assuming a specific sister–group relationship, since the phylogenetic placement of Phthiraptera relative to Holometabola remains contested (Friedemann et al. 2014; Misof et al. 2014). This reduced repertoire is consistent with an overall pattern of immune gene family contraction previously reported for this species, including the complete loss of the Imd pathway (Kim et al. 2011), plausibly related to its host-restricted, blood-feeding lifestyle. However, the PGLS analysis did not detect a significant association between blood-feeding and lysozyme copy number across the broader dataset, and this single-species observation should be interpreted with caution.

Among Holometabola, the most remarkable event is found in Hymenoptera, where the complete absence of C-type copies is present in Ichneumonoidea + Aculeata. This absence of C-type lysozymes in many Hymenoptera species may be associated with the evolution of social immunity mechanisms (Cremer et al. 2007). However, it has also been proposed that the reduced lysozyme repertoire could represent an ancestral condition that facilitated the evolution of social immunity, rather than sociality driving gene loss (Barribeau et al. 2015). This interpretation is further supported by the observation that social immunity does not, in itself, protect against bacterial infections; instead, it involves proactive hygienic behaviors, such as those observed in ants, that help prevent bacterial exposure and infection (Masson et al. 2024). Within Holometabola, elevated copy number is also observed in the single sampled Neuroptera species, *C. carnea*, consistent with this species’ documented resistance to entomopathogenic bacteria such as *Pseudomonas protegens* (Hamze et al. 2022); as with Phthiraptera, however, this single-species pattern awaits confirmation from broader taxonomic sampling.

Coleoptera exhibited considerable variability in lysozyme gene copy number, reflecting its extensive ecological diversity and broad adaptive range (Beutel et al. 2026). In contrast, comparative analysis of C-type and I-type lysozymes across three ladybird beetle species (Coccinellidae) found comparatively stable lysozyme copy numbers despite a highly dynamic expansion of AMP-encoding genes within this family, suggesting that antimicrobial gene family diversification in some Coleoptera lineages may proceed predominantly through AMP expansion rather than lysozyme duplication (Vogel et al. 2017b). *Nicrophorus vespilloides* (Silphidae), which had six C-type lysozyme copies, one of the higher counts observed within Coleoptera, may owe this expansion to its reproductive reliance on decaying carcasses. Although these substrates are maintained under relatively controlled microbial conditions, previous studies have shown that this is mediated mainly by the activity of immune-related genes expressed by *N. vespilloides* itself (Vogel et al. 2011). In particular, lysozymes and other antimicrobial peptides were found to be upregulated in the foregut and midgut, suggesting roles in carcass sanitation, regulation of gut microbiota, and bacterial digestion (Vogel et al. 2017a).

Many other coleopterans exhibit predominantly phytophagous or predatory lifestyles, which are generally associated with lower exposure to microbe-rich substrates. In predatory species, lysozyme gene repertoires may instead be shaped by trophic interactions with their prey, particularly Sternorrhyncha (eg aphids), which represent a major and recurrent prey group for several beetle lineages. These prey insects harbor abundant bacterial symbionts, and experimental evidence indicates that immune-related genes in coleopteran predators are downregulated when alternative, symbiont-poor food sources are provided, supporting the idea that lysozyme expression is at least partly modulated by prey-associated microbiota (Huang et al. 2025).

A similar perspective may extend to Lepidoptera, as all sampled species are phytophagous, which partly accounts for the relative stability observed in lysozyme copy numbers and for the gene loss events inferred at deeper ancestral nodes. Nevertheless, Lepidoptera tend to exhibit a somewhat broader lysozyme gene repertoire than other phytophagous insect groups, a pattern that could be related to their generally less specialized feeding habits. Such dietary breadth may be associated with differences in microbial exposure, although a direct link to immune demand cannot be inferred from copy-number data alone. Additionally, the ingestion of large quantities of plant material by lepidopteran larvae may result in greater bacterial intake than in piercing-sucking insects, as previously suggested (Crava et al. 2015).

Despite this overall pattern, lineage-specific duplication events are sporadically distributed across Lepidoptera, with a notable expansion observed in *Parnassius apollo*. This expansion may be influenced by the unusually large genome size of this species, which has been attributed primarily to the proliferation of transposable elements (Podsiadlowski et al. 2021). Because transposable elements can promote gene duplication, this genomic context may inflate estimates of lysozyme copy number. Therefore, functional characterization will be necessary to determine whether these additional copies correspond to active genes or represent pseudogenized sequences.

Among the dipterans analyzed here, two broad patterns can be distinguished: a comparatively stable and reduced repertoire of C-type lysozymes in Nematocera, and a more diversified set in Brachycera. In mosquitoes, the observed lysozyme repertoire appears consistent with the canonical roles typically attributed to these enzymes, with no clear signatures of lineage-specific specialization associated with their ecology. This pattern may be related to the relatively low microbial load of their primary food sources, namely, blood and nectar in the adult stages. In contrast, during the larval stage, several nematoceran species occupy detritivorous niches in forest litter, where they are exposed to a substantially more diverse and abundant microbial community. This ecological shift likely imposes distinct selective pressures, potentially favoring the maintenance or expansion of a broader lysozyme repertoire in larvae due to their closer association with microbe-rich substrates (Terra et al. 2019). In fact, mosquitoes exhibit a larger lysozyme repertoire than other insects that rely on similarly constrained diets, such as *Pediculus humanus corporis* and aphids. Alternative explanations for this difference include the role for lysozymes during metamorphosis, where they may limit bacterial proliferation during this physiologically vulnerable stage (Ursic Bedoya et al. 2005), and their obligate reliance on a gut microbiota acquired from the aquatic larval environment during feeding (Coon et al. 2016), implying that lysozyme may be involved in regulating these ingested bacteria.

Notwithstanding this pattern, Brachycera show greater heterogeneity in lysozyme copy number, potentially reflecting differences in ecological preferences and life-history traits, notable expansions were detected in the Muscoidea clade and in *Hermetia illucens*. Within Muscoidea, the elevated number of lysozyme genes observed in *Musca* species may be associated with dietary exposure to bacteria-rich substrates. Previous studies have linked lysozymes in these flies to digestive processes in the anterior midgut, where they appear to contribute both to defense against ingested bacteria and to the utilization of microbial biomass as a nutritional resource (Lemos and Terra 1991; Daffre et al. 1994; Regel et al. 1998). In *Stomoxys calcitrans*, however, the expansion of lysozyme copies may not be fully explained by digestive functions alone. Additional selective pressures, such as the need to limit microbial growth in stored blood meals or to cope with the microbe-rich environments of larval developmental substrates, may also have contributed to this pattern (Olafson et al. 2021).

*Hermetia illucens* exhibited the highest number of inferred lysozyme duplications in the dataset. This pattern may be linked to its detritivorous feeding habits and frequent exposure to decomposing organic matter (Bonelli et al. 2019). Consistent with this interpretation, *H. illucens* has been extensively investigated as a source of AMPs with potential applications as alternatives or complements to conventional antibiotics (Scieuzo et al. 2023). Moreover, diets enriched with black soldier fly biomass have been shown to enhance immune responses in aquaculture species (Xiao et al. 2018), reflecting *H. illucens*’ capacity to regulate the expression of lysozymes and other AMPs under both basal and induced conditions (Herman et al. 2024).

Although primarily associated with fruit-based diets, species of the genus *Drosophila* also conform to the general brachyceran pattern. These flies exhibit physiological adaptations to acidic environments that enhance lysosomal digestive efficiency (Regel et al. 1998), and their fermenting food substrates are typically rich in microbial communities. As a result, bacteria present in these environments may readily colonize the drosophilid gut. In this context, lysozymes may contribute not only to digestion but also to the regulation of gut microbiota, as suggested by increased community stochasticity observed in flies carrying lysozyme-deficient mutations (Marra et al. 2021).

Altogether, the evolution of C-type lysozyme copy number in Insecta appears to be shaped predominantly by species-specific ecological contexts rather than by broad, lineage-wide dietary transitions. This interpretation is supported by our PGLS analyses, which identified only isolated, weak associations between C-type lysozyme copy number and dietary categories rather than a broad or consistent pattern, indicating that no single ecological factor alone explains the observed variation and that multiple interacting environmental, physiological, and genomic factors are likely involved. Together with the highly lineage- and species-specific pattern of gains and losses described throughout this section, this result is more consistent with a birth-and-death model of gene family evolution (Nei et al. 1997), in which duplication and loss occur largely independently of any single, consistent selective driver, than with a scenario of pervasive, diet-driven adaptive expansion across Insecta. This does not preclude an adaptive role for lysozyme copy number in specific lineages with independent supporting evidence, such as *M. domestica*, but it cautions against generalizing such a role across the class as a whole.

Nonetheless, the analyses revealed a weak yet consistent tendency among detritivorous species, particularly at the larval stage, to exhibit higher numbers of C-type lysozyme copies. This association is consistent with previous observations in Cyclorrhapha, where detritivory has been linked to expanded lysozyme repertoires (Terra et al. 2019). This trend may have deeper evolutionary roots within Diptera, potentially arising early in Brachycera, given the extensive duplication events inferred in the *H. illucens* lineage.

### Implications of the I-type Lysozyme Genes in Insecta

The canonical role of I-type lysozymes in invertebrates is classically associated with muramidase activity mediated by a double-displacement mechanism, in which conserved aspartic acid and glutamic acid residues act as the nucleophile and proton donor, respectively. This mechanism catalyzes the hydrolysis of the β-1,4-glycosidic bond between N-acetylmuramic acid and N-acetylglucosamine (NAG) residues in bacterial peptidoglycan (Van Herreweghe and Michiels 2012). Notably, all insect I-type lysozyme sequences identified to date lack these catalytic residues, which are conserved in other invertebrates such as mollusks (Xue et al. 2010). This observation suggests that insect I-type lysozymes may not mediate bacterial lysis through the canonical enzymatic mechanism described for other taxa (Beckert et al. 2016).

Despite the apparent loss of catalytic residues, our analyses revealed that I-type lysozyme genes are ubiquitously distributed across the Insecta phylogeny, indicating long-term retention and suggesting that these genes remain functionally relevant. This pattern is particularly striking in Sternorrhyncha (aphids, whiteflies, and scale insects), where most sampled species have lost C-type lysozyme entirely, yet all retain three or four I-type copies, suggesting that I-type lysozymes may compensate for, or operate independently of, the loss of C-type-mediated antibacterial defense in this group. Consistent with this interpretation, AMP Scanner predictions classified almost all insect I-type lysozymes as potentially antimicrobial, and docking simulations revealed interaction energies comparable to those observed for the catalytically active I-type lysozyme from the mollusk species *Meretrix lusoria*. Together, these results suggest that insect I-type lysozymes may retain effective substrate-binding capabilities, even in the absence of classical muramidase activity.

Such apparent functionality may be explained by non-enzymatic antimicrobial mechanisms previously described for I-type lysozymes in other invertebrates. For example, mud crab I-type lysozymes have been shown to bind bacterial surfaces, promote agglutination, disrupt membrane integrity, and induce lattice formation through cross-linking of cell wall components, independently of enzymatic cleavage (Zhou et al 2017), as explained by Ragland and Criss (2017). Similar binding-mediated or membrane-disruptive functions could therefore underlie the antimicrobial activity of insect I-type lysozymes.

In addition to antibacterial roles, insect I-type lysozymes may also have acquired antiviral functions. Recent evidence suggests that these proteins can function as peptidases that hydrolyze peptide bonds at lysine residues within the S2 domain of the Rice gall dwarf virus Pns9 protein, indicating a potential dual role in insect immunity (Du et al. 2026). In this context, the extensive duplication of I-type lysozyme genes observed in the brown planthopper *Nilaparvata lugens* (Hemiptera: Delphacidae), a monophagous herbivore feeding on rice sap and the species with the highest number of I-type copies in our dataset, may be related to selective pressures imposed by interactions with plant-associated viruses. However, functional validation will be required to test this hypothesis directly and to determine the extent to which antiviral activity contributes to the retention and expansion of I-type lysozyme gene families in insects.

In addition to these lineage-specific patterns, our PGLS analyses also revealed a weak but significant positive association between an omnivorous diet and I-type lysozyme copy number, consistent across both adult (coefficient = 0.273; *P* = 0.012) and larval (coefficient = 0.277; *P* = 0.011) stages. Omnivorous insects are exposed to a broader and more heterogeneous range of dietary substrates and associated microbiota than more specialized feeders, which could favor the retention of a larger I-type lysozyme repertoire capable of responding to varied bacterial and, potentially, viral challenges. However, given the number of dietary categories tested, this association should be interpreted with caution, and further work directly testing the functional relevance of I-type copy number in omnivorous lineages would help clarify whether this pattern reflects a genuine ecological signal.

### Putative Adaptive Features of Insect Lysozymes to Peptidase-rich Digestive Environments

As previously described, C-type lysozymes in *Musca domestica* larvae have a dual function, serving not only as immune-related proteins but also as digestive enzymes that hydrolyze bacteria in the diet, thereby contributing to nutrient acquisition. This dual functionality is supported by experimental evidence showing that two lysozymes expressed in the acidic region of the larval midgut display maximal enzymatic activity at low pH (∼4.8) and exhibit resistance to the host's cathepsin D. These features are considered adaptive to the digestive environment and resemble properties of lysozymes found in the stomachs of ruminants (Lemos et al. 1993; Cançado et al. 2008).

PGLS was used to assess whether comparable adaptations to midgut digestive conditions are widespread across insects, addressing our second initial question regarding structural or functional adaptation of lysozymes to digestive environments (see Introduction), in terms of the relationship between feeding habits and the number of predicted cleavage sites for major digestive peptidases. The peptidases considered included the serine peptidases trypsin and chymotrypsin, cysteine peptidases of the papain family, and the aspartic peptidase pepsin, which is functionally analogous to cathepsin D. However, contrary to our initial expectations, no significant associations were detected between dietary categories and variation in the number of predicted cleavage sites for any of the peptidases examined, providing no broad support for this hypothesis across Insecta. This null result does not necessarily contradict the species-specific resistance profile described for *M. domestica* midgut lysozymes, however, since pepsin, used here as a tractable proxy for comparative analysis, is not functionally identical to cathepsin D despite belonging to the same protease class. For example, the lysozyme MdL2 is resistant to *M. domestica* cathepsin D but exhibits approximately a 40% reduction in activity when incubated with bovine pepsin, indicating a finely tuned adaptation to the host-specific digestive environment that a generalized, cross-species cleavage-site prediction is unlikely to capture (Lemos et al. 1993). Such species-specific proteolytic interactions are complex to capture using generalized *in silico* predictions and cannot be explicitly accounted for in the present analyses.

### Expression Profiles of Lysozymes Along the Digestive System of Different Insect Species

Because the gut lumen constitutes a major interface of the insect immune system, the expression patterns discussed below are interpreted under the assumption that digestive and immune functions of lysozyme are not mutually exclusive: high expression in digestive tissue is consistent with, but does not by itself establish, recruitment for nutrient digestion, since the same activity can simultaneously limit bacterial proliferation in the gut lumen.

An additional limitation applies to tissues known to undergo endoreplication in some insects, such as salivary glands and portions of the midgut (Ren et al. 2020): elevated ploidy in these tissues could inflate apparent transcript abundance (TPM) independent of true regulatory upregulation, a confound that cannot be disentangled using bulk RNA-seq data alone. This should be kept in mind when interpreting the comparatively high expression levels reported below, particularly for tissues such as the *P. americana* salivary glands.

C-type lysozymes have previously been reported to be active in the anterior and medial regions of the midgut of *Musca domestica*, where they act synergistically with aspartic peptidases such as cathepsin D to enhance bacterial degradation (Espinoza-Fuentes and Terra 1987). In this species, lysozymes are clearly integrated into the digestive process, contributing to both immune defense and nutrient acquisition, due to their activity in an acidic environment, resulting from the presence of residues N46, S106 and T107, which interact with the catalytic residues and promote a reduction in pKa values (Cançado et al. 2010). Unlike the other model species analyzed here, this dual digestive/immune role in *M. domestica* is corroborated by independent biochemical evidence (acid pH optimum and resistance to host cathepsin D) rather than by expression data alone.

As discussed above, adaptations to bacteria-rich diets appear to be most pronounced within Diptera, with comparatively limited evidence for similar molecular specializations in other detritivorous groups, such as Blattodea. In cockroaches, C-type lysozyme copy numbers (five in *B. germanica* and eight in *P. americana*) are far lower than in *M. domestica* (43 copies), and transcriptomic data indicate that *P. americana* lysozyme expression is predominantly localized in the salivary glands. This expression pattern suggests a role primarily in controlling ingested pathogens at the point of food intake; an antiseptic, immune-type function that nonetheless acts on the same bacterial populations relevant to digestion, rather than a role in bulk bacterial digestion for nutrient acquisition per se. This interpretation is further supported by the co-expression of multiple antimicrobial peptides in the salivary glands of *Blattella germanica* (Zuber et al. 2022). Such a function may reflect an ancestral condition within Blattodea, as the production of antimicrobial compounds in salivary tissues has also been reported in termites (Costa-Leonardo et al. 2021).

Notably, one *P. americana* lysozyme sequence (XP_069673831.1) exhibited its highest expression levels in the foregut and midgut, suggesting that it could represent a candidate lysozyme with a digestive role (Table S14). However, predictions of cleavage-site susceptibility do not indicate resistance to trypsin, the primary digestive protease in *P. americana* (Tamaki et al. 2014), raising uncertainty about the stability and functionality of this protein in the gut lumen. An alternative explanation is that the lysozyme transcripts detected in the foregut are derived from salivary gland tissues that were translocated during dissection rather than being locally synthesized. Even in this scenario, salivary-derived lysozymes may still exert functional effects at this site, which corresponds to the region where the initial steps of digestion occur in cockroaches. Rather than contributing directly to bulk bacterial digestion for nutrient acquisition, these lysozymes may primarily function in regulating the microbiota; a function that is itself immune in nature but operates within, and is difficult to fully separate from, the digestive tract. Given that the core gut microbiota of cockroaches is dominated by Gram-negative bacteria, which are generally less susceptible to lysozyme-mediated lysis, lysozymes may instead help maintain microbial homeostasis by limiting the establishment of exogenous or opportunistic microbes (Tinker and Ottesen 2016).

A regulatory role for lysozymes in host-symbiont interactions may also occur in *R. prolixus*. In this species, C-type lysozymes were found to be highly expressed across multiple midgut regions, particularly in the anterior midgut (Table S12). These expression patterns are consistent with a role in regulating bacteria and other microorganisms associated with blood meals and opportunistic pathogens, including interactions involving *Rhodococcus rhodnii* and *Trypanosoma cruzi* (Ursic-Bedoya et al. 2008; Ribeiro et al. 2014; Díaz-Garrido et al. 2026). As with *P. americana*, this pattern illustrates that gut-localized lysozyme expression need not indicate a strictly digestive role; regulation of blood-meal-associated and symbiotic microorganisms is itself a gut immune function.

In *T. molitor*, most lysozyme transcripts were also detected in the anterior midgut, an intriguing pattern for a species not typically exposed to high levels of environmental bacterial challenge (Table S13). However, *T. molitor* larvae harbor a diverse, metabolically active gut microbiota capable of degrading lignocellulosic substrates and even synthetic polymers such as polystyrene. Although this microbial community varies among individuals and environmental conditions, a core microbiota persists, plausibly reflecting the presence of heritable symbiotic bacteria not readily displaced by dietary shifts (Ilijin et al. 2025). Under this scenario, constitutive expression of lysozymes in the anterior midgut may help preserve the core microbiota by preventing invasion or overgrowth by external bacterial taxa. This pattern further illustrates that high expression in a digestive-tract region should not be interpreted as evidence for a nutrient-digestive role by default, since the same expression is equally consistent with a local, non-digestive antibacterial function protecting the resident microbiota.

Finally, *S. frugiperda* was notable for showing a markedly regionalized expression pattern, with I-type lysozyme transcripts dominating in the anterior midgut (roughly three-fold higher than C-type) while C-type transcripts dominated in the middle and posterior midgut (Table S15); when summed across all three regions, total C-type and I-type expression were nearly equivalent, underscoring that spatial segregation, rather than an overall shift toward I-type expression, is the more accurate description of this species’ pattern. This regional pattern may reflect a greater reliance on antiviral or non-bacteriolytic I-type functions specifically in the anterior midgut, rather than a broader shift away from classical antibacterial activity. Indeed, *S. frugiperda* is exposed to a wide range of viral pathogens transmitted through both parasitoids and host plants, potentially favoring the expression of I-type lysozymes with antiviral activity in this region (Hussain et al. 2021).

### Concluding Remarks

Revisiting the three questions that motivated this study, our results provide only partial support for the hypothesis that lysozymes in detritivorous insects have adapted to a digestive role.

- Do detritivorous insects harbor a greater number of lysozyme gene copies? This hypothesis is only weakly and partially supported. PGLS analyses detected a positive association between detritivory and C-type lysozyme copy number restricted to the larval stage (coefficient = 0.6731; *P* = 0.047), which was not replicated in adults and did not extend to a broad, cross-Insecta pattern. The most pronounced copy-number expansions associated with detritivory were concentrated within Diptera (eg *M. domestica*, *H. illucens*), rather than reflecting a general trend across the class.
- Have insect lysozymes undergone structural or functional adaptations to suit digestive environments? This hypothesis was not supported at the scale of Insecta as a whole. PGLS analyses of predicted peptidase cleavage sites found no significant associations between feeding habits and cleavage-site composition for any of the digestive peptidases examined. The clearest evidence for structural adaptation to digestion remains restricted to *M. domestica*, supported by independent biochemical evidence (acidic pH optimum, resistance to host cathepsin D).
- Does lysozyme expression vary with feeding habits and concentrate in gut regions of intense digestive activity? This hypothesis received partial support. Lysozyme expression was consistently concentrated in gut tissues across the five model species examined, but only in *M. domestica* is this expression clearly tied to a digestive role. In *R. prolixus*, *T. molitor*, *P. americana*, and *S. frugiperda*, gut-localized expression more plausibly reflects an immune function related to regulation of ingested or symbiotic microbiota rather than direct participation in nutrient digestion.

Taken together, these findings indicate that the recruitment of lysozymes as digestive enzymes represents a derived, evolutionary-restricted feature that emerged primarily within Diptera, rather than a general adaptation across Insecta. In contrast, although a relatively high number of lysozyme gene copies is inferred for the last common ancestor of insects, multiple lineage-specific contraction events occurred throughout insect evolution, in many cases reflecting reduced microbial exposure rather than loss of digestive function.

Notably, I-type lysozymes, despite lacking the catalytic residues required for canonical muramidase activity in other invertebrates, have maintained a relatively stable copy number across Insecta. Their widespread conservation, even in lineages that have lost C-type lysozymes, suggests that I-type lysozymes fulfill alternative biological functions, possibly involving non-enzymatic antibacterial mechanisms or antiviral activities rather than classical bacteriolytic roles.

Our results reinforce the need for future studies to prioritize functional assays in order to clarify the roles of lysozymes in insect immunity and physiology. In particular, Hymenoptera represent a promising group for further investigation, as both C-type and I-type lysozymes in this lineage frequently lack residues required for enzymatic activity, raising the possibility of functional diversification. In addition, detailed functional and biochemical characterization of species exhibiting extensive lysozyme gene expansions, such as *Hermetia illucens* and *Nilaparvata lugens*, could provide valuable insights into the antimicrobial and antiviral potential of these enzymes, as well as their applicability in biological control strategies.

## Methodology

### Genomic Data Acquisition and Bioinformatic Curation

The genomes of Insecta and outgroup species were retrieved from the Genome database of the National Center for Biotechnology Information (NCBI) in May 2024 (Table S16). To ensure data quality, only reference genomes with RefSeq gene annotations were retained, except for the genome of *Gryllus longicercus*, which was obtained from the GenBank database, and of *Rhodnius prolixus*, which was sourced from VectorBase. Besides insects, the genomes of the crustaceans *Procambarus clarkii* (Crustacea: Cambaridae), *Scylla paramamosain* (Crustacea: Portunidae), and *Eriocheir sinensis* (Crustacea: Varunidae), as well as the collembolan *Folsomia candida* (Collembola: Isotomidae), were also retrieved to be included as outgroups in the phylogenetic analyses. Genome scaffolds, protein sequences, and gene annotations (GTF/GFF formats) were retrieved using the NCBI Datasets command-line tool (O’Leary et al. 2024). For each protein dataset, the longest isoform for each gene was extracted using AGAT v0.8.1 (Dainat et al. 2022).

Genome quality was assessed using BUSCO v5.6.1 (Manni et al. 2021) in protein mode, with the arthropoda_odb10 lineage for outgroups and the insecta_odb10 lineage for insects. Only genomes with a completeness score above 90% were retained, except for taxonomically unique representatives, where a threshold of 80% was applied. Because genome completeness below 90% increases the risk of fragmented or missing gene models, copy-number estimates for the taxonomically unique representatives retained at 80% to 90% completeness should be interpreted as conservative, and potentially underestimated, relative to species assembled at higher completeness. A total of 275 insect species, comprising 13 different orders, were compiled to form the final dataset after filtering. The BUSCO results and taxonomic information of all genomes selected for this work are available in Table S16.

### Identification and Clustering of Lysozyme Orthologs

To identify the most comprehensible set of lysozyme genes, including those not predicted in the proteomes, and also to curate chimeric sequences, the BITACORA software v.1.4 (Vizueta et al. 2020) was employed to perform a de novo prediction, using the HMMER profile for both C-type and I-type lysozymes. These profiles were constructed from previously defined orthologous groups containing validated representatives of each lysozyme class.

The BITACORA proximity algorithm was executed with a stringent e-value threshold of 0.00001 and a minimum predicted length of 75 bp, ensuring high-confidence matches while minimizing false positives.

All protein sequences included in the original dataset, filtered to retain only the longest isoform per gene, along with the new non-redundant protein sequences predicted by BITACORA, identified by the overlap against the NCBI sequences by the bedtools intersect command (Quinlan and Hall 2010), were clustered into orthologous groups (OGs) using OrthoFinder v. 2.5.4 (Emms and Kelly 2019). The OGs with sequences related to lysozymes were searched using BLASTp, with reviewed protein sequences from these enzymes from Nematoda, Chordata, Arthropoda, and Mollusca species available in the UniProtKB database as queries (Table S17).

The OGs with lysozyme sequences were subsequently classified as C or I type based on domain-architecture predictions using InterProScan v5.61-93.0 (Jones et al. 2014). C-type lysozymes were identified by the presence of at least one of the four characteristic domains (Interpro IDs: IPR001916, IPR019799, and IPR000974), while the presence of the IPR008597 domain identified I-type lysozymes.

To exclude spurious sequences from the datasets, the identified orthologous groups (OGs) containing lysozyme sequences were subjected to additional filtering criteria. First, sequences were retained only if they contained the conserved active site residues specific to each lysozyme type. Subsequently, sequences were filtered by size to retain only those within ±50% of the average length for the respective lysozyme type, as determined from UniProt reference sequences. For C-type lysozymes, the presence of the active site residues 53-E and 70-D was verified through a sequence alignment with the reference sequence of *Gallus gallus* (UniProt ID: P00698). For I-type lysozymes, since none of the sequences had the active site residues (83-E and 94-D) conserved toward the invertebrate type lysozymes, as found in the reference sequence from the Mollusca species *Crassostrea virginica* (UniProt ID: B3A003), the filter was performed considering only the size factor, as aforementioned.

### Phylogenetic Analysis

Each final OG was then aligned individually using MAFFT v7 (Katoh and Standley 2013) with the L-INS-i iterative refinement method. Then the removal of poorly aligned positions was carried out using trimAL v1.4 (Capella-Gutiérrez et al. 2009) with the automated1 heuristic method. Following, we reconstructed maximum likelihood (ML) phylogenetic trees for each OG using IQ-TREE 3 (Wong et al. 2026) with the ModelFinderPlus (MFP) method (Kalyaanamoorthy et al. 2017) to select the best amino acid substitution model and performed 1,000 Ultrafast bootstrap samples (Hoang et al. 2018).

For the species tree, a phylogenetic analysis based on single-copy orthologous genes was conducted for the dataset. To achieve this, the insect genomes were re-evaluated using BUSCO with the Arthropoda lineage dataset only. Protein sequences from single-copy genes present in at least 98% of the sampled species were identified and retrieved using the BUSCO phylogenomics pipeline (https://github.com/jamiemcg/BUSCO_phylogenomics), yielding a total of 191 BUSCOs. The amino acid sequences from the selected BUSCOs were aligned with MAFFT using the L-INS-i iterative refinement strategy and subsequently trimmed with trimAL, as previously described. The processed alignments were concatenated into a single supermatrix using the catfasta2phyml script (https://github.com/nylander/catfasta2phyml). The ML phylogenetic inference was performed using IQ-TREE 3 (Wong et al. 2026), incorporating the MFP algorithm to determine the best-fit amino acid substitution model (Q.insect + I + I + R10) (Kalyaanamoorthy et al. 2017). Branch support was assessed with 1,000 Ultrafast bootstrap replicates. Additionally, a constraint tree reflecting the currently known phylogenetic relationships, as reported in previously published studies (Table S18), was used to guide inference. The resulting phylogenetic tree was visualized and annotated using the iTOL v7 web server (Letunic and Bork 2024). To establish temporal constraints, a dated phylogenetic tree was generated using 11 calibration points, each corresponding to inter-ordinal splits obtained from the TimeTree database (Kumar et al. 2022). Divergence times were estimated using a penalized likelihood approach implemented in r8s (Sanderson 2003), incorporating the total number of sites from the concatenated alignment. These datasets were designed to mitigate potential biases arising from taxonomic overrepresentation or recent gene duplication events.

### Evolutionary Dynamics of Lysozymes in Insecta

To evaluate the molecular adaptations of insect lysozymes, we analyzed changes in gene copy number across species, sequence adaptations that confer resistance to common digestive peptidases (Papain, Trypsin, Chymotrypsin, and Pepsin), and the correlations between these two variables and feeding habits (Table S19).

To estimate the gene loss and duplication events that occurred during insect evolution across each branch, we performed a reconciliation analysis using GeneRax v. 2.1.3 (Morel et al. 2020). For this purpose, the species tree and the gene trees constructed for each OG, containing either filtered I-type or C-type lysozyme sequences, were used as input. The analysis was conducted under the D + L model (duplication plus loss), and the LG + R6 + G and LG + R7 + G amino acid substitution models were applied to C-type and I-type lysozymes, respectively, as the MFP method in IQ-TREE 3 predicted these models to be the best for these sequences.

To assess the correlation between the number of gene copies and feeding habits, we employed PGLS regression (nlme package; Pinheiro et al. 2013) to test whether dietary habits influenced variation in lysozyme gene copy number across species.

Firstly, we accounted for the non-independence among species resulting from their shared evolutionary history by assessing the phylogenetic signal in lysozyme gene copy number. We evaluated phylogenetic signal using two model-based metrics, Blomberg's K (Blomberg et al. 2003) and Pagel's λ (Pagel 1999), together with the autocorrelation-based statistic Moran's I (Gittleman and Kot 1990), investigating how the phylogenetic signal was distributed across evolutionary time by constructing phylogenetic correlograms.

We also addressed potential non-stationarity issues associated with the large evolutionary timespan encompassed by Insecta. To this end, we tested the fit of four evolutionary models across the regimes for each species, here based on taxonomic order, using the OUwie package (Beaulieu et al. 2012). The following models were evaluated: BM1, which tests the agreement of the entire tree with Brownian motion; BMS, Brownian motion accounting for rate differences across regimes; OU1, in which species evolve under an Ornstein-Uhlenbeck (OU) process with single adaptive peak (optimum); OUM, OU model in which each regime determines a specific adaptive peak (optimum). The best-fit model was selected using the corrected Akaike Information Criterion (AICc), which identified the BMS and OU1 as the best-fit models for the C-type and I-type datasets, respectively.

We accounted for these evolutionary parameters by rescaling branch lengths using regime-specific evolutionary rates (σ^2^) estimated with the OUwie package. For the C-type dataset, we fitted a BMS model (Brownian Motion with multiple σ^2^ parameters), and for each regime, we summarized the σ^2^ estimates across 10 stochastic maps by their median. Branch lengths were then rescaled proportionally to the median σ^2^ of the corresponding regime, producing a tree where branch lengths reflect evolutionary rates rather than only elapsed time. The I-type dataset was rescaled using the optimal σ^2^. These re-scaled trees were subsequently used as a backbone in PGLS analyses to test for associations between ecological traits and gene copy number. The gene copy numbers were log-transformed to meet model assumptions and analyzed in relation to food source data obtained from the GBIF database and other online resources (Table S19), under a corBrownian model, using the phytophagous diet as a reference for the intercept, as this trait is the most common throughout insect evolution.

To further characterize the function of the sequences obtained, the sequences from the selected datasets were subjected to additional analyses. First, signal peptides were identified and removed using SignalP 6.0 (Teufel et al. 2022). Subsequently, the sequences were analyzed to quantify the number of cleavage sites for the proteolytic enzymes Papain, Trypsin, Chymotrypsin, and Pepsin, using the Rapid Peptide Generator software (Maillet 2019). To assess whether the distribution of cleavage site counts differed significantly among insect feeding strategies, we employed PGLS as previously described, with the average number of cleavage sites per species for each proteolytic enzyme as the response variable.

### I-type Lysozymes Function Investigation

Since the insect I-type lysozyme sequences had lost their active-site residues, we investigated whether these molecules still retain potential roles in immune defense. To this end, we assessed their classification as AMPs and subsequently evaluated their potential interactions with a simulated common lysozyme substrate.

The potential of these sequences to act as AMPs was assessed using the AMP Scanner V2 tool (Veltri et al. 2018), employing the 021820_FULL_MODEL pre-trained model. Among the I-type lysozyme sequences identified in our model species, those classified as probable AMPs and showing the highest expression levels were selected to simulate their interaction with N-acetylglucosamine trimer (NAG3). The ligand structure was extracted from the three-dimensional I-type lysozyme structure of the mollusk *Meretrix lusoria* (PDB ID: 3AB6). The three-dimensional structures of the selected insect I-type lysozyme sequences were predicted using the AlphaFold3 server (Abramson et al. 2024) and subsequently prepared for docking with the Dock Prep tool implemented in ChimeraX v1.10 (Meng et al. 2023). Protein-ligand docking was then performed using the HADDOCK web server (Honorato et al. 2024). Residues involved in hydrogen-bond interactions in the 3AB6 model were identified using the PLIP web tool (Schake et al. 2025) and defined as active residues during docking to guide the simulation of NAG3 binding.

### Gene Expression Analysis

Gene expression was assessed in five insect species using Illumina Paired-End RNA-seq data, previously generated as described by Dias et al. (2019). The datasets included RNA-seq libraries from various digestive and carcass tissues. In *Periplaneta americana* (Blattodea) adults, samples were obtained from the gastric cecum (GC), salivary glands (SG), foregut (FG), anterior and posterior midgut (AM and PM), hindgut (HG), Malpighian tubules (MT), and carcass (CAR). For *Rhodnius prolixus* (Hemiptera) adults and *Tenebrio molitor* (Coleoptera) larvae, the tissues analyzed comprised the anterior, middle, and posterior midgut (AM, MM, and PM) and carcass. In *Spodoptera frugiperda* (Lepidoptera) larvae, portions of the AM, MM, and PM were included, while for *Musca domestica* (Diptera) larvae, expression was analyzed in subdivided regions of the gut: AM1 and AM2 (first and second portions of the anterior midgut), MM1 and MM2 (middle midgut), PM1–PM3 (posterior midgut), and carcass. The RNA-Seq data were processed through the nf-core/rnaseq pipeline (Ewels et al. 2020), with read alignment performed with STAR (Dobin et al. 2013) against the reference genome of each species, followed by transcript quantification using RSEM (Li and Dewey 2011). Gene expression levels were quantified as TPM for each sample.

## Supplementary Material

evag265_Supplementary_Data

## Data Availability

This study did not generate new sequencing data. All genome assemblies analyzed are publicly available; NCBI and VectorBase accession numbers for the 279 genomes examined (275 insect species and four outgroup taxa) are provided in Table S16, and sequence identifiers for all lysozyme genes analyzed are listed in Tables S6 and S7. Per-species and per-node copy numbers and inferred duplication and loss events are provided in Tables S1 to S4. Lysozyme protein sequences, including those newly annotated in this study with BITACORA, multiple sequence alignments of C-type and I-type lysozymes, copy-number data in tab-delimited format, the time-calibrated species tree, and the input data and R scripts used for the phylogenetic signal (Moran's I) and PGLS analyses are available at GitHub (https://github.com/hector-romao/romao-lysozymes). RNA-seq expression data used in this study were originally generated by Dias et al. (2019) and are described in that publication.
