# Supplementary material for: Duplication, Loss, and Feeding Ecology of Lysozyme Gene Copy Number in Insects
Source: Genome Biol Evol. 2026 Oct 22;18(11):evag265. doi: 10.1093/gbe/evag265 (PMC13736114; doi:10.1093/gbe/evag265)
Supplement: Supplementary file 1 [file evag265_supplementary_data.docx]

**Supplementary Material**

**Figure S1** - Phylogenetic correlograms of Moran's I autocorrelation coefficients as a function of cophenetic distance for C-type (top) and I-type (bottom) lysozyme gene copy number. Positive values indicate phylogenetic autocorrelation (similar copy numbers among closely related taxa) at a given distance interval, negative values indicate dissimilar copy numbers among distantly related taxa, and values near zero indicate no detectable phylogenetic signal. Filled circles denote distance classes with statistically significant autocorrelation (P < 0.05 after Bonferroni correction for multiple comparisons); open circles denote non-significant classes (P ≥ 0.05). The dashed line indicates I = 0.

**Alt text:** Two correlograms of Moran's I against cophenetic distance for C-type (top) and I-type (bottom) lysozyme copy number, showing positive phylogenetic autocorrelation at short distances that decreases towards zero at larger distances.

**Table S1 -** Species-level gene duplication, loss and speciation events inferred by GeneRax for the C-type lysozyme gene family across Arthropoda

| **Species** | **Copy number (specia-tions)** | **Duplica-tions** | **Losses** | **Lineage** |
| --- | --- | --- | --- | --- |
| *Procambarus clarkii* | 8 | 6 | 0 | Crustacea |
| *Eriocheir sinensis* | 0 | 0 | 1 | Crustacea |
| *Scylla paramamosain* | 2 | 1 | 0 | Crustacea |
| *Folsomia candida* | 3 | 1 | 4 | Collembola |
| *Ischnura elegans* | 2 | 0 | 0 | Palaeoptera; Odonata; Zygoptera; Coenagrionoidea; Coenagrionidae |
| *Hetaerina americana* | 2 | 0 | 0 | Palaeoptera; Odonata; Zygoptera; Calopterygoidea; Calopterygidae |
| *Cloeon dipterum* | 1 | 0 | 0 | Palaeoptera; Ephemeroptera; Pisciforma; Baetidae |
| *Neocloeon triangulifer* | 1 | 0 | 0 | Palaeoptera; Ephemeroptera; Pisciforma; Baetidae |
| *Gryllus longicercus* | 4 | 0 | 2 | Polyneoptera; Orthoptera; Ensifera; Gryllidea; Grylloidea ; Gryllidae ; Gryllinae |
| *Anabrus simplex* | 3 | 0 | 3 | Polyneoptera ; Orthoptera ; Ensifera ; Tettigoniidea ; Tettigonioidea ; Tettigoniidae ; Tettigoniinae |
| *Bacillus rossius redtenbacheri* | 3 | 0 | 5 | Polyneoptera; Phasmatodea; Verophasmatodea; Areolatae; Bacilloidea; Bacillidae; Bacillinae; Bacillini |
| *Blattella germanica* | 5 | 1 | 5 | Polyneoptera; Dictyoptera; Blattodea; Blaberoidea; Blattellidae |
| *Periplaneta americana* | 8 | 4 | 3 | Polyneoptera ; Dictyoptera ; Blattodea ; Blattoidea ; Blattidae ; Blattinae |
| *Zootermopsis nevadensis* | 3 | 0 | 1 | Polyneoptera; Dictyoptera; Blattodea; Blattoidea; Termitoidae; Termopsidae; Termopsinae; Termopsini |
| *Coptotermes formosanus* | 3 | 1 | 4 | Polyneoptera; Dictyoptera; Blattodea; Blattoidea; Termitoidae; Rhinotermitidae; Coptotermitinae |
| *Cryptotermes secundus* | 8 | 2 | 0 | Polyneoptera; Dictyoptera; Blattodea; Blattoidea; Termitoidae; Kalotermitidae; Cryptotermitinae |
| *Frankliniella occidentalis* | 6 | 1 | 1 | Paraneoptera; Thysanoptera; Terebrantia; Thripoidea; Thripidae; Thripinae |
| *Thrips palmi* | 6 | 3 | 3 | Paraneoptera; Thysanoptera; Terebrantia; Thripoidea; Thripidae; Thripinae |
| *Homalodisca vitripennis* | 2 | 1 | 1 | Paraneoptera; Hemiptera; Auchenorrhyncha; Cicadomorpha; Membracoidea; Cicadellidae; Cicadellinae; Proconiini |
| *Laodelphax striatellus* | 1 | 0 | 0 | Paraneoptera; Hemiptera; Auchenorrhyncha; Fulgoromorpha; Fulgoroidea; Delphacidae; Criomorphinae |
| *Nilaparvata lugens* | 1 | 0 | 0 | Paraneoptera; Hemiptera; Auchenorrhyncha; Fulgoromorpha; Fulgoroidea; Delphacidae; Delphacinae |
| *Halyomorpha halys* | 0 | 0 | 6 | Paraneoptera; Hemiptera; Prosorrhyncha; Heteroptera; Euheteroptera; Neoheteroptera; Panheteroptera; Pentatomomorpha; Pentatomoidea; Pentatomidae; Pentatominae |
| *Rhodnius prolixus* | 3 | 0 | 3 | Paraneoptera; Hemiptera; Prosorrhyncha; Heteroptera; Euheteroptera; Neoheteroptera; Panheteroptera; Cimicomorpha; Reduvioidea; Reduviidae; Triatominae |
| *Apolygus lucorum* | 2 | 0 | 2 | Paraneoptera; Hemiptera; Prosorrhyncha; Heteroptera; Euheteroptera; Neoheteroptera; Panheteroptera; Cimicomorpha; Cimicoidea; Miridae; Mirinae; Mirini |
| *Cimex lectularius* | 2 | 0 | 2 | Paraneoptera; Hemiptera; Prosorrhyncha; Heteroptera; Euheteroptera; Neoheteroptera; Panheteroptera; Cimicomorpha; Cimicoidea; Cimicidae |
| *Bemisia tabaci* | 2 | 1 | 0 | Paraneoptera; Hemiptera; Sternorrhyncha; Aleyrodoidea; Aleyrodidae; Aleyrodinae |
| *Planococcus citri* | 0 | 0 | 0 | Paraneoptera; Hemiptera; Sternorrhyncha; Coccoidea; Pseudococcidae |
| *Adelges cooleyi* | 0 | 0 | 0 | Paraneoptera; Hemiptera; Sternorrhyncha; Aphidomorpha; Phylloxeroidea; Adelgidae; Adelges; Gilletteella |
| *Daktulosphaira vitifoliae* | 0 | 0 | 0 | Paraneoptera; Hemiptera; Sternorrhyncha; Aphidomorpha; Phylloxeroidea; Phylloxeridae |
| *Sipha flava* | 0 | 0 | 0 | Paraneoptera; Hemiptera; Sternorrhyncha; Aphidomorpha; Aphidoidea; Aphididae; Chaitophorinae |
| *Acyrthosiphon pisum* | 0 | 0 | 0 | Paraneoptera; Hemiptera; Sternorrhyncha; Aphidomorpha; Aphidoidea; Aphididae; Aphidinae; Macrosiphini |
| *Myzus persicae* | 0 | 0 | 0 | Paraneoptera; Hemiptera; Sternorrhyncha; Aphidomorpha; Aphidoidea; Aphididae; Aphidinae; Macrosiphini |
| *Melanaphis sacchari* | 0 | 0 | 0 | Paraneoptera; Hemiptera; Sternorrhyncha; Aphidomorpha; Aphidoidea; Aphididae; Aphidinae; Aphidini |
| *Aphis craccivora* | 0 | 0 | 0 | Paraneoptera; Hemiptera; Sternorrhyncha; Aphidomorpha; Aphidoidea; Aphididae; Aphidinae; Aphidini; Aphis |
| *Aphis gossypii* | 0 | 0 | 0 | Paraneoptera; Hemiptera; Sternorrhyncha; Aphidomorpha; Aphidoidea; Aphididae; Aphidinae; Aphidini |
| *Rhopalosiphum maidis* | 0 | 0 | 0 | Paraneoptera; Hemiptera; Sternorrhyncha; Aphidomorpha; Aphidoidea; Aphididae; Aphidinae; Aphidini |
| *Rhopalosiphum padi* | 0 | 0 | 0 | Paraneoptera; Hemiptera; Sternorrhyncha; Aphidomorpha; Aphidoidea; Aphididae; Aphidinae; Aphidini |
| *Pediculus humanus corporis* | 1 | 0 | 13 | Paraneoptera; Psocodea; Phthiraptera; Anoplura; Pediculidae |
| *Athalia rosae* | 3 | 1 | 1 | Endopterygota; Hymenoptera; Tenthredinoidea; Athaliidae |
| *Diprion similis* | 2 | 0 | 1 | Endopterygota; Hymenoptera; Tenthredinoidea; Diprionidae; Diprioninae |
| *Neodiprion fabricii* | 2 | 0 | 1 | Endopterygota; Hymenoptera; Tenthredinoidea; Diprionidae; Diprioninae |
| *Neodiprion virginianus* | 2 | 0 | 1 | Endopterygota; Hymenoptera; Tenthredinoidea; Diprionidae; Diprioninae |
| *Neodiprion lecontei* | 2 | 0 | 0 | Endopterygota; Hymenoptera; Tenthredinoidea; Diprionidae; Diprioninae |
| *Neodiprion pinetum* | 2 | 0 | 0 | Endopterygota; Hymenoptera; Tenthredinoidea; Diprionidae; Diprioninae |
| *Cephus cinctus* | 1 | 0 | 1 | Endopterygota; Hymenoptera; Cephoidea; Cephidae |
| *Orussus abietinus* | 0 | 0 | 1 | Endopterygota; Hymenoptera; Orussoidea; Orussidae |
| *Belonocnema kinseyi* | 0 | 0 | 0 | Endopterygota; Hymenoptera; Apocrita; Proctotrupomorpha; Cynipoidea; Cynipidae; Cynipinae; Cynipini |
| *Leptopilina boulardi* | 0 | 0 | 0 | Endopterygota; Hymenoptera; Apocrita; Proctotrupomorpha; Cynipoidea; Figitidae; Eucoilinae |
| *Leptopilina heterotoma* | 0 | 0 | 0 | Endopterygota; Hymenoptera; Apocrita; Proctotrupomorpha; Cynipoidea; Figitidae; Eucoilinae |
| *Trichogramma pretiosum* | 0 | 0 | 0 | Endopterygota; Hymenoptera; Apocrita; Proctotrupomorpha; Chalcidoidea; Trichogrammatidae |
| *Copidosoma floridanum* | 0 | 0 | 0 | Endopterygota; Hymenoptera; Apocrita; Proctotrupomorpha; Chalcidoidea; Encyrtidae; Encyrtinae |
| *Phymastichus coffea* | 0 | 0 | 0 | Endopterygota; Hymenoptera; Apocrita; Proctotrupomorpha; Chalcidoidea; Eulophidae; Tetrastichinae |
| *Ceratosolen solmsi marchali* | 0 | 0 | 0 | Endopterygota; Hymenoptera; Apocrita; Proctotrupomorpha; Chalcidoidea; Agaonidae; Agaoninae |
| *Nasonia vitripennis* | 0 | 0 | 0 | Endopterygota; Hymenoptera; Apocrita; Proctotrupomorpha; Chalcidoidea; Pteromalidae; Pteromalinae |
| *Venturia canescens* | 0 | 0 | 1 | Endopterygota; Hymenoptera; Apocrita; Ichneumonoidea; Ichneumonidae; Campopleginae; Dusona group |
| *Aphidius gifuensis* | 0 | 0 | 0 | Endopterygota; Hymenoptera; Apocrita; Ichneumonoidea; Braconidae; Aphidiinae |
| *Fopius arisanus* | 0 | 0 | 0 | Endopterygota; Hymenoptera; Apocrita; Ichneumonoidea; Braconidae; Opiinae |
| *Diachasma alloeum* | 0 | 0 | 0 | Endopterygota; Hymenoptera; Apocrita; Ichneumonoidea; Braconidae; Opiinae |
| *Diachasmimorpha longicaudata* | 0 | 0 | 0 | Endopterygota; Hymenoptera; Apocrita; Ichneumonoidea; Braconidae; Opiinae; Diachasmimorpha |
| *Chelonus insularis* | 1 | 0 | 0 | Endopterygota; Hymenoptera; Apocrita; Ichneumonoidea; Braconidae; Cheloninae |
| *Cotesia glomerata* | 0 | 0 | 0 | Endopterygota; Hymenoptera; Apocrita; Ichneumonoidea; Braconidae; Microgastrinae |
| *Microplitis demolitor* | 0 | 0 | 0 | Endopterygota; Hymenoptera; Apocrita; Ichneumonoidea; Braconidae; Microgastrinae |
| *Microplitis mediator* | 0 | 0 | 0 | Endopterygota; Hymenoptera; Apocrita; Ichneumonoidea; Braconidae; Microgastrinae |
| *Polistes dominula* | 0 | 0 | 0 | Endopterygota; Hymenoptera; Apocrita; Aculeata; Vespoidea; Vespidae; Polistinae; Polistini |
| *Polistes canadensis* | 0 | 0 | 0 | Endopterygota; Hymenoptera; Apocrita; Aculeata; Vespoidea; Vespidae; Polistinae; Polistini |
| *Polistes fuscatus* | 0 | 0 | 0 | Endopterygota; Hymenoptera; Apocrita; Aculeata; Vespoidea; Vespidae; Polistinae; Polistini |
| *Vespula pensylvanica* | 0 | 0 | 0 | Endopterygota; Hymenoptera; Apocrita; Aculeata; Vespoidea; Vespidae; Vespinae |
| *Vespula vulgaris* | 0 | 0 | 0 | Endopterygota; Hymenoptera; Apocrita; Aculeata; Vespoidea; Vespidae; Vespinae |
| *Vespa velutina* | 0 | 0 | 0 | Endopterygota; Hymenoptera; Apocrita; Aculeata; Vespoidea; Vespidae; Vespinae |
| *Vespa crabro* | 0 | 0 | 0 | Endopterygota; Hymenoptera; Apocrita; Aculeata; Vespoidea; Vespidae; Vespinae |
| *Vespa mandarinia* | 0 | 0 | 0 | Endopterygota; Hymenoptera; Apocrita; Aculeata; Vespoidea; Vespidae; Vespinae |
| *Ampulex compressa* | 0 | 0 | 0 | Endopterygota; Hymenoptera; Apocrita; Aculeata; Apoidea; Ampulicidae; Ampulicini |
| *Colletes gigas* | 0 | 0 | 0 | Endopterygota; Hymenoptera; Apocrita; Aculeata; Apoidea; Anthophila; Colletidae; Colletinae |
| *Hylaeus anthracinus* | 0 | 0 | 0 | Endopterygota; Hymenoptera; Apocrita; Aculeata; Apoidea; Anthophila; Colletidae; Hylaeinae; Hylaeus; Nesoprosopis |
| *Hylaeus volcanicus* | 0 | 0 | 0 | Endopterygota; Hymenoptera; Apocrita; Aculeata; Apoidea; Anthophila; Colletidae; Hylaeinae; Hylaeus; Nesoprosopis |
| *Dufourea novaeangliae* | 0 | 0 | 0 | Endopterygota; Hymenoptera; Apocrita; Aculeata; Apoidea; Anthophila; Halictidae; Rophitinae |
| *Megalopta genalis* | 0 | 0 | 0 | Endopterygota; Hymenoptera; Apocrita; Aculeata; Apoidea; Anthophila; Halictidae; Halictinae; Augochlorini |
| *Nomia melanderi* | 0 | 0 | 0 | Endopterygota; Hymenoptera; Apocrita; Aculeata; Apoidea; Anthophila; Halictidae; Nomiinae; Nomia; Acunomia |
| *Megachile rotundata* | 0 | 0 | 0 | Endopterygota; Hymenoptera; Apocrita; Aculeata; Apoidea; Anthophila; Megachilidae; Megachilinae; Megachilini |
| *Osmia bicornis bicornis* | 0 | 0 | 0 | Endopterygota; Hymenoptera; Apocrita; Aculeata; Apoidea; Anthophila; Megachilidae; Megachilinae; Osmiini; Osmia |
| *Osmia lignaria* | 0 | 0 | 0 | Endopterygota; Hymenoptera; Apocrita; Aculeata; Apoidea; Anthophila; Megachilidae; Megachilinae; Osmiini |
| *Ceratina calcarata* | 0 | 0 | 0 | Endopterygota; Hymenoptera; Apocrita; Aculeata; Apoidea; Anthophila; Apidae; Xylocopinae; Ceratinini; Ceratina |
| *Eufriesea mexicana* | 0 | 0 | 0 | Endopterygota; Hymenoptera; Apocrita; Aculeata; Apoidea; Anthophila; Apidae; Apinae; Euglossini |
| *Bombus affinis* | 0 | 0 | 0 | Endopterygota; Hymenoptera; Apocrita; Aculeata; Apoidea; Anthophila; Apidae; Apinae; Bombini |
| *Bombus terrestris* | 0 | 0 | 0 | Endopterygota; Hymenoptera; Apocrita; Aculeata; Apoidea; Anthophila; Apidae; Apinae; Bombini |
| *Frieseomelitta varia* | 0 | 0 | 0 | Endopterygota; Hymenoptera; Apocrita; Aculeata; Apoidea; Anthophila; Apidae; Apinae; Meliponini |
| *Melipona quadrifasciata* | 0 | 0 | 0 | Endopterygota; Hymenoptera; Apocrita; Aculeata; Apoidea; Anthophila; Apidae; Apinae; Meliponini |
| *Apis florea* | 0 | 0 | 0 | Endopterygota; Hymenoptera; Apocrita; Aculeata; Apoidea; Anthophila; Apidae; Apinae; Apini |
| *Apis cerana* | 0 | 0 | 0 | Endopterygota; Hymenoptera; Apocrita; Aculeata; Apoidea; Anthophila; Apidae; Apinae; Apini |
| *Apis mellifera* | 0 | 0 | 0 | Endopterygota; Hymenoptera; Apocrita; Aculeata; Apoidea; Anthophila; Apidae; Apinae; Apini |
| *Apis dorsata* | 0 | 0 | 0 | Endopterygota; Hymenoptera; Apocrita; Aculeata; Apoidea; Anthophila; Apidae; Apinae; Apini |
| *Apis laboriosa* | 0 | 0 | 0 | Endopterygota; Hymenoptera; Apocrita; Aculeata; Apoidea; Anthophila; Apidae; Apinae; Apini |
| *Dinoponera quadriceps* | 0 | 0 | 0 | Endopterygota; Hymenoptera; Apocrita; Aculeata; Formicoidea; Formicidae; Ponerinae; Ponerini |
| *Harpegnathos saltator* | 0 | 0 | 0 | Endopterygota; Hymenoptera; Apocrita; Aculeata; Formicoidea; Formicidae; Ponerinae; Ponerini |
| *Odontomachus brunneus* | 0 | 0 | 0 | Endopterygota; Hymenoptera; Apocrita; Aculeata; Formicoidea; Formicidae; Ponerinae; Ponerini |
| *Eciton burchellii* | 0 | 0 | 0 | Endopterygota; Hymenoptera; Apocrita; Aculeata; Formicoidea; Formicidae; Dorylinae |
| *Ooceraea biroi* | 0 | 0 | 0 | Endopterygota; Hymenoptera; Apocrita; Aculeata; Formicoidea; Formicidae; Dorylinae |
| *Linepithema humile* | 0 | 0 | 0 | Endopterygota; Hymenoptera; Apocrita; Aculeata; Formicoidea; Formicidae; Dolichoderinae |
| *Pseudomyrmex gracilis* | 0 | 0 | 0 | Endopterygota; Hymenoptera; Apocrita; Aculeata; Formicoidea; Formicidae; Pseudomyrmecinae |
| *Nylanderia fulva* | 0 | 0 | 0 | Endopterygota; Hymenoptera; Apocrita; Aculeata; Formicoidea; Formicidae; Formicinae; Lasiini |
| *Camponotus floridanus* | 0 | 0 | 0 | Endopterygota; Hymenoptera; Apocrita; Aculeata; Formicoidea; Formicidae; Formicinae; Camponotini |
| *Cataglyphis hispanica* | 0 | 0 | 0 | Endopterygota; Hymenoptera; Apocrita; Aculeata; Formicoidea; Formicidae; Formicinae; Formicini |
| *Formica exsecta* | 0 | 0 | 0 | Endopterygota; Hymenoptera; Apocrita; Aculeata; Formicoidea; Formicidae; Formicinae; Formicini |
| *Pogonomyrmex barbatus* | 0 | 0 | 0 | Endopterygota; Hymenoptera; Apocrita; Aculeata; Formicoidea; Formicidae; Myrmicinae; Pogonomyrmecini |
| *Vollenhovia emeryi* | 0 | 0 | 0 | Endopterygota; Hymenoptera; Apocrita; Aculeata; Formicoidea; Formicidae; Myrmicinae; Stenammini |
| *Temnothorax curvispinosus* | 0 | 0 | 0 | Endopterygota; Hymenoptera; Apocrita; Aculeata; Formicoidea; Formicidae; Myrmicinae; Crematogastrini |
| *Temnothorax longispinosus* | 0 | 0 | 0 | Endopterygota; Hymenoptera; Apocrita; Aculeata; Formicoidea; Formicidae; Myrmicinae; Crematogastrini |
| *Monomorium pharaonis* | 0 | 0 | 0 | Endopterygota; Hymenoptera; Apocrita; Aculeata; Formicoidea; Formicidae; Myrmicinae; Solenopsidini |
| *Solenopsis invicta* | 0 | 0 | 0 | Endopterygota; Hymenoptera; Apocrita; Aculeata; Formicoidea; Formicidae; Myrmicinae; Solenopsidini |
| *Wasmannia auropunctata* | 0 | 0 | 0 | Endopterygota; Hymenoptera; Apocrita; Aculeata; Formicoidea; Formicidae; Myrmicinae; Blepharidattini |
| *Cyphomyrmex costatus* | 0 | 0 | 0 | Endopterygota; Hymenoptera; Apocrita; Aculeata; Formicoidea; Formicidae; Myrmicinae; Attini |
| *Trachymyrmex septentrionalis* | 0 | 0 | 0 | Endopterygota; Hymenoptera; Apocrita; Aculeata; Formicoidea; Formicidae; Myrmicinae; Attini |
| *Acromyrmex echinatior* | 0 | 0 | 0 | Endopterygota; Hymenoptera; Apocrita; Aculeata; Formicoidea; Formicidae; Myrmicinae; Attini |
| *Atta cephalotes* | 0 | 0 | 0 | Endopterygota; Hymenoptera; Apocrita; Aculeata; Formicoidea; Formicidae; Myrmicinae; Attini |
| *Atta colombica* | 0 | 0 | 0 | Endopterygota; Hymenoptera; Apocrita; Aculeata; Formicoidea; Formicidae; Myrmicinae; Attini |
| *Chrysoperla carnea* | 9 | 4 | 5 | Endopterygota; Neuropterida; Neuroptera; Hemerobiiformia; Chrysopidae; Chrysopinae |
| *Agrilus planipennis* | 3 | 1 | 1 | Endopterygota; Coleoptera; Polyphaga; Elateriformia; Buprestoidea; Buprestidae; Agrilinae |
| *Ignelater luminosus* | 0 | 0 | 2 | Endopterygota; Coleoptera; Polyphaga; Elateriformia; Elateroidea; Elateridae; Agrypninae; Pyrophorini |
| *Abscondita terminalis* | 2 | 0 | 1 | Endopterygota; Coleoptera; Polyphaga; Elateriformia; Elateroidea; Lampyridae; Luciolinae |
| *Lamprigera yunnana* | 1 | 0 | 1 | Endopterygota; Coleoptera; Polyphaga; Elateriformia; Elateroidea; Lampyridae; Lampyrinae |
| *Photinus pyralis* | 1 | 0 | 1 | Endopterygota; Coleoptera; Polyphaga; Elateriformia; Elateroidea; Lampyridae; Lampyrinae |
| *Onthophagus taurus* | 6 | 3 | 1 | Endopterygota; Coleoptera; Polyphaga; Scarabaeiformia; Scarabaeoidea; Scarabaeidae; Scarabaeinae; Onthophagini |
| *Dalotia coriaria* | 4 | 2 | 1 | Endopterygota; Coleoptera; Polyphaga; Staphyliniformia; Staphylinoidea; Staphylinidae; Tachyporinae group; Aleocharinae; Athetini; |
| *Nicrophorus vespilloides* | 6 | 3 | 0 | Endopterygota; Coleoptera; Polyphaga; Staphyliniformia; Staphylinoidea; Silphidae; Nicrophorinae |
| *Tenebrio molitor* | 2 | 0 | 1 | Endopterygota; Coleoptera; Polyphaga; Cucujiformia; Tenebrionoidea; Tenebrionidae; Tenebrioninae |
| *Zophobas morio* | 4 | 1 | 0 | Endopterygota; Coleoptera; Polyphaga; Cucujiformia; Tenebrionoidea; Tenebrionidae; Tenebrioninae |
| *Tribolium castaneum* | 4 | 0 | 0 | Endopterygota; Coleoptera; Polyphaga; Cucujiformia; Tenebrionoidea; Tenebrionidae |
| *Tribolium madens* | 1 | 0 | 3 | Endopterygota; Coleoptera; Polyphaga; Cucujiformia; Tenebrionoidea; Tenebrionidae |
| *Coccinella septempunctata* | 5 | 1 | 0 | Endopterygota; Coleoptera; Polyphaga; Cucujiformia; Coccinelloidea; Coccinellidae; Coccinellinae; Coccinellini |
| *Harmonia axyridis* | 4 | 0 | 0 | Endopterygota; Coleoptera; Polyphaga; Cucujiformia; Coccinelloidea; Coccinellidae; Coccinellinae; Coccinellini |
| *Aethina tumida* | 6 | 3 | 1 | Endopterygota; Coleoptera; Polyphaga; Cucujiformia; Cucujoidea; Nitidulidae; Nitidulinae |
| *Brassicogethes aeneus* | 3 | 0 | 1 | Endopterygota; Coleoptera; Polyphaga; Cucujiformia; Cucujoidea; Nitidulidae; Meligethinae |
| *Cylas formicarius* | 4 | 0 | 2 | Endopterygota; Coleoptera; Polyphaga; Cucujiformia; Curculionoidea; Brentidae; Cyladinae |
| *Anthonomus grandis grandis* | 4 | 0 | 1 | Endopterygota; Coleoptera; Polyphaga; Cucujiformia; Curculionoidea; Curculionidae; Curculioninae; Anthonomini |
| *Dendroctonus ponderosae* | 2 | 0 | 3 | Endopterygota; Coleoptera; Polyphaga; Cucujiformia; Curculionoidea; Curculionidae; Scolytinae |
| *Rhynchophorus ferrugineus* | 5 | 2 | 0 | Endopterygota; Coleoptera; Polyphaga; Cucujiformia; Curculionoidea; Curculionidae; Dryophthorinae |
| *Sitophilus oryzae* | 1 | 0 | 2 | Endopterygota; Coleoptera; Polyphaga; Cucujiformia; Curculionoidea; Curculionidae; Dryophthorinae |
| *Anoplophora glabripennis* | 6 | 3 | 3 | Endopterygota; Coleoptera; Polyphaga; Cucujiformia; Chrysomeloidea; Cerambycidae; Lamiinae; Lamiini |
| *Gonioctena quinquepunctata* | 2 | 0 | 0 | Endopterygota; Coleoptera; Polyphaga; Cucujiformia; Chrysomeloidea; Chrysomelidae; Chrysomelinae; Chrysomelini |
| *Leptinotarsa decemlineata* | 2 | 0 | 0 | Endopterygota; Coleoptera; Polyphaga; Cucujiformia; Chrysomeloidea; Chrysomelidae; Chrysomelinae; Doryphorini |
| *Diabrotica virgifera virgifera* | 6 | 1 | 1 | Endopterygota; Coleoptera; Polyphaga; Cucujiformia; Chrysomeloidea; Chrysomelidae; Galerucinae; Luperini; Diabroticina; Diabroticites |
| *Diorhabda carinulata* | 0 | 0 | 4 | Endopterygota; Coleoptera; Polyphaga; Cucujiformia; Chrysomeloidea; Chrysomelidae; Galerucinae; Galerucini; Atysites |
| *Diorhabda sublineata* | 4 | 0 | 0 | Endopterygota; Coleoptera; Polyphaga; Cucujiformia; Chrysomeloidea; Chrysomelidae; Galerucinae; Galerucini; Atysites |
| *Plutella xylostella* | 2 | 0 | 2 | Endopterygota; Amphiesmenoptera; Lepidoptera; Glossata; Neolepidoptera; Heteroneura; Ditrysia; Yponomeutoidea; Plutellidae |
| *Leguminivora glycinivorella* | 4 | 1 | 1 | Endopterygota; Amphiesmenoptera; Lepidoptera; Glossata; Neolepidoptera; Heteroneura; Ditrysia; Apoditrysia; Tortricoidea; Tortricidae; Olethreutinae; Grapholitini |
| *Cydia pomonella* | 4 | 0 | 0 | Endopterygota; Amphiesmenoptera; Lepidoptera; Glossata; Neolepidoptera; Heteroneura; Ditrysia; Apoditrysia; Tortricoidea; Tortricidae; Olethreutinae; Grapholitini |
| *Cydia splendana* | 4 | 1 | 1 | Endopterygota; Amphiesmenoptera; Lepidoptera; Glossata; Neolepidoptera; Heteroneura; Ditrysia; Apoditrysia; Tortricoidea; Tortricidae; Olethreutinae; Grapholitini |
| *Hyposmocoma kahamanoa* | 3 | 0 | 3 | Endopterygota; Amphiesmenoptera; Lepidoptera; Glossata; Neolepidoptera; Heteroneura; Ditrysia; Gelechioidea; Cosmopterigidae; Cosmopteriginae |
| *Pectinophora gossypiella* | 5 | 0 | 1 | Endopterygota; Amphiesmenoptera; Lepidoptera; Glossata; Neolepidoptera; Heteroneura; Ditrysia; Gelechioidea; Gelechiidae; Apatetrinae |
| *Chilo suppressalis* | 7 | 3 | 1 | Endopterygota; Amphiesmenoptera; Lepidoptera; Glossata; Neolepidoptera; Heteroneura; Ditrysia; Obtectomera; Pyraloidea; Crambidae; Crambinae |
| *Ostrinia furnacalis* | 6 | 1 | 0 | Endopterygota; Amphiesmenoptera; Lepidoptera; Glossata; Neolepidoptera; Heteroneura; Ditrysia; Obtectomera; Pyraloidea; Crambidae; Pyraustinae |
| *Ostrinia nubilalis* | 5 | 0 | 0 | Endopterygota; Amphiesmenoptera; Lepidoptera; Glossata; Neolepidoptera; Heteroneura; Ditrysia; Obtectomera; Pyraloidea; Crambidae; Pyraustinae |
| *Achroia grisella* | 3 | 0 | 1 | Endopterygota; Amphiesmenoptera; Lepidoptera; Glossata; Neolepidoptera; Heteroneura; Ditrysia; Obtectomera; Pyraloidea; Pyralidae; Galleriinae |
| *Galleria mellonella* | 5 | 1 | 0 | Endopterygota; Amphiesmenoptera; Lepidoptera; Glossata; Neolepidoptera; Heteroneura; Ditrysia; Obtectomera; Pyraloidea; Pyralidae; Galleriinae |
| *Amyelois transitella* | 3 | 0 | 1 | Endopterygota; Amphiesmenoptera; Lepidoptera; Glossata; Neolepidoptera; Heteroneura; Ditrysia; Obtectomera; Pyraloidea; Pyralidae; Phycitinae |
| *Plodia interpunctella* | 3 | 0 | 1 | Endopterygota; Amphiesmenoptera; Lepidoptera; Glossata; Neolepidoptera; Heteroneura; Ditrysia; Obtectomera; Pyraloidea; Pyralidae; Phycitinae |
| *Manduca sexta* | 4 | 1 | 0 | Endopterygota; Amphiesmenoptera; Lepidoptera; Glossata; Neolepidoptera; Heteroneura; Ditrysia; Obtectomera; Bombycoidea; Sphingidae; Sphinginae; Sphingini |
| *Bombyx mandarina* | 1 | 0 | 1 | Endopterygota; Amphiesmenoptera; Lepidoptera; Glossata; Neolepidoptera; Heteroneura; Ditrysia; Obtectomera; Bombycoidea; Bombycidae; Bombycinae |
| *Bombyx mori* | 2 | 0 | 0 | Endopterygota; Amphiesmenoptera; Lepidoptera; Glossata; Neolepidoptera; Heteroneura; Ditrysia; Obtectomera; Bombycoidea; Bombycidae; Bombycinae |
| *Arctia plantaginis* | 7 | 4 | 2 | Endopterygota; Amphiesmenoptera; Lepidoptera; Glossata; Neolepidoptera; Heteroneura; Ditrysia; Obtectomera; Noctuoidea; Erebidae; Arctiinae; Arctiini |
| *Trichoplusia ni* | 4 | 0 | 1 | Endopterygota; Amphiesmenoptera; Lepidoptera; Glossata; Neolepidoptera; Heteroneura; Ditrysia; Obtectomera; Noctuoidea; Noctuidae; Plusiinae |
| *Helicoverpa armigera* | 3 | 0 | 0 | Endopterygota; Amphiesmenoptera; Lepidoptera; Glossata; Neolepidoptera; Heteroneura; Ditrysia; Obtectomera; Noctuoidea; Noctuidae; Heliothinae |
| *Helicoverpa zea* | 3 | 0 | 0 | Endopterygota; Amphiesmenoptera; Lepidoptera; Glossata; Neolepidoptera; Heteroneura; Ditrysia; Obtectomera; Noctuoidea; Noctuidae; Heliothinae |
| *Spodoptera frugiperda* | 4 | 0 | 0 | Endopterygota; Amphiesmenoptera; Lepidoptera; Glossata; Neolepidoptera; Heteroneura; Ditrysia; Obtectomera; Noctuoidea; Noctuidae; Amphipyrinae |
| *Spodoptera litura* | 4 | 0 | 0 | Endopterygota; Amphiesmenoptera; Lepidoptera; Glossata; Neolepidoptera; Heteroneura; Ditrysia; Obtectomera; Noctuoidea; Noctuidae; Amphipyrinae |
| *Parnassius apollo* | 8 | 3 | 1 | Endopterygota; Amphiesmenoptera; Lepidoptera; Glossata; Neolepidoptera; Heteroneura; Ditrysia; Obtectomera; Papilionoidea; Papilionidae; Parnassiinae; Parnassini |
| *Papilio polytes* | 5 | 0 | 0 | Endopterygota; Amphiesmenoptera; Lepidoptera; Glossata; Neolepidoptera; Heteroneura; Ditrysia; Obtectomera; Papilionoidea; Papilionidae; Papilioninae; Papilionini |
| *Papilio machaon* | 4 | 0 | 0 | Endopterygota; Amphiesmenoptera; Lepidoptera; Glossata; Neolepidoptera; Heteroneura; Ditrysia; Obtectomera; Papilionoidea; Papilionidae; Papilioninae; Papilionini |
| *Papilio xuthus* | 4 | 1 | 1 | Endopterygota; Amphiesmenoptera; Lepidoptera; Glossata; Neolepidoptera; Heteroneura; Ditrysia; Obtectomera; Papilionoidea; Papilionidae; Papilioninae; Papilionini |
| *Leptidea sinapis* | 2 | 0 | 2 | Endopterygota; Amphiesmenoptera; Lepidoptera; Glossata; Neolepidoptera; Heteroneura; Ditrysia; Obtectomera; Papilionoidea; Pieridae; Dismorphiinae |
| *Colias croceus* | 3 | 0 | 0 | Endopterygota; Amphiesmenoptera; Lepidoptera; Glossata; Neolepidoptera; Heteroneura; Ditrysia; Obtectomera; Papilionoidea; Pieridae; Coliadinae |
| *Zerene cesonia* | 3 | 0 | 0 | Endopterygota; Amphiesmenoptera; Lepidoptera; Glossata; Neolepidoptera; Heteroneura; Ditrysia; Obtectomera; Papilionoidea; Pieridae; Coliadinae |
| *Pieris rapae* | 3 | 0 | 0 | Endopterygota; Amphiesmenoptera; Lepidoptera; Glossata; Neolepidoptera; Heteroneura; Ditrysia; Obtectomera; Papilionoidea; Pieridae; Pierinae; Pierini |
| *Pieris brassicae* | 3 | 0 | 0 | Endopterygota; Amphiesmenoptera; Lepidoptera; Glossata; Neolepidoptera; Heteroneura; Ditrysia; Obtectomera; Papilionoidea; Pieridae; Pierinae; Pierini |
| *Pieris napi* | 3 | 0 | 0 | Endopterygota; Amphiesmenoptera; Lepidoptera; Glossata; Neolepidoptera; Heteroneura; Ditrysia; Obtectomera; Papilionoidea; Pieridae; Pierinae; Pierini |
| *Aricia agestis* | 2 | 0 | 4 | Endopterygota; Amphiesmenoptera; Lepidoptera; Glossata; Neolepidoptera; Heteroneura; Ditrysia; Obtectomera; Papilionoidea; Lycaenidae; Polyommatinae |
| *Danaus plexippus* | 2 | 0 | 4 | Endopterygota; Amphiesmenoptera; Lepidoptera; Glossata; Neolepidoptera; Heteroneura; Ditrysia; Obtectomera; Papilionoidea; Nymphalidae; Danainae; Danaini; Danaina |
| *Bicyclus anynana* | 2 | 0 | 2 | Endopterygota; Amphiesmenoptera; Lepidoptera; Glossata; Neolepidoptera; Heteroneura; Ditrysia; Obtectomera; Papilionoidea; Nymphalidae; Satyrinae; Satyrini; Mycalesina |
| *Pararge aegeria* | 2 | 0 | 2 | Endopterygota; Amphiesmenoptera; Lepidoptera; Glossata; Neolepidoptera; Heteroneura; Ditrysia; Obtectomera; Papilionoidea; Nymphalidae; Satyrinae; Satyrini; Parargina |
| *Maniola hyperantus* | 2 | 0 | 0 | Endopterygota; Amphiesmenoptera; Lepidoptera; Glossata; Neolepidoptera; Heteroneura; Ditrysia; Obtectomera; Papilionoidea; Nymphalidae; Satyrinae; Satyrini; Maniolina |
| *Maniola jurtina* | 2 | 0 | 0 | Endopterygota; Amphiesmenoptera; Lepidoptera; Glossata; Neolepidoptera; Heteroneura; Ditrysia; Obtectomera; Papilionoidea; Nymphalidae; Satyrinae; Satyrini; Maniolina |
| *Brenthis ino* | 2 | 0 | 2 | Endopterygota; Amphiesmenoptera; Lepidoptera; Glossata; Neolepidoptera; Heteroneura; Ditrysia; Obtectomera; Papilionoidea; Nymphalidae; Heliconiinae; Argynnini |
| *Melitaea cinxia* | 2 | 0 | 2 | Endopterygota; Amphiesmenoptera; Lepidoptera; Glossata; Neolepidoptera; Heteroneura; Ditrysia; Obtectomera; Papilionoidea; Nymphalidae; Nymphalinae; Melitaeini; Melitaeina |
| *Nymphalis io* | 2 | 0 | 1 | Endopterygota; Amphiesmenoptera; Lepidoptera; Glossata; Neolepidoptera; Heteroneura; Ditrysia; Obtectomera; Papilionoidea; Nymphalidae; Nymphalinae; Nymphalini |
| *Vanessa cardui* | 2 | 0 | 0 | Endopterygota; Amphiesmenoptera; Lepidoptera; Glossata; Neolepidoptera; Heteroneura; Ditrysia; Obtectomera; Papilionoidea; Nymphalidae; Nymphalinae; Nymphalini |
| *Vanessa atalanta* | 2 | 0 | 0 | Endopterygota; Amphiesmenoptera; Lepidoptera; Glossata; Neolepidoptera; Heteroneura; Ditrysia; Obtectomera; Papilionoidea; Nymphalidae; Nymphalinae; Nymphalini |
| *Vanessa tameamea* | 2 | 0 | 0 | Endopterygota; Amphiesmenoptera; Lepidoptera; Glossata; Neolepidoptera; Heteroneura; Ditrysia; Obtectomera; Papilionoidea; Nymphalidae; Nymphalinae; Nymphalini |
| *Culicoides brevitarsis* | 1 | 0 | 4 | Endopterygota; Diptera; Nematocera; Culicomorpha; Chironomoidea; Ceratopogonidae; Ceratopogoninae; Culicoidini |
| *Clunio marinus* | 4 | 1 | 2 | Endopterygota; Diptera; Nematocera; Culicomorpha; Chironomoidea; Chironomidae; Orthocladiinae |
| *Polypedilum vanderplanki* | 9 | 5 | 1 | Endopterygota; Diptera; Nematocera; Culicomorpha; Chironomoidea; Chironomidae; Chironominae; Chironomini; Polypedilum |
| *Toxorhynchites rutilus septentrionalis* | 3 | 0 | 6 | Endopterygota; Diptera; Nematocera; Culicomorpha; Culicoidea; Culicidae; Toxorhynchitinae |
| *Uranotaenia lowii* | 7 | 2 | 6 | Endopterygota; Diptera; Nematocera; Culicomorpha; Culicoidea; Culicidae; Culicinae; Uranotaeniini; Uranotaenia |
| *Malaya genurostris* | 6 | 1 | 1 | Endopterygota; Diptera; Nematocera; Culicomorpha; Culicoidea; Culicidae; Culicinae; Sabethini |
| *Topomyia yanbarensis* | 6 | 1 | 1 | Endopterygota; Diptera; Nematocera; Culicomorpha; Culicoidea; Culicidae; Culicinae; Sabethini |
| *Sabethes cyaneus* | 9 | 3 | 1 | Endopterygota; Diptera; Nematocera; Culicomorpha; Culicoidea; Culicidae; Culicinae; Sabethini |
| *Wyeomyia smithii* | 8 | 1 | 0 | Endopterygota; Diptera; Nematocera; Culicomorpha; Culicoidea; Culicidae; Culicinae; Sabethini |
| *Culex pipiens pallens* | 2 | 0 | 1 | Endopterygota; Diptera; Nematocera; Culicomorpha; Culicoidea; Culicidae; Culicinae; Culicini |
| *Culex quinquefasciatus* | 3 | 0 | 0 | Endopterygota; Diptera; Nematocera; Culicomorpha; Culicoidea; Culicidae; Culicinae; Culicini |
| *Ochlerotatus camptorhynchus* | 5 | 0 | 4 | Endopterygota; Diptera; Nematocera; Culicomorpha; Culicoidea; Culicidae; Culicinae; Aedini |
| *Armigeres subalbatus* | 7 | 0 | 2 | Endopterygota; Diptera; Nematocera; Culicomorpha; Culicoidea; Culicidae; Culicinae; Aedini |
| *Aedes aegypti* | 6 | 0 | 2 | Endopterygota; Diptera; Nematocera; Culicomorpha; Culicoidea; Culicidae; Culicinae; Aedini |
| *Aedes albopictus* | 7 | 0 | 1 | Endopterygota; Diptera; Nematocera; Culicomorpha; Culicoidea; Culicidae; Culicinae; Aedini |
| *Anopheles coustani* | 2 | 0 | 0 | Endopterygota; Diptera; Nematocera; Culicomorpha; Culicoidea; Culicidae; Anophelinae |
| *Anopheles ziemanni* | 2 | 0 | 0 | Endopterygota; Diptera; Nematocera; Culicomorpha; Culicoidea; Culicidae; Anophelinae |
| *Anopheles bellator* | 2 | 0 | 0 | Endopterygota; Diptera; Nematocera; Culicomorpha; Culicoidea; Culicidae; Anophelinae |
| *Anopheles cruzii* | 2 | 0 | 0 | Endopterygota; Diptera; Nematocera; Culicomorpha; Culicoidea; Culicidae; Anophelinae |
| *Anopheles darlingi* | 3 | 0 | 1 | Endopterygota; Diptera; Nematocera; Culicomorpha; Culicoidea; Culicidae; Anophelinae |
| *Anopheles albimanus* | 3 | 0 | 0 | Endopterygota; Diptera; Nematocera; Culicomorpha; Culicoidea; Culicidae; Anophelinae |
| *Anopheles aquasalis* | 3 | 0 | 0 | Endopterygota; Diptera; Nematocera; Culicomorpha; Culicoidea; Culicidae; Anophelinae |
| *Anopheles maculipalpis* | 7 | 1 | 1 | Endopterygota; Diptera; Nematocera; Culicomorpha; Culicoidea; Culicidae; Anophelinae |
| *Anopheles stephensi* | 5 | 0 | 2 | Endopterygota; Diptera; Nematocera; Culicomorpha; Culicoidea; Culicidae; Anophelinae |
| *Anopheles nili* | 3 | 1 | 5 | Endopterygota; Diptera; Nematocera; Culicomorpha; Culicoidea; Culicidae; Anophelinae |
| *Anopheles funestus* | 4 | 0 | 1 | Endopterygota; Diptera; Nematocera; Culicomorpha; Culicoidea; Culicidae; Anophelinae |
| *Anopheles moucheti* | 5 | 1 | 1 | Endopterygota; Diptera; Nematocera; Culicomorpha; Culicoidea; Culicidae; Anophelinae |
| *Anopheles merus* | 3 | 0 | 2 | Endopterygota; Diptera; Nematocera; Culicomorpha; Culicoidea; Culicidae; Anophelinae |
| *Anopheles arabiensis* | 5 | 0 | 1 | Endopterygota; Diptera; Nematocera; Culicomorpha; Culicoidea; Culicidae; Anophelinae |
| *Anopheles coluzzii* | 4 | 0 | 1 | Endopterygota; Diptera; Nematocera; Culicomorpha; Culicoidea; Culicidae; Anophelinae |
| *Anopheles gambiae* | 6 | 1 | 0 | Endopterygota; Diptera; Nematocera; Culicomorpha; Culicoidea; Culicidae; Anophelinae |
| *Lutzomyia longipalpis* | 6 | 2 | 2 | Endopterygota; Diptera; Nematocera; Psychodomorpha; Psychodoidea; Psychodidae; Phlebotominae |
| *Phlebotomus argentipes* | 4 | 0 | 2 | Endopterygota; Diptera; Nematocera; Psychodomorpha; Psychodoidea; Psychodidae; Phlebotominae |
| *Phlebotomus papatasi* | 3 | 0 | 3 | Endopterygota; Diptera; Nematocera; Psychodomorpha; Psychodoidea; Psychodidae; Phlebotominae |
| *Bradysia coprophila* | 2 | 0 | 1 | Endopterygota; Diptera; Nematocera; Bibionomorpha; Sciaroidea; Sciaridae |
| *Contarinia nasturtii* | 3 | 2 | 0 | Endopterygota; Diptera; Nematocera; Bibionomorpha; Sciaroidea; Cecidomyiidae; Cecidomyiinae; Cecidomyiidi; Cecidomyiini |
| *Sitodiplosis mosellana* | 1 | 0 | 0 | Endopterygota; Diptera; Nematocera; Bibionomorpha; Sciaroidea; Cecidomyiidae; Cecidomyiinae; Cecidomyiidi; Cecidomyiini |
| *Hermetia illucens* | 53 | 47 | 4 | Endopterygota; Diptera; Brachycera; Stratiomyomorpha; Stratiomyidae; Hermetiinae |
| *Condylostylus longicornis* | 4 | 1 | 4 | Endopterygota; Diptera; Brachycera; Muscomorpha; Eremoneura; Empidoidea; Dolichopodidae; Sciapodinae |
| *Episyrphus balteatus* | 5 | 0 | 0 | Endopterygota; Diptera; Brachycera; Muscomorpha; Eremoneura; Cyclorrhapha; Aschiza; Syrphoidea; Syrphidae; Syrphinae; Syrphini |
| *Eupeodes corollae* | 6 | 1 | 0 | Endopterygota; Diptera; Brachycera; Muscomorpha; Eremoneura; Cyclorrhapha; Aschiza; Syrphoidea; Syrphidae; Syrphinae; Syrphini |
| *Anastrepha ludens* | 12 | 3 | 0 | Endopterygota; Diptera; Brachycera; Muscomorpha; Eremoneura; Cyclorrhapha; Schizophora; Acalyptratae; Tephritoidea; Tephritidae; Trypetinae; Toxotrypanini |
| *Anastrepha obliqua* | 11 | 2 | 0 | Endopterygota; Diptera; Brachycera; Muscomorpha; Eremoneura; Cyclorrhapha; Schizophora; Acalyptratae; Tephritoidea; Tephritidae; Trypetinae; Toxotrypanini |
| *Ceratitis capitata* | 13 | 3 | 5 | Endopterygota; Diptera; Brachycera; Muscomorpha; Eremoneura; Cyclorrhapha; Schizophora; Acalyptratae; Tephritoidea; Tephritidae; Dacinae; Ceratitidini |
| *Zeugodacus cucurbitae* | 17 | 7 | 3 | Endopterygota; Diptera; Brachycera; Muscomorpha; Eremoneura; Cyclorrhapha; Schizophora; Acalyptratae; Tephritoidea; Tephritidae; Dacinae; Dacini |
| *Bactrocera tryoni* | 16 | 4 | 3 | Endopterygota; Diptera; Brachycera; Muscomorpha; Eremoneura; Cyclorrhapha; Schizophora; Acalyptratae; Tephritoidea; Tephritidae; Dacinae; Dacini |
| *Bactrocera dorsalis* | 15 | 3 | 3 | Endopterygota; Diptera; Brachycera; Muscomorpha; Eremoneura; Cyclorrhapha; Schizophora; Acalyptratae; Tephritoidea; Tephritidae; Dacinae; Dacini |
| *Bactrocera latifrons* | 12 | 1 | 4 | Endopterygota; Diptera; Brachycera; Muscomorpha; Eremoneura; Cyclorrhapha; Schizophora; Acalyptratae; Tephritoidea; Tephritidae; Dacinae; Dacini |
| *Glossina fuscipes* | 1 | 0 | 10 | Endopterygota; Diptera; Brachycera; Muscomorpha; Eremoneura; Cyclorrhapha; Schizophora; Calyptratae; Hippoboscoidea; Glossinidae |
| *Calliphora vicina* | 24 | 10 | 3 | Endopterygota; Diptera; Brachycera; Muscomorpha; Eremoneura; Cyclorrhapha; Schizophora; Calyptratae; Oestroidea; Calliphoridae; Calliphorinae |
| *Lucilia cuprina* | 23 | 6 | 2 | Endopterygota; Diptera; Brachycera; Muscomorpha; Eremoneura; Cyclorrhapha; Schizophora; Calyptratae; Oestroidea; Calliphoridae; Luciliinae |
| *Lucilia sericata* | 19 | 3 | 3 | Endopterygota; Diptera; Brachycera; Muscomorpha; Eremoneura; Cyclorrhapha; Schizophora; Calyptratae; Oestroidea; Calliphoridae; Luciliinae |
| *Stomoxys calcitrans* | 29 | 17 | 5 | Endopterygota; Diptera; Brachycera; Muscomorpha; Eremoneura; Cyclorrhapha; Schizophora; Calyptratae; Muscoidea; Muscidae; Muscinae; Stomoxyini |
| *Musca domestica* | 43 | 29 | 3 | Endopterygota; Diptera; Brachycera; Muscomorpha; Eremoneura; Cyclorrhapha; Schizophora; Calyptratae; Muscoidea; Muscidae; Muscinae; Muscini |
| *Musca vetustissima* | 21 | 9 | 5 | Endopterygota; Diptera; Brachycera; Muscomorpha; Eremoneura; Cyclorrhapha; Schizophora; Calyptratae; Muscoidea; Muscidae; Muscinae; Muscini |
| *Scaptodrosophila lebanonensis* | 19 | 9 | 7 | Endopterygota; Diptera; Brachycera; Muscomorpha; Eremoneura; Cyclorrhapha; Schizophora; Acalyptratae; Ephydroidea; Drosophilidae; Drosophilinae; Colocasiomyini |
| *Drosophila sulfurigaster albostrigata* | 13 | 1 | 1 | Endopterygota; Diptera; Brachycera; Muscomorpha; Eremoneura; Cyclorrhapha; Schizophora; Acalyptratae; Ephydroidea; Drosophilidae; Drosophilinae; Drosophilini |
| *Drosophila albomicans* | 12 | 1 | 2 | Endopterygota; Diptera; Brachycera; Muscomorpha; Eremoneura; Cyclorrhapha; Schizophora; Acalyptratae; Ephydroidea; Drosophilidae; Drosophilinae; Drosophilini |
| *Drosophila nasuta* | 11 | 1 | 3 | Endopterygota; Diptera; Brachycera; Muscomorpha; Eremoneura; Cyclorrhapha; Schizophora; Acalyptratae; Ephydroidea; Drosophilidae; Drosophilinae; Drosophilini |
| *Drosophila grimshawi* | 11 | 1 | 9 | Endopterygota; Diptera; Brachycera; Muscomorpha; Eremoneura; Cyclorrhapha; Schizophora; Acalyptratae; Ephydroidea; Drosophilidae; Drosophilinae; Drosophilini |
| *Drosophila montana* | 12 | 1 | 3 | Endopterygota; Diptera; Brachycera; Muscomorpha; Eremoneura; Cyclorrhapha; Schizophora; Acalyptratae; Ephydroidea; Drosophilidae; Drosophilinae; Drosophilini |
| *Drosophila novamexicana* | 13 | 1 | 1 | Endopterygota; Diptera; Brachycera; Muscomorpha; Eremoneura; Cyclorrhapha; Schizophora; Acalyptratae; Ephydroidea; Drosophilidae; Drosophilinae; Drosophilini |
| *Drosophila virilis* | 16 | 4 | 1 | Endopterygota; Diptera; Brachycera; Muscomorpha; Eremoneura; Cyclorrhapha; Schizophora; Acalyptratae; Ephydroidea; Drosophilidae; Drosophilinae; Drosophilini |
| *Drosophila hydei* | 17 | 5 | 4 | Endopterygota; Diptera; Brachycera; Muscomorpha; Eremoneura; Cyclorrhapha; Schizophora; Acalyptratae; Ephydroidea; Drosophilidae; Drosophilinae; Drosophilini |
| *Drosophila navojoa* | 12 | 3 | 3 | Endopterygota; Diptera; Brachycera; Muscomorpha; Eremoneura; Cyclorrhapha; Schizophora; Acalyptratae; Ephydroidea; Drosophilidae; Drosophilinae; Drosophilini |
| *Drosophila arizonae* | 11 | 2 | 2 | Endopterygota; Diptera; Brachycera; Muscomorpha; Eremoneura; Cyclorrhapha; Schizophora; Acalyptratae; Ephydroidea; Drosophilidae; Drosophilinae; Drosophilini |
| *Drosophila mojavensis* | 13 | 4 | 2 | Endopterygota; Diptera; Brachycera; Muscomorpha; Eremoneura; Cyclorrhapha; Schizophora; Acalyptratae; Ephydroidea; Drosophilidae; Drosophilinae; Drosophilini |
| *Drosophila obscura* | 12 | 4 | 5 | Endopterygota; Diptera; Brachycera; Muscomorpha; Eremoneura; Cyclorrhapha; Schizophora; Acalyptratae; Ephydroidea; Drosophilidae; Drosophilinae; Drosophilini |
| *Drosophila guanche* | 13 | 4 | 2 | Endopterygota; Diptera; Brachycera; Muscomorpha; Eremoneura; Cyclorrhapha; Schizophora; Acalyptratae; Ephydroidea; Drosophilidae; Drosophilinae; Drosophilini |
| *Drosophila subobscura* | 13 | 4 | 2 | Endopterygota; Diptera; Brachycera; Muscomorpha; Eremoneura; Cyclorrhapha; Schizophora; Acalyptratae; Ephydroidea; Drosophilidae; Drosophilinae; Drosophilini |
| *Drosophila miranda* | 19 | 6 | 1 | Endopterygota; Diptera; Brachycera; Muscomorpha; Eremoneura; Cyclorrhapha; Schizophora; Acalyptratae; Ephydroidea; Drosophilidae; Drosophilinae; Drosophilini |
| *Drosophila persimilis* | 16 | 3 | 1 | Endopterygota; Diptera; Brachycera; Muscomorpha; Eremoneura; Cyclorrhapha; Schizophora; Acalyptratae; Ephydroidea; Drosophilidae; Drosophilinae; Drosophilini |
| *Drosophila pseudoobscura* | 14 | 2 | 2 | Endopterygota; Diptera; Brachycera; Muscomorpha; Eremoneura; Cyclorrhapha; Schizophora; Acalyptratae; Ephydroidea; Drosophilidae; Drosophilinae; Drosophilini |
| *Drosophila ananassae* | 14 | 4 | 3 | Endopterygota; Diptera; Brachycera; Muscomorpha; Eremoneura; Cyclorrhapha; Schizophora; Acalyptratae; Ephydroidea; Drosophilidae; Drosophilinae; Drosophilini |
| *Drosophila bipectinata* | 14 | 3 | 2 | Endopterygota; Diptera; Brachycera; Muscomorpha; Eremoneura; Cyclorrhapha; Schizophora; Acalyptratae; Ephydroidea; Drosophilidae; Drosophilinae; Drosophilini |
| *Drosophila kikkawai* | 22 | 10 | 2 | Endopterygota; Diptera; Brachycera; Muscomorpha; Eremoneura; Cyclorrhapha; Schizophora; Acalyptratae; Ephydroidea; Drosophilidae; Drosophilinae; Drosophilini |
| *Drosophila serrata* | 16 | 3 | 1 | Endopterygota; Diptera; Brachycera; Muscomorpha; Eremoneura; Cyclorrhapha; Schizophora; Acalyptratae; Ephydroidea; Drosophilidae; Drosophilinae; Drosophilini |
| *Drosophila ficusphila* | 12 | 2 | 13 | Endopterygota; Diptera; Brachycera; Muscomorpha; Eremoneura; Cyclorrhapha; Schizophora; Acalyptratae; Ephydroidea; Drosophilidae; Drosophilinae; Drosophilini |
| *Drosophila elegans* | 10 | 1 | 5 | Endopterygota; Diptera; Brachycera; Muscomorpha; Eremoneura; Cyclorrhapha; Schizophora; Acalyptratae; Ephydroidea; Drosophilidae; Drosophilinae; Drosophilini |
| *Drosophila rhopaloa* | 15 | 4 | 3 | Endopterygota; Diptera; Brachycera; Muscomorpha; Eremoneura; Cyclorrhapha; Schizophora; Acalyptratae; Ephydroidea; Drosophilidae; Drosophilinae; Drosophilini |
| *Drosophila takahashii* | 15 | 2 | 6 | Endopterygota; Diptera; Brachycera; Muscomorpha; Eremoneura; Cyclorrhapha; Schizophora; Acalyptratae; Ephydroidea; Drosophilidae; Drosophilinae; Drosophilini |
| *Drosophila biarmipes* | 13 | 3 | 5 | Endopterygota; Diptera; Brachycera; Muscomorpha; Eremoneura; Cyclorrhapha; Schizophora; Acalyptratae; Ephydroidea; Drosophilidae; Drosophilinae; Drosophilini |
| *Drosophila subpulchrella* | 12 | 3 | 1 | Endopterygota; Diptera; Brachycera; Muscomorpha; Eremoneura; Cyclorrhapha; Schizophora; Acalyptratae; Ephydroidea; Drosophilidae; Drosophilinae; Drosophilini |
| *Drosophila suzukii* | 12 | 3 | 1 | Endopterygota; Diptera; Brachycera; Muscomorpha; Eremoneura; Cyclorrhapha; Schizophora; Acalyptratae; Ephydroidea; Drosophilidae; Drosophilinae; Drosophilini |
| *Drosophila eugracilis* | 14 | 2 | 10 | Endopterygota; Diptera; Brachycera; Muscomorpha; Eremoneura; Cyclorrhapha; Schizophora; Acalyptratae; Ephydroidea; Drosophilidae; Drosophilinae; Drosophilini |
| *Drosophila melanogaster* | 11 | 0 | 8 | Endopterygota; Diptera; Brachycera; Muscomorpha; Eremoneura; Cyclorrhapha; Schizophora; Acalyptratae; Ephydroidea; Drosophilidae; Drosophilinae; Drosophilini |
| *Drosophila erecta* | 12 | 2 | 6 | Endopterygota; Diptera; Brachycera; Muscomorpha; Eremoneura; Cyclorrhapha; Schizophora; Acalyptratae; Ephydroidea; Drosophilidae; Drosophilinae; Drosophilini |
| *Drosophila teissieri* | 13 | 2 | 3 | Endopterygota; Diptera; Brachycera; Muscomorpha; Eremoneura; Cyclorrhapha; Schizophora; Acalyptratae; Ephydroidea; Drosophilidae; Drosophilinae; Drosophilini |
| *Drosophila yakuba* | 12 | 1 | 3 | Endopterygota; Diptera; Brachycera; Muscomorpha; Eremoneura; Cyclorrhapha; Schizophora; Acalyptratae; Ephydroidea; Drosophilidae; Drosophilinae; Drosophilini |
| *Drosophila sechellia* | 10 | 0 | 3 | Endopterygota; Diptera; Brachycera; Muscomorpha; Eremoneura; Cyclorrhapha; Schizophora; Acalyptratae; Ephydroidea; Drosophilidae; Drosophilinae; Drosophilini |
| *Drosophila mauritiana* | 12 | 2 | 3 | Endopterygota; Diptera; Brachycera; Muscomorpha; Eremoneura; Cyclorrhapha; Schizophora; Acalyptratae; Ephydroidea; Drosophilidae; Drosophilinae; Drosophilini |
| *Drosophila simulans* | 13 | 2 | 2 | Endopterygota; Diptera; Brachycera; Muscomorpha; Eremoneura; Cyclorrhapha; Schizophora; Acalyptratae; Ephydroidea; Drosophilidae; Drosophilinae; Drosophilini |

**Table S2 –** Speciation, duplication and loss events inferred by GeneRax for the C-type lysozyme gene family at internal nodes of the species tree.

| **Node** | **Copy number (speciations)** | **Duplications** | **Losses** |
| --- | --- | --- | --- |
| Node Abscondita terminalis Lamprigera yunnana | 3 | 1 | 0 |
| Node Achroia grisella Amyelois transitella | 4 | 0 | 3 |
| Node Achroia grisella Galleria mellonella | 4 | 1 | 1 |
| Node Aedes aegypti Aedes albopictus | 8 | 1 | 2 |
| Node Aethina tumida Brassicogethes aeneus | 4 | 0 | 3 |
| Node Aethina tumida Cylas formicarius | 7 | 3 | 2 |
| Node Agrilus planipennis Ignelater luminosus | 3 | 0 | 3 |
| Node Agrilus planipennis Onthophagus taurus | 6 | 1 | 5 |
| Node Amyelois transitella Plodia interpunctella | 4 | 0 | 0 |
| Node Anabrus simplex Bacillus rossius redtenbacheri | 12 | 0 | 6 |
| Node Anabrus simplex Frankliniella occidentalis | 18 | 6 | 0 |
| Node Anabrus simplex Gryllus longicercus | 6 | 0 | 6 |
| Node Anastrepha ludens Anastrepha obliqua | 9 | 1 | 8 |
| Node Anastrepha ludens Ceratitis capitata | 16 | 3 | 4 |
| Node Anastrepha ludens Glossina fuscipes | 17 | 1 | 6 |
| Node Anastrepha ludens Scaptodrosophila lebanonensis | 22 | 18 | 2 |
| Node Anopheles albimanus Anopheles aquasalis | 3 | 0 | 1 |
| Node Anopheles arabiensis Anopheles coluzzii | 6 | 1 | 0 |
| Node Anopheles bellator Anopheles cruzii | 2 | 0 | 2 |
| Node Anopheles bellator Anopheles darlingi | 4 | 0 | 4 |
| Node Anopheles bellator Anopheles maculipalpis | 8 | 1 | 0 |
| Node Anopheles coluzzii Anopheles gambiae | 5 | 0 | 1 |
| Node Anopheles coustani Anopheles bellator | 7 | 0 | 1 |
| Node Anopheles coustani Anopheles ziemanni | 2 | 1 | 6 |
| Node Anopheles darlingi Anopheles albimanus | 4 | 0 | 0 |
| Node Anopheles funestus Anopheles moucheti | 5 | 0 | 2 |
| Node Anopheles maculipalpis Anopheles nili | 10 | 2 | 0 |
| Node Anopheles maculipalpis Anopheles stephensi | 7 | 0 | 3 |
| Node Anopheles merus Anopheles arabiensis | 5 | 0 | 4 |
| Node Anopheles nili Anopheles funestus | 7 | 0 | 2 |
| Node Anopheles nili Anopheles merus | 9 | 1 | 2 |
| Node Anoplophora glabripennis Gonioctena quinquepunctata | 6 | 0 | 1 |
| Node Anthonomus grandis grandis Dendroctonus ponderosae | 5 | 1 | 2 |
| Node Anthonomus grandis grandis Rhynchophorus ferrugineus | 6 | 0 | 0 |
| Node Aphidius gifuensis Chelonus insularis | 1 | 0 | 0 |
| Node Aphidius gifuensis Fopius arisanus | 0 | 0 | 1 |
| Node Apolygus lucorum Cimex lectularius | 4 | 0 | 2 |
| Node Arctia plantaginis Trichoplusia ni | 5 | 0 | 0 |
| Node Aricia agestis Danaus plexippus | 6 | 0 | 1 |
| Node Armigeres subalbatus Aedes aegypti | 9 | 1 | 1 |
| Node Athalia rosae Cephus cinctus | 4 | 0 | 10 |
| Node Athalia rosae Chrysoperla carnea | 14 | 0 | 0 |
| Node Athalia rosae Diprion similis | 3 | 0 | 1 |
| Node Bacillus rossius redtenbacheri Blattella germanica | 8 | 1 | 5 |
| Node Bactrocera dorsalis Bactrocera latifrons | 15 | 0 | 0 |
| Node Bactrocera tryoni Bactrocera dorsalis | 15 | 4 | 2 |
| Node Belonocnema kinseyi Polistes dominula | 1 | 0 | 0 |
| Node Belonocnema kinseyi Trichogramma pretiosum | 0 | 0 | 1 |
| Node Belonocnema kinseyi Venturia canescens | 1 | 0 | 0 |
| Node Bemisia tabaci Planococcus citri | 1 | 0 | 6 |
| Node Bicyclus anynana Brenthis ino | 6 | 0 | 0 |
| Node Bicyclus anynana Maniola hyperantus | 4 | 0 | 2 |
| Node Bicyclus anynana Pararge aegeria | 4 | 0 | 0 |
| Node Blattella germanica Periplaneta americana | 9 | 2 | 1 |
| Node Bombyx mandarina Bombyx mori | 2 | 0 | 1 |
| Node Bradysia coprophila Contarinia nasturtii | 3 | 0 | 7 |
| Node Bradysia coprophila Hermetia illucens | 10 | 0 | 2 |
| Node Brenthis ino Melitaea cinxia | 4 | 1 | 3 |
| Node Calliphora vicina Lucilia cuprina | 17 | 5 | 5 |
| Node Calliphora vicina Stomoxys calcitrans | 17 | 6 | 0 |
| Node Cephus cinctus Orussus abietinus | 2 | 0 | 2 |
| Node Ceratitis capitata Zeugodacus cucurbitae | 15 | 0 | 1 |
| Node Chelonus insularis Cotesia glomerata | 1 | 0 | 0 |
| Node Chilo suppressalis Achroia grisella | 7 | 0 | 1 |
| Node Chilo suppressalis Manduca sexta | 8 | 0 | 3 |
| Node Chilo suppressalis Ostrinia furnacalis | 5 | 0 | 2 |
| Node Chilo suppressalis Parnassius apollo | 11 | 1 | 0 |
| Node Chrysoperla carnea Agrilus planipennis | 10 | 0 | 5 |
| Node Chrysoperla carnea Plutella xylostella | 15 | 3 | 2 |
| Node Cloeon dipterum Anabrus simplex | 12 | 6 | 0 |
| Node Cloeon dipterum Hetaerina americana | 3 | 0 | 9 |
| Node Cloeon dipterum Neocloeon triangulifer | 1 | 0 | 2 |
| Node Clunio marinus Polypedilum vanderplanki | 5 | 1 | 1 |
| Node Coccinella septempunctata Aethina tumida | 6 | 0 | 0 |
| Node Coccinella septempunctata Harmonia axyridis | 4 | 1 | 3 |
| Node Colias croceus Pieris rapae | 4 | 0 | 0 |
| Node Colias croceus Zerene cesonia | 3 | 0 | 1 |
| Node Condylostylus longicornis Episyrphus balteatus | 7 | 1 | 4 |
| Node Contarinia nasturtii Sitodiplosis mosellana | 1 | 0 | 2 |
| Node Coptotermes formosanus Cryptotermes secundus | 6 | 2 | 0 |
| Node Cotesia glomerata Microplitis demolitor | 0 | 0 | 1 |
| Node Culex pipiens pallens Culex quinquefasciatus | 3 | 0 | 7 |
| Node Culex pipiens pallens Ochlerotatus camptorhynchus | 10 | 0 | 1 |
| Node Culicoides brevitarsis Clunio marinus | 5 | 0 | 3 |
| Node Culicoides brevitarsis Lutzomyia longipalpis | 14 | 4 | 2 |
| Node Culicoides brevitarsis Toxorhynchites rutilus septentrionalis | 8 | 0 | 6 |
| Node Cydia pomonella Cydia splendana | 4 | 0 | 0 |
| Node Cylas formicarius Anoplophora glabripennis | 7 | 0 | 0 |
| Node Cylas formicarius Anthonomus grandis grandis | 6 | 2 | 3 |
| Node Dalotia coriaria Nicrophorus vespilloides | 3 | 1 | 2 |
| Node Danaus plexippus Bicyclus anynana | 6 | 0 | 0 |
| Node Diabrotica virgifera virgifera Diorhabda carinulata | 6 | 2 | 0 |
| Node Diorhabda carinulata Diorhabda sublineata | 4 | 0 | 2 |
| Node Diprion similis Neodiprion fabricii | 3 | 0 | 0 |
| Node Drosophila albomicans Drosophila nasuta | 13 | 0 | 0 |
| Node Drosophila ananassae Drosophila bipectinata | 13 | 1 | 13 |
| Node Drosophila ananassae Drosophila kikkawai | 25 | 3 | 3 |
| Node Drosophila arizonae Drosophila mojavensis | 11 | 1 | 2 |
| Node Drosophila biarmipes Drosophila subpulchrella | 15 | 0 | 4 |
| Node Drosophila elegans Drosophila rhopaloa | 14 | 0 | 9 |
| Node Drosophila elegans Drosophila takahashii | 23 | 1 | 1 |
| Node Drosophila erecta Drosophila sechellia | 19 | 0 | 0 |
| Node Drosophila erecta Drosophila teissieri | 16 | 0 | 3 |
| Node Drosophila eugracilis Drosophila melanogaster | 22 | 0 | 4 |
| Node Drosophila ficusphila Drosophila elegans | 23 | 1 | 2 |
| Node Drosophila grimshawi Drosophila montana | 19 | 1 | 0 |
| Node Drosophila guanche Drosophila subobscura | 11 | 0 | 2 |
| Node Drosophila hydei Drosophila navojoa | 16 | 2 | 4 |
| Node Drosophila kikkawai Drosophila ficusphila | 24 | 1 | 2 |
| Node Drosophila kikkawai Drosophila serrata | 14 | 4 | 14 |
| Node Drosophila mauritiana Drosophila simulans | 13 | 0 | 0 |
| Node Drosophila melanogaster Drosophila erecta | 19 | 2 | 5 |
| Node Drosophila miranda Drosophila persimilis | 14 | 3 | 5 |
| Node Drosophila montana Drosophila hydei | 18 | 0 | 1 |
| Node Drosophila montana Drosophila novamexicana | 14 | 0 | 4 |
| Node Drosophila navojoa Drosophila arizonae | 12 | 0 | 4 |
| Node Drosophila novamexicana Drosophila virilis | 13 | 2 | 3 |
| Node Drosophila obscura Drosophila ananassae | 25 | 6 | 5 |
| Node Drosophila obscura Drosophila guanche | 13 | 0 | 3 |
| Node Drosophila obscura Drosophila miranda | 16 | 2 | 11 |
| Node Drosophila persimilis Drosophila pseudoobscura | 14 | 1 | 1 |
| Node Drosophila sechellia Drosophila mauritiana | 13 | 1 | 7 |
| Node Drosophila subpulchrella Drosophila suzukii | 10 | 0 | 5 |
| Node Drosophila sulfurigaster albostrigata Drosophila albomicans | 13 | 4 | 9 |
| Node Drosophila sulfurigaster albostrigata Drosophila grimshawi | 18 | 2 | 8 |
| Node Drosophila sulfurigaster albostrigata Drosophila obscura | 24 | 9 | 2 |
| Node Drosophila takahashii Drosophila biarmipes | 19 | 0 | 7 |
| Node Drosophila takahashii Drosophila eugracilis | 26 | 6 | 3 |
| Node Drosophila teissieri Drosophila yakuba | 14 | 1 | 3 |
| Node Episyrphus balteatus Anastrepha ludens | 6 | 0 | 1 |
| Node Episyrphus balteatus Eupeodes corollae | 5 | 1 | 2 |
| Node Eriocheir sinensis Scylla paramamosain | 1 | 0 | 1 |
| Node Folsomia candida Cloeon dipterum | 6 | 3 | 0 |
| Node Frankliniella occidentalis Homalodisca vitripennis | 9 | 0 | 7 |
| Node Frankliniella occidentalis Pediculus humanus corporis | 16 | 1 | 3 |
| Node Frankliniella occidentalis Thrips palmi | 6 | 3 | 6 |
| Node Glossina fuscipes Calliphora vicina | 11 | 0 | 6 |
| Node Gonioctena quinquepunctata Diabrotica virgifera virgifera | 4 | 0 | 2 |
| Node Gonioctena quinquepunctata Leptinotarsa decemlineata | 2 | 0 | 2 |
| Node Halyomorpha halys Rhodnius prolixus | 6 | 0 | 1 |
| Node Helicoverpa armigera Helicoverpa zea | 3 | 0 | 2 |
| Node Helicoverpa armigera Spodoptera frugiperda | 5 | 0 | 0 |
| Node Hermetia illucens Condylostylus longicornis | 10 | 1 | 1 |
| Node Hetaerina americana Ischnura elegans | 2 | 0 | 1 |
| Node Homalodisca vitripennis Bemisia tabaci | 7 | 0 | 2 |
| Node Homalodisca vitripennis Halyomorpha halys | 7 | 0 | 0 |
| Node Homalodisca vitripennis Laodelphax striatellus | 2 | 0 | 5 |
| Node Hyposmocoma kahamanoa Chilo suppressalis | 10 | 2 | 0 |
| Node Hyposmocoma kahamanoa Pectinophora gossypiella | 6 | 0 | 4 |
| Node Ignelater luminosus Abscondita terminalis | 2 | 0 | 1 |
| Node Lamprigera yunnana Photinus pyralis | 2 | 0 | 1 |
| Node Laodelphax striatellus Nilaparvata lugens | 1 | 0 | 1 |
| Node Leguminivora glycinivorella Cydia pomonella | 4 | 1 | 5 |
| Node Leguminivora glycinivorella Hyposmocoma kahamanoa | 8 | 4 | 0 |
| Node Leptidea sinapis Aricia agestis | 7 | 1 | 1 |
| Node Leptidea sinapis Colias croceus | 4 | 0 | 3 |
| Node Lucilia cuprina Lucilia sericata | 19 | 4 | 2 |
| Node Lutzomyia longipalpis Bradysia coprophila | 12 | 2 | 4 |
| Node Lutzomyia longipalpis Phlebotomus argentipes | 6 | 2 | 8 |
| Node Malaya genurostris Culex pipiens pallens | 11 | 1 | 1 |
| Node Malaya genurostris Sabethes cyaneus | 8 | 1 | 4 |
| Node Malaya genurostris Topomyia yanbarensis | 6 | 1 | 3 |
| Node Manduca sexta Arctia plantaginis | 5 | 0 | 3 |
| Node Manduca sexta Bombyx mandarina | 3 | 0 | 2 |
| Node Maniola hyperantus Maniola jurtina | 2 | 0 | 2 |
| Node Melitaea cinxia Nymphalis io | 4 | 1 | 1 |
| Node Musca domestica Musca vetustissima | 17 | 5 | 5 |
| Node Neodiprion fabricii Neodiprion lecontei | 3 | 1 | 1 |
| Node Neodiprion fabricii Neodiprion virginianus | 3 | 0 | 0 |
| Node Neodiprion lecontei Neodiprion pinetum | 2 | 0 | 1 |
| Node Nymphalis io Vanessa cardui | 3 | 0 | 1 |
| Node Ochlerotatus camptorhynchus Armigeres subalbatus | 9 | 1 | 2 |
| Node Onthophagus taurus Dalotia coriaria | 4 | 0 | 1 |
| Node Onthophagus taurus Tenebrio molitor | 5 | 0 | 1 |
| Node Orussus abietinus Belonocnema kinseyi | 1 | 0 | 1 |
| Node Ostrinia furnacalis Ostrinia nubilalis | 5 | 1 | 1 |
| Node Papilio machaon Papilio xuthus | 4 | 0 | 1 |
| Node Papilio polytes Papilio machaon | 5 | 0 | 1 |
| Node Parnassius apollo Leptidea sinapis | 7 | 0 | 4 |
| Node Parnassius apollo Papilio polytes | 6 | 3 | 4 |
| Node Pediculus humanus corporis Athalia rosae | 14 | 0 | 2 |
| Node Periplaneta americana Zootermopsis nevadensis | 7 | 0 | 2 |
| Node Phlebotomus argentipes Phlebotomus papatasi | 6 | 0 | 0 |
| Node Pieris brassicae Pieris napi | 3 | 0 | 0 |
| Node Pieris rapae Pieris brassicae | 3 | 0 | 1 |
| Node Planococcus citri Adelges cooleyi | 0 | 0 | 1 |
| Node Plutella xylostella Culicoides brevitarsis | 12 | 0 | 3 |
| Node Plutella xylostella Leguminivora glycinivorella | 4 | 1 | 9 |
| Node Polistes dominula Ampulex compressa | 0 | 0 | 1 |
| Node Procambarus clarkii Eriocheir sinensis | 2 | 0 | 1 |
| Node Procambarus clarkii Folsomia candida | 3 | 2 | 0 |
| Node Rhodnius prolixus Apolygus lucorum | 6 | 0 | 0 |
| Node Rhynchophorus ferrugineus Sitophilus oryzae | 3 | 0 | 3 |
| Node Sabethes cyaneus Wyeomyia smithii | 7 | 0 | 1 |
| Node Scaptodrosophila lebanonensis Drosophila sulfurigaster albostrigata | 17 | 2 | 7 |
| Node Spodoptera frugiperda Spodoptera litura | 4 | 0 | 1 |
| Node Stomoxys calcitrans Musca domestica | 17 | 3 | 3 |
| Node Tenebrio molitor Coccinella septempunctata | 6 | 3 | 2 |
| Node Tenebrio molitor Tribolium castaneum | 3 | 0 | 3 |
| Node Tenebrio molitor Zophobas morio | 3 | 0 | 0 |
| Node Toxorhynchites rutilus septentrionalis Anopheles coustani | 8 | 4 | 4 |
| Node Toxorhynchites rutilus septentrionalis Uranotaenia lowii | 9 | 2 | 1 |
| Node Tribolium castaneum Tribolium madens | 4 | 1 | 0 |
| Node Trichoplusia ni Helicoverpa armigera | 5 | 1 | 1 |
| Node Uranotaenia lowii Malaya genurostris | 11 | 2 | 0 |
| Node Vanessa atalanta Vanessa tameamea | 2 | 0 | 0 |
| Node Vanessa cardui Vanessa atalanta | 2 | 0 | 1 |
| Node Venturia canescens Aphidius gifuensis | 1 | 0 | 0 |
| Node Zeugodacus cucurbitae Bactrocera tryoni | 13 | 0 | 2 |
| Node Zootermopsis nevadensis Coptotermes formosanus | 4 | 0 | 3 |

***** Internal nodes with no inferred C-type lysozyme lineages are omitted.

**Table S3 –** Species-level gene duplication, loss and speciation events inferred by GeneRax for the I-type lysozyme gene family across Arthropoda

| **Species** | **Copy number (specia-tions)** | **Duplica-tions** | **Losses** | **Lineage** |
| --- | --- | --- | --- | --- |
| *Procambarus clarkii* | 4 | 1 | 0 | Crustacea |
| *Eriocheir sinensis* | 4 | 2 | 0 | Crustacea |
| *Scylla paramamosain* | 2 | 1 | 1 | Crustacea |
| *Folsomia candida* | 4 | 2 | 4 | Collembola |
| *Ischnura elegans* | 3 | 1 | 0 | Palaeoptera; Odonata; Zygoptera; Coenagrionoidea; Coenagrionidae |
| *Hetaerina americana* | 2 | 0 | 0 | Palaeoptera; Odonata; Zygoptera; Calopterygoidea; Calopterygidae |
| *Cloeon dipterum* | 2 | 0 | 1 | Palaeoptera; Ephemeroptera; Pisciforma; Baetidae |
| *Neocloeon triangulifer* | 4 | 1 | 0 | Palaeoptera; Ephemeroptera; Pisciforma; Baetidae |
| *Gryllus longicercus* | 4 | 0 | 4 | Polyneoptera; Orthoptera; Ensifera; Gryllidea; Grylloidea ; Gryllidae ; Gryllinae |
| *Anabrus simplex* | 8 | 1 | 1 | Polyneoptera ; Orthoptera ; Ensifera ; Tettigoniidea ; Tettigonioidea ; Tettigoniidae ; Tettigoniinae |
| *Bacillus rossius redtenbacheri* | 6 | 0 | 1 | Polyneoptera; Phasmatodea; Verophasmatodea; Areolatae; Bacilloidea; Bacillidae; Bacillinae; Bacillini |
| *Blattella germanica* | 5 | 1 | 3 | Polyneoptera; Dictyoptera; Blattodea; Blaberoidea; Blattellidae |
| *Periplaneta americana* | 7 | 2 | 2 | Polyneoptera ; Dictyoptera ; Blattodea ; Blattoidea ; Blattidae ; Blattinae |
| *Zootermopsis nevadensis* | 6 | 0 | 0 | Polyneoptera; Dictyoptera; Blattodea; Blattoidea; Termitoidae; Termopsidae; Termopsinae; Termopsini |
| *Coptotermes formosanus* | 3 | 0 | 2 | Polyneoptera; Dictyoptera; Blattodea; Blattoidea; Termitoidae; Rhinotermitidae; Coptotermitinae |
| *Cryptotermes secundus* | 6 | 2 | 1 | Polyneoptera; Dictyoptera; Blattodea; Blattoidea; Termitoidae; Kalotermitidae; Cryptotermitinae |
| *Frankliniella occidentalis* | 6 | 1 | 0 | Paraneoptera; Thysanoptera; Terebrantia; Thripoidea; Thripidae; Thripinae |
| *Thrips palmi* | 4 | 0 | 1 | Paraneoptera; Thysanoptera; Terebrantia; Thripoidea; Thripidae; Thripinae |
| *Homalodisca vitripennis* | 8 | 3 | 3 | Paraneoptera; Hemiptera; Auchenorrhyncha; Cicadomorpha; Membracoidea; Cicadellidae; Cicadellinae; Proconiini |
| *Laodelphax striatellus* | 9 | 1 | 0 | Paraneoptera; Hemiptera; Auchenorrhyncha; Fulgoromorpha; Fulgoroidea; Delphacidae; Criomorphinae |
| *Nilaparvata lugens* | 15 | 7 | 0 | Paraneoptera; Hemiptera; Auchenorrhyncha; Fulgoromorpha; Fulgoroidea; Delphacidae; Delphacinae |
| *Halyomorpha halys* | 2 | 1 | 2 | Paraneoptera; Hemiptera; Prosorrhyncha; Heteroptera; Euheteroptera; Neoheteroptera; Panheteroptera; Pentatomomorpha; Pentatomoidea; Pentatomidae; Pentatominae |
| *Rhodnius prolixus* | 2 | 1 | 2 | Paraneoptera; Hemiptera; Prosorrhyncha; Heteroptera; Euheteroptera; Neoheteroptera; Panheteroptera; Cimicomorpha; Reduvioidea; Reduviidae; Triatominae |
| *Apolygus lucorum* | 1 | 0 | 2 | Paraneoptera; Hemiptera; Prosorrhyncha; Heteroptera; Euheteroptera; Neoheteroptera; Panheteroptera; Cimicomorpha; Cimicoidea; Miridae; Mirinae; Mirini |
| *Cimex lectularius* | 4 | 2 | 1 | Paraneoptera; Hemiptera; Prosorrhyncha; Heteroptera; Euheteroptera; Neoheteroptera; Panheteroptera; Cimicomorpha; Cimicoidea; Cimicidae |
| *Bemisia tabaci* | 3 | 0 | 3 | Paraneoptera; Hemiptera; Sternorrhyncha; Aleyrodoidea; Aleyrodidae; Aleyrodinae |
| *Planococcus citri* | 4 | 0 | 2 | Paraneoptera; Hemiptera; Sternorrhyncha; Coccoidea; Pseudococcidae |
| *Adelges cooleyi* | 4 | 1 | 0 | Paraneoptera; Hemiptera; Sternorrhyncha; Aphidomorpha; Phylloxeroidea; Adelgidae; Adelges; Gilletteella |
| *Daktulosphaira vitifoliae* | 4 | 1 | 0 | Paraneoptera; Hemiptera; Sternorrhyncha; Aphidomorpha; Phylloxeroidea; Phylloxeridae |
| *Sipha flava* | 3 | 0 | 1 | Paraneoptera; Hemiptera; Sternorrhyncha; Aphidomorpha; Aphidoidea; Aphididae; Chaitophorinae |
| *Acyrthosiphon pisum* | 3 | 0 | 0 | Paraneoptera; Hemiptera; Sternorrhyncha; Aphidomorpha; Aphidoidea; Aphididae; Aphidinae; Macrosiphini |
| *Myzus persicae* | 3 | 0 | 0 | Paraneoptera; Hemiptera; Sternorrhyncha; Aphidomorpha; Aphidoidea; Aphididae; Aphidinae; Macrosiphini |
| *Melanaphis sacchari* | 3 | 0 | 0 | Paraneoptera; Hemiptera; Sternorrhyncha; Aphidomorpha; Aphidoidea; Aphididae; Aphidinae; Aphidini |
| *Aphis craccivora* | 3 | 0 | 0 | Paraneoptera; Hemiptera; Sternorrhyncha; Aphidomorpha; Aphidoidea; Aphididae; Aphidinae; Aphidini; Aphis |
| *Aphis gossypii* | 4 | 1 | 0 | Paraneoptera; Hemiptera; Sternorrhyncha; Aphidomorpha; Aphidoidea; Aphididae; Aphidinae; Aphidini |
| *Rhopalosiphum maidis* | 3 | 0 | 0 | Paraneoptera; Hemiptera; Sternorrhyncha; Aphidomorpha; Aphidoidea; Aphididae; Aphidinae; Aphidini |
| *Rhopalosiphum padi* | 3 | 0 | 0 | Paraneoptera; Hemiptera; Sternorrhyncha; Aphidomorpha; Aphidoidea; Aphididae; Aphidinae; Aphidini |
| *Pediculus humanus corporis* | 3 | 0 | 11 | Paraneoptera; Psocodea; Phthiraptera; Anoplura; Pediculidae |
| *Athalia rosae* | 1 | 0 | 0 | Endopterygota; Hymenoptera; Tenthredinoidea; Athaliidae |
| *Diprion similis* | 1 | 0 | 0 | Endopterygota; Hymenoptera; Tenthredinoidea; Diprionidae; Diprioninae |
| *Neodiprion fabricii* | 1 | 0 | 0 | Endopterygota; Hymenoptera; Tenthredinoidea; Diprionidae; Diprioninae |
| *Neodiprion virginianus* | 1 | 0 | 0 | Endopterygota; Hymenoptera; Tenthredinoidea; Diprionidae; Diprioninae |
| *Neodiprion lecontei* | 1 | 0 | 0 | Endopterygota; Hymenoptera; Tenthredinoidea; Diprionidae; Diprioninae |
| *Neodiprion pinetum* | 1 | 0 | 0 | Endopterygota; Hymenoptera; Tenthredinoidea; Diprionidae; Diprioninae |
| *Cephus cinctus* | 1 | 0 | 2 | Endopterygota; Hymenoptera; Cephoidea; Cephidae |
| *Orussus abietinus* | 1 | 0 | 3 | Endopterygota; Hymenoptera; Orussoidea; Orussidae |
| *Belonocnema kinseyi* | 2 | 0 | 0 | Endopterygota; Hymenoptera; Apocrita; Proctotrupomorpha; Cynipoidea; Cynipidae; Cynipinae; Cynipini |
| *Leptopilina boulardi* | 1 | 0 | 0 | Endopterygota; Hymenoptera; Apocrita; Proctotrupomorpha; Cynipoidea; Figitidae; Eucoilinae |
| *Leptopilina heterotoma* | 1 | 0 | 0 | Endopterygota; Hymenoptera; Apocrita; Proctotrupomorpha; Cynipoidea; Figitidae; Eucoilinae |
| *Trichogramma pretiosum* | 2 | 1 | 1 | Endopterygota; Hymenoptera; Apocrita; Proctotrupomorpha; Chalcidoidea; Trichogrammatidae |
| *Copidosoma floridanum* | 2 | 0 | 0 | Endopterygota; Hymenoptera; Apocrita; Proctotrupomorpha; Chalcidoidea; Encyrtidae; Encyrtinae |
| *Phymastichus coffea* | 1 | 0 | 0 | Endopterygota; Hymenoptera; Apocrita; Proctotrupomorpha; Chalcidoidea; Eulophidae; Tetrastichinae |
| *Ceratosolen solmsi marchali* | 1 | 0 | 0 | Endopterygota; Hymenoptera; Apocrita; Proctotrupomorpha; Chalcidoidea; Agaonidae; Agaoninae |
| *Nasonia vitripennis* | 1 | 0 | 0 | Endopterygota; Hymenoptera; Apocrita; Proctotrupomorpha; Chalcidoidea; Pteromalidae; Pteromalinae |
| *Venturia canescens* | 1 | 0 | 2 | Endopterygota; Hymenoptera; Apocrita; Ichneumonoidea; Ichneumonidae; Campopleginae; Dusona group |
| *Aphidius gifuensis* | 2 | 0 | 0 | Endopterygota; Hymenoptera; Apocrita; Ichneumonoidea; Braconidae; Aphidiinae |
| *Fopius arisanus* | 1 | 0 | 0 | Endopterygota; Hymenoptera; Apocrita; Ichneumonoidea; Braconidae; Opiinae |
| *Diachasma alloeum* | 2 | 0 | 0 | Endopterygota; Hymenoptera; Apocrita; Ichneumonoidea; Braconidae; Opiinae |
| *Diachasmimorpha longicaudata* | 2 | 0 | 0 | Endopterygota; Hymenoptera; Apocrita; Ichneumonoidea; Braconidae; Opiinae; Diachasmimorpha |
| *Chelonus insularis* | 1 | 0 | 0 | Endopterygota; Hymenoptera; Apocrita; Ichneumonoidea; Braconidae; Cheloninae |
| *Cotesia glomerata* | 4 | 3 | 0 | Endopterygota; Hymenoptera; Apocrita; Ichneumonoidea; Braconidae; Microgastrinae |
| *Microplitis demolitor* | 1 | 0 | 0 | Endopterygota; Hymenoptera; Apocrita; Ichneumonoidea; Braconidae; Microgastrinae |
| *Microplitis mediator* | 1 | 0 | 0 | Endopterygota; Hymenoptera; Apocrita; Ichneumonoidea; Braconidae; Microgastrinae |
| *Polistes dominula* | 1 | 0 | 0 | Endopterygota; Hymenoptera; Apocrita; Aculeata; Vespoidea; Vespidae; Polistinae; Polistini |
| *Polistes canadensis* | 1 | 0 | 0 | Endopterygota; Hymenoptera; Apocrita; Aculeata; Vespoidea; Vespidae; Polistinae; Polistini |
| *Polistes fuscatus* | 1 | 0 | 0 | Endopterygota; Hymenoptera; Apocrita; Aculeata; Vespoidea; Vespidae; Polistinae; Polistini |
| *Vespula pensylvanica* | 1 | 0 | 0 | Endopterygota; Hymenoptera; Apocrita; Aculeata; Vespoidea; Vespidae; Vespinae |
| *Vespula vulgaris* | 1 | 0 | 0 | Endopterygota; Hymenoptera; Apocrita; Aculeata; Vespoidea; Vespidae; Vespinae |
| *Vespa velutina* | 1 | 0 | 0 | Endopterygota; Hymenoptera; Apocrita; Aculeata; Vespoidea; Vespidae; Vespinae |
| *Vespa crabro* | 1 | 0 | 0 | Endopterygota; Hymenoptera; Apocrita; Aculeata; Vespoidea; Vespidae; Vespinae |
| *Vespa mandarinia* | 1 | 0 | 0 | Endopterygota; Hymenoptera; Apocrita; Aculeata; Vespoidea; Vespidae; Vespinae |
| *Ampulex compressa* | 1 | 0 | 1 | Endopterygota; Hymenoptera; Apocrita; Aculeata; Apoidea; Ampulicidae; Ampulicini |
| *Colletes gigas* | 1 | 0 | 0 | Endopterygota; Hymenoptera; Apocrita; Aculeata; Apoidea; Anthophila; Colletidae; Colletinae |
| *Hylaeus anthracinus* | 1 | 0 | 0 | Endopterygota; Hymenoptera; Apocrita; Aculeata; Apoidea; Anthophila; Colletidae; Hylaeinae; Hylaeus; Nesoprosopis |
| *Hylaeus volcanicus* | 1 | 0 | 0 | Endopterygota; Hymenoptera; Apocrita; Aculeata; Apoidea; Anthophila; Colletidae; Hylaeinae; Hylaeus; Nesoprosopis |
| *Dufourea novaeangliae* | 1 | 0 | 0 | Endopterygota; Hymenoptera; Apocrita; Aculeata; Apoidea; Anthophila; Halictidae; Rophitinae |
| *Megalopta genalis* | 1 | 0 | 0 | Endopterygota; Hymenoptera; Apocrita; Aculeata; Apoidea; Anthophila; Halictidae; Halictinae; Augochlorini |
| *Nomia melanderi* | 1 | 0 | 0 | Endopterygota; Hymenoptera; Apocrita; Aculeata; Apoidea; Anthophila; Halictidae; Nomiinae; Nomia; Acunomia |
| *Megachile rotundata* | 1 | 0 | 0 | Endopterygota; Hymenoptera; Apocrita; Aculeata; Apoidea; Anthophila; Megachilidae; Megachilinae; Megachilini |
| *Osmia bicornis bicornis* | 1 | 0 | 0 | Endopterygota; Hymenoptera; Apocrita; Aculeata; Apoidea; Anthophila; Megachilidae; Megachilinae; Osmiini; Osmia |
| *Osmia lignaria* | 2 | 1 | 0 | Endopterygota; Hymenoptera; Apocrita; Aculeata; Apoidea; Anthophila; Megachilidae; Megachilinae; Osmiini |
| *Ceratina calcarata* | 1 | 0 | 0 | Endopterygota; Hymenoptera; Apocrita; Aculeata; Apoidea; Anthophila; Apidae; Xylocopinae; Ceratinini; Ceratina |
| *Eufriesea mexicana* | 1 | 0 | 0 | Endopterygota; Hymenoptera; Apocrita; Aculeata; Apoidea; Anthophila; Apidae; Apinae; Euglossini |
| *Bombus affinis* | 1 | 0 | 0 | Endopterygota; Hymenoptera; Apocrita; Aculeata; Apoidea; Anthophila; Apidae; Apinae; Bombini |
| *Bombus terrestris* | 1 | 0 | 0 | Endopterygota; Hymenoptera; Apocrita; Aculeata; Apoidea; Anthophila; Apidae; Apinae; Bombini |
| *Frieseomelitta varia* | 3 | 2 | 0 | Endopterygota; Hymenoptera; Apocrita; Aculeata; Apoidea; Anthophila; Apidae; Apinae; Meliponini |
| *Melipona quadrifasciata* | 1 | 0 | 0 | Endopterygota; Hymenoptera; Apocrita; Aculeata; Apoidea; Anthophila; Apidae; Apinae; Meliponini |
| *Apis florea* | 1 | 0 | 0 | Endopterygota; Hymenoptera; Apocrita; Aculeata; Apoidea; Anthophila; Apidae; Apinae; Apini |
| *Apis cerana* | 1 | 0 | 0 | Endopterygota; Hymenoptera; Apocrita; Aculeata; Apoidea; Anthophila; Apidae; Apinae; Apini |
| *Apis mellifera* | 1 | 0 | 0 | Endopterygota; Hymenoptera; Apocrita; Aculeata; Apoidea; Anthophila; Apidae; Apinae; Apini |
| *Apis dorsata* | 1 | 0 | 0 | Endopterygota; Hymenoptera; Apocrita; Aculeata; Apoidea; Anthophila; Apidae; Apinae; Apini |
| *Apis laboriosa* | 1 | 0 | 0 | Endopterygota; Hymenoptera; Apocrita; Aculeata; Apoidea; Anthophila; Apidae; Apinae; Apini |
| *Dinoponera quadriceps* | 1 | 0 | 0 | Endopterygota; Hymenoptera; Apocrita; Aculeata; Formicoidea; Formicidae; Ponerinae; Ponerini |
| *Harpegnathos saltator* | 1 | 0 | 0 | Endopterygota; Hymenoptera; Apocrita; Aculeata; Formicoidea; Formicidae; Ponerinae; Ponerini |
| *Odontomachus brunneus* | 1 | 0 | 0 | Endopterygota; Hymenoptera; Apocrita; Aculeata; Formicoidea; Formicidae; Ponerinae; Ponerini |
| *Eciton burchellii* | 1 | 0 | 1 | Endopterygota; Hymenoptera; Apocrita; Aculeata; Formicoidea; Formicidae; Dorylinae |
| *Ooceraea biroi* | 1 | 0 | 1 | Endopterygota; Hymenoptera; Apocrita; Aculeata; Formicoidea; Formicidae; Dorylinae |
| *Linepithema humile* | 1 | 0 | 1 | Endopterygota; Hymenoptera; Apocrita; Aculeata; Formicoidea; Formicidae; Dolichoderinae |
| *Pseudomyrmex gracilis* | 1 | 0 | 1 | Endopterygota; Hymenoptera; Apocrita; Aculeata; Formicoidea; Formicidae; Pseudomyrmecinae |
| *Nylanderia fulva* | 1 | 0 | 3 | Endopterygota; Hymenoptera; Apocrita; Aculeata; Formicoidea; Formicidae; Formicinae; Lasiini |
| *Camponotus floridanus* | 1 | 0 | 3 | Endopterygota; Hymenoptera; Apocrita; Aculeata; Formicoidea; Formicidae; Formicinae; Camponotini |
| *Cataglyphis hispanica* | 2 | 0 | 1 | Endopterygota; Hymenoptera; Apocrita; Aculeata; Formicoidea; Formicidae; Formicinae; Formicini |
| *Formica exsecta* | 3 | 1 | 1 | Endopterygota; Hymenoptera; Apocrita; Aculeata; Formicoidea; Formicidae; Formicinae; Formicini |
| *Pogonomyrmex barbatus* | 1 | 0 | 2 | Endopterygota; Hymenoptera; Apocrita; Aculeata; Formicoidea; Formicidae; Myrmicinae; Pogonomyrmecini |
| *Vollenhovia emeryi* | 3 | 1 | 3 | Endopterygota; Hymenoptera; Apocrita; Aculeata; Formicoidea; Formicidae; Myrmicinae; Stenammini |
| *Temnothorax curvispinosus* | 3 | 1 | 1 | Endopterygota; Hymenoptera; Apocrita; Aculeata; Formicoidea; Formicidae; Myrmicinae; Crematogastrini |
| *Temnothorax longispinosus* | 3 | 0 | 0 | Endopterygota; Hymenoptera; Apocrita; Aculeata; Formicoidea; Formicidae; Myrmicinae; Crematogastrini |
| *Monomorium pharaonis* | 3 | 0 | 0 | Endopterygota; Hymenoptera; Apocrita; Aculeata; Formicoidea; Formicidae; Myrmicinae; Solenopsidini |
| *Solenopsis invicta* | 3 | 0 | 0 | Endopterygota; Hymenoptera; Apocrita; Aculeata; Formicoidea; Formicidae; Myrmicinae; Solenopsidini |
| *Wasmannia auropunctata* | 4 | 2 | 3 | Endopterygota; Hymenoptera; Apocrita; Aculeata; Formicoidea; Formicidae; Myrmicinae; Blepharidattini |
| *Cyphomyrmex costatus* | 2 | 0 | 2 | Endopterygota; Hymenoptera; Apocrita; Aculeata; Formicoidea; Formicidae; Myrmicinae; Attini |
| *Trachymyrmex septentrionalis* | 3 | 0 | 0 | Endopterygota; Hymenoptera; Apocrita; Aculeata; Formicoidea; Formicidae; Myrmicinae; Attini |
| *Acromyrmex echinatior* | 3 | 0 | 0 | Endopterygota; Hymenoptera; Apocrita; Aculeata; Formicoidea; Formicidae; Myrmicinae; Attini |
| *Atta cephalotes* | 3 | 0 | 0 | Endopterygota; Hymenoptera; Apocrita; Aculeata; Formicoidea; Formicidae; Myrmicinae; Attini |
| *Atta colombica* | 3 | 0 | 0 | Endopterygota; Hymenoptera; Apocrita; Aculeata; Formicoidea; Formicidae; Myrmicinae; Attini |
| *Chrysoperla carnea* | 1 | 0 | 7 | Endopterygota; Neuropterida; Neuroptera; Hemerobiiformia; Chrysopidae; Chrysopinae |
| *Agrilus planipennis* | 2 | 0 | 4 | Endopterygota; Coleoptera; Polyphaga; Elateriformia; Buprestoidea; Buprestidae; Agrilinae |
| *Ignelater luminosus* | 6 | 2 | 2 | Endopterygota; Coleoptera; Polyphaga; Elateriformia; Elateroidea; Elateridae; Agrypninae; Pyrophorini |
| *Abscondita terminalis* | 5 | 0 | 1 | Endopterygota; Coleoptera; Polyphaga; Elateriformia; Elateroidea; Lampyridae; Luciolinae |
| *Lamprigera yunnana* | 7 | 2 | 2 | Endopterygota; Coleoptera; Polyphaga; Elateriformia; Elateroidea; Lampyridae; Lampyrinae |
| *Photinus pyralis* | 7 | 2 | 2 | Endopterygota; Coleoptera; Polyphaga; Elateriformia; Elateroidea; Lampyridae; Lampyrinae |
| *Onthophagus taurus* | 5 | 1 | 2 | Endopterygota; Coleoptera; Polyphaga; Scarabaeiformia; Scarabaeoidea; Scarabaeidae; Scarabaeinae; Onthophagini |
| *Dalotia coriaria* | 2 | 0 | 1 | Endopterygota; Coleoptera; Polyphaga; Staphyliniformia; Staphylinoidea; Staphylinidae; Tachyporinae group; Aleocharinae; Athetini; |
| *Nicrophorus vespilloides* | 4 | 1 | 0 | Endopterygota; Coleoptera; Polyphaga; Staphyliniformia; Staphylinoidea; Silphidae; Nicrophorinae |
| *Tenebrio molitor* | 10 | 6 | 4 | Endopterygota; Coleoptera; Polyphaga; Cucujiformia; Tenebrionoidea; Tenebrionidae; Tenebrioninae |
| *Zophobas morio* | 14 | 7 | 1 | Endopterygota; Coleoptera; Polyphaga; Cucujiformia; Tenebrionoidea; Tenebrionidae; Tenebrioninae |
| *Tribolium castaneum* | 4 | 1 | 0 | Endopterygota; Coleoptera; Polyphaga; Cucujiformia; Tenebrionoidea; Tenebrionidae |
| *Tribolium madens* | 4 | 1 | 0 | Endopterygota; Coleoptera; Polyphaga; Cucujiformia; Tenebrionoidea; Tenebrionidae |
| *Coccinella septempunctata* | 5 | 0 | 1 | Endopterygota; Coleoptera; Polyphaga; Cucujiformia; Coccinelloidea; Coccinellidae; Coccinellinae; Coccinellini |
| *Harmonia axyridis* | 6 | 0 | 0 | Endopterygota; Coleoptera; Polyphaga; Cucujiformia; Coccinelloidea; Coccinellidae; Coccinellinae; Coccinellini |
| *Aethina tumida* | 8 | 2 | 2 | Endopterygota; Coleoptera; Polyphaga; Cucujiformia; Cucujoidea; Nitidulidae; Nitidulinae |
| *Brassicogethes aeneus* | 7 | 1 | 2 | Endopterygota; Coleoptera; Polyphaga; Cucujiformia; Cucujoidea; Nitidulidae; Meligethinae |
| *Cylas formicarius* | 3 | 1 | 0 | Endopterygota; Coleoptera; Polyphaga; Cucujiformia; Curculionoidea; Brentidae; Cyladinae |
| *Anthonomus grandis grandis* | 1 | 0 | 0 | Endopterygota; Coleoptera; Polyphaga; Cucujiformia; Curculionoidea; Curculionidae; Curculioninae; Anthonomini |
| *Dendroctonus ponderosae* | 1 | 0 | 0 | Endopterygota; Coleoptera; Polyphaga; Cucujiformia; Curculionoidea; Curculionidae; Scolytinae |
| *Rhynchophorus ferrugineus* | 2 | 0 | 0 | Endopterygota; Coleoptera; Polyphaga; Cucujiformia; Curculionoidea; Curculionidae; Dryophthorinae |
| *Sitophilus oryzae* | 3 | 1 | 0 | Endopterygota; Coleoptera; Polyphaga; Cucujiformia; Curculionoidea; Curculionidae; Dryophthorinae |
| *Anoplophora glabripennis* | 3 | 0 | 1 | Endopterygota; Coleoptera; Polyphaga; Cucujiformia; Chrysomeloidea; Cerambycidae; Lamiinae; Lamiini |
| *Gonioctena quinquepunctata* | 2 | 0 | 0 | Endopterygota; Coleoptera; Polyphaga; Cucujiformia; Chrysomeloidea; Chrysomelidae; Chrysomelinae; Chrysomelini |
| *Leptinotarsa decemlineata* | 2 | 0 | 0 | Endopterygota; Coleoptera; Polyphaga; Cucujiformia; Chrysomeloidea; Chrysomelidae; Chrysomelinae; Doryphorini |
| *Diabrotica virgifera virgifera* | 3 | 1 | 0 | Endopterygota; Coleoptera; Polyphaga; Cucujiformia; Chrysomeloidea; Chrysomelidae; Galerucinae; Luperini; Diabroticina; Diabroticites |
| *Diorhabda carinulata* | 2 | 0 | 0 | Endopterygota; Coleoptera; Polyphaga; Cucujiformia; Chrysomeloidea; Chrysomelidae; Galerucinae; Galerucini; Atysites |
| *Diorhabda sublineata* | 4 | 2 | 0 | Endopterygota; Coleoptera; Polyphaga; Cucujiformia; Chrysomeloidea; Chrysomelidae; Galerucinae; Galerucini; Atysites |
| *Plutella xylostella* | 1 | 0 | 3 | Endopterygota; Amphiesmenoptera; Lepidoptera; Glossata; Neolepidoptera; Heteroneura; Ditrysia; Yponomeutoidea; Plutellidae |
| *Leguminivora glycinivorella* | 4 | 1 | 0 | Endopterygota; Amphiesmenoptera; Lepidoptera; Glossata; Neolepidoptera; Heteroneura; Ditrysia; Apoditrysia; Tortricoidea; Tortricidae; Olethreutinae; Grapholitini |
| *Cydia pomonella* | 3 | 0 | 0 | Endopterygota; Amphiesmenoptera; Lepidoptera; Glossata; Neolepidoptera; Heteroneura; Ditrysia; Apoditrysia; Tortricoidea; Tortricidae; Olethreutinae; Grapholitini |
| *Cydia splendana* | 3 | 0 | 0 | Endopterygota; Amphiesmenoptera; Lepidoptera; Glossata; Neolepidoptera; Heteroneura; Ditrysia; Apoditrysia; Tortricoidea; Tortricidae; Olethreutinae; Grapholitini |
| *Hyposmocoma kahamanoa* | 5 | 2 | 1 | Endopterygota; Amphiesmenoptera; Lepidoptera; Glossata; Neolepidoptera; Heteroneura; Ditrysia; Gelechioidea; Cosmopterigidae; Cosmopteriginae |
| *Pectinophora gossypiella* | 3 | 0 | 1 | Endopterygota; Amphiesmenoptera; Lepidoptera; Glossata; Neolepidoptera; Heteroneura; Ditrysia; Gelechioidea; Gelechiidae; Apatetrinae |
| *Chilo suppressalis* | 6 | 3 | 4 | Endopterygota; Amphiesmenoptera; Lepidoptera; Glossata; Neolepidoptera; Heteroneura; Ditrysia; Obtectomera; Pyraloidea; Crambidae; Crambinae |
| *Ostrinia furnacalis* | 4 | 0 | 0 | Endopterygota; Amphiesmenoptera; Lepidoptera; Glossata; Neolepidoptera; Heteroneura; Ditrysia; Obtectomera; Pyraloidea; Crambidae; Pyraustinae |
| *Ostrinia nubilalis* | 4 | 0 | 0 | Endopterygota; Amphiesmenoptera; Lepidoptera; Glossata; Neolepidoptera; Heteroneura; Ditrysia; Obtectomera; Pyraloidea; Crambidae; Pyraustinae |
| *Achroia grisella* | 4 | 1 | 0 | Endopterygota; Amphiesmenoptera; Lepidoptera; Glossata; Neolepidoptera; Heteroneura; Ditrysia; Obtectomera; Pyraloidea; Pyralidae; Galleriinae |
| *Galleria mellonella* | 6 | 3 | 0 | Endopterygota; Amphiesmenoptera; Lepidoptera; Glossata; Neolepidoptera; Heteroneura; Ditrysia; Obtectomera; Pyraloidea; Pyralidae; Galleriinae |
| *Amyelois transitella* | 3 | 0 | 0 | Endopterygota; Amphiesmenoptera; Lepidoptera; Glossata; Neolepidoptera; Heteroneura; Ditrysia; Obtectomera; Pyraloidea; Pyralidae; Phycitinae |
| *Plodia interpunctella* | 3 | 0 | 0 | Endopterygota; Amphiesmenoptera; Lepidoptera; Glossata; Neolepidoptera; Heteroneura; Ditrysia; Obtectomera; Pyraloidea; Pyralidae; Phycitinae |
| *Manduca sexta* | 5 | 2 | 2 | Endopterygota; Amphiesmenoptera; Lepidoptera; Glossata; Neolepidoptera; Heteroneura; Ditrysia; Obtectomera; Bombycoidea; Sphingidae; Sphinginae; Sphingini |
| *Bombyx mandarina* | 3 | 0 | 0 | Endopterygota; Amphiesmenoptera; Lepidoptera; Glossata; Neolepidoptera; Heteroneura; Ditrysia; Obtectomera; Bombycoidea; Bombycidae; Bombycinae |
| *Bombyx mori* | 3 | 0 | 0 | Endopterygota; Amphiesmenoptera; Lepidoptera; Glossata; Neolepidoptera; Heteroneura; Ditrysia; Obtectomera; Bombycoidea; Bombycidae; Bombycinae |
| *Arctia plantaginis* | 4 | 1 | 3 | Endopterygota; Amphiesmenoptera; Lepidoptera; Glossata; Neolepidoptera; Heteroneura; Ditrysia; Obtectomera; Noctuoidea; Erebidae; Arctiinae; Arctiini |
| *Trichoplusia ni* | 3 | 0 | 0 | Endopterygota; Amphiesmenoptera; Lepidoptera; Glossata; Neolepidoptera; Heteroneura; Ditrysia; Obtectomera; Noctuoidea; Noctuidae; Plusiinae |
| *Helicoverpa armigera* | 3 | 0 | 0 | Endopterygota; Amphiesmenoptera; Lepidoptera; Glossata; Neolepidoptera; Heteroneura; Ditrysia; Obtectomera; Noctuoidea; Noctuidae; Heliothinae |
| *Helicoverpa zea* | 3 | 0 | 0 | Endopterygota; Amphiesmenoptera; Lepidoptera; Glossata; Neolepidoptera; Heteroneura; Ditrysia; Obtectomera; Noctuoidea; Noctuidae; Heliothinae |
| *Spodoptera frugiperda* | 3 | 0 | 0 | Endopterygota; Amphiesmenoptera; Lepidoptera; Glossata; Neolepidoptera; Heteroneura; Ditrysia; Obtectomera; Noctuoidea; Noctuidae; Amphipyrinae |
| *Spodoptera litura* | 3 | 0 | 0 | Endopterygota; Amphiesmenoptera; Lepidoptera; Glossata; Neolepidoptera; Heteroneura; Ditrysia; Obtectomera; Noctuoidea; Noctuidae; Amphipyrinae |
| *Parnassius apollo* | 6 | 2 | 1 | Endopterygota; Amphiesmenoptera; Lepidoptera; Glossata; Neolepidoptera; Heteroneura; Ditrysia; Obtectomera; Papilionoidea; Papilionidae; Parnassiinae; Parnassini |
| *Papilio polytes* | 5 | 2 | 1 | Endopterygota; Amphiesmenoptera; Lepidoptera; Glossata; Neolepidoptera; Heteroneura; Ditrysia; Obtectomera; Papilionoidea; Papilionidae; Papilioninae; Papilionini |
| *Papilio machaon* | 3 | 0 | 1 | Endopterygota; Amphiesmenoptera; Lepidoptera; Glossata; Neolepidoptera; Heteroneura; Ditrysia; Obtectomera; Papilionoidea; Papilionidae; Papilioninae; Papilionini |
| *Papilio xuthus* | 3 | 0 | 1 | Endopterygota; Amphiesmenoptera; Lepidoptera; Glossata; Neolepidoptera; Heteroneura; Ditrysia; Obtectomera; Papilionoidea; Papilionidae; Papilioninae; Papilionini |
| *Leptidea sinapis* | 2 | 0 | 2 | Endopterygota; Amphiesmenoptera; Lepidoptera; Glossata; Neolepidoptera; Heteroneura; Ditrysia; Obtectomera; Papilionoidea; Pieridae; Dismorphiinae |
| *Colias croceus* | 3 | 0 | 1 | Endopterygota; Amphiesmenoptera; Lepidoptera; Glossata; Neolepidoptera; Heteroneura; Ditrysia; Obtectomera; Papilionoidea; Pieridae; Coliadinae |
| *Zerene cesonia* | 4 | 1 | 1 | Endopterygota; Amphiesmenoptera; Lepidoptera; Glossata; Neolepidoptera; Heteroneura; Ditrysia; Obtectomera; Papilionoidea; Pieridae; Coliadinae |
| *Pieris rapae* | 4 | 0 | 0 | Endopterygota; Amphiesmenoptera; Lepidoptera; Glossata; Neolepidoptera; Heteroneura; Ditrysia; Obtectomera; Papilionoidea; Pieridae; Pierinae; Pierini |
| *Pieris brassicae* | 5 | 1 | 0 | Endopterygota; Amphiesmenoptera; Lepidoptera; Glossata; Neolepidoptera; Heteroneura; Ditrysia; Obtectomera; Papilionoidea; Pieridae; Pierinae; Pierini |
| *Pieris napi* | 4 | 0 | 0 | Endopterygota; Amphiesmenoptera; Lepidoptera; Glossata; Neolepidoptera; Heteroneura; Ditrysia; Obtectomera; Papilionoidea; Pieridae; Pierinae; Pierini |
| *Aricia agestis* | 5 | 1 | 3 | Endopterygota; Amphiesmenoptera; Lepidoptera; Glossata; Neolepidoptera; Heteroneura; Ditrysia; Obtectomera; Papilionoidea; Lycaenidae; Polyommatinae |
| *Danaus plexippus* | 8 | 4 | 3 | Endopterygota; Amphiesmenoptera; Lepidoptera; Glossata; Neolepidoptera; Heteroneura; Ditrysia; Obtectomera; Papilionoidea; Nymphalidae; Danainae; Danaini; Danaina |
| *Bicyclus anynana* | 2 | 0 | 1 | Endopterygota; Amphiesmenoptera; Lepidoptera; Glossata; Neolepidoptera; Heteroneura; Ditrysia; Obtectomera; Papilionoidea; Nymphalidae; Satyrinae; Satyrini; Mycalesina |
| *Pararge aegeria* | 3 | 0 | 0 | Endopterygota; Amphiesmenoptera; Lepidoptera; Glossata; Neolepidoptera; Heteroneura; Ditrysia; Obtectomera; Papilionoidea; Nymphalidae; Satyrinae; Satyrini; Parargina |
| *Maniola hyperantus* | 3 | 0 | 1 | Endopterygota; Amphiesmenoptera; Lepidoptera; Glossata; Neolepidoptera; Heteroneura; Ditrysia; Obtectomera; Papilionoidea; Nymphalidae; Satyrinae; Satyrini; Maniolina |
| *Maniola jurtina* | 4 | 0 | 0 | Endopterygota; Amphiesmenoptera; Lepidoptera; Glossata; Neolepidoptera; Heteroneura; Ditrysia; Obtectomera; Papilionoidea; Nymphalidae; Satyrinae; Satyrini; Maniolina |
| *Brenthis ino* | 3 | 0 | 2 | Endopterygota; Amphiesmenoptera; Lepidoptera; Glossata; Neolepidoptera; Heteroneura; Ditrysia; Obtectomera; Papilionoidea; Nymphalidae; Heliconiinae; Argynnini |
| *Melitaea cinxia* | 3 | 0 | 1 | Endopterygota; Amphiesmenoptera; Lepidoptera; Glossata; Neolepidoptera; Heteroneura; Ditrysia; Obtectomera; Papilionoidea; Nymphalidae; Nymphalinae; Melitaeini; Melitaeina |
| *Nymphalis io* | 3 | 0 | 1 | Endopterygota; Amphiesmenoptera; Lepidoptera; Glossata; Neolepidoptera; Heteroneura; Ditrysia; Obtectomera; Papilionoidea; Nymphalidae; Nymphalinae; Nymphalini |
| *Vanessa cardui* | 3 | 0 | 0 | Endopterygota; Amphiesmenoptera; Lepidoptera; Glossata; Neolepidoptera; Heteroneura; Ditrysia; Obtectomera; Papilionoidea; Nymphalidae; Nymphalinae; Nymphalini |
| *Vanessa atalanta* | 3 | 0 | 0 | Endopterygota; Amphiesmenoptera; Lepidoptera; Glossata; Neolepidoptera; Heteroneura; Ditrysia; Obtectomera; Papilionoidea; Nymphalidae; Nymphalinae; Nymphalini |
| *Vanessa tameamea* | 4 | 1 | 0 | Endopterygota; Amphiesmenoptera; Lepidoptera; Glossata; Neolepidoptera; Heteroneura; Ditrysia; Obtectomera; Papilionoidea; Nymphalidae; Nymphalinae; Nymphalini |
| *Culicoides brevitarsis* | 2 | 0 | 2 | Endopterygota; Diptera; Nematocera; Culicomorpha; Chironomoidea; Ceratopogonidae; Ceratopogoninae; Culicoidini |
| *Clunio marinus* | 1 | 0 | 1 | Endopterygota; Diptera; Nematocera; Culicomorpha; Chironomoidea; Chironomidae; Orthocladiinae |
| *Polypedilum vanderplanki* | 2 | 0 | 0 | Endopterygota; Diptera; Nematocera; Culicomorpha; Chironomoidea; Chironomidae; Chironominae; Chironomini; Polypedilum |
| *Toxorhynchites rutilus septentrionalis* | 2 | 0 | 0 | Endopterygota; Diptera; Nematocera; Culicomorpha; Culicoidea; Culicidae; Toxorhynchitinae |
| *Uranotaenia lowii* | 2 | 0 | 1 | Endopterygota; Diptera; Nematocera; Culicomorpha; Culicoidea; Culicidae; Culicinae; Uranotaeniini; Uranotaenia |
| *Malaya genurostris* | 2 | 0 | 0 | Endopterygota; Diptera; Nematocera; Culicomorpha; Culicoidea; Culicidae; Culicinae; Sabethini |
| *Topomyia yanbarensis* | 2 | 0 | 0 | Endopterygota; Diptera; Nematocera; Culicomorpha; Culicoidea; Culicidae; Culicinae; Sabethini |
| *Sabethes cyaneus* | 2 | 0 | 0 | Endopterygota; Diptera; Nematocera; Culicomorpha; Culicoidea; Culicidae; Culicinae; Sabethini |
| *Wyeomyia smithii* | 2 | 0 | 0 | Endopterygota; Diptera; Nematocera; Culicomorpha; Culicoidea; Culicidae; Culicinae; Sabethini |
| *Culex pipiens pallens* | 2 | 0 | 0 | Endopterygota; Diptera; Nematocera; Culicomorpha; Culicoidea; Culicidae; Culicinae; Culicini |
| *Culex quinquefasciatus* | 3 | 1 | 0 | Endopterygota; Diptera; Nematocera; Culicomorpha; Culicoidea; Culicidae; Culicinae; Culicini |
| *Ochlerotatus camptorhynchus* | 1 | 0 | 1 | Endopterygota; Diptera; Nematocera; Culicomorpha; Culicoidea; Culicidae; Culicinae; Aedini |
| *Armigeres subalbatus* | 2 | 0 | 0 | Endopterygota; Diptera; Nematocera; Culicomorpha; Culicoidea; Culicidae; Culicinae; Aedini |
| *Aedes aegypti* | 2 | 0 | 0 | Endopterygota; Diptera; Nematocera; Culicomorpha; Culicoidea; Culicidae; Culicinae; Aedini |
| *Aedes albopictus* | 2 | 0 | 0 | Endopterygota; Diptera; Nematocera; Culicomorpha; Culicoidea; Culicidae; Culicinae; Aedini |
| *Anopheles coustani* | 2 | 0 | 0 | Endopterygota; Diptera; Nematocera; Culicomorpha; Culicoidea; Culicidae; Anophelinae |
| *Anopheles ziemanni* | 2 | 0 | 0 | Endopterygota; Diptera; Nematocera; Culicomorpha; Culicoidea; Culicidae; Anophelinae |
| *Anopheles bellator* | 1 | 0 | 1 | Endopterygota; Diptera; Nematocera; Culicomorpha; Culicoidea; Culicidae; Anophelinae |
| *Anopheles cruzii* | 2 | 0 | 0 | Endopterygota; Diptera; Nematocera; Culicomorpha; Culicoidea; Culicidae; Anophelinae |
| *Anopheles darlingi* | 2 | 0 | 0 | Endopterygota; Diptera; Nematocera; Culicomorpha; Culicoidea; Culicidae; Anophelinae |
| *Anopheles albimanus* | 3 | 1 | 0 | Endopterygota; Diptera; Nematocera; Culicomorpha; Culicoidea; Culicidae; Anophelinae |
| *Anopheles aquasalis* | 2 | 0 | 0 | Endopterygota; Diptera; Nematocera; Culicomorpha; Culicoidea; Culicidae; Anophelinae |
| *Anopheles maculipalpis* | 3 | 1 | 0 | Endopterygota; Diptera; Nematocera; Culicomorpha; Culicoidea; Culicidae; Anophelinae |
| *Anopheles stephensi* | 3 | 1 | 0 | Endopterygota; Diptera; Nematocera; Culicomorpha; Culicoidea; Culicidae; Anophelinae |
| *Anopheles nili* | 3 | 1 | 0 | Endopterygota; Diptera; Nematocera; Culicomorpha; Culicoidea; Culicidae; Anophelinae |
| *Anopheles funestus* | 2 | 0 | 0 | Endopterygota; Diptera; Nematocera; Culicomorpha; Culicoidea; Culicidae; Anophelinae |
| *Anopheles moucheti* | 2 | 0 | 0 | Endopterygota; Diptera; Nematocera; Culicomorpha; Culicoidea; Culicidae; Anophelinae |
| *Anopheles merus* | 2 | 0 | 0 | Endopterygota; Diptera; Nematocera; Culicomorpha; Culicoidea; Culicidae; Anophelinae |
| *Anopheles arabiensis* | 2 | 0 | 0 | Endopterygota; Diptera; Nematocera; Culicomorpha; Culicoidea; Culicidae; Anophelinae |
| *Anopheles coluzzii* | 2 | 0 | 0 | Endopterygota; Diptera; Nematocera; Culicomorpha; Culicoidea; Culicidae; Anophelinae |
| *Anopheles gambiae* | 2 | 0 | 0 | Endopterygota; Diptera; Nematocera; Culicomorpha; Culicoidea; Culicidae; Anophelinae |
| *Lutzomyia longipalpis* | 1 | 0 | 0 | Endopterygota; Diptera; Nematocera; Psychodomorpha; Psychodoidea; Psychodidae; Phlebotominae |
| *Phlebotomus argentipes* | 1 | 0 | 0 | Endopterygota; Diptera; Nematocera; Psychodomorpha; Psychodoidea; Psychodidae; Phlebotominae |
| *Phlebotomus papatasi* | 0 | 0 | 1 | Endopterygota; Diptera; Nematocera; Psychodomorpha; Psychodoidea; Psychodidae; Phlebotominae |
| *Bradysia coprophila* | 3 | 1 | 1 | Endopterygota; Diptera; Nematocera; Bibionomorpha; Sciaroidea; Sciaridae |
| *Contarinia nasturtii* | 1 | 0 | 0 | Endopterygota; Diptera; Nematocera; Bibionomorpha; Sciaroidea; Cecidomyiidae; Cecidomyiinae; Cecidomyiidi; Cecidomyiini |
| *Sitodiplosis mosellana* | 1 | 0 | 0 | Endopterygota; Diptera; Nematocera; Bibionomorpha; Sciaroidea; Cecidomyiidae; Cecidomyiinae; Cecidomyiidi; Cecidomyiini |
| *Hermetia illucens* | 1 | 0 | 4 | Endopterygota; Diptera; Brachycera; Stratiomyomorpha; Stratiomyidae; Hermetiinae |
| *Condylostylus longicornis* | 5 | 2 | 2 | Endopterygota; Diptera; Brachycera; Muscomorpha; Eremoneura; Empidoidea; Dolichopodidae; Sciapodinae |
| *Episyrphus balteatus* | 8 | 5 | 1 | Endopterygota; Diptera; Brachycera; Muscomorpha; Eremoneura; Cyclorrhapha; Aschiza; Syrphoidea; Syrphidae; Syrphinae; Syrphini |
| *Eupeodes corollae* | 4 | 1 | 1 | Endopterygota; Diptera; Brachycera; Muscomorpha; Eremoneura; Cyclorrhapha; Aschiza; Syrphoidea; Syrphidae; Syrphinae; Syrphini |
| *Anastrepha ludens* | 2 | 0 | 0 | Endopterygota; Diptera; Brachycera; Muscomorpha; Eremoneura; Cyclorrhapha; Schizophora; Acalyptratae; Tephritoidea; Tephritidae; Trypetinae; Toxotrypanini |
| *Anastrepha obliqua* | 2 | 0 | 0 | Endopterygota; Diptera; Brachycera; Muscomorpha; Eremoneura; Cyclorrhapha; Schizophora; Acalyptratae; Tephritoidea; Tephritidae; Trypetinae; Toxotrypanini |
| *Ceratitis capitata* | 2 | 0 | 0 | Endopterygota; Diptera; Brachycera; Muscomorpha; Eremoneura; Cyclorrhapha; Schizophora; Acalyptratae; Tephritoidea; Tephritidae; Dacinae; Ceratitidini |
| *Zeugodacus cucurbitae* | 2 | 0 | 0 | Endopterygota; Diptera; Brachycera; Muscomorpha; Eremoneura; Cyclorrhapha; Schizophora; Acalyptratae; Tephritoidea; Tephritidae; Dacinae; Dacini |
| *Bactrocera tryoni* | 2 | 0 | 0 | Endopterygota; Diptera; Brachycera; Muscomorpha; Eremoneura; Cyclorrhapha; Schizophora; Acalyptratae; Tephritoidea; Tephritidae; Dacinae; Dacini |
| *Bactrocera dorsalis* | 2 | 0 | 0 | Endopterygota; Diptera; Brachycera; Muscomorpha; Eremoneura; Cyclorrhapha; Schizophora; Acalyptratae; Tephritoidea; Tephritidae; Dacinae; Dacini |
| *Bactrocera latifrons* | 2 | 0 | 0 | Endopterygota; Diptera; Brachycera; Muscomorpha; Eremoneura; Cyclorrhapha; Schizophora; Acalyptratae; Tephritoidea; Tephritidae; Dacinae; Dacini |
| *Glossina fuscipes* | 3 | 1 | 1 | Endopterygota; Diptera; Brachycera; Muscomorpha; Eremoneura; Cyclorrhapha; Schizophora; Calyptratae; Hippoboscoidea; Glossinidae |
| *Calliphora vicina* | 3 | 1 | 0 | Endopterygota; Diptera; Brachycera; Muscomorpha; Eremoneura; Cyclorrhapha; Schizophora; Calyptratae; Oestroidea; Calliphoridae; Calliphorinae |
| *Lucilia cuprina* | 2 | 0 | 0 | Endopterygota; Diptera; Brachycera; Muscomorpha; Eremoneura; Cyclorrhapha; Schizophora; Calyptratae; Oestroidea; Calliphoridae; Luciliinae |
| *Lucilia sericata* | 3 | 1 | 0 | Endopterygota; Diptera; Brachycera; Muscomorpha; Eremoneura; Cyclorrhapha; Schizophora; Calyptratae; Oestroidea; Calliphoridae; Luciliinae |
| *Stomoxys calcitrans* | 3 | 0 | 0 | Endopterygota; Diptera; Brachycera; Muscomorpha; Eremoneura; Cyclorrhapha; Schizophora; Calyptratae; Muscoidea; Muscidae; Muscinae; Stomoxyini |
| *Musca domestica* | 4 | 1 | 0 | Endopterygota; Diptera; Brachycera; Muscomorpha; Eremoneura; Cyclorrhapha; Schizophora; Calyptratae; Muscoidea; Muscidae; Muscinae; Muscini |
| *Musca vetustissima* | 3 | 0 | 0 | Endopterygota; Diptera; Brachycera; Muscomorpha; Eremoneura; Cyclorrhapha; Schizophora; Calyptratae; Muscoidea; Muscidae; Muscinae; Muscini |
| *Scaptodrosophila lebanonensis* | 5 | 0 | 1 | Endopterygota; Diptera; Brachycera; Muscomorpha; Eremoneura; Cyclorrhapha; Schizophora; Acalyptratae; Ephydroidea; Drosophilidae; Drosophilinae; Colocasiomyini |
| *Drosophila sulfurigaster albostrigata* | 6 | 0 | 0 | Endopterygota; Diptera; Brachycera; Muscomorpha; Eremoneura; Cyclorrhapha; Schizophora; Acalyptratae; Ephydroidea; Drosophilidae; Drosophilinae; Drosophilini |
| *Drosophila albomicans* | 6 | 0 | 0 | Endopterygota; Diptera; Brachycera; Muscomorpha; Eremoneura; Cyclorrhapha; Schizophora; Acalyptratae; Ephydroidea; Drosophilidae; Drosophilinae; Drosophilini |
| *Drosophila nasuta* | 6 | 0 | 0 | Endopterygota; Diptera; Brachycera; Muscomorpha; Eremoneura; Cyclorrhapha; Schizophora; Acalyptratae; Ephydroidea; Drosophilidae; Drosophilinae; Drosophilini |
| *Drosophila grimshawi* | 5 | 0 | 2 | Endopterygota; Diptera; Brachycera; Muscomorpha; Eremoneura; Cyclorrhapha; Schizophora; Acalyptratae; Ephydroidea; Drosophilidae; Drosophilinae; Drosophilini |
| *Drosophila montana* | 7 | 1 | 2 | Endopterygota; Diptera; Brachycera; Muscomorpha; Eremoneura; Cyclorrhapha; Schizophora; Acalyptratae; Ephydroidea; Drosophilidae; Drosophilinae; Drosophilini |
| *Drosophila novamexicana* | 6 | 0 | 1 | Endopterygota; Diptera; Brachycera; Muscomorpha; Eremoneura; Cyclorrhapha; Schizophora; Acalyptratae; Ephydroidea; Drosophilidae; Drosophilinae; Drosophilini |
| *Drosophila virilis* | 6 | 0 | 1 | Endopterygota; Diptera; Brachycera; Muscomorpha; Eremoneura; Cyclorrhapha; Schizophora; Acalyptratae; Ephydroidea; Drosophilidae; Drosophilinae; Drosophilini |
| *Drosophila hydei* | 4 | 0 | 2 | Endopterygota; Diptera; Brachycera; Muscomorpha; Eremoneura; Cyclorrhapha; Schizophora; Acalyptratae; Ephydroidea; Drosophilidae; Drosophilinae; Drosophilini |
| *Drosophila navojoa* | 4 | 0 | 1 | Endopterygota; Diptera; Brachycera; Muscomorpha; Eremoneura; Cyclorrhapha; Schizophora; Acalyptratae; Ephydroidea; Drosophilidae; Drosophilinae; Drosophilini |
| *Drosophila arizonae* | 1 | 0 | 4 | Endopterygota; Diptera; Brachycera; Muscomorpha; Eremoneura; Cyclorrhapha; Schizophora; Acalyptratae; Ephydroidea; Drosophilidae; Drosophilinae; Drosophilini |
| *Drosophila mojavensis* | 5 | 0 | 0 | Endopterygota; Diptera; Brachycera; Muscomorpha; Eremoneura; Cyclorrhapha; Schizophora; Acalyptratae; Ephydroidea; Drosophilidae; Drosophilinae; Drosophilini |
| *Drosophila obscura* | 5 | 0 | 2 | Endopterygota; Diptera; Brachycera; Muscomorpha; Eremoneura; Cyclorrhapha; Schizophora; Acalyptratae; Ephydroidea; Drosophilidae; Drosophilinae; Drosophilini |
| *Drosophila guanche* | 6 | 0 | 0 | Endopterygota; Diptera; Brachycera; Muscomorpha; Eremoneura; Cyclorrhapha; Schizophora; Acalyptratae; Ephydroidea; Drosophilidae; Drosophilinae; Drosophilini |
| *Drosophila subobscura* | 5 | 0 | 1 | Endopterygota; Diptera; Brachycera; Muscomorpha; Eremoneura; Cyclorrhapha; Schizophora; Acalyptratae; Ephydroidea; Drosophilidae; Drosophilinae; Drosophilini |
| *Drosophila miranda* | 9 | 3 | 0 | Endopterygota; Diptera; Brachycera; Muscomorpha; Eremoneura; Cyclorrhapha; Schizophora; Acalyptratae; Ephydroidea; Drosophilidae; Drosophilinae; Drosophilini |
| *Drosophila persimilis* | 5 | 0 | 0 | Endopterygota; Diptera; Brachycera; Muscomorpha; Eremoneura; Cyclorrhapha; Schizophora; Acalyptratae; Ephydroidea; Drosophilidae; Drosophilinae; Drosophilini |
| *Drosophila pseudoobscura* | 5 | 0 | 0 | Endopterygota; Diptera; Brachycera; Muscomorpha; Eremoneura; Cyclorrhapha; Schizophora; Acalyptratae; Ephydroidea; Drosophilidae; Drosophilinae; Drosophilini |
| *Drosophila ananassae* | 5 | 0 | 0 | Endopterygota; Diptera; Brachycera; Muscomorpha; Eremoneura; Cyclorrhapha; Schizophora; Acalyptratae; Ephydroidea; Drosophilidae; Drosophilinae; Drosophilini |
| *Drosophila bipectinata* | 6 | 1 | 0 | Endopterygota; Diptera; Brachycera; Muscomorpha; Eremoneura; Cyclorrhapha; Schizophora; Acalyptratae; Ephydroidea; Drosophilidae; Drosophilinae; Drosophilini |
| *Drosophila kikkawai* | 5 | 1 | 2 | Endopterygota; Diptera; Brachycera; Muscomorpha; Eremoneura; Cyclorrhapha; Schizophora; Acalyptratae; Ephydroidea; Drosophilidae; Drosophilinae; Drosophilini |
| *Drosophila serrata* | 5 | 0 | 1 | Endopterygota; Diptera; Brachycera; Muscomorpha; Eremoneura; Cyclorrhapha; Schizophora; Acalyptratae; Ephydroidea; Drosophilidae; Drosophilinae; Drosophilini |
| *Drosophila ficusphila* | 4 | 0 | 6 | Endopterygota; Diptera; Brachycera; Muscomorpha; Eremoneura; Cyclorrhapha; Schizophora; Acalyptratae; Ephydroidea; Drosophilidae; Drosophilinae; Drosophilini |
| *Drosophila elegans* | 4 | 0 | 3 | Endopterygota; Diptera; Brachycera; Muscomorpha; Eremoneura; Cyclorrhapha; Schizophora; Acalyptratae; Ephydroidea; Drosophilidae; Drosophilinae; Drosophilini |
| *Drosophila rhopaloa* | 5 | 0 | 2 | Endopterygota; Diptera; Brachycera; Muscomorpha; Eremoneura; Cyclorrhapha; Schizophora; Acalyptratae; Ephydroidea; Drosophilidae; Drosophilinae; Drosophilini |
| *Drosophila takahashii* | 5 | 0 | 3 | Endopterygota; Diptera; Brachycera; Muscomorpha; Eremoneura; Cyclorrhapha; Schizophora; Acalyptratae; Ephydroidea; Drosophilidae; Drosophilinae; Drosophilini |
| *Drosophila biarmipes* | 5 | 0 | 2 | Endopterygota; Diptera; Brachycera; Muscomorpha; Eremoneura; Cyclorrhapha; Schizophora; Acalyptratae; Ephydroidea; Drosophilidae; Drosophilinae; Drosophilini |
| *Drosophila subpulchrella* | 4 | 0 | 1 | Endopterygota; Diptera; Brachycera; Muscomorpha; Eremoneura; Cyclorrhapha; Schizophora; Acalyptratae; Ephydroidea; Drosophilidae; Drosophilinae; Drosophilini |
| *Drosophila suzukii* | 4 | 0 | 1 | Endopterygota; Diptera; Brachycera; Muscomorpha; Eremoneura; Cyclorrhapha; Schizophora; Acalyptratae; Ephydroidea; Drosophilidae; Drosophilinae; Drosophilini |
| *Drosophila eugracilis* | 5 | 0 | 2 | Endopterygota; Diptera; Brachycera; Muscomorpha; Eremoneura; Cyclorrhapha; Schizophora; Acalyptratae; Ephydroidea; Drosophilidae; Drosophilinae; Drosophilini |
| *Drosophila melanogaster* | 5 | 0 | 1 | Endopterygota; Diptera; Brachycera; Muscomorpha; Eremoneura; Cyclorrhapha; Schizophora; Acalyptratae; Ephydroidea; Drosophilidae; Drosophilinae; Drosophilini |
| *Drosophila erecta* | 6 | 1 | 0 | Endopterygota; Diptera; Brachycera; Muscomorpha; Eremoneura; Cyclorrhapha; Schizophora; Acalyptratae; Ephydroidea; Drosophilidae; Drosophilinae; Drosophilini |
| *Drosophila teissieri* | 5 | 0 | 0 | Endopterygota; Diptera; Brachycera; Muscomorpha; Eremoneura; Cyclorrhapha; Schizophora; Acalyptratae; Ephydroidea; Drosophilidae; Drosophilinae; Drosophilini |
| *Drosophila yakuba* | 5 | 0 | 0 | Endopterygota; Diptera; Brachycera; Muscomorpha; Eremoneura; Cyclorrhapha; Schizophora; Acalyptratae; Ephydroidea; Drosophilidae; Drosophilinae; Drosophilini |
| *Drosophila sechellia* | 5 | 0 | 0 | Endopterygota; Diptera; Brachycera; Muscomorpha; Eremoneura; Cyclorrhapha; Schizophora; Acalyptratae; Ephydroidea; Drosophilidae; Drosophilinae; Drosophilini |
| *Drosophila mauritiana* | 5 | 0 | 0 | Endopterygota; Diptera; Brachycera; Muscomorpha; Eremoneura; Cyclorrhapha; Schizophora; Acalyptratae; Ephydroidea; Drosophilidae; Drosophilinae; Drosophilini |
| *Drosophila simulans* | 5 | 0 | 0 | Endopterygota; Diptera; Brachycera; Muscomorpha; Eremoneura; Cyclorrhapha; Schizophora; Acalyptratae; Ephydroidea; Drosophilidae; Drosophilinae; Drosophilini |

**Table S4 -** Speciation, duplication and loss events inferred by GeneRax for the I-type lysozyme gene family at internal nodes of the species tree.

| **Node** | **Copy number (speciations)** | **Duplications** | **Losses** |
| --- | --- | --- | --- |
| Node Abscondita terminalis Lamprigera yunnana | 6 | 1 | 1 |
| Node Achroia grisella Amyelois transitella | 4 | 0 | 3 |
| Node Achroia grisella Galleria mellonella | 3 | 0 | 1 |
| Node Acromyrmex echinatior Atta cephalotes | 3 | 0 | 0 |
| Node Acyrthosiphon pisum Melanaphis sacchari | 3 | 0 | 1 |
| Node Acyrthosiphon pisum Myzus persicae | 3 | 0 | 0 |
| Node Adelges cooleyi Daktulosphaira vitifoliae | 3 | 0 | 2 |
| Node Adelges cooleyi Sipha flava | 5 | 0 | 1 |
| Node Aedes aegypti Aedes albopictus | 2 | 0 | 0 |
| Node Aethina tumida Brassicogethes aeneus | 8 | 0 | 0 |
| Node Aethina tumida Cylas formicarius | 8 | 0 | 4 |
| Node Agrilus planipennis Ignelater luminosus | 6 | 0 | 8 |
| Node Agrilus planipennis Onthophagus taurus | 14 | 7 | 1 |
| Node Ampulex compressa Colletes gigas | 2 | 0 | 0 |
| Node Ampulex compressa Harpegnathos saltator | 2 | 0 | 0 |
| Node Amyelois transitella Plodia interpunctella | 3 | 0 | 1 |
| Node Anabrus simplex Bacillus rossius redtenbacheri | 8 | 0 | 3 |
| Node Anabrus simplex Frankliniella occidentalis | 11 | 2 | 0 |
| Node Anabrus simplex Gryllus longicercus | 8 | 2 | 2 |
| Node Anastrepha ludens Anastrepha obliqua | 2 | 0 | 0 |
| Node Anastrepha ludens Ceratitis capitata | 2 | 0 | 1 |
| Node Anastrepha ludens Glossina fuscipes | 3 | 0 | 1 |
| Node Anastrepha ludens Scaptodrosophila lebanonensis | 4 | 1 | 1 |
| Node Anopheles albimanus Anopheles aquasalis | 2 | 0 | 0 |
| Node Anopheles arabiensis Anopheles coluzzii | 2 | 0 | 0 |
| Node Anopheles bellator Anopheles cruzii | 2 | 0 | 0 |
| Node Anopheles bellator Anopheles darlingi | 2 | 0 | 1 |
| Node Anopheles bellator Anopheles maculipalpis | 3 | 0 | 0 |
| Node Anopheles coluzzii Anopheles gambiae | 2 | 0 | 0 |
| Node Anopheles coustani Anopheles bellator | 3 | 1 | 0 |
| Node Anopheles coustani Anopheles ziemanni | 2 | 0 | 1 |
| Node Anopheles darlingi Anopheles albimanus | 2 | 0 | 0 |
| Node Anopheles funestus Anopheles moucheti | 2 | 0 | 0 |
| Node Anopheles maculipalpis Anopheles nili | 2 | 0 | 1 |
| Node Anopheles maculipalpis Anopheles stephensi | 2 | 0 | 0 |
| Node Anopheles merus Anopheles arabiensis | 2 | 0 | 0 |
| Node Anopheles nili Anopheles funestus | 2 | 0 | 0 |
| Node Anopheles nili Anopheles merus | 2 | 0 | 0 |
| Node Anoplophora glabripennis Gonioctena quinquepunctata | 4 | 0 | 0 |
| Node Anthonomus grandis grandis Dendroctonus ponderosae | 1 | 0 | 1 |
| Node Anthonomus grandis grandis Rhynchophorus ferrugineus | 2 | 0 | 0 |
| Node Aphidius gifuensis Chelonus insularis | 3 | 0 | 0 |
| Node Aphidius gifuensis Fopius arisanus | 2 | 0 | 1 |
| Node Aphis craccivora Aphis gossypii | 3 | 0 | 0 |
| Node Aphis craccivora Rhopalosiphum maidis | 3 | 0 | 0 |
| Node Apis cerana Apis dorsata | 1 | 0 | 0 |
| Node Apis cerana Apis mellifera | 1 | 0 | 0 |
| Node Apis dorsata Apis laboriosa | 1 | 0 | 0 |
| Node Apis florea Apis cerana | 1 | 0 | 0 |
| Node Apolygus lucorum Cimex lectularius | 3 | 0 | 0 |
| Node Arctia plantaginis Trichoplusia ni | 6 | 0 | 1 |
| Node Aricia agestis Danaus plexippus | 7 | 0 | 1 |
| Node Armigeres subalbatus Aedes aegypti | 2 | 0 | 0 |
| Node Athalia rosae Cephus cinctus | 3 | 0 | 9 |
| Node Athalia rosae Chrysoperla carnea | 12 | 0 | 2 |
| Node Athalia rosae Diprion similis | 1 | 0 | 2 |
| Node Atta cephalotes Atta colombica | 3 | 0 | 0 |
| Node Bacillus rossius redtenbacheri Blattella germanica | 7 | 1 | 2 |
| Node Bactrocera dorsalis Bactrocera latifrons | 2 | 0 | 0 |
| Node Bactrocera tryoni Bactrocera dorsalis | 2 | 0 | 0 |
| Node Belonocnema kinseyi Leptopilina heterotoma | 2 | 0 | 1 |
| Node Belonocnema kinseyi Polistes dominula | 4 | 0 | 0 |
| Node Belonocnema kinseyi Trichogramma pretiosum | 3 | 0 | 1 |
| Node Belonocnema kinseyi Venturia canescens | 4 | 0 | 0 |
| Node Bemisia tabaci Planococcus citri | 6 | 0 | 3 |
| Node Bicyclus anynana Brenthis ino | 6 | 0 | 1 |
| Node Bicyclus anynana Maniola hyperantus | 4 | 0 | 2 |
| Node Bicyclus anynana Pararge aegeria | 3 | 0 | 1 |
| Node Blattella germanica Periplaneta americana | 7 | 1 | 1 |
| Node Bombus affinis Apis florea | 1 | 0 | 0 |
| Node Bombus affinis Bombus terrestris | 1 | 0 | 0 |
| Node Bombus affinis Frieseomelitta varia | 1 | 0 | 0 |
| Node Bombyx mandarina Bombyx mori | 3 | 0 | 2 |
| Node Bradysia coprophila Contarinia nasturtii | 3 | 0 | 4 |
| Node Bradysia coprophila Hermetia illucens | 7 | 0 | 1 |
| Node Brenthis ino Melitaea cinxia | 5 | 0 | 1 |
| Node Calliphora vicina Lucilia cuprina | 2 | 0 | 0 |
| Node Calliphora vicina Stomoxys calcitrans | 2 | 0 | 1 |
| Node Camponotus floridanus Cataglyphis hispanica | 4 | 0 | 0 |
| Node Cataglyphis hispanica Formica exsecta | 3 | 0 | 1 |
| Node Cephus cinctus Orussus abietinus | 3 | 0 | 0 |
| Node Ceratina calcarata Eufriesea mexicana | 1 | 0 | 0 |
| Node Ceratitis capitata Zeugodacus cucurbitae | 2 | 0 | 0 |
| Node Ceratosolen solmsi marchali Nasonia vitripennis | 1 | 0 | 0 |
| Node Chelonus insularis Cotesia glomerata | 1 | 0 | 2 |
| Node Chilo suppressalis Achroia grisella | 7 | 0 | 2 |
| Node Chilo suppressalis Manduca sexta | 9 | 1 | 2 |
| Node Chilo suppressalis Ostrinia furnacalis | 7 | 0 | 0 |
| Node Chilo suppressalis Parnassius apollo | 10 | 0 | 0 |
| Node Chrysoperla carnea Agrilus planipennis | 8 | 0 | 5 |
| Node Chrysoperla carnea Plutella xylostella | 13 | 1 | 0 |
| Node Cloeon dipterum Anabrus simplex | 9 | 3 | 0 |
| Node Cloeon dipterum Hetaerina americana | 5 | 0 | 4 |
| Node Cloeon dipterum Neocloeon triangulifer | 3 | 0 | 2 |
| Node Clunio marinus Polypedilum vanderplanki | 2 | 0 | 2 |
| Node Coccinella septempunctata Aethina tumida | 12 | 0 | 3 |
| Node Coccinella septempunctata Harmonia axyridis | 6 | 0 | 6 |
| Node Colias croceus Pieris rapae | 4 | 0 | 0 |
| Node Colias croceus Zerene cesonia | 4 | 0 | 0 |
| Node Colletes gigas Dufourea novaeangliae | 1 | 0 | 0 |
| Node Colletes gigas Hylaeus anthracinus | 1 | 0 | 0 |
| Node Colletes gigas Megachile rotundata | 1 | 0 | 1 |
| Node Condylostylus longicornis Episyrphus balteatus | 5 | 0 | 0 |
| Node Contarinia nasturtii Sitodiplosis mosellana | 1 | 0 | 2 |
| Node Copidosoma floridanum Phymastichus coffea | 2 | 0 | 0 |
| Node Coptotermes formosanus Cryptotermes secundus | 5 | 0 | 1 |
| Node Cotesia glomerata Microplitis demolitor | 1 | 0 | 0 |
| Node Culex pipiens pallens Culex quinquefasciatus | 2 | 0 | 1 |
| Node Culex pipiens pallens Ochlerotatus camptorhynchus | 3 | 0 | 0 |
| Node Culicoides brevitarsis Clunio marinus | 4 | 0 | 1 |
| Node Culicoides brevitarsis Lutzomyia longipalpis | 10 | 0 | 1 |
| Node Culicoides brevitarsis Toxorhynchites rutilus septentrionalis | 5 | 0 | 5 |
| Node Cydia pomonella Cydia splendana | 3 | 0 | 0 |
| Node Cylas formicarius Anoplophora glabripennis | 4 | 0 | 4 |
| Node Cylas formicarius Anthonomus grandis grandis | 2 | 0 | 2 |
| Node Cyphomyrmex costatus Trachymyrmex septentrionalis | 4 | 0 | 1 |
| Node Dalotia coriaria Nicrophorus vespilloides | 3 | 0 | 3 |
| Node Danaus plexippus Bicyclus anynana | 7 | 0 | 0 |
| Node Diabrotica virgifera virgifera Diorhabda carinulata | 2 | 0 | 1 |
| Node Diachasma alloeum Diachasmimorpha longicaudata | 2 | 1 | 0 |
| Node Dinoponera quadriceps Odontomachus brunneus | 1 | 0 | 0 |
| Node Diorhabda carinulata Diorhabda sublineata | 2 | 0 | 0 |
| Node Diprion similis Neodiprion fabricii | 1 | 0 | 0 |
| Node Drosophila albomicans Drosophila nasuta | 6 | 0 | 0 |
| Node Drosophila ananassae Drosophila bipectinata | 5 | 0 | 7 |
| Node Drosophila ananassae Drosophila kikkawai | 12 | 0 | 0 |
| Node Drosophila arizonae Drosophila mojavensis | 5 | 0 | 0 |
| Node Drosophila biarmipes Drosophila subpulchrella | 7 | 0 | 1 |
| Node Drosophila elegans Drosophila rhopaloa | 7 | 0 | 2 |
| Node Drosophila elegans Drosophila takahashii | 9 | 1 | 2 |
| Node Drosophila erecta Drosophila sechellia | 6 | 0 | 0 |
| Node Drosophila erecta Drosophila teissieri | 5 | 0 | 1 |
| Node Drosophila eugracilis Drosophila melanogaster | 7 | 0 | 2 |
| Node Drosophila ficusphila Drosophila elegans | 10 | 0 | 1 |
| Node Drosophila grimshawi Drosophila montana | 7 | 0 | 1 |
| Node Drosophila guanche Drosophila subobscura | 6 | 0 | 1 |
| Node Drosophila hydei Drosophila navojoa | 6 | 0 | 1 |
| Node Drosophila kikkawai Drosophila ficusphila | 11 | 0 | 1 |
| Node Drosophila kikkawai Drosophila serrata | 6 | 0 | 5 |
| Node Drosophila mauritiana Drosophila simulans | 5 | 0 | 0 |
| Node Drosophila melanogaster Drosophila erecta | 6 | 1 | 2 |
| Node Drosophila miranda Drosophila persimilis | 6 | 0 | 2 |
| Node Drosophila montana Drosophila hydei | 7 | 1 | 1 |
| Node Drosophila montana Drosophila novamexicana | 8 | 1 | 0 |
| Node Drosophila navojoa Drosophila arizonae | 5 | 0 | 1 |
| Node Drosophila novamexicana Drosophila virilis | 7 | 0 | 1 |
| Node Drosophila obscura Drosophila ananassae | 12 | 4 | 2 |
| Node Drosophila obscura Drosophila guanche | 7 | 0 | 1 |
| Node Drosophila obscura Drosophila miranda | 8 | 1 | 5 |
| Node Drosophila persimilis Drosophila pseudoobscura | 5 | 0 | 1 |
| Node Drosophila sechellia Drosophila mauritiana | 5 | 0 | 1 |
| Node Drosophila subpulchrella Drosophila suzukii | 5 | 0 | 2 |
| Node Drosophila sulfurigaster albostrigata Drosophila albomicans | 6 | 0 | 2 |
| Node Drosophila sulfurigaster albostrigata Drosophila grimshawi | 8 | 0 | 2 |
| Node Drosophila sulfurigaster albostrigata Drosophila obscura | 10 | 4 | 0 |
| Node Drosophila takahashii Drosophila biarmipes | 8 | 0 | 1 |
| Node Drosophila takahashii Drosophila eugracilis | 9 | 1 | 1 |
| Node Drosophila teissieri Drosophila yakuba | 5 | 0 | 0 |
| Node Dufourea novaeangliae Megalopta genalis | 1 | 0 | 0 |
| Node Eciton burchellii Linepithema humile | 3 | 1 | 0 |
| Node Eciton burchellii Ooceraea biroi | 2 | 0 | 1 |
| Node Episyrphus balteatus Anastrepha ludens | 4 | 0 | 1 |
| Node Episyrphus balteatus Eupeodes corollae | 4 | 1 | 1 |
| Node Eriocheir sinensis Scylla paramamosain | 2 | 1 | 2 |
| Node Eufriesea mexicana Bombus affinis | 1 | 0 | 0 |
| Node Folsomia candida Cloeon dipterum | 6 | 1 | 0 |
| Node Fopius arisanus Diachasma alloeum | 1 | 0 | 1 |
| Node Frankliniella occidentalis Homalodisca vitripennis | 12 | 0 | 2 |
| Node Frankliniella occidentalis Pediculus humanus corporis | 14 | 3 | 0 |
| Node Frankliniella occidentalis Thrips palmi | 5 | 1 | 8 |
| Node Frieseomelitta varia Melipona quadrifasciata | 1 | 0 | 0 |
| Node Glossina fuscipes Calliphora vicina | 3 | 0 | 0 |
| Node Gonioctena quinquepunctata Diabrotica virgifera virgifera | 3 | 0 | 1 |
| Node Gonioctena quinquepunctata Leptinotarsa decemlineata | 2 | 0 | 1 |
| Node Halyomorpha halys Rhodnius prolixus | 3 | 0 | 6 |
| Node Harpegnathos saltator Dinoponera quadriceps | 1 | 0 | 1 |
| Node Harpegnathos saltator Eciton burchellii | 2 | 1 | 1 |
| Node Helicoverpa armigera Helicoverpa zea | 3 | 0 | 0 |
| Node Helicoverpa armigera Spodoptera frugiperda | 3 | 0 | 0 |
| Node Hermetia illucens Condylostylus longicornis | 5 | 0 | 2 |
| Node Hetaerina americana Ischnura elegans | 2 | 0 | 3 |
| Node Homalodisca vitripennis Bemisia tabaci | 9 | 0 | 3 |
| Node Homalodisca vitripennis Halyomorpha halys | 9 | 0 | 0 |
| Node Homalodisca vitripennis Laodelphax striatellus | 8 | 0 | 1 |
| Node Hylaeus anthracinus Hylaeus volcanicus | 1 | 0 | 0 |
| Node Hyposmocoma kahamanoa Chilo suppressalis | 10 | 4 | 1 |
| Node Hyposmocoma kahamanoa Pectinophora gossypiella | 4 | 0 | 6 |
| Node Ignelater luminosus Abscondita terminalis | 6 | 0 | 0 |
| Node Lamprigera yunnana Photinus pyralis | 7 | 1 | 0 |
| Node Laodelphax striatellus Nilaparvata lugens | 8 | 2 | 2 |
| Node Leguminivora glycinivorella Cydia pomonella | 3 | 0 | 4 |
| Node Leguminivora glycinivorella Hyposmocoma kahamanoa | 7 | 3 | 0 |
| Node Leptidea sinapis Aricia agestis | 8 | 0 | 1 |
| Node Leptidea sinapis Colias croceus | 4 | 0 | 4 |
| Node Leptopilina heterotoma Leptopilina boulardi | 1 | 0 | 1 |
| Node Linepithema humile Nylanderia fulva | 4 | 1 | 0 |
| Node Linepithema humile Pseudomyrmex gracilis | 2 | 0 | 2 |
| Node Lucilia cuprina Lucilia sericata | 2 | 0 | 0 |
| Node Lutzomyia longipalpis Bradysia coprophila | 8 | 0 | 2 |
| Node Lutzomyia longipalpis Phlebotomus argentipes | 1 | 0 | 7 |
| Node Malaya genurostris Culex pipiens pallens | 3 | 0 | 0 |
| Node Malaya genurostris Sabethes cyaneus | 3 | 0 | 0 |
| Node Malaya genurostris Topomyia yanbarensis | 2 | 0 | 1 |
| Node Manduca sexta Arctia plantaginis | 7 | 0 | 2 |
| Node Manduca sexta Bombyx mandarina | 5 | 0 | 2 |
| Node Maniola hyperantus Maniola jurtina | 4 | 0 | 0 |
| Node Megachile rotundata Ceratina calcarata | 1 | 0 | 0 |
| Node Megachile rotundata Osmia bicornis bicornis | 1 | 0 | 0 |
| Node Megalopta genalis Nomia melanderi | 1 | 0 | 0 |
| Node Melanaphis sacchari Aphis craccivora | 3 | 0 | 0 |
| Node Melitaea cinxia Nymphalis io | 4 | 0 | 1 |
| Node Microplitis demolitor Microplitis mediator | 1 | 0 | 0 |
| Node Monomorium pharaonis Solenopsis invicta | 3 | 0 | 2 |
| Node Monomorium pharaonis Wasmannia auropunctata | 5 | 1 | 1 |
| Node Musca domestica Musca vetustissima | 3 | 0 | 0 |
| Node Neodiprion fabricii Neodiprion lecontei | 1 | 0 | 0 |
| Node Neodiprion fabricii Neodiprion virginianus | 1 | 0 | 0 |
| Node Neodiprion lecontei Neodiprion pinetum | 1 | 0 | 0 |
| Node Nylanderia fulva Camponotus floridanus | 4 | 1 | 1 |
| Node Nylanderia fulva Pogonomyrmex barbatus | 4 | 0 | 0 |
| Node Nymphalis io Vanessa cardui | 4 | 0 | 0 |
| Node Ochlerotatus camptorhynchus Armigeres subalbatus | 2 | 0 | 1 |
| Node Onthophagus taurus Dalotia coriaria | 6 | 0 | 9 |
| Node Onthophagus taurus Tenebrio molitor | 15 | 1 | 0 |
| Node Orussus abietinus Belonocnema kinseyi | 4 | 1 | 0 |
| Node Osmia bicornis bicornis Osmia lignaria | 1 | 0 | 0 |
| Node Ostrinia furnacalis Ostrinia nubilalis | 4 | 0 | 3 |
| Node Papilio machaon Papilio xuthus | 4 | 0 | 0 |
| Node Papilio polytes Papilio machaon | 4 | 0 | 1 |
| Node Parnassius apollo Leptidea sinapis | 9 | 2 | 3 |
| Node Parnassius apollo Papilio polytes | 5 | 0 | 4 |
| Node Pediculus humanus corporis Athalia rosae | 14 | 0 | 0 |
| Node Periplaneta americana Zootermopsis nevadensis | 7 | 0 | 0 |
| Node Phlebotomus argentipes Phlebotomus papatasi | 1 | 0 | 0 |
| Node Phymastichus coffea Ceratosolen solmsi marchali | 1 | 0 | 1 |
| Node Pieris brassicae Pieris napi | 4 | 0 | 0 |
| Node Pieris rapae Pieris brassicae | 4 | 1 | 1 |
| Node Planococcus citri Adelges cooleyi | 6 | 0 | 0 |
| Node Plutella xylostella Culicoides brevitarsis | 11 | 0 | 2 |
| Node Plutella xylostella Leguminivora glycinivorella | 4 | 1 | 8 |
| Node Pogonomyrmex barbatus Vollenhovia emeryi | 3 | 0 | 1 |
| Node Polistes canadensis Polistes fuscatus | 1 | 0 | 0 |
| Node Polistes dominula Ampulex compressa | 2 | 1 | 3 |
| Node Polistes dominula Polistes canadensis | 1 | 0 | 0 |
| Node Polistes dominula Vespula pensylvanica | 1 | 0 | 1 |
| Node Procambarus clarkii Eriocheir sinensis | 3 | 0 | 2 |
| Node Procambarus clarkii Folsomia candida | 5 | 4 | 0 |
| Node Rhodnius prolixus Apolygus lucorum | 3 | 0 | 0 |
| Node Rhopalosiphum maidis Rhopalosiphum padi | 3 | 0 | 0 |
| Node Rhynchophorus ferrugineus Sitophilus oryzae | 2 | 0 | 0 |
| Node Sabethes cyaneus Wyeomyia smithii | 2 | 0 | 1 |
| Node Scaptodrosophila lebanonensis Drosophila sulfurigaster albostrigata | 6 | 2 | 0 |
| Node Sipha flava Acyrthosiphon pisum | 4 | 0 | 1 |
| Node Spodoptera frugiperda Spodoptera litura | 3 | 0 | 0 |
| Node Stomoxys calcitrans Musca domestica | 3 | 1 | 0 |
| Node Temnothorax curvispinosus Temnothorax longispinosus | 3 | 0 | 2 |
| Node Tenebrio molitor Coccinella septempunctata | 15 | 1 | 1 |
| Node Tenebrio molitor Tribolium castaneum | 8 | 0 | 7 |
| Node Tenebrio molitor Zophobas morio | 8 | 0 | 0 |
| Node Toxorhynchites rutilus septentrionalis Anopheles coustani | 2 | 0 | 3 |
| Node Toxorhynchites rutilus septentrionalis Uranotaenia lowii | 2 | 0 | 0 |
| Node Trachymyrmex septentrionalis Acromyrmex echinatior | 3 | 0 | 1 |
| Node Tribolium castaneum Tribolium madens | 3 | 0 | 5 |
| Node Trichogramma pretiosum Copidosoma floridanum | 2 | 0 | 1 |
| Node Trichoplusia ni Helicoverpa armigera | 3 | 0 | 3 |
| Node Uranotaenia lowii Malaya genurostris | 3 | 1 | 0 |
| Node Vanessa atalanta Vanessa tameamea | 3 | 0 | 0 |
| Node Vanessa cardui Vanessa atalanta | 3 | 0 | 1 |
| Node Venturia canescens Aphidius gifuensis | 3 | 0 | 1 |
| Node Vespa crabro Vespa mandarinia | 1 | 0 | 0 |
| Node Vespa velutina Vespa crabro | 1 | 0 | 0 |
| Node Vespula pensylvanica Vespa velutina | 1 | 0 | 0 |
| Node Vespula pensylvanica Vespula vulgaris | 1 | 0 | 0 |
| Node Vollenhovia emeryi Monomorium pharaonis | 5 | 2 | 0 |
| Node Vollenhovia emeryi Temnothorax curvispinosus | 5 | 0 | 0 |
| Node Wasmannia auropunctata Cyphomyrmex costatus | 5 | 0 | 0 |
| Node Zeugodacus cucurbitae Bactrocera tryoni | 2 | 0 | 0 |
| Node Zootermopsis nevadensis Coptotermes formosanus | 6 | 0 | 1 |

**Table S5** - Docking results obtained with HADDOCK, showing electrostatic and van der Waals interaction energies, buried surface area, restraint violations, cluster assignment, and the resulting HADDOCK score for each model species.

| **Species** | **Protein sequence accession ID** | **Electrostatic Energy** | **Van der Waals Energy** | **Buried Surface Area** | **Restraints Violation** | **Cluster** | **HADDOCK Score** |
| --- | --- | --- | --- | --- | --- | --- | --- |
| *Meretrix lusoria* | 3AB6.1/P86383.1 | -112,5 | -46,3 | 862,7 | 0 | 1 | -68,3 |
| *Periplaneta americana* | XP_069684582.1 | -122,1 | -37,9 | 814,9 | 14,5 | 2 | -57,2 |
| *Rhodnius prolixus* | RPRC011594-PA | -144 | -31,8 | 764,6 | 4 | 2 | -57,5 |
| *Tenebrio molitor* | Tmol_Bit_LysI_319 | -114 | -34,8 | 771,9 | 0,1 | 3 | -58,1 |
| *Spodoptera frugiperda* | XP_035444103.1 | -119,9 | -39,8 | 774 | 14,2 | 1 | -63,1 |
| *Musca domestica* | XP_019892932.2 | -147,8 | -37,4 | 806,2 | 7,5 | 2 | -63,6 |

**Table S6** - Results predicted by AMP scanner for each sequence C-type lysozyme copies.

| Species | Sequence accession number | Prediction_Class | Prediction_Probability |
| --- | --- | --- | --- |
| *Abscondita terminalis* | Ater_Bit_LysC_2 | AMP | 1 |
| *Achroia grisella* | XP_059055429.1 | AMP | 0.9999 |
| *Achroia grisella* | XP_059055439.1 | AMP | 0.9995 |
| *Achroia grisella* | Agri_Bit_LysC_5 | AMP | 0.5821 |
| *Aedes aegypti* | XP_021699294.1 | AMP | 1 |
| *Aedes aegypti* | XP_021699295.1 | AMP | 1 |
| *Aedes aegypti* | Aaeg_Bit_LysC_7 | AMP | 0.9877 |
| *Aedes albopictus* | XP_019560054.3 | AMP | 0.9999 |
| *Aedes albopictus* | XP_019560056.3 | AMP | 1 |
| *Aedes albopictus* | XP_019562191.1 | AMP | 1 |
| *Aedes albopictus* | XP_029735634.2 | AMP | 1 |
| *Aethina tumida* | XP_019867332.1 | AMP | 0.9919 |
| *Aethina tumida* | XP_019872118.1 | AMP | 0.9999 |
| *Aethina tumida* | Atum_Bit_LysC_14 | AMP | 0.9996 |
| *Aethina tumida* | Atum_Bit_LysC_15 | AMP | 0.9972 |
| *Aethina tumida* | Atum_Bit_LysC_16 | Non-AMP | 0.1859 |
| *Aethina tumida* | Atum_Bit_LysC_17 | AMP | 0.9996 |
| *Agrilus planipennis* | Apla_Bit_LysC_18 | AMP | 0.9943 |
| *Agrilus planipennis* | Apla_Bit_LysC_19 | AMP | 0.9913 |
| *Agrilus planipennis* | Apla_Bit_LysC_20 | AMP | 0.9958 |
| *Amyelois transitella* | XP_013196660.1 | AMP | 0.9997 |
| *Amyelois transitella* | Atra_Bit_LysC_21 | AMP | 0.9186 |
| *Anabrus simplex* | XP_066996509.2 | AMP | 0.9999 |
| *Anabrus simplex* | XP_067002033.2 | AMP | 1 |
| *Anabrus simplex* | XP_068083738.1 | AMP | 0.9995 |
| *Anastrepha ludens* | XP_053965636.1 | AMP | 0.9996 |
| *Anastrepha ludens* | XP_053965638.1 | AMP | 0.9992 |
| *Anastrepha ludens* | XP_053965639.1 | AMP | 0.9993 |
| *Anastrepha ludens* | XP_053966453.1 | AMP | 1 |
| *Anastrepha ludens* | XP_053966455.1 | AMP | 0.9862 |
| *Anastrepha ludens* | XP_053966456.1 | AMP | 0.9797 |
| *Anastrepha ludens* | XP_053966457.1 | AMP | 0.9862 |
| *Anastrepha ludens* | XP_053966458.1 | AMP | 0.9564 |
| *Anastrepha obliqua* | XP_054737972.1 | AMP | 0.9968 |
| *Anastrepha obliqua* | XP_054740623.1 | AMP | 0.9982 |
| *Anastrepha obliqua* | XP_054741316.1 | AMP | 0.9992 |
| *Anastrepha obliqua* | XP_054741317.1 | AMP | 0.9999 |
| *Anastrepha obliqua* | XP_054741318.1 | AMP | 0.9703 |
| *Anastrepha obliqua* | XP_054741319.1 | AMP | 0.9969 |
| *Anastrepha obliqua* | Aobl_Bit_LysC_25 | AMP | 0.9992 |
| *Anastrepha obliqua* | Aobl_Bit_LysC_26 | AMP | 0.999 |
| *Anopheles albimanus* | XP_035791660.1 | AMP | 1 |
| *Anopheles aquasalis* | XP_050095280.1 | AMP | 0.9999 |
| *Anopheles arabiensis* | XP_040160486.1 | AMP | 0.987 |
| *Anopheles arabiensis* | XP_040160488.1 | AMP | 1 |
| *Anopheles arabiensis* | XP_040160490.1 | AMP | 0.9996 |
| *Anopheles bellator* | XP_058053456.1 | AMP | 1 |
| *Anopheles bellator* | XP_058064248.1 | AMP | 0.9994 |
| *Anopheles coluzzii* | XP_040219419.2 | AMP | 0.9996 |
| *Anopheles coluzzii* | XP_040219422.2 | AMP | 1 |
| *Anopheles coluzzii* | XP_049461420.1 | AMP | 0.9892 |
| *Anopheles coustani* | XP_058124699.1 | AMP | 0.9998 |
| *Anopheles coustani* | XP_058124700.1 | AMP | 1 |
| *Anopheles cruzii* | XP_052860690.1 | AMP | 1 |
| *Anopheles cruzii* | XP_052872831.1 | AMP | 0.9999 |
| *Anopheles darlingi* | XP_049541918.1 | AMP | 0.9999 |
| *Anopheles darlingi* | XP_049541919.1 | AMP | 0.9925 |
| *Anopheles funestus* | XP_049297859.1 | AMP | 1 |
| *Anopheles gambiae* | XP_001237086.2 | AMP | 0.9955 |
| *Anopheles gambiae* | XP_061496669.1 | AMP | 0.9993 |
| *Anopheles gambiae* | XP_308481.1 | AMP | 1 |
| *Anopheles gambiae* | XP_308483.3 | AMP | 0.9942 |
| *Anopheles gambiae* | Agam_Bit_LysC_47 | AMP | 0.9992 |
| *Anopheles maculipalpis* | XP_050075833.1 | AMP | 1 |
| *Anopheles maculipalpis* | XP_050075897.1 | AMP | 0.9988 |
| *Anopheles maculipalpis* | Amac_Bit_LysC_50 | AMP | 0.7192 |
| *Anopheles merus* | XP_041773892.1 | AMP | 0.9979 |
| *Anopheles merus* | XP_041773894.1 | AMP | 1 |
| *Anopheles moucheti* | XP_052899308.1 | AMP | 0.9999 |
| *Anopheles moucheti* | XP_052899309.1 | AMP | 0.9999 |
| *Anopheles nili* | XP_053674624.1 | AMP | 0.9999 |
| *Anopheles nili* | XP_053675128.1 | AMP | 1 |
| *Anopheles nili* | Anil_Bit_LysC_60 | AMP | 0.9999 |
| *Anopheles stephensi* | XP_035908481.1 | AMP | 0.8545 |
| *Anopheles stephensi* | XP_035908483.1 | AMP | 0.9999 |
| *Anopheles ziemanni* | XP_058176132.1 | AMP | 1 |
| *Anopheles ziemanni* | XP_058176143.1 | AMP | 0.9998 |
| *Anoplophora glabripennis* | XP_018562961.1 | AMP | 0.9989 |
| *Anoplophora glabripennis* | XP_018566901.1 | AMP | 0.9991 |
| *Anoplophora glabripennis* | XP_018566916.1 | AMP | 0.997 |
| *Anoplophora glabripennis* | Agla_Bit_LysC_65 | AMP | 0.9983 |
| *Anoplophora glabripennis* | Agla_Bit_LysC_66 | AMP | 0.9861 |
| *Anoplophora glabripennis* | Agla_Bit_LysC_67 | AMP | 0.9999 |
| *Anthonomus grandis grandis* | XP_050299141.1 | AMP | 0.9729 |
| *Anthonomus grandis grandis* | XP_050304684.1 | AMP | 0.9998 |
| *Anthonomus grandis grandis* | Agra_Bit_LysC_70 | AMP | 0.5136 |
| *Apolygus lucorum* | KAF6201276.1 | AMP | 0.9506 |
| *Apolygus lucorum* | Aluc_Bit_LysC_72 | AMP | 0.9997 |
| *Arctia plantaginis* | CAB3222611.1 | AMP | 1 |
| *Arctia plantaginis* | CAB3235271.1 | AMP | 1 |
| *Arctia plantaginis* | CAB3235273.1 | AMP | 0.9994 |
| *Arctia plantaginis* | CAB3250741.1 | AMP | 0.9993 |
| *Arctia plantaginis* | CAB3250742.1 | AMP | 0.9958 |
| *Arctia plantaginis* | CAB3250743.1 | AMP | 0.9985 |
| *Arctia plantaginis* | CAB3250744.1 | AMP | 0.9941 |
| *Aricia agestis* | XP_041988124.1 | AMP | 0.9998 |
| *Aricia agestis* | XP_041988125.1 | AMP | 1 |
| *Armigeres subalbatus* | XP_062534050.1 | AMP | 1 |
| *Armigeres subalbatus* | XP_062534056.1 | AMP | 1 |
| *Armigeres subalbatus* | XP_062550871.1 | AMP | 1 |
| *Armigeres subalbatus* | XP_062558375.1 | AMP | 1 |
| *Athalia rosae* | XP_012258668.2 | AMP | 0.9922 |
| *Athalia rosae* | XP_012261627.2 | AMP | 1 |
| *Athalia rosae* | XP_012267191.2 | AMP | 0.9998 |
| *Bacillus rossius redtenbacheri* | XP_063236222.1 | AMP | 0.9997 |
| *Bacillus rossius redtenbacheri* | XP_063239276.1 | AMP | 1 |
| *Bacillus rossius redtenbacheri* | XP_063242984.1 | AMP | 0.9393 |
| *Bactrocera dorsalis* | XP_011199990.2 | AMP | 0.9996 |
| *Bactrocera dorsalis* | XP_011199991.2 | AMP | 0.9801 |
| *Bactrocera dorsalis* | XP_011206449.2 | AMP | 1 |
| *Bactrocera dorsalis* | XP_011206450.2 | AMP | 1 |
| *Bactrocera dorsalis* | XP_011206451.1 | AMP | 0.99 |
| *Bactrocera dorsalis* | XP_011206452.1 | AMP | 0.9609 |
| *Bactrocera dorsalis* | XP_011206453.2 | AMP | 0.9609 |
| *Bactrocera dorsalis* | XP_011206454.1 | AMP | 0.9947 |
| *Bactrocera dorsalis* | XP_011206455.2 | AMP | 0.9289 |
| *Bactrocera dorsalis* | XP_011206456.2 | AMP | 0.9971 |
| *Bactrocera dorsalis* | XP_049307629.1 | AMP | 0.9994 |
| *Bactrocera dorsalis* | XP_049309706.1 | AMP | 0.9871 |
| *Bactrocera latifrons* | XP_018784449.1 | AMP | 0.9975 |
| *Bactrocera latifrons* | XP_018784507.1 | AMP | 0.9943 |
| *Bactrocera latifrons* | XP_018784508.1 | AMP | 0.9975 |
| *Bactrocera latifrons* | XP_018784510.1 | AMP | 0.9999 |
| *Bactrocera latifrons* | XP_018786149.1 | AMP | 0.9993 |
| *Bactrocera latifrons* | Blat_Bit_LysC_86 | AMP | 0.9996 |
| *Bactrocera latifrons* | Blat_Bit_LysC_89 | AMP | 0.9954 |
| *Bactrocera tryoni* | XP_039954296.1 | AMP | 0.9996 |
| *Bactrocera tryoni* | XP_039954297.1 | AMP | 0.9993 |
| *Bactrocera tryoni* | XP_039956973.1 | AMP | 0.9985 |
| *Bactrocera tryoni* | XP_039957069.1 | AMP | 1 |
| *Bactrocera tryoni* | XP_039957071.1 | AMP | 0.9982 |
| *Bactrocera tryoni* | XP_039957072.1 | AMP | 1 |
| *Bactrocera tryoni* | XP_039957073.1 | AMP | 0.9635 |
| *Bactrocera tryoni* | XP_039957074.1 | AMP | 0.9321 |
| *Bactrocera tryoni* | Btry_Bit_LysC_92 | AMP | 0.9442 |
| *Bactrocera tryoni* | Btry_Bit_LysC_93 | AMP | 0.9963 |
| *Bactrocera tryoni* | Btry_Bit_LysC_94 | AMP | 0.9635 |
| *Bactrocera tryoni* | Btry_Bit_LysC_97 | AMP | 0.9943 |
| *Bemisia tabaci* | XP_018914386.1 | AMP | 0.9849 |
| *Bemisia tabaci* | XP_018914387.1 | AMP | 0.9997 |
| *Bicyclus anynana* | XP_023955128.1 | AMP | 0.9999 |
| *Bicyclus anynana* | Bany_Bit_LysC_98 | AMP | 0.9699 |
| *Blattella germanica* | PSN37595.1 | AMP | 1 |
| *Blattella germanica* | PSN47214.1 | AMP | 0.8061 |
| *Blattella germanica* | PSN47217.1 | AMP | 0.9955 |
| *Bombyx mandarina* | XP_028030251.1 | AMP | 1 |
| *Bombyx mori* | NP_001037448.1 | AMP | 1 |
| *Bombyx mori* | Bmor_Bit_LysC_100 | AMP | 0.758 |
| *Brassicogethes aeneus* | CAH0546601.1 | AMP | 0.9999 |
| *Brassicogethes aeneus* | CAH0546602.1 | AMP | 1 |
| *Brassicogethes aeneus* | Baen_Bit_LysC_104 | AMP | 0.9978 |
| *Brenthis ino* | CAH0714697.1 | AMP | 0.9996 |
| *Brenthis ino* | Bino_Bit_LysC_106 | AMP | 0.9922 |
| *Calliphora vicina* | XP_065355865.1 | AMP | 0.9998 |
| *Calliphora vicina* | XP_065355948.1 | AMP | 0.9998 |
| *Calliphora vicina* | XP_065355949.1 | AMP | 0.9998 |
| *Calliphora vicina* | XP_065356564.1 | AMP | 0.9998 |
| *Calliphora vicina* | XP_065356598.1 | AMP | 0.9998 |
| *Calliphora vicina* | XP_065356694.1 | AMP | 0.9998 |
| *Calliphora vicina* | XP_065356764.1 | AMP | 0.9998 |
| *Calliphora vicina* | XP_065356770.1 | AMP | 0.9983 |
| *Calliphora vicina* | XP_065367382.1 | AMP | 0.9997 |
| *Calliphora vicina* | XP_065367456.1 | AMP | 0.9994 |
| *Calliphora vicina* | XP_065367470.1 | AMP | 0.999 |
| *Calliphora vicina* | XP_065367589.1 | AMP | 0.9999 |
| *Calliphora vicina* | XP_065367603.1 | AMP | 0.9999 |
| *Calliphora vicina* | XP_065367717.1 | AMP | 0.9998 |
| *Calliphora vicina* | XP_065368094.1 | AMP | 1 |
| *Calliphora vicina* | XP_065368258.1 | AMP | 1 |
| *Cephus cinctus* | XP_015597738.1 | AMP | 0.9084 |
| *Ceratitis capitata* | XP_004517799.1 | AMP | 0.6409 |
| *Ceratitis capitata* | XP_004517800.1 | AMP | 0.6409 |
| *Ceratitis capitata* | XP_004522949.1 | AMP | 0.9976 |
| *Ceratitis capitata* | XP_004522950.1 | AMP | 0.9998 |
| *Ceratitis capitata* | XP_012155684.1 | AMP | 1 |
| *Ceratitis capitata* | XP_020713530.1 | AMP | 0.998 |
| *Ceratitis capitata* | XP_020717732.1 | Non-AMP | 0.4054 |
| *Ceratitis capitata* | XP_023158883.1 | AMP | 0.6409 |
| *Chelonus insularis* | XP_034937417.1 | AMP | 0.9819 |
| *Chilo suppressalis* | RVE48686.1 | AMP | 0.9999 |
| *Chilo suppressalis* | RVE49532.1 | AMP | 1 |
| *Chilo suppressalis* | RVE51865.1 | AMP | 0.9994 |
| *Chilo suppressalis* | RVE51866.1 | AMP | 1 |
| *Chilo suppressalis* | RVE51867.1 | AMP | 0.9984 |
| *Chilo suppressalis* | Csup_Bit_LysC_118 | AMP | 0.9999 |
| *Chilo suppressalis* | Csup_Bit_LysC_119 | Non-AMP | 0.1245 |
| *Chrysoperla carnea* | XP_044726453.1 | AMP | 0.9992 |
| *Chrysoperla carnea* | XP_044726455.1 | AMP | 0.9998 |
| *Chrysoperla carnea* | XP_044726460.1 | AMP | 0.9999 |
| *Chrysoperla carnea* | XP_044729787.1 | AMP | 0.9999 |
| *Chrysoperla carnea* | XP_044730425.1 | AMP | 0.9974 |
| *Chrysoperla carnea* | XP_044732070.1 | AMP | 1 |
| *Chrysoperla carnea* | XP_044743167.1 | AMP | 0.9859 |
| *Chrysoperla carnea* | Ccar_Bit_LysC_120 | AMP | 0.9544 |
| *Chrysoperla carnea* | Ccar_Bit_LysC_123 | AMP | 0.7551 |
| *Cimex lectularius* | XP_014239425.1 | AMP | 0.9975 |
| *Cimex lectularius* | XP_014244232.1 | AMP | 0.9988 |
| *Cloeon dipterum* | XP_065336862.1 | AMP | 0.9885 |
| *Clunio marinus* | CRL05171.1 | AMP | 1 |
| *Coccinella septempunctata* | XP_044749297.1 | AMP | 0.9967 |
| *Coccinella septempunctata* | XP_044753475.1 | AMP | 0.8705 |
| *Coccinella septempunctata* | XP_044754217.1 | AMP | 1 |
| *Coccinella septempunctata* | XP_044757049.1 | AMP | 0.9901 |
| *Coccinella septempunctata* | Csep_Bit_LysC_128 | AMP | 0.9119 |
| *Colias croceus* | XP_045495178.1 | AMP | 1 |
| *Colias croceus* | XP_045495179.1 | AMP | 0.9928 |
| *Colias croceus* | Ccro_Bit_LysC_130 | AMP | 0.9983 |
| *Condylostylus longicornis* | XP_055374658.1 | AMP | 0.9999 |
| *Condylostylus longicornis* | XP_055380046.1 | AMP | 0.9989 |
| *Condylostylus longicornis* | XP_055380222.1 | AMP | 0.9946 |
| *Coptotermes formosanus* | GFG32422.1 | AMP | 0.9931 |
| *Coptotermes formosanus* | GFG39621.1 | AMP | 0.9994 |
| *Coptotermes formosanus* | Cfor_Bit_LysC_136 | AMP | 0.9987 |
| *Cryptotermes secundus* | XP_023707583.1 | AMP | 1 |
| *Cryptotermes secundus* | XP_023708064.1 | AMP | 0.9178 |
| *Cryptotermes secundus* | XP_023708067.1 | AMP | 0.9999 |
| *Cryptotermes secundus* | XP_023708070.1 | AMP | 0.9951 |
| *Cryptotermes secundus* | XP_023708072.1 | AMP | 1 |
| *Cryptotermes secundus* | XP_023708073.1 | AMP | 1 |
| *Cryptotermes secundus* | XP_023711547.1 | AMP | 0.9992 |
| *Cryptotermes secundus* | XP_023720269.1 | AMP | 0.9976 |
| *Culex pipiens pallens* | XP_039437504.1 | AMP | 1 |
| *Culex quinquefasciatus* | XP_001847112.1 | AMP | 1 |
| *Cydia pomonella* | XP_061729742.1 | AMP | 0.9999 |
| *Cydia pomonella* | XP_061729744.1 | AMP | 0.9998 |
| *Cydia pomonella* | XP_061729746.1 | AMP | 0.9989 |
| *Cydia pomonella* | Cpom_Bit_LysC_143 | AMP | 0.9838 |
| *Cydia splendana* | XP_063619140.1 | AMP | 0.9994 |
| *Cydia splendana* | XP_063628351.1 | AMP | 0.9997 |
| *Cydia splendana* | XP_063628462.1 | AMP | 0.9998 |
| *Cydia splendana* | Cspl_Bit_LysC_145 | Non-AMP | 0.3916 |
| *Cylas formicarius* | XP_060528660.1 | AMP | 0.9999 |
| *Cylas formicarius* | Cfor_Bit_LysC_146 | AMP | 0.9413 |
| *Cylas formicarius* | Cfor_Bit_LysC_147 | Non-AMP | 0.2193 |
| *Cylas formicarius* | Cfor_Bit_LysC_148 | AMP | 0.6907 |
| *Dalotia coriaria* | XP_065156599.1 | AMP | 0.9961 |
| *Dalotia coriaria* | Dcor_Bit_LysC_149 | AMP | 0.9217 |
| *Dalotia coriaria* | Dcor_Bit_LysC_150 | Non-AMP | 0.0025 |
| *Dalotia coriaria* | Dcor_Bit_LysC_151 | AMP | 0.8323 |
| *Danaus plexippus* | XP_032515409.2 | AMP | 0.9999 |
| *Danaus plexippus* | Dple_Bit_LysC_152 | AMP | 0.9968 |
| *Dendroctonus ponderosae* | XP_019755695.2 | AMP | 0.5576 |
| *Dendroctonus ponderosae* | Dpon_Bit_LysC_153 | AMP | 0.9663 |
| *Diabrotica virgifera virgifera* | XP_028134252.2 | AMP | 0.998 |
| *Diabrotica virgifera virgifera* | XP_050502010.1 | AMP | 0.9731 |
| *Diabrotica virgifera virgifera* | XP_050502011.1 | AMP | 0.9969 |
| *Diabrotica virgifera virgifera* | Dvir_Bit_LysC_154 | AMP | 0.9849 |
| *Diorhabda sublineata* | XP_056631455.1 | AMP | 0.9996 |
| *Diorhabda sublineata* | Dsub_Bit_LysC_159 | AMP | 0.9875 |
| *Diprion similis* | XP_046735096.1 | AMP | 0.9766 |
| *Diprion similis* | XP_046752913.1 | AMP | 1 |
| *Drosophila albomicans* | XP_034103458.1 | AMP | 0.9999 |
| *Drosophila albomicans* | XP_034103459.1 | AMP | 0.9996 |
| *Drosophila albomicans* | XP_034103461.1 | AMP | 0.9991 |
| *Drosophila albomicans* | XP_034103462.1 | AMP | 0.9992 |
| *Drosophila albomicans* | XP_034103463.1 | AMP | 0.9996 |
| *Drosophila albomicans* | XP_034107204.1 | AMP | 0.9999 |
| *Drosophila albomicans* | XP_034109317.1 | Non-AMP | 0.0717 |
| *Drosophila albomicans* | XP_051860878.1 | AMP | 0.9996 |
| *Drosophila ananassae* | XP_001956716.1 | AMP | 0.9799 |
| *Drosophila ananassae* | XP_001956717.1 | AMP | 1 |
| *Drosophila ananassae* | XP_001956719.1 | AMP | 0.9964 |
| *Drosophila ananassae* | XP_001956720.1 | AMP | 0.995 |
| *Drosophila ananassae* | XP_001956722.1 | AMP | 0.9998 |
| *Drosophila ananassae* | XP_001956727.1 | AMP | 0.9998 |
| *Drosophila ananassae* | XP_001959002.1 | AMP | 1 |
| *Drosophila ananassae* | XP_032309533.1 | AMP | 0.9964 |
| *Drosophila ananassae* | XP_032309534.1 | AMP | 0.9996 |
| *Drosophila ananassae* | XP_032309535.1 | AMP | 0.9996 |
| *Drosophila arizonae* | XP_017860579.1 | AMP | 1 |
| *Drosophila arizonae* | XP_017863132.1 | AMP | 0.9997 |
| *Drosophila arizonae* | XP_017863134.1 | AMP | 0.9999 |
| *Drosophila arizonae* | XP_017863135.1 | AMP | 0.9967 |
| *Drosophila arizonae* | XP_017863136.1 | AMP | 0.9986 |
| *Drosophila arizonae* | XP_017865271.1 | Non-AMP | 0.3863 |
| *Drosophila biarmipes* | XP_016946698.1 | AMP | 0.9999 |
| *Drosophila biarmipes* | XP_016954853.2 | AMP | 0.9943 |
| *Drosophila biarmipes* | XP_016956872.1 | AMP | 1 |
| *Drosophila biarmipes* | XP_016956886.1 | AMP | 0.9943 |
| *Drosophila biarmipes* | XP_016956899.1 | AMP | 0.9943 |
| *Drosophila biarmipes* | XP_016956922.1 | AMP | 0.9943 |
| *Drosophila biarmipes* | XP_016956933.1 | AMP | 0.9762 |
| *Drosophila biarmipes* | XP_016959139.1 | AMP | 0.9772 |
| *Drosophila biarmipes* | XP_050741955.1 | AMP | 0.9813 |
| *Drosophila bipectinata* | XP_017093461.1 | AMP | 1 |
| *Drosophila bipectinata* | XP_017103990.2 | AMP | 0.9999 |
| *Drosophila bipectinata* | XP_017103991.2 | AMP | 1 |
| *Drosophila bipectinata* | XP_017103992.1 | AMP | 0.9964 |
| *Drosophila bipectinata* | XP_017103993.1 | AMP | 0.9964 |
| *Drosophila bipectinata* | XP_017103994.2 | AMP | 0.9999 |
| *Drosophila bipectinata* | XP_017103995.2 | AMP | 0.9895 |
| *Drosophila bipectinata* | XP_017103996.2 | AMP | 0.9997 |
| *Drosophila bipectinata* | XP_017104049.2 | AMP | 0.9656 |
| *Drosophila bipectinata* | XP_043069954.1 | AMP | 0.9799 |
| *Drosophila elegans* | XP_017124426.1 | AMP | 1 |
| *Drosophila elegans* | XP_017126756.1 | AMP | 0.9997 |
| *Drosophila elegans* | XP_017127138.1 | AMP | 1 |
| *Drosophila elegans* | XP_017127139.1 | AMP | 0.9978 |
| *Drosophila elegans* | XP_017127140.1 | AMP | 0.9978 |
| *Drosophila elegans* | XP_017127141.1 | AMP | 0.998 |
| *Drosophila elegans* | XP_017131951.1 | AMP | 0.6615 |
| *Drosophila erecta* | XP_001971088.1 | AMP | 0.9984 |
| *Drosophila erecta* | XP_001971094.1 | AMP | 0.9981 |
| *Drosophila erecta* | XP_001971096.1 | AMP | 1 |
| *Drosophila erecta* | XP_001971097.1 | AMP | 0.9996 |
| *Drosophila erecta* | XP_001975398.1 | AMP | 0.9998 |
| *Drosophila erecta* | XP_001982998.1 | AMP | 0.9981 |
| *Drosophila erecta* | XP_001982999.1 | AMP | 0.9981 |
| *Drosophila erecta* | XP_015012801.2 | Non-AMP | 0.0619 |
| *Drosophila erecta* | XP_026833569.1 | AMP | 0.9981 |
| *Drosophila eugracilis* | XP_017063418.1 | AMP | 0.9986 |
| *Drosophila eugracilis* | XP_017063430.1 | AMP | 0.9965 |
| *Drosophila eugracilis* | XP_017063457.1 | AMP | 1 |
| *Drosophila eugracilis* | XP_017063458.1 | AMP | 0.9962 |
| *Drosophila eugracilis* | XP_017063459.1 | AMP | 0.9962 |
| *Drosophila eugracilis* | XP_017063460.1 | AMP | 0.9962 |
| *Drosophila eugracilis* | XP_017063461.1 | AMP | 0.9959 |
| *Drosophila eugracilis* | XP_017063462.1 | AMP | 0.9984 |
| *Drosophila eugracilis* | XP_017063463.1 | AMP | 0.9591 |
| *Drosophila eugracilis* | XP_017078913.1 | AMP | 0.9999 |
| *Drosophila eugracilis* | XP_017081553.1 | Non-AMP | 0.1446 |
| *Drosophila ficusphila* | XP_017049345.1 | AMP | 0.9999 |
| *Drosophila ficusphila* | XP_017051123.1 | Non-AMP | 0.4818 |
| *Drosophila ficusphila* | XP_017059465.1 | AMP | 0.989 |
| *Drosophila ficusphila* | XP_017059589.1 | AMP | 1 |
| *Drosophila ficusphila* | XP_017059591.1 | AMP | 0.9942 |
| *Drosophila ficusphila* | XP_017059593.1 | AMP | 0.9996 |
| *Drosophila ficusphila* | XP_017059594.1 | AMP | 0.998 |
| *Drosophila ficusphila* | XP_017059596.1 | AMP | 0.9984 |
| *Drosophila ficusphila* | Dfic_Bit_LysC_189 | AMP | 0.9984 |
| *Drosophila grimshawi* | XP_001983604.1 | AMP | 0.9999 |
| *Drosophila grimshawi* | XP_001983606.1 | AMP | 0.9992 |
| *Drosophila grimshawi* | XP_001983607.1 | AMP | 0.9999 |
| *Drosophila grimshawi* | XP_001983608.1 | AMP | 0.9999 |
| *Drosophila grimshawi* | XP_001983609.1 | AMP | 0.9999 |
| *Drosophila grimshawi* | XP_001985890.1 | AMP | 1 |
| *Drosophila grimshawi* | XP_032597148.1 | Non-AMP | 0.363 |
| *Drosophila guanche* | XP_034131453.1 | AMP | 0.9998 |
| *Drosophila guanche* | XP_034133758.1 | AMP | 0.9974 |
| *Drosophila guanche* | XP_034133759.1 | AMP | 1 |
| *Drosophila guanche* | XP_034133760.1 | AMP | 0.9865 |
| *Drosophila guanche* | XP_034133762.1 | AMP | 0.9865 |
| *Drosophila guanche* | XP_034133763.1 | AMP | 0.9956 |
| *Drosophila guanche* | XP_034133765.1 | AMP | 0.9956 |
| *Drosophila guanche* | XP_034133766.1 | AMP | 0.9956 |
| *Drosophila guanche* | XP_034133767.1 | AMP | 0.9956 |
| *Drosophila guanche* | XP_034137934.1 | AMP | 0.8517 |
| *Drosophila hydei* | XP_023165164.2 | AMP | 0.9996 |
| *Drosophila hydei* | XP_023167108.1 | AMP | 0.9998 |
| *Drosophila hydei* | XP_023167109.2 | AMP | 0.9855 |
| *Drosophila hydei* | XP_023167110.1 | AMP | 0.9855 |
| *Drosophila hydei* | XP_023167111.1 | AMP | 1 |
| *Drosophila hydei* | XP_023168800.2 | AMP | 0.808 |
| *Drosophila hydei* | XP_023169112.1 | AMP | 0.9999 |
| *Drosophila hydei* | XP_030079472.1 | AMP | 0.9999 |
| *Drosophila hydei* | XP_030079478.1 | AMP | 0.9988 |
| *Drosophila hydei* | XP_030079480.1 | AMP | 0.9704 |
| *Drosophila hydei* | XP_030079481.1 | AMP | 0.9855 |
| *Drosophila hydei* | XP_030079514.1 | AMP | 0.9998 |
| *Drosophila hydei* | XP_030079515.1 | AMP | 0.9704 |
| *Drosophila kikkawai* | XP_017017725.2 | AMP | 0.9998 |
| *Drosophila kikkawai* | XP_017017741.1 | AMP | 0.9954 |
| *Drosophila kikkawai* | XP_017017764.1 | AMP | 0.9724 |
| *Drosophila kikkawai* | XP_017017765.1 | AMP | 0.9998 |
| *Drosophila kikkawai* | XP_017017767.1 | AMP | 0.9998 |
| *Drosophila kikkawai* | XP_017017770.1 | AMP | 0.9998 |
| *Drosophila kikkawai* | XP_017017771.1 | AMP | 0.9991 |
| *Drosophila kikkawai* | XP_017033212.1 | AMP | 1 |
| *Drosophila kikkawai* | XP_041631408.1 | AMP | 0.9998 |
| *Drosophila kikkawai* | XP_041631409.1 | AMP | 0.9998 |
| *Drosophila kikkawai* | XP_041632263.1 | AMP | 0.9954 |
| *Drosophila kikkawai* | XP_041632265.1 | AMP | 0.9999 |
| *Drosophila kikkawai* | XP_041632269.1 | AMP | 0.9998 |
| *Drosophila kikkawai* | XP_041632270.1 | AMP | 0.9996 |
| *Drosophila kikkawai* | XP_041632271.1 | AMP | 0.9998 |
| *Drosophila kikkawai* | XP_041632272.1 | AMP | 0.9724 |
| *Drosophila kikkawai* | XP_041632273.1 | AMP | 0.9991 |
| *Drosophila kikkawai* | Dkik_Bit_LysC_206 | AMP | 0.9987 |
| *Drosophila mauritiana* | XP_033156103.1 | AMP | 0.9999 |
| *Drosophila mauritiana* | XP_033156793.1 | Non-AMP | 0.0459 |
| *Drosophila mauritiana* | XP_033157261.1 | AMP | 0.9992 |
| *Drosophila mauritiana* | XP_033157262.1 | AMP | 1 |
| *Drosophila mauritiana* | XP_033157263.1 | AMP | 0.9984 |
| *Drosophila mauritiana* | XP_033157264.1 | AMP | 0.9984 |
| *Drosophila mauritiana* | XP_033157265.1 | AMP | 0.9992 |
| *Drosophila mauritiana* | XP_033157266.1 | AMP | 0.9942 |
| *Drosophila mauritiana* | XP_033158206.1 | AMP | 0.9654 |
| *Drosophila melanogaster* | NP_001261245.1 | AMP | 0.9944 |
| *Drosophila melanogaster* | NP_476823.1 | AMP | 0.9944 |
| *Drosophila melanogaster* | NP_476827.2 | AMP | 0.9997 |
| *Drosophila melanogaster* | NP_476828.1 | AMP | 1 |
| *Drosophila melanogaster* | NP_476829.1 | AMP | 0.9859 |
| *Drosophila melanogaster* | NP_523881.1 | AMP | 0.9998 |
| *Drosophila melanogaster* | NP_611098.1 | AMP | 0.9999 |
| *Drosophila melanogaster* | NP_725299.2 | Non-AMP | 0.1161 |
| *Drosophila miranda* | XP_017137322.1 | AMP | 0.9986 |
| *Drosophila miranda* | XP_017137323.2 | AMP | 0.9986 |
| *Drosophila miranda* | XP_017137324.1 | AMP | 0.9996 |
| *Drosophila miranda* | XP_017137325.1 | AMP | 1 |
| *Drosophila miranda* | XP_017137327.1 | AMP | 0.9888 |
| *Drosophila miranda* | XP_017137329.2 | AMP | 0.9988 |
| *Drosophila miranda* | XP_017137331.1 | AMP | 0.9967 |
| *Drosophila miranda* | XP_017137333.2 | AMP | 0.9977 |
| *Drosophila miranda* | XP_017137334.2 | AMP | 0.9977 |
| *Drosophila miranda* | XP_017137336.1 | AMP | 0.9988 |
| *Drosophila miranda* | XP_017146199.1 | AMP | 0.9998 |
| *Drosophila miranda* | XP_033242420.1 | AMP | 0.9977 |
| *Drosophila miranda* | XP_033251072.1 | AMP | 0.9966 |
| *Drosophila miranda* | XP_033253905.1 | AMP | 1 |
| *Drosophila miranda* | XP_033253907.1 | AMP | 1 |
| *Drosophila mojavensis* | XP_002006261.1 | AMP | 0.9998 |
| *Drosophila mojavensis* | XP_002012057.1 | AMP | 0.9987 |
| *Drosophila mojavensis* | XP_002012058.1 | AMP | 0.9987 |
| *Drosophila mojavensis* | XP_002012059.1 | AMP | 1 |
| *Drosophila mojavensis* | XP_002012060.1 | AMP | 0.9987 |
| *Drosophila mojavensis* | XP_002012061.1 | AMP | 0.9994 |
| *Drosophila mojavensis* | XP_002012064.1 | AMP | 0.9993 |
| *Drosophila mojavensis* | XP_002012065.1 | AMP | 0.9993 |
| *Drosophila mojavensis* | XP_043866731.1 | AMP | 0.583 |
| *Drosophila montana* | XP_064536044.1 | AMP | 0.8538 |
| *Drosophila montana* | XP_064541585.1 | AMP | 0.9696 |
| *Drosophila montana* | XP_064541586.1 | AMP | 0.9539 |
| *Drosophila montana* | XP_064541587.1 | AMP | 0.7473 |
| *Drosophila montana* | XP_064541588.1 | AMP | 0.7375 |
| *Drosophila montana* | XP_064541590.1 | AMP | 0.9078 |
| *Drosophila montana* | XP_064541634.1 | AMP | 0.9999 |
| *Drosophila montana* | XP_064541636.1 | AMP | 0.8686 |
| *Drosophila montana* | XP_064549010.1 | AMP | 0.9997 |
| *Drosophila nasuta* | XP_060656560.1 | AMP | 0.9999 |
| *Drosophila nasuta* | XP_060658458.1 | AMP | 0.9992 |
| *Drosophila nasuta* | XP_060660534.1 | Non-AMP | 0.0958 |
| *Drosophila nasuta* | XP_060660913.1 | AMP | 1 |
| *Drosophila nasuta* | XP_060660914.1 | AMP | 0.9996 |
| *Drosophila nasuta* | XP_060660915.1 | AMP | 0.9996 |
| *Drosophila nasuta* | XP_060660916.1 | AMP | 0.9996 |
| *Drosophila nasuta* | XP_060660917.1 | AMP | 0.9994 |
| *Drosophila nasuta* | XP_060660919.1 | AMP | 0.9988 |
| *Drosophila navojoa* | XP_017956436.1 | AMP | 0.9997 |
| *Drosophila navojoa* | XP_017956439.2 | AMP | 0.9998 |
| *Drosophila navojoa* | XP_017959361.1 | AMP | 0.6449 |
| *Drosophila navojoa* | XP_017960749.1 | AMP | 0.9998 |
| *Drosophila navojoa* | XP_030241748.1 | AMP | 0.9997 |
| *Drosophila navojoa* | XP_030241749.1 | AMP | 0.9998 |
| *Drosophila navojoa* | XP_030241750.1 | AMP | 0.9998 |
| *Drosophila navojoa* | XP_030241751.1 | AMP | 0.9997 |
| *Drosophila navojoa* | XP_030241752.1 | AMP | 0.9997 |
| *Drosophila novamexicana* | XP_030554350.1 | AMP | 0.9998 |
| *Drosophila novamexicana* | XP_030560717.1 | AMP | 1 |
| *Drosophila novamexicana* | XP_030560718.1 | AMP | 0.9996 |
| *Drosophila novamexicana* | XP_030560719.1 | AMP | 0.9983 |
| *Drosophila novamexicana* | XP_030560720.1 | AMP | 0.9851 |
| *Drosophila novamexicana* | XP_030560721.1 | AMP | 0.9347 |
| *Drosophila novamexicana* | XP_030560723.1 | AMP | 0.9999 |
| *Drosophila novamexicana* | XP_030560724.1 | AMP | 0.9347 |
| *Drosophila novamexicana* | XP_030563572.1 | AMP | 0.5183 |
| *Drosophila obscura* | XP_022224620.2 | AMP | 0.9422 |
| *Drosophila obscura* | XP_022226708.2 | AMP | 0.9997 |
| *Drosophila obscura* | XP_022226709.2 | AMP | 0.9858 |
| *Drosophila obscura* | XP_022226710.1 | AMP | 0.9865 |
| *Drosophila obscura* | XP_022226712.2 | AMP | 0.994 |
| *Drosophila obscura* | XP_022226714.2 | AMP | 0.994 |
| *Drosophila obscura* | XP_022226717.1 | AMP | 0.994 |
| *Drosophila obscura* | XP_022226718.1 | AMP | 0.994 |
| *Drosophila obscura* | XP_022227198.2 | AMP | 0.9999 |
| *Drosophila obscura* | XP_041450587.1 | AMP | 0.994 |
| *Drosophila persimilis* | XP_002026115.1 | AMP | 0.9977 |
| *Drosophila persimilis* | XP_002026116.1 | AMP | 0.9964 |
| *Drosophila persimilis* | XP_002026117.2 | AMP | 0.9977 |
| *Drosophila persimilis* | XP_002026118.2 | AMP | 0.9964 |
| *Drosophila persimilis* | XP_002026119.1 | AMP | 0.9977 |
| *Drosophila persimilis* | XP_002026120.1 | AMP | 0.9996 |
| *Drosophila persimilis* | XP_002029500.2 | AMP | 0.9964 |
| *Drosophila persimilis* | XP_026840814.1 | AMP | 0.9999 |
| *Drosophila persimilis* | XP_026849743.1 | AMP | 0.9888 |
| *Drosophila persimilis* | XP_026849746.1 | AMP | 0.9964 |
| *Drosophila persimilis* | XP_026849747.1 | AMP | 0.9964 |
| *Drosophila persimilis* | XP_026849748.1 | AMP | 1 |
| *Drosophila pseudoobscura* | XP_001352467.1 | AMP | 0.9888 |
| *Drosophila pseudoobscura* | XP_002135314.1 | AMP | 1 |
| *Drosophila pseudoobscura* | XP_002135317.2 | AMP | 0.9964 |
| *Drosophila pseudoobscura* | XP_002135318.2 | AMP | 0.9964 |
| *Drosophila pseudoobscura* | XP_002135319.3 | AMP | 0.9964 |
| *Drosophila pseudoobscura* | XP_002135320.3 | AMP | 0.9964 |
| *Drosophila pseudoobscura* | XP_002135321.1 | AMP | 0.9962 |
| *Drosophila pseudoobscura* | XP_002135322.1 | AMP | 0.9964 |
| *Drosophila pseudoobscura* | XP_002135323.1 | AMP | 0.9964 |
| *Drosophila pseudoobscura* | XP_002135324.1 | AMP | 0.9975 |
| *Drosophila pseudoobscura* | XP_033234778.1 | AMP | 0.9999 |
| *Drosophila rhopaloa* | XP_016969740.2 | AMP | 0.9976 |
| *Drosophila rhopaloa* | XP_016969769.2 | AMP | 0.9975 |
| *Drosophila rhopaloa* | XP_016973307.1 | AMP | 0.7834 |
| *Drosophila rhopaloa* | XP_016984106.1 | AMP | 0.9994 |
| *Drosophila rhopaloa* | XP_016984107.1 | AMP | 0.9994 |
| *Drosophila rhopaloa* | XP_016984109.1 | AMP | 0.9606 |
| *Drosophila rhopaloa* | XP_016984110.2 | AMP | 0.9606 |
| *Drosophila rhopaloa* | XP_016984111.1 | AMP | 0.9994 |
| *Drosophila rhopaloa* | XP_016985964.1 | AMP | 0.9999 |
| *Drosophila rhopaloa* | XP_016988508.1 | AMP | 0.9777 |
| *Drosophila rhopaloa* | XP_044313922.1 | AMP | 0.9994 |
| *Drosophila rhopaloa* | XP_044313923.1 | AMP | 1 |
| *Drosophila sechellia* | XP_002034086.1 | AMP | 0.9999 |
| *Drosophila sechellia* | XP_002034869.1 | AMP | 0.9992 |
| *Drosophila sechellia* | XP_002034870.1 | AMP | 0.9984 |
| *Drosophila sechellia* | XP_002034871.1 | AMP | 0.9984 |
| *Drosophila sechellia* | XP_002034873.2 | AMP | 0.9964 |
| *Drosophila sechellia* | XP_032575946.1 | AMP | 1 |
| *Drosophila serrata* | XP_020798342.1 | AMP | 1 |
| *Drosophila serrata* | XP_020801510.1 | AMP | 0.9995 |
| *Drosophila serrata* | XP_020801517.1 | AMP | 0.979 |
| *Drosophila serrata* | XP_020801518.1 | AMP | 0.9991 |
| *Drosophila serrata* | XP_020801519.1 | AMP | 0.9996 |
| *Drosophila serrata* | XP_020801520.1 | AMP | 0.9981 |
| *Drosophila serrata* | XP_020801521.1 | AMP | 0.9984 |
| *Drosophila serrata* | XP_020801523.1 | AMP | 0.9993 |
| *Drosophila serrata* | XP_020801524.1 | AMP | 0.9998 |
| *Drosophila serrata* | XP_020806014.1 | AMP | 0.9495 |
| *Drosophila simulans* | XP_002076490.1 | AMP | 0.9942 |
| *Drosophila simulans* | XP_002083176.1 | AMP | 0.9544 |
| *Drosophila simulans* | XP_002083180.1 | AMP | 0.9992 |
| *Drosophila simulans* | XP_002083181.2 | AMP | 0.9964 |
| *Drosophila simulans* | XP_016022646.2 | AMP | 0.9942 |
| *Drosophila simulans* | XP_016029776.1 | AMP | 1 |
| *Drosophila simulans* | XP_016029777.2 | AMP | 0.9984 |
| *Drosophila simulans* | XP_039147853.1 | AMP | 0.9999 |
| *Drosophila simulans* | XP_039149016.1 | AMP | 0.9984 |
| *Drosophila subobscura* | XP_034652295.1 | AMP | 0.7912 |
| *Drosophila subobscura* | XP_034654347.1 | AMP | 0.9998 |
| *Drosophila subobscura* | XP_034655124.1 | AMP | 0.9978 |
| *Drosophila subobscura* | XP_034655125.1 | AMP | 0.9999 |
| *Drosophila subobscura* | XP_034655126.1 | AMP | 0.9968 |
| *Drosophila subobscura* | XP_034655127.1 | AMP | 0.9865 |
| *Drosophila subobscura* | XP_034655128.1 | AMP | 0.9998 |
| *Drosophila subobscura* | XP_034655130.1 | AMP | 0.9956 |
| *Drosophila subobscura* | XP_034655132.1 | AMP | 0.9978 |
| *Drosophila subobscura* | XP_034655134.1 | AMP | 0.9959 |
| *Drosophila subpulchrella* | XP_037714944.1 | AMP | 0.9998 |
| *Drosophila subpulchrella* | XP_037719826.1 | AMP | 0.9539 |
| *Drosophila subpulchrella* | XP_037722981.1 | AMP | 1 |
| *Drosophila subpulchrella* | XP_037722982.1 | AMP | 0.9668 |
| *Drosophila subpulchrella* | XP_037722984.1 | AMP | 0.9961 |
| *Drosophila subpulchrella* | XP_037722985.1 | AMP | 0.9606 |
| *Drosophila subpulchrella* | XP_037722986.1 | AMP | 0.994 |
| *Drosophila subpulchrella* | XP_037722987.1 | AMP | 0.9961 |
| *Drosophila sulfurigaster albostrigata* | XP_062128615.1 | AMP | 0.9999 |
| *Drosophila sulfurigaster albostrigata* | XP_062130795.1 | AMP | 1 |
| *Drosophila sulfurigaster albostrigata* | XP_062130796.1 | AMP | 0.9988 |
| *Drosophila sulfurigaster albostrigata* | XP_062130797.1 | AMP | 0.9994 |
| *Drosophila sulfurigaster albostrigata* | XP_062130798.1 | AMP | 0.9994 |
| *Drosophila sulfurigaster albostrigata* | XP_062130799.1 | AMP | 0.9994 |
| *Drosophila sulfurigaster albostrigata* | XP_062130800.1 | AMP | 0.9996 |
| *Drosophila sulfurigaster albostrigata* | XP_062130801.1 | AMP | 0.9992 |
| *Drosophila sulfurigaster albostrigata* | XP_062136347.1 | Non-AMP | 0.0828 |
| *Drosophila suzukii* | XP_016928324.1 | AMP | 0.9999 |
| *Drosophila suzukii* | XP_016933529.1 | AMP | 0.9548 |
| *Drosophila suzukii* | XP_016934098.1 | AMP | 1 |
| *Drosophila suzukii* | XP_016934099.1 | AMP | 0.9961 |
| *Drosophila suzukii* | XP_016934100.1 | AMP | 0.9961 |
| *Drosophila suzukii* | XP_016934101.1 | AMP | 0.9668 |
| *Drosophila suzukii* | XP_016934102.2 | AMP | 0.9606 |
| *Drosophila suzukii* | XP_036670261.1 | AMP | 0.9961 |
| *Drosophila takahashii* | XP_016993183.1 | AMP | 0.9987 |
| *Drosophila takahashii* | XP_016993203.2 | AMP | 0.9987 |
| *Drosophila takahashii* | XP_016993204.2 | AMP | 0.9704 |
| *Drosophila takahashii* | XP_016993205.2 | AMP | 1 |
| *Drosophila takahashii* | XP_016993206.1 | AMP | 0.9809 |
| *Drosophila takahashii* | XP_016993207.2 | AMP | 0.9987 |
| *Drosophila takahashii* | XP_016993208.2 | AMP | 0.9997 |
| *Drosophila takahashii* | XP_016993244.2 | AMP | 0.9991 |
| *Drosophila takahashii* | XP_016993245.2 | AMP | 0.7685 |
| *Drosophila takahashii* | XP_017010131.2 | AMP | 0.9997 |
| *Drosophila takahashii* | XP_044250346.1 | AMP | 0.9997 |
| *Drosophila teissieri* | XP_043644553.1 | AMP | 0.9998 |
| *Drosophila teissieri* | XP_043650677.1 | AMP | 0.9942 |
| *Drosophila teissieri* | XP_043650678.1 | AMP | 0.9973 |
| *Drosophila teissieri* | XP_043650679.1 | AMP | 1 |
| *Drosophila teissieri* | XP_043650680.1 | AMP | 0.9967 |
| *Drosophila teissieri* | XP_043650681.1 | AMP | 0.9967 |
| *Drosophila teissieri* | XP_043650683.1 | AMP | 0.9967 |
| *Drosophila teissieri* | Dtei_Bit_LysC_277 | AMP | 0.9952 |
| *Drosophila teissieri* | Dtei_Bit_LysC_278 | AMP | 0.9762 |
| *Drosophila virilis* | XP_002046428.1 | AMP | 0.9999 |
| *Drosophila virilis* | XP_002046430.1 | AMP | 1 |
| *Drosophila virilis* | XP_002046431.1 | AMP | 0.9347 |
| *Drosophila virilis* | XP_002046433.1 | AMP | 0.9347 |
| *Drosophila virilis* | XP_002046434.2 | AMP | 0.9851 |
| *Drosophila virilis* | XP_002046436.1 | AMP | 0.9982 |
| *Drosophila virilis* | XP_002049593.1 | AMP | 0.9999 |
| *Drosophila virilis* | XP_015030333.1 | Non-AMP | 0.4118 |
| *Drosophila virilis* | XP_032289303.1 | AMP | 0.9982 |
| *Drosophila virilis* | XP_032289307.1 | AMP | 0.9851 |
| *Drosophila virilis* | XP_032289312.1 | AMP | 0.9347 |
| *Drosophila virilis* | XP_032291322.1 | AMP | 0.9995 |
| *Drosophila virilis* | XP_032292738.1 | AMP | 0.9999 |
| *Drosophila yakuba* | XP_002092255.1 | AMP | 0.9998 |
| *Drosophila yakuba* | XP_002093075.1 | AMP | 0.9962 |
| *Drosophila yakuba* | XP_002093080.1 | AMP | 0.9971 |
| *Drosophila yakuba* | XP_002093081.1 | AMP | 0.9987 |
| *Drosophila yakuba* | XP_002093082.1 | AMP | 0.9987 |
| *Drosophila yakuba* | XP_002093083.1 | AMP | 0.9975 |
| *Drosophila yakuba* | XP_002093084.1 | AMP | 1 |
| *Drosophila yakuba* | XP_002093085.1 | AMP | 0.9859 |
| *Episyrphus balteatus* | XP_055850699.1 | AMP | 0.9998 |
| *Episyrphus balteatus* | Ebal_Bit_LysC_287 | AMP | 0.9996 |
| *Eupeodes corollae* | XP_055906822.1 | AMP | 0.9962 |
| *Eupeodes corollae* | Ecor_Bit_LysC_291 | AMP | 0.9987 |
| *Folsomia candida* | XP_021955478.1 | AMP | 0.9984 |
| *Folsomia candida* | XP_021968137.1 | AMP | 0.9941 |
| *Folsomia candida* | Fcan_Bit_LysC_294 | AMP | 0.6207 |
| *Frankliniella occidentalis* | XP_026284668.1 | AMP | 0.9999 |
| *Frankliniella occidentalis* | XP_026287319.1 | AMP | 0.9999 |
| *Frankliniella occidentalis* | XP_026291031.2 | AMP | 0.9999 |
| *Frankliniella occidentalis* | XP_052122717.1 | AMP | 0.9998 |
| *Galleria mellonella* | XP_026750267.2 | AMP | 0.9999 |
| *Galleria mellonella* | XP_026755257.1 | AMP | 0.9999 |
| *Galleria mellonella* | XP_052756229.1 | AMP | 0.9999 |
| *Galleria mellonella* | Gmel_Bit_LysC_297 | Non-AMP | 0.3389 |
| *Glossina fuscipes* | XP_037887180.1 | Non-AMP | 0.1388 |
| *Gryllus longicercus* | KAK7788628.1 | AMP | 0.9998 |
| *Gryllus longicercus* | KAK7790150.1 | AMP | 0.9999 |
| *Gryllus longicercus* | KAK7864931.1 | AMP | 0.9994 |
| *Gryllus longicercus* | KAK7872489.1 | AMP | 0.9995 |
| *Harmonia axyridis* | XP_045463003.1 | AMP | 0.9219 |
| *Harmonia axyridis* | XP_045473803.1 | AMP | 0.9996 |
| *Harmonia axyridis* | XP_045480522.1 | AMP | 0.9992 |
| *Harmonia axyridis* | Haxy_Bit_LysC_302 | Non-AMP | 0.1593 |
| *Helicoverpa armigera* | XP_049693234.2 | AMP | 0.9999 |
| *Helicoverpa armigera* | XP_049696526.1 | AMP | 0.9997 |
| *Helicoverpa armigera* | XP_049696527.2 | AMP | 0.9999 |
| *Helicoverpa zea* | XP_047019562.1 | AMP | 1 |
| *Helicoverpa zea* | XP_047019592.1 | AMP | 0.9997 |
| *Helicoverpa zea* | XP_047026248.1 | AMP | 0.9999 |
| *Hermetia illucens* | XP_037915159.1 | AMP | 1 |
| *Hermetia illucens* | XP_037915160.1 | AMP | 1 |
| *Hermetia illucens* | XP_037915161.1 | AMP | 1 |
| *Hermetia illucens* | XP_037915163.1 | AMP | 1 |
| *Hermetia illucens* | XP_037915164.1 | AMP | 1 |
| *Hermetia illucens* | XP_037915165.1 | AMP | 1 |
| *Hermetia illucens* | XP_037915166.1 | AMP | 1 |
| *Hermetia illucens* | XP_037915170.1 | AMP | 1 |
| *Hermetia illucens* | XP_037915171.1 | AMP | 1 |
| *Hermetia illucens* | XP_037915726.1 | AMP | 0.9999 |
| *Hermetia illucens* | XP_037915727.1 | AMP | 0.9999 |
| *Hermetia illucens* | XP_037915916.1 | AMP | 0.9997 |
| *Hermetia illucens* | XP_037915947.1 | AMP | 1 |
| *Hermetia illucens* | XP_037916025.1 | AMP | 1 |
| *Hermetia illucens* | XP_037916066.1 | AMP | 0.9998 |
| *Hermetia illucens* | XP_037916109.1 | AMP | 1 |
| *Hermetia illucens* | XP_037916134.1 | AMP | 0.988 |
| *Hermetia illucens* | XP_037916159.1 | AMP | 1 |
| *Hermetia illucens* | XP_037916186.1 | AMP | 1 |
| *Hermetia illucens* | XP_037916231.1 | AMP | 1 |
| *Hermetia illucens* | XP_037916232.1 | AMP | 1 |
| *Hermetia illucens* | XP_037916382.1 | AMP | 0.9998 |
| *Hermetia illucens* | XP_037916493.1 | AMP | 0.9999 |
| *Hermetia illucens* | XP_037916606.1 | AMP | 0.9999 |
| *Hermetia illucens* | XP_037916608.1 | AMP | 0.9999 |
| *Hermetia illucens* | XP_037916609.1 | AMP | 0.9998 |
| *Hermetia illucens* | XP_037916627.1 | AMP | 1 |
| *Hermetia illucens* | XP_037916836.1 | AMP | 1 |
| *Hermetia illucens* | XP_037916841.1 | AMP | 1 |
| *Hermetia illucens* | XP_037916843.1 | AMP | 1 |
| *Hermetia illucens* | XP_037917973.1 | AMP | 1 |
| *Hermetia illucens* | XP_037918653.1 | AMP | 0.9999 |
| *Hermetia illucens* | Hill_Bit_LysC_310 | AMP | 1 |
| *Hetaerina americana* | XP_071449479.1 | AMP | 0.9948 |
| *Homalodisca vitripennis* | Hvit_Bit_LysC_311 | Non-AMP | 0.2542 |
| *Homalodisca vitripennis* | Hvit_Bit_LysC_312 | Non-AMP | 0.2446 |
| *Hyposmocoma kahamanoa* | XP_026318553.1 | AMP | 0.9848 |
| *Hyposmocoma kahamanoa* | Hkah_Bit_LysC_313 | AMP | 0.8947 |
| *Ischnura elegans* | Iele_Bit_LysC_315 | AMP | 0.9846 |
| *Lamprigera yunnana* | Lyun_Bit_LysC_316 | AMP | 0.5809 |
| *Laodelphax striatellus* | RZF40092.1 | AMP | 0.9886 |
| *Leguminivora glycinivorella* | XP_047985978.1 | AMP | 0.9955 |
| *Leguminivora glycinivorella* | XP_047990122.1 | AMP | 0.9998 |
| *Leguminivora glycinivorella* | XP_047992391.1 | AMP | 0.9999 |
| *Leguminivora glycinivorella* | XP_047993532.1 | AMP | 0.9957 |
| *Leptidea sinapis* | XP_050668783.1 | AMP | 1 |
| *Leptidea sinapis* | Lsin_Bit_LysC_317 | AMP | 0.9353 |
| *Leptinotarsa decemlineata* | Ldec_Bit_LysC_319 | AMP | 0.9948 |
| *Lucilia cuprina* | XP_023294221.2 | AMP | 0.9988 |
| *Lucilia cuprina* | XP_023294222.2 | AMP | 0.9985 |
| *Lucilia cuprina* | XP_023294224.2 | AMP | 0.9988 |
| *Lucilia cuprina* | XP_023295380.2 | AMP | 0.9947 |
| *Lucilia cuprina* | XP_023295381.2 | AMP | 0.9999 |
| *Lucilia cuprina* | XP_023296952.2 | AMP | 0.9995 |
| *Lucilia cuprina* | XP_023304564.2 | AMP | 1 |
| *Lucilia cuprina* | XP_023304565.2 | AMP | 1 |
| *Lucilia cuprina* | XP_023304566.2 | AMP | 1 |
| *Lucilia cuprina* | XP_023304567.1 | AMP | 1 |
| *Lucilia cuprina* | XP_046811323.1 | AMP | 0.894 |
| *Lucilia cuprina* | XP_046811569.1 | AMP | 0.9978 |
| *Lucilia cuprina* | XP_046811570.1 | AMP | 0.9999 |
| *Lucilia cuprina* | XP_046811571.1 | AMP | 0.9999 |
| *Lucilia cuprina* | XP_046811572.1 | AMP | 0.9988 |
| *Lucilia cuprina* | XP_046811574.1 | AMP | 0.9999 |
| *Lucilia cuprina* | XP_046811575.1 | AMP | 1 |
| *Lucilia sericata* | XP_037807016.1 | AMP | 0.9993 |
| *Lucilia sericata* | XP_037807017.1 | AMP | 0.9997 |
| *Lucilia sericata* | XP_037807018.1 | AMP | 0.9994 |
| *Lucilia sericata* | XP_037813930.1 | AMP | 0.9933 |
| *Lucilia sericata* | XP_037813931.1 | AMP | 1 |
| *Lucilia sericata* | XP_037813933.1 | AMP | 0.9997 |
| *Lucilia sericata* | XP_037813934.1 | AMP | 0.9998 |
| *Lucilia sericata* | XP_037813935.1 | AMP | 0.9998 |
| *Lucilia sericata* | XP_037813936.1 | AMP | 1 |
| *Lucilia sericata* | XP_037813957.1 | AMP | 0.9997 |
| *Lucilia sericata* | XP_037819680.1 | AMP | 1 |
| *Lucilia sericata* | XP_037819683.1 | AMP | 1 |
| *Lucilia sericata* | XP_037819684.1 | AMP | 1 |
| *Lucilia sericata* | XP_037819685.1 | AMP | 1 |
| *Lucilia sericata* | XP_037819686.1 | AMP | 1 |
| *Lucilia sericata* | XP_037819687.1 | AMP | 1 |
| *Lucilia sericata* | XP_037819688.1 | AMP | 1 |
| *Lutzomyia longipalpis* | XP_055679740.1 | AMP | 0.7595 |
| *Lutzomyia longipalpis* | XP_055679751.1 | AMP | 0.9999 |
| *Lutzomyia longipalpis* | XP_055679766.1 | AMP | 0.9995 |
| *Lutzomyia longipalpis* | XP_055679781.1 | AMP | 0.9997 |
| *Lutzomyia longipalpis* | Llon_Bit_LysC_331 | AMP | 0.9999 |
| *Malaya genurostris* | XP_058456243.1 | AMP | 1 |
| *Malaya genurostris* | XP_058456244.1 | AMP | 1 |
| *Malaya genurostris* | XP_058456245.1 | AMP | 1 |
| *Malaya genurostris* | XP_058456246.1 | AMP | 1 |
| *Manduca sexta* | XP_030024172.2 | AMP | 0.9997 |
| *Manduca sexta* | XP_037298374.1 | AMP | 0.9997 |
| *Manduca sexta* | XP_037302755.1 | AMP | 0.9997 |
| *Manduca sexta* | Msex_Bit_LysC_336 | AMP | 0.9992 |
| *Maniola hyperantus* | XP_034826897.1 | AMP | 1 |
| *Maniola hyperantus* | Mhyp_Bit_LysC_337 | AMP | 0.9926 |
| *Maniola jurtina* | XP_045768363.1 | AMP | 1 |
| *Maniola jurtina* | Mjur_Bit_LysC_340 | AMP | 0.9453 |
| *Melitaea cinxia* | XP_045447626.1 | AMP | 0.9995 |
| *Melitaea cinxia* | Mcin_Bit_LysC_341 | AMP | 0.937 |
| *Musca domestica* | NP_001295901.1 | AMP | 0.9986 |
| *Musca domestica* | XP_005178468.1 | AMP | 0.9998 |
| *Musca domestica* | XP_005178472.1 | AMP | 0.9996 |
| *Musca domestica* | XP_005178489.1 | AMP | 0.9999 |
| *Musca domestica* | XP_005184906.2 | AMP | 0.8236 |
| *Musca domestica* | XP_005185366.1 | AMP | 0.9982 |
| *Musca domestica* | XP_005185375.2 | AMP | 0.9993 |
| *Musca domestica* | XP_005185827.1 | AMP | 0.9986 |
| *Musca domestica* | XP_005185835.2 | AMP | 0.9986 |
| *Musca domestica* | XP_005185836.1 | AMP | 0.9973 |
| *Musca domestica* | XP_011290482.1 | AMP | 0.9992 |
| *Musca domestica* | XP_011290483.1 | AMP | 0.9992 |
| *Musca domestica* | XP_011290487.2 | AMP | 0.9995 |
| *Musca domestica* | XP_011291482.2 | AMP | 1 |
| *Musca domestica* | XP_019890619.1 | AMP | 0.9988 |
| *Musca domestica* | XP_019890620.1 | AMP | 0.998 |
| *Musca domestica* | XP_019890621.1 | AMP | 0.9992 |
| *Musca domestica* | XP_019890622.1 | AMP | 0.9995 |
| *Musca domestica* | XP_019890623.1 | AMP | 0.9976 |
| *Musca domestica* | XP_019893133.2 | AMP | 0.9986 |
| *Musca domestica* | XP_019893139.2 | AMP | 0.9972 |
| *Musca domestica* | XP_019893140.1 | AMP | 0.9986 |
| *Musca domestica* | XP_058982249.1 | AMP | 0.9982 |
| *Musca domestica* | XP_058982250.1 | AMP | 0.9982 |
| *Musca domestica* | XP_058983573.1 | AMP | 0.9986 |
| *Musca domestica* | XP_058983574.1 | AMP | 0.9976 |
| *Musca domestica* | XP_058983575.1 | AMP | 0.9976 |
| *Musca domestica* | XP_058983577.1 | AMP | 0.998 |
| *Musca domestica* | XP_058983578.1 | AMP | 0.9986 |
| *Musca domestica* | XP_058983579.1 | AMP | 0.9973 |
| *Musca domestica* | XP_058983580.1 | AMP | 0.9986 |
| *Musca domestica* | XP_058983581.1 | AMP | 0.9986 |
| *Musca domestica* | XP_058983582.1 | AMP | 0.9986 |
| *Musca domestica* | XP_058983584.1 | AMP | 0.9986 |
| *Musca domestica* | XP_058983585.1 | AMP | 0.9976 |
| *Musca domestica* | XP_058983586.1 | AMP | 0.9951 |
| *Musca domestica* | XP_058984772.1 | AMP | 0.9974 |
| *Musca domestica* | Mdom_Bit_LysC_344 | AMP | 1 |
| *Musca domestica* | Mdom_Bit_LysC_345 | AMP | 0.9974 |
| *Musca domestica* | Mdom_Bit_LysC_347 | AMP | 0.9884 |
| *Musca vetustissima* | XP_061391475.1 | AMP | 0.9919 |
| *Musca vetustissima* | XP_061391476.1 | AMP | 0.9997 |
| *Musca vetustissima* | XP_061391499.1 | AMP | 0.9939 |
| *Musca vetustissima* | XP_061393098.1 | AMP | 0.9975 |
| *Musca vetustissima* | XP_061393099.1 | AMP | 0.9975 |
| *Musca vetustissima* | XP_061393100.1 | AMP | 0.9984 |
| *Musca vetustissima* | XP_061393101.1 | AMP | 0.9984 |
| *Musca vetustissima* | XP_061393102.1 | AMP | 0.9984 |
| *Musca vetustissima* | XP_061393103.1 | AMP | 0.9984 |
| *Musca vetustissima* | XP_061393105.1 | AMP | 0.9994 |
| *Musca vetustissima* | XP_061393107.1 | AMP | 0.9998 |
| *Musca vetustissima* | XP_061393134.1 | AMP | 0.9994 |
| *Musca vetustissima* | XP_061393135.1 | AMP | 0.9984 |
| *Musca vetustissima* | XP_061393136.1 | AMP | 0.9984 |
| *Musca vetustissima* | XP_061394300.1 | AMP | 1 |
| *Musca vetustissima* | XP_061397155.1 | AMP | 1 |
| *Musca vetustissima* | XP_061400438.1 | AMP | 0.9967 |
| *Musca vetustissima* | XP_061400439.1 | AMP | 0.9989 |
| *Musca vetustissima* | XP_061400440.1 | AMP | 0.9919 |
| *Musca vetustissima* | XP_061400445.1 | AMP | 0.9997 |
| *Neocloeon triangulifer* | XP_059490317.1 | AMP | 0.9964 |
| *Neodiprion fabricii* | XP_046429880.1 | AMP | 1 |
| *Neodiprion fabricii* | XP_046432265.1 | AMP | 0.9675 |
| *Neodiprion lecontei* | XP_015515023.1 | AMP | 1 |
| *Neodiprion lecontei* | XP_046599771.1 | AMP | 0.6328 |
| *Neodiprion pinetum* | XP_046489051.1 | AMP | 0.865 |
| *Neodiprion pinetum* | XP_046492441.1 | AMP | 1 |
| *Neodiprion virginianus* | XP_046626075.1 | AMP | 0.865 |
| *Neodiprion virginianus* | XP_046628271.1 | AMP | 1 |
| *Nicrophorus vespilloides* | XP_017768435.1 | AMP | 0.9826 |
| *Nicrophorus vespilloides* | XP_017768438.1 | AMP | 0.997 |
| *Nicrophorus vespilloides* | XP_017770657.1 | AMP | 0.9927 |
| *Nicrophorus vespilloides* | XP_017771952.1 | AMP | 0.9912 |
| *Nicrophorus vespilloides* | XP_017773019.1 | Non-AMP | 0.0137 |
| *Nicrophorus vespilloides* | Nves_Bit_LysC_348 | Non-AMP | 0.2689 |
| *Nilaparvata lugens* | XP_039289163.1 | AMP | 0.9928 |
| *Nymphalis io* | XP_050346632.1 | AMP | 0.9996 |
| *Nymphalis io* | Nio_Bit_LysC_351 | AMP | 0.9994 |
| *Ochlerotatus camptorhynchus* | XP_065080660.1 | AMP | 1 |
| *Ochlerotatus camptorhynchus* | XP_065082770.1 | AMP | 1 |
| *Onthophagus taurus* | Otau_Bit_LysC_356 | AMP | 0.9996 |
| *Onthophagus taurus* | Otau_Bit_LysC_357 | AMP | 0.9997 |
| *Onthophagus taurus* | Otau_Bit_LysC_358 | AMP | 0.9392 |
| *Onthophagus taurus* | Otau_Bit_LysC_359 | AMP | 0.9807 |
| *Onthophagus taurus* | Otau_Bit_LysC_360 | AMP | 0.9866 |
| *Onthophagus taurus* | Otau_Bit_LysC_361 | AMP | 0.9027 |
| *Ostrinia furnacalis* | XP_028156922.1 | AMP | 0.9609 |
| *Ostrinia furnacalis* | XP_028166301.1 | AMP | 0.9997 |
| *Ostrinia furnacalis* | XP_028169326.1 | AMP | 0.9994 |
| *Ostrinia furnacalis* | Ofur_Bit_LysC_364 | Non-AMP | 0.2249 |
| *Ostrinia nubilalis* | XP_063823050.1 | AMP | 0.9994 |
| *Ostrinia nubilalis* | XP_063827039.1 | AMP | 0.9993 |
| *Ostrinia nubilalis* | Onub_Bit_LysC_369 | Non-AMP | 0.2249 |
| *Papilio machaon* | XP_014369273.2 | AMP | 1 |
| *Papilio machaon* | XP_014369280.1 | AMP | 0.9999 |
| *Papilio machaon* | XP_045534718.1 | AMP | 0.9983 |
| *Papilio machaon* | Pmac_Bit_LysC_370 | AMP | 0.9967 |
| *Papilio polytes* | XP_013149139.1 | AMP | 0.9984 |
| *Papilio polytes* | XP_013149146.1 | AMP | 1 |
| *Papilio polytes* | XP_013149147.1 | AMP | 0.9999 |
| *Papilio polytes* | XP_013149253.1 | AMP | 0.9999 |
| *Papilio polytes* | Ppol_Bit_LysC_371 | AMP | 0.9898 |
| *Papilio xuthus* | XP_013180573.1 | AMP | 1 |
| *Papilio xuthus* | XP_013181578.1 | AMP | 0.9999 |
| *Papilio xuthus* | Pxut_Bit_LysC_372 | AMP | 0.9967 |
| *Papilio xuthus* | Pxut_Bit_LysC_373 | AMP | 1 |
| *Pararge aegeria* | XP_039751588.1 | AMP | 1 |
| *Pararge aegeria* | Paeg_Bit_LysC_374 | AMP | 0.9958 |
| *Parnassius apollo* | CAG4985632.1 | AMP | 0.8096 |
| *Parnassius apollo* | CAG4985635.1 | AMP | 0.9998 |
| *Parnassius apollo* | CAG4985638.1 | AMP | 0.9979 |
| *Parnassius apollo* | CAG4985641.1 | AMP | 0.9997 |
| *Parnassius apollo* | CAG4985643.1 | AMP | 0.9998 |
| *Parnassius apollo* | CAG4985646.1 | AMP | 0.9986 |
| *Parnassius apollo* | CAG4985650.1 | AMP | 1 |
| *Parnassius apollo* | Papo_Bit_LysC_376 | AMP | 0.9869 |
| *Pectinophora gossypiella* | XP_049868484.1 | AMP | 0.9886 |
| *Pectinophora gossypiella* | XP_049868495.1 | AMP | 0.9999 |
| *Pectinophora gossypiella* | XP_049871326.1 | AMP | 0.9818 |
| *Pectinophora gossypiella* | Pgos_Bit_LysC_377 | AMP | 1 |
| *Pectinophora gossypiella* | Pgos_Bit_LysC_379 | AMP | 0.8894 |
| *Pediculus humanus corporis* | XP_002431986.1 | AMP | 0.95 |
| *Periplaneta americana* | XP_069673831.1 | AMP | 1 |
| *Periplaneta americana* | XP_069673832.1 | AMP | 0.9532 |
| *Periplaneta americana* | XP_069681842.1 | AMP | 0.9999 |
| *Periplaneta americana* | XP_069681844.1 | AMP | 0.9849 |
| *Periplaneta americana* | XP_069700793.1 | AMP | 0.9719 |
| *Periplaneta americana* | XP_069702532.1 | AMP | 1 |
| *Periplaneta americana* | XP_069702533.1 | AMP | 1 |
| *Periplaneta americana* | XP_069704375.1 | AMP | 0.9999 |
| *Phlebotomus argentipes* | XP_059620889.1 | AMP | 0.9987 |
| *Phlebotomus argentipes* | XP_059621308.1 | AMP | 0.9993 |
| *Phlebotomus papatasi* | XP_055715754.1 | AMP | 0.9225 |
| *Phlebotomus papatasi* | XP_055715755.1 | AMP | 0.9979 |
| *Photinus pyralis* | XP_031356443.1* | AMP | 0.977 |
| *Pieris brassicae* | XP_045514986.1 | AMP | 1 |
| *Pieris brassicae* | XP_045515842.1 | AMP | 0.9999 |
| *Pieris brassicae* | Pbra_Bit_LysC_385 | AMP | 0.9664 |
| *Pieris napi* | XP_047507076.1 | AMP | 1 |
| *Pieris napi* | XP_047507357.1 | AMP | 0.9986 |
| *Pieris napi* | Pnap_Bit_LysC_386 | AMP | 0.979 |
| *Pieris rapae* | XP_022123928.2 | AMP | 0.9988 |
| *Pieris rapae* | XP_022123937.1 | AMP | 0.9999 |
| *Pieris rapae* | Prap_Bit_LysC_387 | AMP | 0.9642 |
| *Plodia interpunctella* | XP_053605608.1 | AMP | 0.9998 |
| *Plodia interpunctella* | XP_053612493.1 | AMP | 1 |
| *Plodia interpunctella* | Pint_Bit_LysC_389 | AMP | 0.9998 |
| *Plutella xylostella* | XP_011551067.3 | AMP | 0.9999 |
| *Plutella xylostella* | Pxyl_Bit_LysC_390 | AMP | 0.9915 |
| *Polypedilum vanderplanki* | KAG5671028.1 | AMP | 0.9997 |
| *Polypedilum vanderplanki* | KAG5671029.1 | AMP | 1 |
| *Polypedilum vanderplanki* | KAG5671030.1 | AMP | 0.9981 |
| *Polypedilum vanderplanki* | KAG5671031.1 | AMP | 0.9992 |
| *Polypedilum vanderplanki* | KAG5671032.1 | AMP | 0.9235 |
| *Polypedilum vanderplanki* | KAG5676352.1 | AMP | 0.9998 |
| *Procambarus clarkii* | XP_045588443.1 | AMP | 0.9994 |
| *Procambarus clarkii* | XP_045593091.1 | AMP | 0.9999 |
| *Procambarus clarkii* | XP_045593101.1 | AMP | 0.9467 |
| *Procambarus clarkii* | XP_045593103.1 | AMP | 0.5084 |
| *Procambarus clarkii* | XP_045596313.1 | AMP | 0.9972 |
| *Procambarus clarkii* | XP_045608560.1 | AMP | 0.9966 |
| *Procambarus clarkii* | Pcla_Bit_LysC_394 | AMP | 0.9978 |
| *Procambarus clarkii* | Pcla_Bit_LysC_395 | AMP | 0.9803 |
| *Rhodnius prolixus* | RPRC015440 | AMP | 0.9997 |
| *Rhodnius prolixus* | RPRC015441 | AMP | 0.9996 |
| *Rhodnius prolixus* | RPRC015442 | AMP | 0.9998 |
| *Rhynchophorus ferrugineus* | KAF7263020.1 | AMP | 0.8651 |
| *Rhynchophorus ferrugineus* | KAF7266659.1 | AMP | 0.9997 |
| *Rhynchophorus ferrugineus* | KAF7266661.1 | AMP | 0.9908 |
| *Rhynchophorus ferrugineus* | Rfer_Bit_LysC_396 | AMP | 0.9817 |
| *Sabethes cyaneus* | XP_053684417.1 | AMP | 1 |
| *Sabethes cyaneus* | XP_053689352.1 | AMP | 1 |
| *Sabethes cyaneus* | Scya_Bit_LysC_399 | AMP | 1 |
| *Scaptodrosophila lebanonensis* | XP_030374009.1 | AMP | 0.9422 |
| *Scaptodrosophila lebanonensis* | XP_030375504.1 | AMP | 0.9997 |
| *Scaptodrosophila lebanonensis* | XP_030375505.1 | AMP | 0.9996 |
| *Scaptodrosophila lebanonensis* | XP_030375506.1 | AMP | 0.9996 |
| *Scaptodrosophila lebanonensis* | XP_030375508.1 | AMP | 0.9999 |
| *Scaptodrosophila lebanonensis* | XP_030375509.1 | AMP | 0.9998 |
| *Scaptodrosophila lebanonensis* | XP_030375510.1 | AMP | 0.9998 |
| *Scaptodrosophila lebanonensis* | XP_030375512.1 | AMP | 0.9998 |
| *Scaptodrosophila lebanonensis* | XP_030375513.1 | AMP | 0.9998 |
| *Scaptodrosophila lebanonensis* | XP_030375514.1 | AMP | 0.9998 |
| *Scaptodrosophila lebanonensis* | XP_030375515.1 | AMP | 0.9998 |
| *Scaptodrosophila lebanonensis* | XP_030375516.1 | AMP | 0.9998 |
| *Scaptodrosophila lebanonensis* | XP_030375517.1 | AMP | 0.9998 |
| *Scaptodrosophila lebanonensis* | XP_030387648.1 | AMP | 0.988 |
| *Scaptodrosophila lebanonensis* | XP_030388214.1 | AMP | 1 |
| *Scylla paramamosain* | XP_063881396.1* | AMP | 0.9999 |
| *Sitophilus oryzae* | Sory_Bit_LysC_413 | Non-AMP | 0.0002 |
| *Spodoptera frugiperda* | XP_035433980.2 | AMP | 0.9997 |
| *Spodoptera frugiperda* | XP_035434307.1 | AMP | 0.9987 |
| *Spodoptera frugiperda* | XP_035441310.1 | AMP | 0.999 |
| *Spodoptera frugiperda* | Sfru_Bit_LysC_414 | AMP | 0.7006 |
| *Spodoptera litura* | XP_022814833.1 | AMP | 0.9988 |
| *Spodoptera litura* | XP_022814852.1 | AMP | 0.999 |
| *Spodoptera litura* | XP_022834886.1 | AMP | 0.9992 |
| *Spodoptera litura* | Slit_Bit_LysC_417 | AMP | 0.7825 |
| *Stomoxys calcitrans* | XP_013113128.1 | AMP | 0.9999 |
| *Stomoxys calcitrans* | XP_013113129.1 | AMP | 0.9998 |
| *Stomoxys calcitrans* | XP_013113136.1 | AMP | 0.9997 |
| *Stomoxys calcitrans* | XP_013113137.1 | AMP | 0.9972 |
| *Stomoxys calcitrans* | XP_013113138.1 | AMP | 0.9993 |
| *Stomoxys calcitrans* | XP_013113139.1 | AMP | 0.9972 |
| *Stomoxys calcitrans* | XP_013113140.1 | AMP | 0.9997 |
| *Stomoxys calcitrans* | XP_013113141.1 | AMP | 0.9997 |
| *Stomoxys calcitrans* | XP_013114848.2 | AMP | 0.9302 |
| *Stomoxys calcitrans* | XP_013114849.2 | AMP | 0.9999 |
| *Stomoxys calcitrans* | XP_013114850.2 | AMP | 0.9993 |
| *Stomoxys calcitrans* | XP_013114853.1 | AMP | 0.9993 |
| *Stomoxys calcitrans* | XP_013117897.1 | AMP | 0.9996 |
| *Stomoxys calcitrans* | XP_013117994.1 | AMP | 0.9829 |
| *Stomoxys calcitrans* | XP_059224512.1 | AMP | 0.9993 |
| *Stomoxys calcitrans* | XP_059224513.1 | AMP | 0.9993 |
| *Stomoxys calcitrans* | XP_059224514.1 | AMP | 0.9993 |
| *Stomoxys calcitrans* | XP_059224515.1 | AMP | 0.9993 |
| *Stomoxys calcitrans* | XP_059226474.1 | AMP | 0.9998 |
| *Stomoxys calcitrans* | XP_059226475.1 | AMP | 0.9999 |
| *Stomoxys calcitrans* | XP_059226476.1 | AMP | 0.9998 |
| *Stomoxys calcitrans* | XP_059226477.1 | AMP | 0.9999 |
| *Stomoxys calcitrans* | XP_059226478.1 | AMP | 0.9998 |
| *Stomoxys calcitrans* | Scal_Bit_LysC_420 | AMP | 0.9981 |
| *Tenebrio molitor* | Tmol_Bit_LysC_423 | AMP | 0.8897 |
| *Tenebrio molitor* | Tmol_Bit_LysC_424 | AMP | 0.9378 |
| *Thrips palmi* | XP_034230753.1 | AMP | 0.9993 |
| *Thrips palmi* | XP_034235752.1 | AMP | 0.9995 |
| *Thrips palmi* | XP_034255300.1 | AMP | 0.9995 |
| *Thrips palmi* | XP_034255301.1 | AMP | 0.9992 |
| *Thrips palmi* | XP_034255541.1 | AMP | 1 |
| *Thrips palmi* | XP_034255542.1 | AMP | 0.9999 |
| *Topomyia yanbarensis* | XP_058837208.1 | AMP | 1 |
| *Topomyia yanbarensis* | XP_058837210.1 | AMP | 1 |
| *Topomyia yanbarensis* | XP_058837211.1 | AMP | 1 |
| *Toxorhynchites rutilus septentrionalis* | XP_055619622.1 | AMP | 1 |
| *Toxorhynchites rutilus septentrionalis* | XP_055619623.1 | AMP | 1 |
| *Tribolium castaneum* | Tcas_Bit_LysC_431 | AMP | 0.9989 |
| *Tribolium castaneum* | Tcas_Bit_LysC_432 | AMP | 0.9504 |
| *Tribolium castaneum* | Tcas_Bit_LysC_433 | AMP | 0.9995 |
| *Tribolium castaneum* | Tcas_Bit_LysC_434 | AMP | 0.9953 |
| *Tribolium madens* | XP_044258630.1 | AMP | 0.9999 |
| *Trichoplusia ni* | XP_026731948.1 | AMP | 0.9998 |
| *Trichoplusia ni* | XP_026732891.1 | AMP | 1 |
| *Trichoplusia ni* | Tni_Bit_LysC_436 | AMP | 0.8687 |
| *Uranotaenia lowii* | XP_055602141.1 | AMP | 1 |
| *Uranotaenia lowii* | XP_055602142.1 | AMP | 1 |
| *Uranotaenia lowii* | XP_055608009.1 | AMP | 1 |
| *Vanessa atalanta* | XP_047530675.1 | AMP | 1 |
| *Vanessa atalanta* | Vata_Bit_LysC_442 | AMP | 0.9997 |
| *Vanessa cardui* | XP_046962766.1 | AMP | 0.9999 |
| *Vanessa cardui* | Vcar_Bit_LysC_443 | AMP | 0.9995 |
| *Vanessa tameamea* | XP_026484424.1 | AMP | 1 |
| *Vanessa tameamea* | Vtam_Bit_LysC_445 | AMP | 0.9995 |
| *Wyeomyia smithii* | XP_055529484.1 | AMP | 1 |
| *Wyeomyia smithii* | XP_055529486.1 | AMP | 1 |
| *Wyeomyia smithii* | XP_055532641.1 | AMP | 1 |
| *Zerene cesonia* | XP_038214847.1 | AMP | 0.9999 |
| *Zerene cesonia* | XP_038214848.1 | AMP | 1 |
| *Zerene cesonia* | Zces_Bit_LysC_452 | AMP | 0.9986 |
| *Zeugodacus cucurbitae* | XP_011180840.2 | AMP | 0.5194 |
| *Zeugodacus cucurbitae* | XP_011180841.2 | AMP | 1 |
| *Zeugodacus cucurbitae* | XP_011180844.2 | AMP | 0.9999 |
| *Zeugodacus cucurbitae* | XP_011180845.2 | AMP | 0.9478 |
| *Zeugodacus cucurbitae* | XP_011180848.2 | AMP | 0.9478 |
| *Zeugodacus cucurbitae* | XP_011180851.1 | AMP | 0.9601 |
| *Zeugodacus cucurbitae* | XP_011185402.2 | AMP | 0.9998 |
| *Zeugodacus cucurbitae* | XP_011185403.1 | AMP | 0.9998 |
| *Zeugodacus cucurbitae* | XP_011185404.1 | AMP | 0.9998 |
| *Zeugodacus cucurbitae* | XP_028895454.2 | AMP | 0.9978 |
| *Zeugodacus cucurbitae* | XP_054088797.1 | AMP | 0.9601 |
| *Zeugodacus cucurbitae* | XP_054088799.1 | AMP | 0.9478 |
| *Zeugodacus cucurbitae* | Zcuc_Bit_LysC_453 | AMP | 1 |
| *Zeugodacus cucurbitae* | Zcuc_Bit_LysC_454 | AMP | 0.9999 |
| *Zootermopsis nevadensis* | XP_021915816.1 | AMP | 0.8272 |
| *Zootermopsis nevadensis* | XP_021915960.1 | AMP | 0.9999 |
| *Zootermopsis nevadensis* | XP_021924973.1 | AMP | 1 |
| *Zophobas morio* | XP_063904723.1 | AMP | 0.9978 |
| *Zophobas morio* | XP_063920227.1 | AMP | 0.6676 |
| *Zophobas morio* | XP_063920752.1 | AMP | 0.9996 |
| *Zophobas morio* | Zmor_Bit_LysC_459 | AMP | 0.613 |
| *Abscondita terminalis* | Ater_Bit_LysC_1 | Non-AMP | 0.1369 |
| *Aedes aegypti* | Aaeg_Bit_LysC_6 | AMP | 0.9943 |
| *Aedes aegypti* | Aaeg_Bit_LysC_8 | AMP | 0.9999 |
| *Aedes aegypti* | Aaeg_Bit_LysC_9 | AMP | 0.9745 |
| *Aedes albopictus* | Aalb_Bit_LysC_10 | AMP | 0.9976 |
| *Aedes albopictus* | Aalb_Bit_LysC_11 | AMP | 0.9911 |
| *Aedes albopictus* | Aalb_Bit_LysC_12* | AMP | 0.9944 |
| *Amyelois transitella* | XP_013193987.2 | AMP | 0.9999 |
| *Anastrepha ludens* | XP_053963940.1 | AMP | 0.5905 |
| *Anastrepha ludens* | XP_053966452.1 | AMP | 0.9996 |
| *Anastrepha ludens* | Alud_Bit_LysC_22 | AMP | 0.9357 |
| *Anastrepha ludens* | Alud_Bit_LysC_23 | Non-AMP | 0.1479 |
| *Anastrepha obliqua* | XP_054741227.1 | AMP | 0.5905 |
| *Anastrepha obliqua* | Aobl_Bit_LysC_24 | AMP | 0.9424 |
| *Anastrepha obliqua* | Aobl_Bit_LysC_27 | Non-AMP | 0.1826 |
| *Anopheles albimanus* | Aalb_Bit_LysC_28 | AMP | 0.9733 |
| *Anopheles albimanus* | Aalb_Bit_LysC_29* | AMP | 0.9586 |
| *Anopheles aquasalis* | Aaqu_Bit_LysC_30 | AMP | 0.7313 |
| *Anopheles aquasalis* | Aaqu_Bit_LysC_31* | AMP | 0.9966 |
| *Anopheles arabiensis* | Aara_Bit_LysC_32 | AMP | 0.9999 |
| *Anopheles arabiensis* | Aara_Bit_LysC_35 | AMP | 0.8713 |
| *Anopheles coluzzii* | Acol_Bit_LysC_37 | AMP | 0.8801 |
| *Anopheles darlingi* | Adar_Bit_LysC_41* | AMP | 0.7657 |
| *Anopheles funestus* | Afun_Bit_LysC_43 | AMP | 0.9492 |
| *Anopheles funestus* | Afun_Bit_LysC_45 | AMP | 0.8295 |
| *Anopheles funestus* | Afun_Bit_LysC_46 | AMP | 0.9964 |
| *Anopheles gambiae* | Agam_Bit_LysC_48 | AMP | 0.8065 |
| *Anopheles maculipalpis* | Amac_Bit_LysC_51 | AMP | 0.9942 |
| *Anopheles maculipalpis* | Amac_Bit_LysC_52 | AMP | 0.9902 |
| *Anopheles maculipalpis* | Amac_Bit_LysC_53 | AMP | 0.9904 |
| *Anopheles maculipalpis* | Amac_Bit_LysC_54 | AMP | 0.9663 |
| *Anopheles merus* | Amer_Bit_LysC_55 | AMP | 0.9046 |
| *Anopheles moucheti* | Amou_Bit_LysC_57 | Non-AMP | 0.3262 |
| *Anopheles moucheti* | Amou_Bit_LysC_58 | AMP | 0.9933 |
| *Anopheles moucheti* | Amou_Bit_LysC_59 | AMP | 0.9627 |
| *Anopheles stephensi* | Aste_Bit_LysC_61 | AMP | 0.9691 |
| *Anopheles stephensi* | Aste_Bit_LysC_62 | AMP | 0.9848 |
| *Anopheles stephensi* | Aste_Bit_LysC_63 | AMP | 0.9915 |
| *Anthonomus grandis grandis* | Agra_Bit_LysC_69 | AMP | 0.8833 |
| *Armigeres subalbatus* | Asub_Bit_LysC_75 | AMP | 0.9995 |
| *Armigeres subalbatus* | Asub_Bit_LysC_76* | AMP | 0.9897 |
| *Armigeres subalbatus* | Asub_Bit_LysC_78 | AMP | 0.9851 |
| *Bactrocera dorsalis* | XP_049306979.1 | AMP | 0.835 |
| *Bactrocera dorsalis* | Bdor_Bit_LysC_80 | AMP | 0.9747 |
| *Bactrocera dorsalis* | Bdor_Bit_LysC_81 | AMP | 0.9412 |
| *Bactrocera latifrons* | XP_018793567.1* | AMP | 0.8369 |
| *Bactrocera latifrons* | Blat_Bit_LysC_83 | AMP | 0.8334 |
| *Bactrocera latifrons* | Blat_Bit_LysC_84 | Non-AMP | 0.0614 |
| *Bactrocera latifrons* | Blat_Bit_LysC_87 | AMP | 0.813 |
| *Bactrocera latifrons* | Blat_Bit_LysC_90 | AMP | 0.9875 |
| *Bactrocera tryoni* | XP_039957829.1* | AMP | 0.5844 |
| *Bactrocera tryoni* | Btry_Bit_LysC_91 | AMP | 0.5067 |
| *Bactrocera tryoni* | Btry_Bit_LysC_95 | AMP | 0.9412 |
| *Bactrocera tryoni* | Btry_Bit_LysC_96 | Non-AMP | 0.0643 |
| *Blattella germanica* | PSN54899.1 | AMP | 0.9999 |
| *Blattella germanica* | Bger_Bit_LysC_99 | AMP | 0.9943 |
| *Bradysia coprophila* | Bcop_Bit_LysC_102 | Non-AMP | 0.414 |
| *Bradysia coprophila* | Bcop_Bit_LysC_103 | AMP | 0.9218 |
| *Calliphora vicina* | XP_065368584.1 | AMP | 0.8016 |
| *Calliphora vicina* | Cvic_Bit_LysC_107 | AMP | 0.9927 |
| *Calliphora vicina* | Cvic_Bit_LysC_108 | AMP | 0.9971 |
| *Calliphora vicina* | Cvic_Bit_LysC_109 | AMP | 0.9145 |
| *Calliphora vicina* | Cvic_Bit_LysC_110 | AMP | 0.9998 |
| *Calliphora vicina* | Cvic_Bit_LysC_111 | AMP | 0.9723 |
| *Calliphora vicina* | Cvic_Bit_LysC_112 | AMP | 0.9884 |
| *Calliphora vicina* | Cvic_Bit_LysC_113 | AMP | 0.964 |
| *Ceratitis capitata* | XP_012155935.1 | AMP | 0.7632 |
| *Ceratitis capitata* | Ccap_Bit_LysC_114 | AMP | 0.9399 |
| *Ceratitis capitata* | Ccap_Bit_LysC_115 | Non-AMP | 0.2976 |
| *Ceratitis capitata* | Ccap_Bit_LysC_116 | Non-AMP | 0.2697 |
| *Ceratitis capitata* | Ccap_Bit_LysC_117 | Non-AMP | 0.3199 |
| *Clunio marinus* | CRL05170.1 | AMP | 0.9999 |
| *Clunio marinus* | Cmar_Bit_LysC_125 | Non-AMP | 0.4096 |
| *Clunio marinus* | Cmar_Bit_LysC_126 | AMP | 0.9054 |
| *Condylostylus longicornis* | Clon_Bit_LysC_131* | AMP | 0.9818 |
| *Contarinia nasturtii* | Cnas_Bit_LysC_133 | AMP | 0.9727 |
| *Contarinia nasturtii* | Cnas_Bit_LysC_134 | Non-AMP | 0.4426 |
| *Contarinia nasturtii* | Cnas_Bit_LysC_135 | AMP | 0.9973 |
| *Culex pipiens pallens* | Cpip_Bit_LysC_138 | AMP | 0.9936 |
| *Culex quinquefasciatus* | Cqui_Bit_LysC_140 | AMP | 0.9997 |
| *Culex quinquefasciatus* | Cqui_Bit_LysC_141 | AMP | 0.9942 |
| *Culicoides brevitarsis* | Cbre_Bit_LysC_142 | Non-AMP | 0.473 |
| *Diabrotica virgifera virgifera* | Dvir_Bit_LysC_155 | Non-AMP | 0.0363 |
| *Diabrotica virgifera virgifera* | Dvir_Bit_LysC_156 | AMP | 0.9869 |
| *Diorhabda sublineata* | Dsub_Bit_LysC_157 | Non-AMP | 0.1023 |
| *Diorhabda sublineata* | Dsub_Bit_LysC_158 | AMP | 0.7638 |
| *Drosophila albomicans* | Dalb_Bit_LysC_160 | Non-AMP | 0.3816 |
| *Drosophila albomicans* | Dalb_Bit_LysC_161 | Non-AMP | 0.231 |
| *Drosophila albomicans* | Dalb_Bit_LysC_162 | AMP | 0.7559 |
| *Drosophila albomicans* | Dalb_Bit_LysC_163 | AMP | 0.6426 |
| *Drosophila ananassae* | XP_032310747.1 | AMP | 0.9999 |
| *Drosophila ananassae* | Dana_Bit_LysC_164 | Non-AMP | 0.0186 |
| *Drosophila ananassae* | Dana_Bit_LysC_165 | AMP | 0.9098 |
| *Drosophila ananassae* | Dana_Bit_LysC_166 | Non-AMP | 0.0115 |
| *Drosophila arizonae* | XP_017860836.1 | AMP | 0.9996 |
| *Drosophila arizonae* | Dari_Bit_LysC_167 | Non-AMP | 0.1528 |
| *Drosophila arizonae* | Dari_Bit_LysC_169 | Non-AMP | 0.4333 |
| *Drosophila arizonae* | Dari_Bit_LysC_170 | Non-AMP | 0.011 |
| *Drosophila arizonae* | Dari_Bit_LysC_171 | AMP | 0.8992 |
| *Drosophila biarmipes* | XP_016957504.1 | Non-AMP | 0.1616 |
| *Drosophila biarmipes* | Dbia_Bit_LysC_172 | AMP | 0.9994 |
| *Drosophila biarmipes* | Dbia_Bit_LysC_173 | Non-AMP | 0.1551 |
| *Drosophila biarmipes* | Dbia_Bit_LysC_174 | Non-AMP | 0.0177 |
| *Drosophila bipectinata* | XP_017090724.2 | AMP | 0.9211 |
| *Drosophila bipectinata* | Dbip_Bit_LysC_175 | AMP | 0.9043 |
| *Drosophila bipectinata* | Dbip_Bit_LysC_176 | Non-AMP | 0.4127 |
| *Drosophila bipectinata* | Dbip_Bit_LysC_177 | Non-AMP | 0.0115 |
| *Drosophila elegans* | Dele_Bit_LysC_178 | Non-AMP | 0.0105 |
| *Drosophila elegans* | Dele_Bit_LysC_179 | AMP | 0.9205 |
| *Drosophila elegans* | Dele_Bit_LysC_180 | Non-AMP | 0.2988 |
| *Drosophila erecta* | Dere_Bit_LysC_181 | Non-AMP | 0.1281 |
| *Drosophila erecta* | Dere_Bit_LysC_182 | AMP | 0.9062 |
| *Drosophila erecta* | Dere_Bit_LysC_183 | Non-AMP | 0.0135 |
| *Drosophila eugracilis* | Deug_Bit_LysC_184 | Non-AMP | 0.1024 |
| *Drosophila eugracilis* | Deug_Bit_LysC_185 | Non-AMP | 0.0093 |
| *Drosophila eugracilis* | Deug_Bit_LysC_186 | AMP | 0.936 |
| *Drosophila ficusphila* | Dfic_Bit_LysC_187 | AMP | 0.9982 |
| *Drosophila ficusphila* | Dfic_Bit_LysC_188 | Non-AMP | 0.4617 |
| *Drosophila ficusphila* | Dfic_Bit_LysC_190 | Non-AMP | 0.005 |
| *Drosophila grimshawi* | Dgri_Bit_LysC_191 | Non-AMP | 0.1002 |
| *Drosophila grimshawi* | Dgri_Bit_LysC_192 | AMP | 0.9787 |
| *Drosophila grimshawi* | Dgri_Bit_LysC_193 | Non-AMP | 0.1418 |
| *Drosophila grimshawi* | Dgri_Bit_LysC_194 | Non-AMP | 0.0067 |
| *Drosophila guanche* | Dgua_Bit_LysC_195 | Non-AMP | 0.1762 |
| *Drosophila guanche* | Dgua_Bit_LysC_196 | AMP | 0.8811 |
| *Drosophila guanche* | Dgua_Bit_LysC_197 | AMP | 0.8366 |
| *Drosophila hydei* | Dhyd_Bit_LysC_198 | AMP | 0.8926 |
| *Drosophila hydei* | Dhyd_Bit_LysC_199 | Non-AMP | 0.1829 |
| *Drosophila hydei* | Dhyd_Bit_LysC_200 | Non-AMP | 0.1461 |
| *Drosophila hydei* | Dhyd_Bit_LysC_202 | Non-AMP | 0.0036 |
| *Drosophila kikkawai* | XP_017016924.1 | AMP | 0.9927 |
| *Drosophila kikkawai* | Dkik_Bit_LysC_204 | Non-AMP | 0.0057 |
| *Drosophila kikkawai* | Dkik_Bit_LysC_205 | AMP | 0.5355 |
| *Drosophila kikkawai* | Dkik_Bit_LysC_207 | AMP | 0.9399 |
| *Drosophila mauritiana* | Dmau_Bit_LysC_208 | Non-AMP | 0.0843 |
| *Drosophila mauritiana* | Dmau_Bit_LysC_209 | Non-AMP | 0.0201 |
| *Drosophila mauritiana* | Dmau_Bit_LysC_210 | AMP | 0.8838 |
| *Drosophila melanogaster* | Dmel_Bit_LysC_211 | Non-AMP | 0.0515 |
| *Drosophila melanogaster* | Dmel_Bit_LysC_212 | AMP | 0.9204 |
| *Drosophila melanogaster* | Dmel_Bit_LysC_213 | Non-AMP | 0.0924 |
| *Drosophila miranda* | XP_017148190.1 | AMP | 0.9977 |
| *Drosophila miranda* | Dmir_Bit_LysC_214 | AMP | 0.902 |
| *Drosophila miranda* | Dmir_Bit_LysC_215 | Non-AMP | 0.008 |
| *Drosophila miranda* | Dmir_Bit_LysC_216 | Non-AMP | 0.0037 |
| *Drosophila mojavensis* | Dmoj_Bit_LysC_217 | AMP | 0.8759 |
| *Drosophila mojavensis* | Dmoj_Bit_LysC_218 | Non-AMP | 0.3043 |
| *Drosophila mojavensis* | Dmoj_Bit_LysC_219 | Non-AMP | 0.1528 |
| *Drosophila mojavensis* | Dmoj_Bit_LysC_220 | Non-AMP | 0.0172 |
| *Drosophila montana* | Dmon_Bit_LysC_221 | AMP | 0.8414 |
| *Drosophila montana* | Dmon_Bit_LysC_222 | Non-AMP | 0.0376 |
| *Drosophila montana* | Dmon_Bit_LysC_223 | Non-AMP | 0.0132 |
| *Drosophila nasuta* | Dnas_Bit_LysC_224 | AMP | 0.6749 |
| *Drosophila nasuta* | Dnas_Bit_LysC_225 | Non-AMP | 0.1648 |
| *Drosophila navojoa* | Dnav_Bit_LysC_226 | Non-AMP | 0.011 |
| *Drosophila navojoa* | Dnav_Bit_LysC_227 | AMP | 0.8759 |
| *Drosophila navojoa* | Dnav_Bit_LysC_228 | Non-AMP | 0.1212 |
| *Drosophila novamexicana* | Dnov_Bit_LysC_229 | Non-AMP | 0.1786 |
| *Drosophila novamexicana* | Dnov_Bit_LysC_230 | AMP | 0.8446 |
| *Drosophila novamexicana* | Dnov_Bit_LysC_231 | Non-AMP | 0.0425 |
| *Drosophila novamexicana* | Dnov_Bit_LysC_232 | Non-AMP | 0.0184 |
| *Drosophila obscura* | Dobs_Bit_LysC_233 | AMP | 0.8243 |
| *Drosophila obscura* | Dobs_Bit_LysC_234 | AMP | 0.9148 |
| *Drosophila persimilis* | XP_026841034.1 | AMP | 0.9842 |
| *Drosophila persimilis* | Dper_Bit_LysC_235 | Non-AMP | 0.0033 |
| *Drosophila persimilis* | Dper_Bit_LysC_236 | AMP | 0.8284 |
| *Drosophila persimilis* | Dper_Bit_LysC_237 | AMP | 0.902 |
| *Drosophila pseudoobscura* | Dpse_Bit_LysC_238 | Non-AMP | 0.0026 |
| *Drosophila pseudoobscura* | Dpse_Bit_LysC_239 | AMP | 0.813 |
| *Drosophila pseudoobscura* | Dpse_Bit_LysC_240 | AMP | 0.902 |
| *Drosophila rhopaloa* | Drho_Bit_LysC_241 | AMP | 0.9984 |
| *Drosophila rhopaloa* | Drho_Bit_LysC_242 | Non-AMP | 0.01 |
| *Drosophila rhopaloa* | Drho_Bit_LysC_243 | Non-AMP | 0.323 |
| *Drosophila sechellia* | XP_032572413.1 | Non-AMP | 0.3187 |
| *Drosophila sechellia* | Dsec_Bit_LysC_244 | Non-AMP | 0.1131 |
| *Drosophila sechellia* | Dsec_Bit_LysC_245 | AMP | 0.8908 |
| *Drosophila sechellia* | Dsec_Bit_LysC_246 | Non-AMP | 0.0265 |
| *Drosophila serrata* | Dser_Bit_LysC_247 | AMP | 0.9723 |
| *Drosophila serrata* | Dser_Bit_LysC_248 | Non-AMP | 0.2953 |
| *Drosophila serrata* | Dser_Bit_LysC_249 | Non-AMP | 0.0164 |
| *Drosophila serrata* | Dser_Bit_LysC_250 | AMP | 0.6008 |
| *Drosophila serrata* | Dser_Bit_LysC_251 | AMP | 0.9124 |
| *Drosophila serrata* | Dser_Bit_LysC_252 | Non-AMP | 0.0136 |
| *Drosophila simulans* | XP_016027391.1 | AMP | 0.6142 |
| *Drosophila simulans* | Dsim_Bit_LysC_253 | AMP | 0.8953 |
| *Drosophila simulans* | Dsim_Bit_LysC_254 | Non-AMP | 0.019 |
| *Drosophila simulans* | Dsim_Bit_LysC_255 | Non-AMP | 0.1129 |
| *Drosophila subobscura* | Dsub_Bit_LysC_256 | Non-AMP | 0.1762 |
| *Drosophila subobscura* | Dsub_Bit_LysC_257 | AMP | 0.8811 |
| *Drosophila subobscura* | Dsub_Bit_LysC_258 | Non-AMP | 0.2094 |
| *Drosophila subpulchrella* | XP_037713180.1 | Non-AMP | 0.2604 |
| *Drosophila subpulchrella* | Dsub_Bit_LysC_259 | Non-AMP | 0.2499 |
| *Drosophila subpulchrella* | Dsub_Bit_LysC_260 | Non-AMP | 0.0357 |
| *Drosophila subpulchrella* | Dsub_Bit_LysC_261 | AMP | 0.9933 |
| *Drosophila sulfurigaster albostrigata* | Dsul_Bit_LysC_262 | AMP | 0.6749 |
| *Drosophila sulfurigaster albostrigata* | Dsul_Bit_LysC_263 | Non-AMP | 0.231 |
| *Drosophila sulfurigaster albostrigata* | Dsul_Bit_LysC_264 | Non-AMP | 0.0303 |
| *Drosophila sulfurigaster albostrigata* | Dsul_Bit_LysC_265 | AMP | 0.6031 |
| *Drosophila suzukii* | XP_016943152.1 | Non-AMP | 0.209 |
| *Drosophila suzukii* | Dsuz_Bit_LysC_266 | Non-AMP | 0.0138 |
| *Drosophila suzukii* | Dsuz_Bit_LysC_267 | AMP | 0.9933 |
| *Drosophila suzukii* | Dsuz_Bit_LysC_268 | Non-AMP | 0.3189 |
| *Drosophila takahashii* | XP_017001034.2 | AMP | 0.5529 |
| *Drosophila takahashii* | Dtak_Bit_LysC_269 | Non-AMP | 0.0988 |
| *Drosophila takahashii* | Dtak_Bit_LysC_273 | Non-AMP | 0.0145 |
| *Drosophila takahashii* | Dtak_Bit_LysC_274 | AMP | 0.9862 |
| *Drosophila teissieri* | XP_043645872.1 | AMP | 0.7701 |
| *Drosophila teissieri* | Dtei_Bit_LysC_275 | Non-AMP | 0.0087 |
| *Drosophila teissieri* | Dtei_Bit_LysC_276 | Non-AMP | 0.1046 |
| *Drosophila teissieri* | Dtei_Bit_LysC_279 | AMP | 0.9193 |
| *Drosophila virilis* | Dvir_Bit_LysC_280 | Non-AMP | 0.0662 |
| *Drosophila virilis* | Dvir_Bit_LysC_281 | AMP | 0.804 |
| *Drosophila virilis* | Dvir_Bit_LysC_282 | Non-AMP | 0.0065 |
| *Drosophila yakuba* | XP_015052851.1 | Non-AMP | 0.3642 |
| *Drosophila yakuba* | Dyak_Bit_LysC_283 | Non-AMP | 0.0112 |
| *Drosophila yakuba* | Dyak_Bit_LysC_284 | AMP | 0.9007 |
| *Drosophila yakuba* | Dyak_Bit_LysC_285 | Non-AMP | 0.1046 |
| *Episyrphus balteatus* | Ebal_Bit_LysC_286 | Non-AMP | 0.0829 |
| *Episyrphus balteatus* | Ebal_Bit_LysC_288 | AMP | 0.9659 |
| *Episyrphus balteatus* | Ebal_Bit_LysC_289 | Non-AMP | 0.1131 |
| *Eupeodes corollae* | XP_055910463.1 | AMP | 0.9998 |
| *Eupeodes corollae* | Ecor_Bit_LysC_290 | AMP | 0.9383 |
| *Eupeodes corollae* | Ecor_Bit_LysC_292 | Non-AMP | 0.0839 |
| *Eupeodes corollae* | Ecor_Bit_LysC_293 | AMP | 0.9447 |
| *Frankliniella occidentalis* | XP_026289145.2 | AMP | 0.9685 |
| *Frankliniella occidentalis* | Focc_Bit_LysC_295 | AMP | 0.9915 |
| *Galleria mellonella* | Gmel_Bit_LysC_298 | AMP | 0.98 |
| *Gonioctena quinquepunctata* | Gqui_Bit_LysC_299 | AMP | 0.9982 |
| *Gonioctena quinquepunctata* | Gqui_Bit_LysC_300 | AMP | 0.9996 |
| *Hermetia illucens* | XP_037915162.1 | AMP | 1 |
| *Hermetia illucens* | XP_037915168.1 | AMP | 1 |
| *Hermetia illucens* | XP_037915169.1 | AMP | 1 |
| *Hermetia illucens* | XP_037915172.1 | AMP | 1 |
| *Hermetia illucens* | XP_037915173.1 | AMP | 1 |
| *Hermetia illucens* | XP_037915176.1 | AMP | 1 |
| *Hermetia illucens* | XP_037915177.1 | AMP | 1 |
| *Hermetia illucens* | XP_037915178.1 | AMP | 1 |
| *Hermetia illucens* | XP_037915179.1 | AMP | 1 |
| *Hermetia illucens* | XP_037915180.1 | AMP | 1 |
| *Hermetia illucens* | XP_037915181.1 | AMP | 1 |
| *Hermetia illucens* | XP_037915182.1 | AMP | 1 |
| *Hermetia illucens* | XP_037915183.1 | AMP | 1 |
| *Hermetia illucens* | XP_037915184.1 | AMP | 1 |
| *Hermetia illucens* | XP_037915185.1 | AMP | 1 |
| *Hermetia illucens* | XP_037915187.1 | AMP | 1 |
| *Hermetia illucens* | Hill_Bit_LysC_305 | Non-AMP | 0.49 |
| *Hermetia illucens* | Hill_Bit_LysC_306* | AMP | 0.9198 |
| *Hermetia illucens* | Hill_Bit_LysC_308 | AMP | 0.8631 |
| *Hermetia illucens* | Hill_Bit_LysC_309 | AMP | 0.926 |
| *Hetaerina americana* | XP_071452927.1* | AMP | 0.9991 |
| *Hyposmocoma kahamanoa* | Hkah_Bit_LysC_314 | AMP | 0.9984 |
| *Ischnura elegans* | XP_046385445.1 | AMP | 0.9995 |
| *Leptinotarsa decemlineata* | Ldec_Bit_LysC_318 | AMP | 0.9948 |
| *Lucilia cuprina* | XP_023296951.2 | AMP | 0.9553 |
| *Lucilia cuprina* | Lcup_Bit_LysC_320 | AMP | 0.9938 |
| *Lucilia cuprina* | Lcup_Bit_LysC_321 | AMP | 0.8715 |
| *Lucilia cuprina* | Lcup_Bit_LysC_325 | Non-AMP | 0.1102 |
| *Lucilia cuprina* | Lcup_Bit_LysC_328 | AMP | 0.9994 |
| *Lucilia cuprina* | Lcup_Bit_LysC_329 | AMP | 0.9749 |
| *Lucilia sericata* | XP_037813955.1 | AMP | 0.9594 |
| *Lucilia sericata* | Lser_Bit_LysC_330 | AMP | 0.9943 |
| *Lutzomyia longipalpis* | Llon_Bit_LysC_332 | AMP | 0.9717 |
| *Malaya genurostris* | Mgen_Bit_LysC_334 | AMP | 0.9707 |
| *Malaya genurostris* | Mgen_Bit_LysC_335 | AMP | 0.9988 |
| *Musca domestica* | XP_058984770.1 | AMP | 0.8657 |
| *Musca domestica* | Mdom_Bit_LysC_342 | AMP | 0.9973 |
| *Musca domestica* | Mdom_Bit_LysC_343 | Non-AMP | 0.0664 |
| *Musca vetustissima* | XP_061391442.1 | Non-AMP | 0.3917 |
| *Ochlerotatus camptorhynchus* | Ocam_Bit_LysC_352* | AMP | 0.9995 |
| *Ochlerotatus camptorhynchus* | Ocam_Bit_LysC_354 | AMP | 0.9785 |
| *Ochlerotatus camptorhynchus* | Ocam_Bit_LysC_355 | AMP | 0.9969 |
| *Ostrinia furnacalis* | Ofur_Bit_LysC_362 | AMP | 0.9999 |
| *Ostrinia furnacalis* | Ofur_Bit_LysC_365 | AMP | 1 |
| *Ostrinia nubilalis* | Onub_Bit_LysC_366 | AMP | 0.9999 |
| *Ostrinia nubilalis* | Onub_Bit_LysC_367 | AMP | 1 |
| *Phlebotomus argentipes* | Parg_Bit_LysC_380 | AMP | 0.9926 |
| *Phlebotomus argentipes* | Parg_Bit_LysC_381* | AMP | 0.9761 |
| *Phlebotomus papatasi* | Ppap_Bit_LysC_384* | AMP | 0.9739 |
| *Polypedilum vanderplanki* | Pvan_Bit_LysC_391 | AMP | 0.9968 |
| *Polypedilum vanderplanki* | Pvan_Bit_LysC_392 | Non-AMP | 0.4851 |
| *Polypedilum vanderplanki* | Pvan_Bit_LysC_393 | AMP | 0.8794 |
| *Rhynchophorus ferrugineus* | KAF7266660.1 | AMP | 1 |
| *Sabethes cyaneus* | Scya_Bit_LysC_397 | AMP | 0.9962 |
| *Sabethes cyaneus* | Scya_Bit_LysC_400* | AMP | 0.9887 |
| *Sabethes cyaneus* | Scya_Bit_LysC_401 | Non-AMP | 0.0722 |
| *Sabethes cyaneus* | Scya_Bit_LysC_403 | AMP | 0.9586 |
| *Sabethes cyaneus* | Scya_Bit_LysC_404 | AMP | 0.976 |
| *Sabethes cyaneus* | Scya_Bit_LysC_405 | AMP | 0.7952 |
| *Scaptodrosophila lebanonensis* | XP_030388068.1 | AMP | 0.9988 |
| *Scaptodrosophila lebanonensis* | Sleb_Bit_LysC_406 | AMP | 0.9492 |
| *Scaptodrosophila lebanonensis* | Sleb_Bit_LysC_407 | Non-AMP | 0.0136 |
| *Scaptodrosophila lebanonensis* | Sleb_Bit_LysC_408 | Non-AMP | 0.1587 |
| *Scylla paramamosain* | Spar_Bit_LysC_411* | AMP | 0.9999 |
| *Sitodiplosis mosellana* | Smos_Bit_LysC_412 | AMP | 0.7018 |
| *Stomoxys calcitrans* | XP_013099402.2 | AMP | 1 |
| *Stomoxys calcitrans* | XP_013117888.1 | AMP | 0.9142 |
| *Stomoxys calcitrans* | Scal_Bit_LysC_418 | Non-AMP | 0.3424 |
| *Stomoxys calcitrans* | Scal_Bit_LysC_421 | Non-AMP | 0.2218 |
| *Stomoxys calcitrans* | Scal_Bit_LysC_422 | Non-AMP | 0.3327 |
| *Topomyia yanbarensis* | Tyan_Bit_LysC_425 | AMP | 0.9954 |
| *Topomyia yanbarensis* | Tyan_Bit_LysC_426 | AMP | 0.9989 |
| *Topomyia yanbarensis* | Tyan_Bit_LysC_427 | AMP | 0.9997 |
| *Toxorhynchites rutilus septentrionalis* | Trut_Bit_LysC_430 | AMP | 0.8428 |
| *Trichoplusia ni* | Tni_Bit_LysC_437 | AMP | 0.9992 |
| *Uranotaenia lowii* | Ulow_Bit_LysC_438 | AMP | 0.918 |
| *Uranotaenia lowii* | Ulow_Bit_LysC_439 | AMP | 0.9843 |
| *Uranotaenia lowii* | Ulow_Bit_LysC_440 | Non-AMP | 0.051 |
| *Uranotaenia lowii* | Ulow_Bit_LysC_441 | AMP | 0.9999 |
| *Wyeomyia smithii* | Wsmi_Bit_LysC_446 | Non-AMP | 0.2053 |
| *Wyeomyia smithii* | Wsmi_Bit_LysC_447 | Non-AMP | 0.4849 |
| *Wyeomyia smithii* | Wsmi_Bit_LysC_448 | AMP | 0.582 |
| *Wyeomyia smithii* | Wsmi_Bit_LysC_449 | AMP | 0.7186 |
| *Wyeomyia smithii* | Wsmi_Bit_LysC_450 | AMP | 0.8679 |
| *Zeugodacus cucurbitae* | Zcuc_Bit_LysC_455 | Non-AMP | 0.0444 |
| *Zeugodacus cucurbitae* | Zcuc_Bit_LysC_456* | AMP | 0.7144 |
| *Zeugodacus cucurbitae* | Zcuc_Bit_LysC_457 | AMP | 0.9705 |

*Sequence names marked with an asterisk indicate low-confidence predictions due to reduced sequence length.

**Table S7** - Results predicted by AMP scanner for each sequence I-type lysozyme copies.

| **Species** | **SeqID** | **Prediction_Class** | **Prediction_Probability** |
| --- | --- | --- | --- |
| *Abscondita terminalis* | KAF5280354.1 | AMP | 0.9999 |
| *Abscondita terminalis* | KAF5291386.1 | AMP | 0.9998 |
| *Abscondita terminalis* | KAF5291491.1 | AMP | 0.9981 |
| *Achroia grisella* | XP_059057658.1 | AMP | 0.9983 |
| *Achroia grisella* | XP_059057679.1 | AMP | 0.9999 |
| *Achroia grisella* | XP_059057901.1 | AMP | 0.9998 |
| *Acromyrmex echinatior* | XP_011054762.1 | AMP | 0.975 |
| *Acromyrmex echinatior* | XP_011054763.1 | AMP | 0.995 |
| *Acromyrmex echinatior* | XP_011054766.1 | AMP | 0.9985 |
| *Acyrthosiphon pisum* | NP_001155465.1 | AMP | 0.9909 |
| *Acyrthosiphon pisum* | NP_001156290.1 | AMP | 0.9997 |
| *Acyrthosiphon pisum* | XP_001949318.2 | AMP | 0.9999 |
| *Adelges cooleyi* | XP_050430201.1 | AMP | 0.9998 |
| *Adelges cooleyi* | XP_050430202.1 | AMP | 0.9999 |
| *Aedes aegypti* | XP_001653399.1 | AMP | 0.9998 |
| *Aedes aegypti* | XP_021706771.1 | AMP | 0.9999 |
| *Aedes albopictus* | XP_019529410.2 | AMP | 0.9998 |
| *Aedes albopictus* | XP_029716839.1 | AMP | 0.9999 |
| *Aethina tumida* | XP_019866650.1 | AMP | 0.9999 |
| *Aethina tumida* | XP_019871899.1 | AMP | 0.9994 |
| *Aethina tumida* | XP_019872050.1 | AMP | 0.9998 |
| *Aethina tumida* | XP_019877370.1 | AMP | 1 |
| *Aethina tumida* | XP_019880378.1 | AMP | 0.9064 |
| *Aethina tumida* | XP_019880411.2 | AMP | 0.9931 |
| *Aethina tumida* | XP_049826320.1 | Non-AMP | 0.4409 |
| *Aethina tumida* | XP_049826532.1 | AMP | 0.9997 |
| *Agrilus planipennis* | XP_018319801.1 | AMP | 0.9998 |
| *Agrilus planipennis* | XP_018332491.1 | AMP | 0.9999 |
| *Ampulex compressa* | KAG7197751.1 | AMP | 0.9996 |
| *Amyelois transitella* | XP_013200866.1 | AMP | 0.9991 |
| *Amyelois transitella* | XP_060804852.1 | AMP | 0.9999 |
| *Amyelois transitella* | XP_060804862.1 | AMP | 0.9999 |
| *Anabrus simplex* | XP_067011434.2 | AMP | 0.9998 |
| *Anabrus simplex* | XP_067011443.1 | AMP | 0.9981 |
| *Anabrus simplex* | XP_067011504.2 | AMP | 0.9963 |
| *Anabrus simplex* | XP_067011516.2 | AMP | 0.9995 |
| *Anabrus simplex* | XP_068085865.1 | AMP | 0.9972 |
| *Anabrus simplex* | Asim_Bit_LysI_13 | AMP | 0.9999 |
| *Anastrepha ludens* | XP_053963913.1 | AMP | 0.9997 |
| *Anastrepha obliqua* | XP_054739455.1 | AMP | 0.9997 |
| *Anopheles albimanus* | XP_035787305.1 | AMP | 0.9989 |
| *Anopheles albimanus* | XP_035790116.1 | AMP | 1 |
| *Anopheles albimanus* | Aalb_Bit_LysI_18 | AMP | 0.9986 |
| *Anopheles aquasalis* | XP_050094991.1 | AMP | 0.9986 |
| *Anopheles arabiensis* | XP_040169303.1 | AMP | 0.9988 |
| *Anopheles arabiensis* | XP_040172453.1 | AMP | 0.9995 |
| *Anopheles bellator* | XP_058053247.1 | AMP | 0.9971 |
| *Anopheles coluzzii* | XP_040231303.1 | AMP | 0.9989 |
| *Anopheles coluzzii* | XP_040240181.2 | AMP | 0.9992 |
| *Anopheles coustani* | XP_058130205.1 | AMP | 0.9972 |
| *Anopheles coustani* | Acou_Bit_LysI_20 | AMP | 1 |
| *Anopheles cruzii* | XP_052869241.1 | AMP | 0.9985 |
| *Anopheles cruzii* | XP_052872413.1 | AMP | 0.9997 |
| *Anopheles darlingi* | XP_049544687.1 | AMP | 0.9989 |
| *Anopheles darlingi* | XP_049549285.1 | AMP | 1 |
| *Anopheles funestus* | XP_049279333.1 | AMP | 0.9999 |
| *Anopheles funestus* | XP_049288959.1 | AMP | 0.9985 |
| *Anopheles gambiae* | XP_309527.4 | AMP | 0.9988 |
| *Anopheles gambiae* | XP_310846.5 | AMP | 0.9992 |
| *Anopheles maculipalpis* | XP_050072165.1 | AMP | 1 |
| *Anopheles merus* | XP_041764501.1 | AMP | 0.9992 |
| *Anopheles merus* | XP_041784664.1 | AMP | 0.9988 |
| *Anopheles moucheti* | XP_052900856.1 | AMP | 0.9998 |
| *Anopheles moucheti* | XP_052903210.1 | AMP | 0.9995 |
| *Anopheles nili* | XP_053678939.1 | AMP | 1 |
| *Anopheles nili* | XP_053681166.1 | AMP | 0.9991 |
| *Anopheles nili* | Anil_Bit_LysI_23 | AMP | 1 |
| *Anopheles stephensi* | XP_035895099.1 | AMP | 0.9997 |
| *Anopheles stephensi* | XP_035903243.1 | AMP | 0.9984 |
| *Anopheles stephensi* | Aste_Bit_LysI_24 | AMP | 0.9998 |
| *Anopheles ziemanni* | XP_058171699.1 | AMP | 0.9972 |
| *Anopheles ziemanni* | XP_058175843.1 | AMP | 1 |
| *Anoplophora glabripennis* | XP_018567491.1 | AMP | 0.9999 |
| *Anoplophora glabripennis* | XP_018567494.1 | AMP | 0.985 |
| *Anoplophora glabripennis* | XP_018577274.1 | AMP | 0.9326 |
| *Anthonomus grandis grandis* | XP_050308861.1 | AMP | 0.9996 |
| *Aphidius gifuensis* | XP_044010230.1 | AMP | 0.9986 |
| *Aphidius gifuensis* | XP_044015570.1 | AMP | 0.9999 |
| *Aphis craccivora* | KAF0763625.1 | AMP | 0.999 |
| *Aphis gossypii* | XP_027836879.1 | AMP | 0.9999 |
| *Aphis gossypii* | XP_027836880.1 | AMP | 0.9997 |
| *Aphis gossypii* | XP_027836881.1 | AMP | 0.9997 |
| *Apis cerana* | XP_061929234.1 | AMP | 0.9982 |
| *Apis dorsata* | XP_006611951.1 | AMP | 0.9981 |
| *Apis florea* | XP_003693440.1 | AMP | 0.9979 |
| *Apis laboriosa* | XP_043791153.1 | AMP | 0.9981 |
| *Apis mellifera* | XP_393161.3 | AMP | 0.9981 |
| *Apolygus lucorum* | KAF6208716.1 | AMP | 0.9993 |
| *Arctia plantaginis* | CAB3230188.1 | AMP | 0.9996 |
| *Arctia plantaginis* | CAB3246916.1 | AMP | 0.9999 |
| *Arctia plantaginis* | Apla_Bit_LysI_30 | AMP | 0.9998 |
| *Aricia agestis* | XP_041981992.1 | AMP | 0.9961 |
| *Aricia agestis* | XP_041981997.1 | AMP | 0.9991 |
| *Aricia agestis* | XP_041982000.1 | AMP | 0.9995 |
| *Aricia agestis* | XP_041982001.1 | AMP | 0.999 |
| *Armigeres subalbatus* | XP_062551159.1 | AMP | 0.9992 |
| *Armigeres subalbatus* | XP_062557977.1 | AMP | 0.9993 |
| *Athalia rosae* | XP_012261020.2 | AMP | 0.9988 |
| *Atta cephalotes* | XP_012057581.1 | AMP | 0.9947 |
| *Atta cephalotes* | XP_012057582.1 | AMP | 0.9948 |
| *Atta cephalotes* | XP_012057632.1 | AMP | 0.9984 |
| *Atta colombica* | XP_018045716.1 | AMP | 0.995 |
| *Atta colombica* | XP_018045722.1 | AMP | 0.9947 |
| *Atta colombica* | XP_018045730.1 | AMP | 0.9985 |
| *Bacillus rossius redtenbacheri* | XP_063228967.1 | AMP | 0.9988 |
| *Bacillus rossius redtenbacheri* | XP_063243382.1 | AMP | 0.9998 |
| *Bacillus rossius redtenbacheri* | XP_063243383.1 | AMP | 0.9997 |
| *Bacillus rossius redtenbacheri* | XP_063243386.1 | AMP | 0.9991 |
| *Bacillus rossius redtenbacheri* | XP_063243618.1 | AMP | 0.841 |
| *Bactrocera dorsalis* | XP_011213791.1 | AMP | 0.9998 |
| *Bactrocera latifrons* | XP_018798928.1 | AMP | 0.9997 |
| *Bactrocera tryoni* | XP_039957323.1 | AMP | 0.9997 |
| *Belonocnema kinseyi* | XP_033228797.1 | AMP | 0.998 |
| *Bemisia tabaci* | XP_018912502.1 | AMP | 0.9996 |
| *Bicyclus anynana* | XP_023937544.2 | AMP | 0.999 |
| *Bicyclus anynana* | XP_052743212.1 | AMP | 0.9996 |
| *Blattella germanica* | PSN30087.1 | AMP | 0.9999 |
| *Blattella germanica* | PSN44787.1 | AMP | 0.9838 |
| *Bombus affinis* | XP_050578182.1 | AMP | 0.9984 |
| *Bombus terrestris* | XP_003394100.1 | AMP | 0.9984 |
| *Bombyx mandarina* | XP_028042436.1 | AMP | 0.9992 |
| *Bombyx mandarina* | XP_028042581.1 | AMP | 0.9997 |
| *Bombyx mori* | XP_004926301.1 | AMP | 1 |
| *Bombyx mori* | XP_004926302.1 | AMP | 0.9993 |
| *Bombyx mori* | XP_012543940.1 | AMP | 0.9997 |
| *Bradysia coprophila* | XP_037032108.1 | AMP | 0.9955 |
| *Bradysia coprophila* | XP_037050797.1 | AMP | 0.9984 |
| *Bradysia coprophila* | Bcop_Bit_LysI_57 | AMP | 0.9905 |
| *Brassicogethes aeneus* | CAH0547600.1 | AMP | 0.9999 |
| *Brassicogethes aeneus* | CAH0553837.1 | AMP | 0.999 |
| *Brassicogethes aeneus* | CAH0553864.1 | AMP | 1 |
| *Brassicogethes aeneus* | CAH0563606.1 | AMP | 0.9946 |
| *Brassicogethes aeneus* | CAH0563635.1 | AMP | 0.9997 |
| *Brassicogethes aeneus* | CAH0563638.1 | AMP | 0.9997 |
| *Brassicogethes aeneus* | CAH0563640.1 | AMP | 0.9989 |
| *Brenthis ino* | CAH0720439.1 | AMP | 0.9995 |
| *Brenthis ino* | CAH0720440.1 | AMP | 0.9974 |
| *Brenthis ino* | CAH0720503.1 | AMP | 0.9989 |
| *Calliphora vicina* | XP_065367465.1 | AMP | 0.9994 |
| *Calliphora vicina* | XP_065369652.1 | AMP | 0.9549 |
| *Camponotus floridanus* | XP_011251218.1 | AMP | 0.9992 |
| *Cataglyphis hispanica* | XP_050465440.1 | AMP | 0.9983 |
| *Cataglyphis hispanica* | XP_050466072.1 | AMP | 0.9987 |
| *Cephus cinctus* | XP_015600321.1 | AMP | 0.9991 |
| *Ceratina calcarata* | XP_017887289.1 | AMP | 0.9981 |
| *Ceratitis capitata* | XP_004520300.1 | AMP | 0.9998 |
| *Chelonus insularis* | XP_034936055.1 | AMP | 0.9998 |
| *Chilo suppressalis* | RVE45466.1 | AMP | 0.9995 |
| *Chilo suppressalis* | RVE45468.1 | AMP | 1 |
| *Chrysoperla carnea* | XP_044738420.1 | AMP | 0.9998 |
| *Cimex lectularius* | XP_014259832.1* | AMP | 1 |
| *Cimex lectularius* | XP_024086362.1 | AMP | 0.9996 |
| *Cimex lectularius* | Clec_Bit_LysI_71 | AMP | 1 |
| *Cloeon dipterum* | XP_065340083.1 | AMP | 0.9997 |
| *Cloeon dipterum* | XP_065341333.1 | AMP | 0.9991 |
| *Clunio marinus* | CRK95595.1 | AMP | 0.9282 |
| *Coccinella septempunctata* | XP_044758967.1 | AMP | 0.9999 |
| *Coccinella septempunctata* | XP_044758968.1 | AMP | 0.9948 |
| *Coccinella septempunctata* | XP_044759553.1 | AMP | 0.9998 |
| *Coccinella septempunctata* | XP_044759592.1 | AMP | 0.9952 |
| *Colias croceus* | XP_045500255.1 | AMP | 0.9995 |
| *Colias croceus* | XP_045500256.1 | AMP | 0.9999 |
| *Colias croceus* | XP_045500363.1 | AMP | 0.9996 |
| *Colletes gigas* | XP_043254615.1 | AMP | 0.9989 |
| *Condylostylus longicornis* | XP_055372737.1 | AMP | 0.9999 |
| *Condylostylus longicornis* | XP_055383329.1 | AMP | 0.9975 |
| *Condylostylus longicornis* | XP_055383412.1 | AMP | 0.9986 |
| *Contarinia nasturtii* | XP_031622100.1 | AMP | 1 |
| *Copidosoma floridanum* | XP_014219639.1 | AMP | 0.978 |
| *Copidosoma floridanum* | XP_014219782.1 | AMP | 0.9997 |
| *Coptotermes formosanus* | GFG32751.1 | AMP | 0.9979 |
| *Cotesia glomerata* | XP_044595690.1 | AMP | 0.9999 |
| *Cotesia glomerata* | XP_044595691.1 | AMP | 0.9999 |
| *Cryptotermes secundus* | XP_023710050.1 | AMP | 0.9978 |
| *Cryptotermes secundus* | XP_023720996.1 | AMP | 0.9991 |
| *Cryptotermes secundus* | XP_023724511.1 | AMP | 0.9995 |
| *Cryptotermes secundus* | XP_023724520.1 | AMP | 0.9902 |
| *Culex pipiens pallens* | XP_039432737.1 | AMP | 0.9995 |
| *Culex pipiens pallens* | XP_039442526.1 | AMP | 0.9997 |
| *Culex quinquefasciatus* | XP_001850854.2 | AMP | 0.9995 |
| *Culex quinquefasciatus* | XP_001869584.1 | AMP | 0.9995 |
| *Culicoides brevitarsis* | XP_063709554.1 | AMP | 0.9998 |
| *Culicoides brevitarsis* | XP_063710136.1 | AMP | 0.9992 |
| *Cydia pomonella* | XP_061718776.1 | AMP | 0.9996 |
| *Cydia pomonella* | XP_061718893.1 | AMP | 0.9993 |
| *Cydia pomonella* | XP_061718898.1 | AMP | 0.9999 |
| *Cydia splendana* | XP_063625533.1 | AMP | 0.9999 |
| *Cydia splendana* | XP_063625912.1 | AMP | 0.9993 |
| *Cydia splendana* | XP_063625913.1 | AMP | 0.9998 |
| *Cylas formicarius* | XP_060522272.1 | AMP | 0.9999 |
| *Cylas formicarius* | XP_060522299.1 | AMP | 0.9999 |
| *Cylas formicarius* | Cfor_Bit_LysI_93 | AMP | 0.9998 |
| *Daktulosphaira vitifoliae* | XP_050535820.1 | AMP | 0.9998 |
| *Daktulosphaira vitifoliae* | XP_050535823.1 | AMP | 0.9999 |
| *Dalotia coriaria* | XP_065157864.1 | AMP | 0.9999 |
| *Dalotia coriaria* | XP_065159526.1 | AMP | 0.9996 |
| *Danaus plexippus* | XP_032519101.2 | AMP | 0.9998 |
| *Danaus plexippus* | XP_032519102.2 | AMP | 0.9755 |
| *Danaus plexippus* | XP_032519113.1 | AMP | 0.9963 |
| *Danaus plexippus* | XP_032519140.2 | AMP | 0.9981 |
| *Danaus plexippus* | XP_061379210.1 | AMP | 0.9998 |
| *Danaus plexippus* | XP_061379293.1 | AMP | 0.9997 |
| *Dendroctonus ponderosae* | XP_019770495.1 | AMP | 0.9999 |
| *Diabrotica virgifera virgifera* | XP_028139698.2 | AMP | 0.9996 |
| *Diabrotica virgifera virgifera* | XP_028147223.2 | AMP | 0.9957 |
| *Diabrotica virgifera virgifera* | XP_050499110.1 | AMP | 0.9977 |
| *Diachasma alloeum* | XP_015113936.1 | AMP | 0.9999 |
| *Diachasmimorpha longicaudata* | XP_063973737.1 | AMP | 0.9999 |
| *Dinoponera quadriceps* | XP_014478038.1 | AMP | 0.9961 |
| *Diorhabda carinulata* | XP_057665005.1 | AMP | 0.9996 |
| *Diorhabda sublineata* | XP_056632869.1 | AMP | 0.9995 |
| *Diorhabda sublineata* | XP_056633228.1 | AMP | 0.9914 |
| *Diorhabda sublineata* | XP_056636029.1 | AMP | 0.9516 |
| *Diprion similis* | XP_046752470.1 | AMP | 0.9974 |
| *Drosophila albomicans* | XP_034101809.1 | AMP | 0.9958 |
| *Drosophila albomicans* | XP_034105438.1 | AMP | 0.9181 |
| *Drosophila albomicans* | XP_034108111.1 | AMP | 0.9959 |
| *Drosophila albomicans* | XP_034108112.1 | AMP | 0.9755 |
| *Drosophila albomicans* | XP_034108113.1 | AMP | 0.954 |
| *Drosophila ananassae* | XP_001960607.1 | AMP | 0.9795 |
| *Drosophila ananassae* | XP_001960608.1 | AMP | 0.9961 |
| *Drosophila ananassae* | XP_001960609.1 | AMP | 0.9112 |
| *Drosophila ananassae* | XP_001960610.1 | AMP | 0.9968 |
| *Drosophila biarmipes* | XP_016967990.1 | AMP | 0.9975 |
| *Drosophila biarmipes* | XP_016968180.1 | AMP | 0.9998 |
| *Drosophila biarmipes* | XP_016968181.1 | AMP | 0.9705 |
| *Drosophila bipectinata* | XP_017107974.1 | AMP | 0.9429 |
| *Drosophila bipectinata* | XP_017107983.1 | AMP | 0.9968 |
| *Drosophila bipectinata* | XP_017108514.2 | AMP | 0.9809 |
| *Drosophila bipectinata* | XP_017108515.2 | AMP | 0.9997 |
| *Drosophila bipectinata* | XP_017108565.1 | AMP | 0.997 |
| *Drosophila elegans* | XP_017124248.1 | AMP | 0.9995 |
| *Drosophila elegans* | XP_017124904.1 | AMP | 0.9975 |
| *Drosophila elegans* | XP_017125023.1 | AMP | 0.8959 |
| *Drosophila elegans* | XP_017125024.1 | AMP | 0.9997 |
| *Drosophila erecta* | XP_001975293.1 | AMP | 0.9618 |
| *Drosophila erecta* | XP_001975294.1 | AMP | 0.9985 |
| *Drosophila erecta* | XP_001975295.1 | AMP | 0.9826 |
| *Drosophila erecta* | XP_001975296.1 | AMP | 0.9909 |
| *Drosophila erecta* | Dere_Bit_LysI_115 | AMP | 0.9587 |
| *Drosophila eugracilis* | XP_017078142.1 | AMP | 0.9926 |
| *Drosophila eugracilis* | XP_017078834.1 | AMP | 0.9851 |
| *Drosophila eugracilis* | XP_017078835.1 | AMP | 0.9991 |
| *Drosophila eugracilis* | XP_017078836.1 | AMP | 0.9897 |
| *Drosophila ficusphila* | XP_017049516.1 | AMP | 0.9976 |
| *Drosophila ficusphila* | XP_017049517.1 | AMP | 0.9928 |
| *Drosophila ficusphila* | XP_017049518.2 | AMP | 0.9992 |
| *Drosophila ficusphila* | XP_017049519.1 | AMP | 0.9988 |
| *Drosophila grimshawi* | XP_001995413.2 | AMP | 0.9906 |
| *Drosophila grimshawi* | XP_001995414.1 | AMP | 0.9842 |
| *Drosophila grimshawi* | XP_001995415.1 | AMP | 0.9734 |
| *Drosophila grimshawi* | XP_032596805.1 | AMP | 0.9983 |
| *Drosophila guanche* | XP_034121841.1 | AMP | 0.9997 |
| *Drosophila guanche* | XP_034122092.1 | AMP | 0.9805 |
| *Drosophila guanche* | XP_034122218.1 | AMP | 0.9962 |
| *Drosophila guanche* | XP_034122349.1 | AMP | 0.9879 |
| *Drosophila guanche* | Dgua_Bit_LysI_120 | AMP | 0.9951 |
| *Drosophila hydei* | XP_023162250.1 | AMP | 0.9851 |
| *Drosophila hydei* | XP_023162833.2 | AMP | 0.9075 |
| *Drosophila hydei* | XP_023162865.2 | AMP | 0.9979 |
| *Drosophila hydei* | XP_023163021.2 | AMP | 0.9934 |
| *Drosophila kikkawai* | XP_017024388.1 | AMP | 0.9999 |
| *Drosophila kikkawai* | XP_017024390.1 | AMP | 0.9976 |
| *Drosophila kikkawai* | XP_017024453.1 | AMP | 0.703 |
| *Drosophila kikkawai* | XP_017024523.1 | AMP | 0.9985 |
| *Drosophila kikkawai* | Dkik_Bit_LysI_125 | AMP | 0.9978 |
| *Drosophila mauritiana* | XP_033153946.1 | AMP | 0.9804 |
| *Drosophila mauritiana* | XP_033153947.1 | AMP | 0.9514 |
| *Drosophila mauritiana* | XP_033153948.1 | AMP | 0.9977 |
| *Drosophila mauritiana* | XP_033155542.1 | AMP | 0.9909 |
| *Drosophila melanogaster* | NP_611163.2 | AMP | 0.9867 |
| *Drosophila melanogaster* | NP_611164.3 | AMP | 0.9991 |
| *Drosophila melanogaster* | NP_611165.2 | AMP | 0.9704 |
| *Drosophila melanogaster* | NP_652048.2 | AMP | 0.9485 |
| *Drosophila miranda* | XP_017145974.1 | AMP | 0.8991 |
| *Drosophila miranda* | XP_017150036.2 | AMP | 0.9971 |
| *Drosophila miranda* | XP_017150427.1 | AMP | 0.997 |
| *Drosophila miranda* | XP_017150428.1 | AMP | 0.9949 |
| *Drosophila miranda* | XP_033246840.1 | AMP | 0.9971 |
| *Drosophila miranda* | XP_033251848.1 | AMP | 0.9922 |
| *Drosophila mojavensis* | XP_002006620.1 | AMP | 0.9988 |
| *Drosophila mojavensis* | XP_032586218.2 | AMP | 0.9681 |
| *Drosophila mojavensis* | XP_043867237.1 | AMP | 0.9889 |
| *Drosophila montana* | XP_064535936.1 | AMP | 0.9956 |
| *Drosophila montana* | XP_064535937.1 | AMP | 0.9982 |
| *Drosophila montana* | XP_064535962.1 | AMP | 0.9906 |
| *Drosophila montana* | XP_064535967.1 | AMP | 0.9825 |
| *Drosophila montana* | XP_064535968.1 | AMP | 0.9899 |
| *Drosophila montana* | Dmon_Bit_LysI_136 | AMP | 0.9935 |
| *Drosophila nasuta* | XP_060654699.1 | AMP | 0.9959 |
| *Drosophila nasuta* | XP_060654701.1 | AMP | 0.9853 |
| *Drosophila nasuta* | XP_060654702.1 | AMP | 0.9999 |
| *Drosophila nasuta* | XP_060655724.1 | AMP | 0.8051 |
| *Drosophila nasuta* | XP_060660871.1 | AMP | 0.9958 |
| *Drosophila navojoa* | XP_017960257.1 | AMP | 0.8785 |
| *Drosophila navojoa* | XP_017960258.1 | AMP | 0.9988 |
| *Drosophila navojoa* | XP_017960259.1 | AMP | 0.9889 |
| *Drosophila navojoa* | XP_017960260.1 | AMP | 0.9976 |
| *Drosophila novamexicana* | XP_030563370.1 | AMP | 0.9964 |
| *Drosophila novamexicana* | XP_030563371.1 | AMP | 0.9976 |
| *Drosophila novamexicana* | XP_030563443.1 | AMP | 0.9588 |
| *Drosophila novamexicana* | XP_030563477.1 | AMP | 0.9825 |
| *Drosophila novamexicana* | XP_030563480.1 | AMP | 0.9981 |
| *Drosophila obscura* | XP_022230244.2 | AMP | 0.8623 |
| *Drosophila obscura* | XP_022230245.1 | AMP | 0.9899 |
| *Drosophila obscura* | XP_022230254.1 | AMP | 0.9975 |
| *Drosophila persimilis* | XP_002016414.1 | AMP | 0.9971 |
| *Drosophila persimilis* | XP_002016415.1 | AMP | 0.9273 |
| *Drosophila persimilis* | XP_002016416.1 | AMP | 0.9961 |
| *Drosophila persimilis* | XP_002016417.2 | AMP | 0.9973 |
| *Drosophila pseudoobscura* | XP_001360779.2 | AMP | 0.9971 |
| *Drosophila pseudoobscura* | XP_001360780.4 | AMP | 0.9987 |
| *Drosophila pseudoobscura* | XP_001360781.3 | AMP | 0.8991 |
| *Drosophila pseudoobscura* | XP_001360782.4 | AMP | 0.9974 |
| *Drosophila rhopaloa* | XP_016988659.2 | AMP | 0.998 |
| *Drosophila rhopaloa* | XP_016988814.1 | AMP | 0.991 |
| *Drosophila rhopaloa* | XP_016988815.1 | AMP | 0.9447 |
| *Drosophila rhopaloa* | XP_016988832.1 | AMP | 0.9995 |
| *Drosophila sechellia* | XP_002034193.1 | AMP | 0.9909 |
| *Drosophila sechellia* | XP_002034194.2 | AMP | 0.9598 |
| *Drosophila sechellia* | XP_002034196.1 | AMP | 0.9984 |
| *Drosophila sechellia* | XP_002034197.1 | AMP | 0.9598 |
| *Drosophila serrata* | XP_020799746.1 | AMP | 0.9986 |
| *Drosophila serrata* | XP_020818069.1 | AMP | 0.999 |
| *Drosophila serrata* | XP_020818080.1 | AMP | 0.9999 |
| *Drosophila serrata* | XP_020818092.1 | AMP | 0.8157 |
| *Drosophila simulans* | XP_002081833.1 | AMP | 0.9514 |
| *Drosophila simulans* | XP_002081834.1 | AMP | 0.9979 |
| *Drosophila simulans* | XP_002081835.1 | AMP | 0.9725 |
| *Drosophila simulans* | XP_016028063.1 | AMP | 0.9909 |
| *Drosophila subobscura* | XP_034650405.1 | AMP | 0.9994 |
| *Drosophila subobscura* | XP_034650407.1 | AMP | 0.9971 |
| *Drosophila subobscura* | XP_034650408.1 | AMP | 0.9135 |
| *Drosophila subobscura* | XP_034650409.1 | AMP | 0.9888 |
| *Drosophila subpulchrella* | XP_037717617.1 | AMP | 0.9831 |
| *Drosophila subpulchrella* | XP_037717618.1 | AMP | 0.993 |
| *Drosophila subpulchrella* | XP_037717619.1 | AMP | 0.9512 |
| *Drosophila subpulchrella* | XP_037717621.1 | AMP | 0.9976 |
| *Drosophila sulfurigaster albostrigata* | XP_062127526.1 | AMP | 0.8033 |
| *Drosophila sulfurigaster albostrigata* | XP_062127527.1 | AMP | 0.9958 |
| *Drosophila sulfurigaster albostrigata* | XP_062131871.1 | AMP | 0.9958 |
| *Drosophila sulfurigaster albostrigata* | XP_062131872.1 | AMP | 0.9876 |
| *Drosophila sulfurigaster albostrigata* | XP_062131874.1 | AMP | 0.9999 |
| *Drosophila suzukii* | XP_016927878.1 | AMP | 0.9967 |
| *Drosophila suzukii* | XP_016929517.1 | AMP | 0.9283 |
| *Drosophila suzukii* | XP_016929518.1 | AMP | 0.9358 |
| *Drosophila suzukii* | XP_016929519.1 | AMP | 0.999 |
| *Drosophila takahashii* | XP_017001973.2 | AMP | 0.985 |
| *Drosophila takahashii* | XP_017001974.1 | AMP | 0.9892 |
| *Drosophila takahashii* | XP_017001975.2 | AMP | 0.9999 |
| *Drosophila takahashii* | XP_017001981.1 | AMP | 0.985 |
| *Drosophila teissieri* | XP_043644072.1 | AMP | 0.9979 |
| *Drosophila teissieri* | XP_043644498.1 | AMP | 0.9395 |
| *Drosophila teissieri* | XP_043644973.1 | AMP | 0.9911 |
| *Drosophila teissieri* | XP_043645883.1 | AMP | 0.9838 |
| *Drosophila virilis* | XP_002048988.2 | AMP | 0.9955 |
| *Drosophila virilis* | XP_002048989.1 | AMP | 0.9983 |
| *Drosophila virilis* | XP_002048990.1 | AMP | 0.9625 |
| *Drosophila virilis* | XP_002048992.1 | AMP | 0.9889 |
| *Drosophila virilis* | XP_002048993.3 | AMP | 0.993 |
| *Drosophila yakuba* | XP_002092146.2 | AMP | 0.9888 |
| *Drosophila yakuba* | XP_002092147.1 | AMP | 0.9979 |
| *Drosophila yakuba* | XP_002092148.1 | AMP | 0.9427 |
| *Drosophila yakuba* | XP_002092149.1 | AMP | 0.9909 |
| *Dufourea novaeangliae* | XP_015429338.1 | AMP | 0.9994 |
| *Eciton burchellii* | KAH0955834.1 | AMP | 0.9987 |
| *Episyrphus balteatus* | XP_055855253.1 | AMP | 1 |
| *Episyrphus balteatus* | XP_055855624.1 | AMP | 0.9998 |
| *Episyrphus balteatus* | Ebal_Bit_LysI_182 | AMP | 0.9998 |
| *Eriocheir sinensis* | XP_050716977.1 | AMP | 0.9998 |
| *Eriocheir sinensis* | XP_050716984.1 | AMP | 0.9998 |
| *Eufriesea mexicana* | XP_017757357.1 | AMP | 0.9944 |
| *Eupeodes corollae* | XP_055905749.1 | AMP | 0.9996 |
| *Eupeodes corollae* | XP_055905910.1 | AMP | 0.9996 |
| *Eupeodes corollae* | XP_055905911.1 | AMP | 0.9999 |
| *Folsomia candida* | XP_021948031.1 | AMP | 0.9993 |
| *Folsomia candida* | XP_021948033.1 | AMP | 0.9981 |
| *Folsomia candida* | Fcan_Bit_LysI_187 | AMP | 0.9975 |
| *Folsomia candida* | Fcan_Bit_LysI_188 | AMP | 0.9952 |
| *Fopius arisanus* | XP_011313368.1 | AMP | 0.9999 |
| *Formica exsecta* | XP_029663395.1 | AMP | 0.9981 |
| *Formica exsecta* | XP_029663397.1 | AMP | 0.9867 |
| *Frankliniella occidentalis* | Focc_Bit_LysI_191 | AMP | 1 |
| *Frankliniella occidentalis* | Focc_Bit_LysI_193 | AMP | 0.987 |
| *Frieseomelitta varia* | XP_043522477.1 | AMP | 0.9977 |
| *Galleria mellonella* | XP_031764870.1 | AMP | 0.9996 |
| *Galleria mellonella* | XP_052751132.1 | AMP | 0.9983 |
| *Galleria mellonella* | XP_052751158.1 | AMP | 0.9999 |
| *Glossina fuscipes* | XP_037897161.1 | AMP | 0.9706 |
| *Glossina fuscipes* | XP_037898951.1 | AMP | 0.9995 |
| *Gonioctena quinquepunctata* | KAG5865195.1 | AMP | 0.6676 |
| *Gonioctena quinquepunctata* | KAG5887020.1 | AMP | 1 |
| *Gryllus longicercus* | KAK7868956.1 | AMP | 0.9963 |
| *Gryllus longicercus* | KAK7869611.1 | AMP | 0.9998 |
| *Gryllus longicercus* | KAK7869612.1 | AMP | 0.9997 |
| *Gryllus longicercus* | KAK7869613.1 | AMP | 0.9989 |
| *Halyomorpha halys* | XP_014272957.1 | AMP | 0.9978 |
| *Harmonia axyridis* | XP_045475786.1 | AMP | 0.9998 |
| *Harmonia axyridis* | XP_045475794.1 | AMP | 0.7801 |
| *Harmonia axyridis* | XP_045475795.1 | AMP | 0.9999 |
| *Harmonia axyridis* | XP_045477746.1 | AMP | 0.9972 |
| *Harmonia axyridis* | XP_045477750.1 | AMP | 0.9996 |
| *Harmonia axyridis* | XP_045477751.1 | AMP | 0.9704 |
| *Harpegnathos saltator* | XP_011154006.1 | AMP | 0.9991 |
| *Helicoverpa armigera* | XP_021196195.3 | AMP | 0.9989 |
| *Helicoverpa armigera* | XP_049706905.2 | AMP | 0.9998 |
| *Helicoverpa armigera* | XP_063895237.1 | AMP | 0.9999 |
| *Helicoverpa zea* | XP_047032192.1 | AMP | 0.9993 |
| *Helicoverpa zea* | XP_047032257.1 | AMP | 0.9998 |
| *Helicoverpa zea* | XP_047032394.1 | AMP | 0.9999 |
| *Hermetia illucens* | XP_037917352.1 | AMP | 0.9999 |
| *Hetaerina americana* | XP_071439142.1 | AMP | 0.9992 |
| *Hetaerina americana* | XP_071455191.1 | AMP | 0.9976 |
| *Homalodisca vitripennis* | XP_046673719.1 | AMP | 0.9997 |
| *Homalodisca vitripennis* | XP_046675156.1 | AMP | 0.9997 |
| *Homalodisca vitripennis* | Hvit_Bit_LysI_208 | AMP | 0.9982 |
| *Hylaeus anthracinus* | XP_054007809.1 | AMP | 0.9993 |
| *Hylaeus volcanicus* | XP_053987056.1 | AMP | 0.9986 |
| *Hyposmocoma kahamanoa* | XP_026321249.1 | AMP | 0.9995 |
| *Hyposmocoma kahamanoa* | XP_026321478.1 | AMP | 0.9999 |
| *Ignelater luminosus* | KAF2880577.1 | AMP | 0.997 |
| *Ignelater luminosus* | KAF2880578.1 | AMP | 0.9453 |
| *Ignelater luminosus* | KAF2881207.1 | AMP | 0.9996 |
| *Ignelater luminosus* | KAF2881208.1 | AMP | 0.9998 |
| *Ignelater luminosus* | KAF2900787.1 | AMP | 1 |
| *Ignelater luminosus* | Ilum_Bit_LysI_214 | AMP | 0.9998 |
| *Ischnura elegans* | XP_046406548.1 | AMP | 0.9993 |
| *Ischnura elegans* | XP_046406711.1 | AMP | 0.9981 |
| *Ischnura elegans* | XP_046407288.1 | AMP | 0.9987 |
| *Lamprigera yunnana* | KAF5270630.1 | AMP | 0.9999 |
| *Lamprigera yunnana* | KAF5282601.1 | AMP | 1 |
| *Lamprigera yunnana* | KAF5288757.1 | AMP | 0.9916 |
| *Laodelphax striatellus* | RZF31955.1 | AMP | 0.9999 |
| *Laodelphax striatellus* | RZF31956.1 | AMP | 0.9999 |
| *Laodelphax striatellus* | RZF31957.1 | AMP | 1 |
| *Laodelphax striatellus* | RZF38433.1 | AMP | 0.9986 |
| *Laodelphax striatellus* | RZF43287.1 | AMP | 0.9016 |
| *Laodelphax striatellus* | RZF47177.1 | AMP | 0.996 |
| *Laodelphax striatellus* | RZF47247.1 | AMP | 0.9999 |
| *Leguminivora glycinivorella* | XP_047992342.1 | AMP | 0.9996 |
| *Leguminivora glycinivorella* | XP_047992344.1 | AMP | 0.9994 |
| *Leguminivora glycinivorella* | XP_047992557.1 | AMP | 0.9999 |
| *Leptidea sinapis* | XP_050665855.1 | AMP | 0.9996 |
| *Leptidea sinapis* | XP_050665869.1 | AMP | 1 |
| *Leptinotarsa decemlineata* | XP_023027517.1 | AMP | 0.9996 |
| *Leptopilina boulardi* | XP_051153286.1 | AMP | 0.9996 |
| *Leptopilina heterotoma* | XP_043482938.1 | AMP | 0.9996 |
| *Linepithema humile* | XP_012235794.1 | AMP | 0.9994 |
| *Lucilia cuprina* | XP_046810602.1 | AMP | 0.9995 |
| *Lucilia sericata* | XP_037814307.1 | AMP | 0.9995 |
| *Lucilia sericata* | Lser_Bit_LysI_228 | AMP | 0.9996 |
| *Lutzomyia longipalpis* | XP_055694017.1 | AMP | 0.9999 |
| *Malaya genurostris* | XP_058461445.1 | AMP | 0.9999 |
| *Malaya genurostris* | Mgen_Bit_LysI_230 | AMP | 0.9976 |
| *Manduca sexta* | XP_030022728.2 | AMP | 0.9999 |
| *Manduca sexta* | XP_030032079.1 | AMP | 0.9988 |
| *Manduca sexta* | XP_030037656.1 | AMP | 0.9986 |
| *Manduca sexta* | XP_037293183.1 | AMP | 0.9984 |
| *Maniola hyperantus* | XP_034834743.1 | AMP | 0.9998 |
| *Maniola hyperantus* | XP_034834939.1 | AMP | 0.9994 |
| *Maniola jurtina* | XP_045774324.1 | AMP | 0.9998 |
| *Maniola jurtina* | XP_045774325.1 | AMP | 0.9988 |
| *Maniola jurtina* | XP_045775262.1 | AMP | 0.9998 |
| *Megachile rotundata* | XP_003708146.1 | AMP | 0.994 |
| *Megalopta genalis* | XP_033331203.1 | AMP | 0.9983 |
| *Melanaphis sacchari* | XP_025204785.1 | AMP | 0.9999 |
| *Melanaphis sacchari* | XP_025204808.1 | AMP | 0.9998 |
| *Melanaphis sacchari* | XP_025204821.1 | AMP | 0.9998 |
| *Melitaea cinxia* | XP_045451169.1 | AMP | 1 |
| *Melitaea cinxia* | XP_045451177.1 | AMP | 0.998 |
| *Melitaea cinxia* | XP_045451358.1 | AMP | 0.9996 |
| *Monomorium pharaonis* | XP_012523214.2 | AMP | 0.9993 |
| *Monomorium pharaonis* | XP_012523223.2 | AMP | 0.9367 |
| *Monomorium pharaonis* | XP_012534115.1 | AMP | 0.9991 |
| *Musca domestica* | XP_019892932.2 | AMP | 0.9998 |
| *Musca vetustissima* | XP_061398689.1 | AMP | 0.9998 |
| *Myzus persicae* | XP_022168115.1 | AMP | 0.9999 |
| *Myzus persicae* | XP_022168131.1 | AMP | 0.9997 |
| *Myzus persicae* | XP_022168136.1 | AMP | 0.9997 |
| *Nasonia vitripennis* | XP_031786281.1 | AMP | 0.9998 |
| *Neocloeon triangulifer* | XP_059475803.1 | AMP | 0.9999 |
| *Neocloeon triangulifer* | XP_059475804.1 | AMP | 0.9994 |
| *Neocloeon triangulifer* | XP_059482021.1 | AMP | 0.9932 |
| *Neodiprion fabricii* | XP_046417441.1 | AMP | 0.9988 |
| *Neodiprion lecontei* | XP_015512018.2 | AMP | 0.9991 |
| *Neodiprion pinetum* | XP_046474960.1 | AMP | 0.9991 |
| *Neodiprion virginianus* | XP_046604985.1 | AMP | 0.999 |
| *Nicrophorus vespilloides* | XP_017782892.1 | AMP | 0.9997 |
| *Nicrophorus vespilloides* | XP_017786634.1 | AMP | 0.8649 |
| *Nicrophorus vespilloides* | XP_017786636.1 | AMP | 0.9965 |
| *Nilaparvata lugens* | XP_039275327.1 | AMP | 0.9856 |
| *Nilaparvata lugens* | XP_039275329.1 | AMP | 0.9144 |
| *Nilaparvata lugens* | XP_039275335.1 | AMP | 0.9208 |
| *Nilaparvata lugens* | XP_039275632.1 | AMP | 0.9999 |
| *Nilaparvata lugens* | XP_039280087.1 | AMP | 0.9989 |
| *Nilaparvata lugens* | XP_039280368.1 | AMP | 0.9284 |
| *Nilaparvata lugens* | XP_039280379.1 | AMP | 0.9943 |
| *Nilaparvata lugens* | XP_039284892.1 | AMP | 0.9999 |
| *Nilaparvata lugens* | XP_039284906.1 | AMP | 1 |
| *Nilaparvata lugens* | XP_039284912.1 | AMP | 1 |
| *Nilaparvata lugens* | XP_039301533.1 | AMP | 0.8837 |
| *Nilaparvata lugens* | Nlug_Bit_LysI_253 | AMP | 0.9869 |
| *Nilaparvata lugens* | Nlug_Bit_LysI_254 | AMP | 0.9899 |
| *Nomia melanderi* | XP_031844191.1 | AMP | 0.9996 |
| *Nylanderia fulva* | XP_029178206.1 | AMP | 0.9975 |
| *Nymphalis io* | XP_050347671.1 | AMP | 0.9995 |
| *Nymphalis io* | XP_050347672.1 | AMP | 0.9994 |
| *Nymphalis io* | XP_050347673.1 | AMP | 0.9993 |
| *Ochlerotatus camptorhynchus* | XP_065091966.1 | AMP | 0.9998 |
| *Odontomachus brunneus* | XP_032676328.1 | AMP | 0.9958 |
| *Onthophagus taurus* | XP_022905649.1 | AMP | 0.9931 |
| *Onthophagus taurus* | XP_022909582.1 | AMP | 0.7527 |
| *Onthophagus taurus* | XP_022916282.1 | AMP | 0.9999 |
| *Onthophagus taurus* | XP_022917006.1 | AMP | 0.9997 |
| *Onthophagus taurus* | XP_022920100.1 | AMP | 0.9997 |
| *Ooceraea biroi* | XP_011347650.1 | AMP | 0.9979 |
| *Orussus abietinus* | XP_012270123.1 | AMP | 0.9985 |
| *Osmia bicornis bicornis* | XP_029049920.1 | AMP | 0.9977 |
| *Osmia lignaria* | XP_034182671.1 | AMP | 0.9977 |
| *Ostrinia furnacalis* | XP_028161325.1 | AMP | 0.9998 |
| *Ostrinia furnacalis* | XP_028161455.1 | AMP | 0.9994 |
| *Ostrinia furnacalis* | XP_028161463.1 | AMP | 1 |
| *Ostrinia furnacalis* | XP_028172991.1 | AMP | 0.9991 |
| *Ostrinia nubilalis* | XP_063824435.1 | AMP | 0.9991 |
| *Ostrinia nubilalis* | XP_063829054.1 | AMP | 0.9998 |
| *Ostrinia nubilalis* | XP_063829396.1 | AMP | 1 |
| *Ostrinia nubilalis* | XP_063829397.1 | AMP | 0.9994 |
| *Papilio machaon* | XP_014357737.2 | AMP | 0.9991 |
| *Papilio machaon* | XP_014361407.2 | AMP | 0.9985 |
| *Papilio machaon* | XP_045537211.1 | AMP | 0.9999 |
| *Papilio polytes* | XP_013140804.1 | AMP | 0.9999 |
| *Papilio polytes* | XP_013140984.1 | AMP | 0.9998 |
| *Papilio polytes* | XP_013140998.1 | AMP | 0.9999 |
| *Papilio xuthus* | XP_013166586.1 | AMP | 0.9974 |
| *Papilio xuthus* | XP_013179764.1 | AMP | 0.9997 |
| *Papilio xuthus* | XP_013180160.1 | AMP | 0.9999 |
| *Pararge aegeria* | XP_039756259.1 | AMP | 0.9932 |
| *Pararge aegeria* | XP_039756415.1 | AMP | 0.9996 |
| *Pararge aegeria* | XP_039756541.1 | AMP | 0.9995 |
| *Parnassius apollo* | CAG5033417.1 | AMP | 0.9998 |
| *Parnassius apollo* | Papo_Bit_LysI_269 | AMP | 0.9998 |
| *Parnassius apollo* | Papo_Bit_LysI_270 | AMP | 0.9999 |
| *Pectinophora gossypiella* | XP_049876206.1 | AMP | 0.9994 |
| *Pectinophora gossypiella* | XP_049876207.1 | AMP | 0.9998 |
| *Pectinophora gossypiella* | XP_049876210.1 | AMP | 0.9986 |
| *Pediculus humanus corporis* | XP_002432291.1 | AMP | 0.9973 |
| *Periplaneta americana* | XP_069675917.1 | AMP | 0.9994 |
| *Periplaneta americana* | XP_069684582.1 | AMP | 0.9991 |
| *Periplaneta americana* | XP_069684586.1 | Non-AMP | 0.472 |
| *Periplaneta americana* | XP_069684589.1 | AMP | 0.9985 |
| *Periplaneta americana* | XP_069684590.1 | AMP | 0.9999 |
| *Phlebotomus argentipes* | XP_059622353.1 | AMP | 0.9997 |
| *Photinus pyralis* | XP_031343566.1 | AMP | 0.9854 |
| *Photinus pyralis* | XP_031343568.1 | AMP | 0.9281 |
| *Photinus pyralis* | XP_031352464.1 | AMP | 0.9932 |
| *Photinus pyralis* | XP_031352536.1 | AMP | 0.9968 |
| *Photinus pyralis* | XP_031354613.1 | AMP | 0.9997 |
| *Photinus pyralis* | XP_031355015.1 | AMP | 0.9999 |
| *Phymastichus coffea* | XP_058797514.1 | AMP | 0.9998 |
| *Pieris brassicae* | XP_045522728.1 | AMP | 1 |
| *Pieris brassicae* | XP_045523216.1 | AMP | 0.9999 |
| *Pieris brassicae* | XP_045523460.1 | AMP | 0.9997 |
| *Pieris napi* | XP_047521846.1 | AMP | 0.9997 |
| *Pieris napi* | XP_047521933.1 | AMP | 0.9998 |
| *Pieris napi* | XP_047521941.1 | AMP | 1 |
| *Pieris rapae* | XP_022118333.2 | AMP | 1 |
| *Pieris rapae* | XP_022118347.2 | AMP | 0.9999 |
| *Pieris rapae* | XP_022119358.2 | AMP | 0.9997 |
| *Planococcus citri* | XP_065219084.1 | AMP | 0.9995 |
| *Planococcus citri* | XP_065219901.1 | AMP | 0.9608 |
| *Planococcus citri* | XP_065220772.1 | AMP | 0.9982 |
| *Planococcus citri* | XP_065220773.1 | AMP | 0.9993 |
| *Plodia interpunctella* | XP_053610465.1 | AMP | 0.9985 |
| *Plodia interpunctella* | XP_053610466.1 | AMP | 0.9997 |
| *Plodia interpunctella* | XP_053611021.1 | AMP | 0.9999 |
| *Plutella xylostella* | XP_037973282.2 | AMP | 0.9995 |
| *Pogonomyrmex barbatus* | XP_011630017.1 | AMP | 0.9997 |
| *Polistes canadensis* | XP_014615199.1 | AMP | 0.9988 |
| *Polistes dominula* | XP_015186257.1 | AMP | 0.9975 |
| *Polistes fuscatus* | XP_043491128.1 | AMP | 0.999 |
| *Polypedilum vanderplanki* | KAG5681060.1 | AMP | 0.9993 |
| *Procambarus clarkii* | XP_045609367.1 | AMP | 0.9992 |
| *Procambarus clarkii* | XP_045609368.1 | AMP | 1 |
| *Procambarus clarkii* | XP_045609373.1 | AMP | 1 |
| *Procambarus clarkii* | XP_045609376.1 | AMP | 0.9991 |
| *Pseudomyrmex gracilis* | XP_020277533.1 | AMP | 0.9997 |
| *Rhopalosiphum maidis* | XP_026808594.1 | AMP | 0.9999 |
| *Rhopalosiphum maidis* | XP_026809847.1 | AMP | 0.9998 |
| *Rhopalosiphum maidis* | XP_026813536.1 | AMP | 0.9998 |
| *Rhopalosiphum padi* | XP_060840585.1 | AMP | 0.9999 |
| *Rhopalosiphum padi* | XP_060841310.1 | AMP | 0.9998 |
| *Rhopalosiphum padi* | XP_060842535.1 | AMP | 0.9998 |
| *Rhynchophorus ferrugineus* | KAF7267050.1 | AMP | 1 |
| *Rhynchophorus ferrugineus* | KAF7267053.1 | AMP | 0.9998 |
| *Sabethes cyaneus* | XP_053684879.1 | AMP | 0.9999 |
| *Sabethes cyaneus* | XP_053693932.1 | AMP | 0.9996 |
| *Scaptodrosophila lebanonensis* | XP_030382593.1 | AMP | 0.9891 |
| *Scaptodrosophila lebanonensis* | XP_030382648.1 | AMP | 0.8355 |
| *Scaptodrosophila lebanonensis* | XP_030382652.1 | AMP | 0.9968 |
| *Scaptodrosophila lebanonensis* | XP_030387482.1 | AMP | 0.9998 |
| *Scylla paramamosain* | XP_063858873.1 | AMP | 0.9994 |
| *Sipha flava* | XP_025407187.1 | AMP | 0.9999 |
| *Sitodiplosis mosellana* | XP_055319149.1 | AMP | 0.9999 |
| *Sitophilus oryzae* | XP_030767998.1 | AMP | 0.9999 |
| *Sitophilus oryzae* | XP_030768031.1 | AMP | 0.9999 |
| *Solenopsis invicta* | XP_025988686.1 | AMP | 0.9824 |
| *Solenopsis invicta* | Sinv_Bit_LysI_304 | AMP | 0.999 |
| *Spodoptera frugiperda* | XP_035444103.1 | AMP | 0.9993 |
| *Spodoptera frugiperda* | XP_050549629.1 | AMP | 0.9999 |
| *Spodoptera frugiperda* | XP_050549807.1 | AMP | 0.9992 |
| *Spodoptera litura* | XP_022822579.1 | AMP | 0.9995 |
| *Spodoptera litura* | XP_022822980.1 | AMP | 1 |
| *Spodoptera litura* | XP_022823164.1 | AMP | 0.9998 |
| *Stomoxys calcitrans* | XP_013100126.1 | AMP | 0.9992 |
| *Temnothorax curvispinosus* | XP_024883366.1 | AMP | 0.9941 |
| *Temnothorax curvispinosus* | XP_024883393.1 | AMP | 0.9961 |
| *Temnothorax curvispinosus* | XP_024883394.1 | AMP | 0.9961 |
| *Temnothorax longispinosus* | Tlon_Bit_LysI_316 | AMP | 0.9891 |
| *Tenebrio molitor* | CAH1375994.1 | AMP | 1 |
| *Tenebrio molitor* | CAH1375995.1 | AMP | 0.9992 |
| *Tenebrio molitor* | CAH1375996.1 | AMP | 0.9816 |
| *Tenebrio molitor* | CAH1377254.1 | AMP | 0.9986 |
| *Tenebrio molitor* | CAH1377255.1 | AMP | 0.9995 |
| *Tenebrio molitor* | CAH1377256.1 | AMP | 0.9942 |
| *Tenebrio molitor* | CAH1377258.1 | AMP | 0.9999 |
| *Tenebrio molitor* | Tmol_Bit_LysI_319 | AMP | 1 |
| *Thrips palmi* | XP_034244875.1 | AMP | 0.9997 |
| *Thrips palmi* | XP_034244880.1 | AMP | 0.9972 |
| *Thrips palmi* | Tpal_Bit_LysI_321 | AMP | 0.9998 |
| *Topomyia yanbarensis* | XP_058819470.1 | AMP | 0.9998 |
| *Topomyia yanbarensis* | XP_058833866.1 | AMP | 0.9997 |
| *Toxorhynchites rutilus septentrionalis* | XP_055642625.1 | AMP | 0.9991 |
| *Trachymyrmex septentrionalis* | XP_018351309.1 | AMP | 0.9986 |
| *Trachymyrmex septentrionalis* | XP_018351318.1 | AMP | 0.9987 |
| *Trachymyrmex septentrionalis* | XP_018351321.1 | AMP | 0.9984 |
| *Tribolium castaneum* | XP_008196186.2 | AMP | 0.9703 |
| *Tribolium castaneum* | XP_015837940.1 | AMP | 0.9904 |
| *Tribolium castaneum* | XP_015837941.1 | AMP | 1 |
| *Tribolium castaneum* | Tcas_Bit_LysI_325 | AMP | 0.9761 |
| *Tribolium madens* | XP_044252909.1 | AMP | 0.9823 |
| *Tribolium madens* | XP_044252932.1 | AMP | 0.9865 |
| *Tribolium madens* | XP_044252938.1 | AMP | 1 |
| *Tribolium madens* | XP_044272652.1 | AMP | 0.9959 |
| *Trichogramma pretiosum* | XP_014221985.1 | AMP | 0.9999 |
| *Trichogramma pretiosum* | XP_014222012.1 | AMP | 0.9996 |
| *Trichoplusia ni* | XP_026734095.1 | AMP | 0.9999 |
| *Trichoplusia ni* | XP_026735897.1 | AMP | 1 |
| *Trichoplusia ni* | XP_026735904.1 | AMP | 0.9999 |
| *Uranotaenia lowii* | XP_055599146.1 | AMP | 0.9996 |
| *Vanessa atalanta* | XP_047535403.1 | AMP | 0.9996 |
| *Vanessa atalanta* | XP_047535463.1 | AMP | 0.9936 |
| *Vanessa atalanta* | XP_047535557.1 | AMP | 0.9999 |
| *Vanessa cardui* | XP_046969447.1 | AMP | 0.9908 |
| *Vanessa cardui* | XP_046969639.1 | AMP | 0.9995 |
| *Vanessa cardui* | XP_046969725.1 | AMP | 0.9997 |
| *Vanessa tameamea* | XP_026495706.2 | AMP | 0.9998 |
| *Vanessa tameamea* | XP_026495858.1 | AMP | 0.9994 |
| *Vanessa tameamea* | XP_064073527.1 | AMP | 0.9933 |
| *Venturia canescens* | XP_043276197.1 | AMP | 0.9996 |
| *Vespa velutina* | XP_047348953.1 | AMP | 0.9996 |
| *Vespula pensylvanica* | XP_043667361.1 | AMP | 0.9993 |
| *Vespula vulgaris* | XP_050848304.1 | AMP | 0.9993 |
| *Vollenhovia emeryi* | XP_011865731.1 | AMP | 0.9981 |
| *Vollenhovia emeryi* | XP_011865733.1 | AMP | 0.9935 |
| *Vollenhovia emeryi* | XP_011868866.1 | AMP | 0.9999 |
| *Wasmannia auropunctata* | XP_011696003.1 | AMP | 0.9998 |
| *Wasmannia auropunctata* | XP_011696004.1 | AMP | 0.9987 |
| *Wasmannia auropunctata* | XP_011696023.1 | AMP | 0.9899 |
| *Wasmannia auropunctata* | Waur_Bit_LysI_334 | AMP | 0.9926 |
| *Wyeomyia smithii* | XP_055535877.1 | AMP | 0.9998 |
| *Wyeomyia smithii* | XP_055546992.1 | AMP | 0.9983 |
| *Zerene cesonia* | XP_038222416.1 | AMP | 1 |
| *Zerene cesonia* | XP_038222477.1 | AMP | 0.9995 |
| *Zerene cesonia* | XP_038222597.1 | AMP | 0.9994 |
| *Zeugodacus cucurbitae* | XP_011192348.1 | AMP | 0.9998 |
| *Zootermopsis nevadensis* | XP_021918550.1 | AMP | 0.9996 |
| *Zootermopsis nevadensis* | XP_021918552.1 | AMP | 0.9991 |
| *Zootermopsis nevadensis* | XP_021918555.1 | AMP | 0.9992 |
| *Zootermopsis nevadensis* | XP_021918570.1 | AMP | 0.9989 |
| *Zootermopsis nevadensis* | XP_021926418.1 | AMP | 0.9996 |
| *Zophobas morio* | XP_063903686.1 | AMP | 0.9998 |
| *Zophobas morio* | XP_063903687.1 | AMP | 0.9991 |
| *Zophobas morio* | XP_063904458.1 | AMP | 0.9998 |
| *Zophobas morio* | XP_063904459.1 | AMP | 1 |
| *Zophobas morio* | XP_063904539.1 | AMP | 0.993 |
| *Zophobas morio* | XP_063904776.1 | AMP | 0.9999 |
| *Zophobas morio* | XP_063916582.1 | AMP | 0.9936 |
| *Zophobas morio* | XP_063916830.1 | AMP | 0.9101 |
| *Zophobas morio* | XP_063916949.1 | AMP | 0.9675 |
| *Zophobas morio* | Zmor_Bit_LysI_339 | AMP | 0.9998 |
| *Zophobas morio* | Zmor_Bit_LysI_341 | AMP | 0.9999 |
| *Abscondita terminalis* | KAF5269310.1 | AMP | 0.9291 |
| *Abscondita terminalis* | Ater_Bit_LysI_2 | AMP | 0.9998 |
| *Achroia grisella* | Agri_Bit_LysI_4 | AMP | 0.9996 |
| *Adelges cooleyi* | Acoo_Bit_LysI_7 | AMP | 0.9992 |
| *Adelges cooleyi* | Acoo_Bit_LysI_8 | AMP | 0.9982 |
| *Anabrus simplex* | XP_068085863.1 | AMP | 0.9986 |
| *Anabrus simplex* | Asim_Bit_LysI_14 | AMP | 0.9999 |
| *Anastrepha ludens* | Alud_Bit_LysI_16 | AMP | 0.9977 |
| *Anastrepha obliqua* | Aobl_Bit_LysI_17 | AMP | 0.9982 |
| *Anopheles aquasalis* | XP_050084419.1 | AMP | 1 |
| *Anopheles maculipalpis* | XP_050079812.1 | AMP | 0.9981 |
| *Anopheles maculipalpis* | Amac_Bit_LysI_22 | AMP | 0.9981 |
| *Aphis craccivora* | Acra_Bit_LysI_26 | AMP | 0.9904 |
| *Aphis craccivora* | Acra_Bit_LysI_27 | AMP | 0.9997 |
| *Aphis gossypii* | Agos_Bit_LysI_28 | AMP | 0.9874 |
| *Arctia plantaginis* | Apla_Bit_LysI_29 | AMP | 0.9985 |
| *Aricia agestis* | Aage_Bit_LysI_31 | AMP | 0.9963 |
| *Bacillus rossius redtenbacheri* | Bros_Bit_LysI_35 | AMP | 0.9985 |
| *Bactrocera dorsalis* | Bdor_Bit_LysI_41 | AMP | 0.9204 |
| *Bactrocera latifrons* | Blat_Bit_LysI_43 | AMP | 0.9995 |
| *Bactrocera tryoni* | Btry_Bit_LysI_44 | AMP | 0.8939 |
| *Belonocnema kinseyi* | XP_033229112.1 | AMP | 0.998 |
| *Bemisia tabaci* | Btab_Bit_LysI_46 | AMP | 0.9954 |
| *Bemisia tabaci* | Btab_Bit_LysI_48 | AMP | 0.9927 |
| *Blattella germanica* | Bger_Bit_LysI_49 | AMP | 0.9999 |
| *Blattella germanica* | Bger_Bit_LysI_50 | AMP | 0.9999 |
| *Blattella germanica* | Bger_Bit_LysI_51 | AMP | 0.999 |
| *Bombyx mandarina* | XP_028041526.1 | AMP | 1 |
| *Calliphora vicina* | Cvic_Bit_LysI_62 | AMP | 0.8863 |
| *Ceratitis capitata* | Ccap_Bit_LysI_65 | AMP | 0.9999 |
| *Ceratosolen solmsi marchali* | XP_011505724.1 | AMP | 0.9864 |
| *Chilo suppressalis* | RVE42167.1 | AMP | 0.9999 |
| *Chilo suppressalis* | RVE49873.1 | AMP | 0.9999 |
| *Chilo suppressalis* | Csup_Bit_LysI_68 | AMP | 0.9994 |
| *Chilo suppressalis* | Csup_Bit_LysI_69 | AMP | 0.9996 |
| *Cimex lectularius* | Clec_Bit_LysI_70 | AMP | 0.9996 |
| *Coccinella septempunctata* | Csep_Bit_LysI_76 | AMP | 0.9998 |
| *Condylostylus longicornis* | Clon_Bit_LysI_80 | AMP | 0.996 |
| *Condylostylus longicornis* | Clon_Bit_LysI_81 | AMP | 0.9987 |
| *Coptotermes formosanus* | GFG31204.1 | AMP | 0.9998 |
| *Coptotermes formosanus* | GFG39893.1 | AMP | 0.9993 |
| *Cotesia glomerata* | Cglo_Bit_LysI_83 | AMP | 0.9993 |
| *Cotesia glomerata* | Cglo_Bit_LysI_84 | AMP | 0.9999 |
| *Cryptotermes secundus* | XP_023724516.1 | AMP | 0.9978 |
| *Cryptotermes secundus* | Csec_Bit_LysI_86 | AMP | 0.9967 |
| *Culex quinquefasciatus* | Cqui_Bit_LysI_88 | AMP | 0.9993 |
| *Cyphomyrmex costatus* | Ccos_Bit_LysI_94 | AMP | 0.9948 |
| *Cyphomyrmex costatus* | Ccos_Bit_LysI_95 | AMP | 0.9585 |
| *Daktulosphaira vitifoliae* | Dvit_Bit_LysI_96 | AMP | 0.9999 |
| *Daktulosphaira vitifoliae* | Dvit_Bit_LysI_97 | AMP | 0.9972 |
| *Danaus plexippus* | Dple_Bit_LysI_98 | AMP | 0.9994 |
| *Danaus plexippus* | Dple_Bit_LysI_99 | AMP | 0.9836 |
| *Diachasma alloeum* | Dall_Bit_LysI_100 | AMP | 0.9998 |
| *Diachasmimorpha longicaudata* | Dlon_Bit_LysI_101 | AMP | 0.9999 |
| *Diorhabda carinulata* | XP_057652365.1 | AMP | 0.981 |
| *Diorhabda sublineata* | Dsub_Bit_LysI_102 | AMP | 0.9899 |
| *Drosophila albomicans* | Dalb_Bit_LysI_103* | AMP | 0.8386 |
| *Drosophila ananassae* | Dana_Bit_LysI_104* | AMP | 0.9993 |
| *Drosophila arizonae* | Dari_Bit_LysI_105 | AMP | 0.9128 |
| *Drosophila biarmipes* | XP_016967679.1 | AMP | 0.9871 |
| *Drosophila biarmipes* | Dbia_Bit_LysI_107* | AMP | 0.9805 |
| *Drosophila bipectinata* | Dbip_Bit_LysI_110* | AMP | 0.9988 |
| *Drosophila erecta* | Dere_Bit_LysI_114* | AMP | 0.9999 |
| *Drosophila eugracilis* | Deug_Bit_LysI_117* | AMP | 0.9948 |
| *Drosophila grimshawi* | Dgri_Bit_LysI_119* | AMP | 0.9951 |
| *Drosophila guanche* | Dgua_Bit_LysI_121 | AMP | 0.9999 |
| *Drosophila mauritiana* | Dmau_Bit_LysI_126* | AMP | 0.9995 |
| *Drosophila melanogaster* | Dmel_Bit_LysI_129* | AMP | 0.9996 |
| *Drosophila miranda* | Dmir_Bit_LysI_132 | AMP | 0.7031 |
| *Drosophila miranda* | Dmir_Bit_LysI_133* | AMP | 0.9989 |
| *Drosophila miranda* | Dmir_Bit_LysI_134 | AMP | 0.9957 |
| *Drosophila mojavensis* | XP_002006621.2 | AMP | 0.9994 |
| *Drosophila mojavensis* | Dmoj_Bit_LysI_135 | AMP | 0.9252 |
| *Drosophila montana* | XP_064536162.1* | AMP | 0.9647 |
| *Drosophila nasuta* | Dnas_Bit_LysI_137* | AMP | 0.6418 |
| *Drosophila novamexicana* | XP_030572543.1* | AMP | 0.9656 |
| *Drosophila obscura* | XP_022230242.2* | AMP | 0.9925 |
| *Drosophila obscura* | Dobs_Bit_LysI_142 | AMP | 0.9999 |
| *Drosophila persimilis* | Dper_Bit_LysI_147 | AMP | 0.9984 |
| *Drosophila pseudoobscura* | Dpse_Bit_LysI_150 | AMP | 0.9995 |
| *Drosophila rhopaloa* | Drho_Bit_LysI_152* | AMP | 0.9035 |
| *Drosophila sechellia* | Dsec_Bit_LysI_155* | AMP | 0.9975 |
| *Drosophila serrata* | Dser_Bit_LysI_157 | AMP | 1 |
| *Drosophila simulans* | Dsim_Bit_LysI_161* | AMP | 0.9991 |
| *Drosophila subobscura* | Dsub_Bit_LysI_162 | AMP | 0.9995 |
| *Drosophila sulfurigaster albostrigata* | Dsul_Bit_LysI_167* | AMP | 0.8963 |
| *Drosophila takahashii* | Dtak_Bit_LysI_169 | AMP | 0.9963 |
| *Drosophila teissieri* | Dtei_Bit_LysI_171* | AMP | 0.9991 |
| *Drosophila virilis* | XP_002046889.1* | AMP | 0.8561 |
| *Drosophila yakuba* | Dyak_Bit_LysI_174* | AMP | 0.9999 |
| *Episyrphus balteatus* | Ebal_Bit_LysI_176 | AMP | 0.9999 |
| *Episyrphus balteatus* | Ebal_Bit_LysI_177 | AMP | 0.9996 |
| *Episyrphus balteatus* | Ebal_Bit_LysI_178 | AMP | 0.9997 |
| *Episyrphus balteatus* | Ebal_Bit_LysI_180 | AMP | 0.9999 |
| *Episyrphus balteatus* | Ebal_Bit_LysI_181 | AMP | 0.9999 |
| *Eriocheir sinensis* | XP_050716991.1 | AMP | 0.9999 |
| *Eriocheir sinensis* | Esin_Bit_LysI_183 | AMP | 0.9998 |
| *Eupeodes corollae* | Ecor_Bit_LysI_185 | AMP | 0.9997 |
| *Formica exsecta* | XP_029670970.1 | AMP | 0.9949 |
| *Frankliniella occidentalis* | Focc_Bit_LysI_190 | AMP | 0.9925 |
| *Frankliniella occidentalis* | Focc_Bit_LysI_192 | AMP | 0.9984 |
| *Frankliniella occidentalis* | Focc_Bit_LysI_194 | AMP | 0.975 |
| *Frankliniella occidentalis* | Focc_Bit_LysI_195 | AMP | 0.9996 |
| *Frieseomelitta varia* | XP_043512327.1 | AMP | 1 |
| *Frieseomelitta varia* | Fvar_Bit_LysI_196 | AMP | 1 |
| *Galleria mellonella* | Gmel_Bit_LysI_198 | AMP | 0.9999 |
| *Galleria mellonella* | Gmel_Bit_LysI_199 | AMP | 0.9996 |
| *Galleria mellonella* | Gmel_Bit_LysI_200 | AMP | 0.9908 |
| *Glossina fuscipes* | XP_037901473.1* | AMP | 0.9942 |
| *Halyomorpha halys* | XP_024215773.1 | AMP | 0.9995 |
| *Homalodisca vitripennis* | XP_046674380.1 | AMP | 0.9998 |
| *Homalodisca vitripennis* | XP_046674492.1 | AMP | 0.9982 |
| *Homalodisca vitripennis* | XP_046688119.1 | AMP | 0.9997 |
| *Homalodisca vitripennis* | Hvit_Bit_LysI_205 | AMP | 0.9981 |
| *Homalodisca vitripennis* | Hvit_Bit_LysI_207 | AMP | 0.9993 |
| *Hyposmocoma kahamanoa* | Hkah_Bit_LysI_210 | AMP | 0.9991 |
| *Hyposmocoma kahamanoa* | Hkah_Bit_LysI_211 | AMP | 0.9998 |
| *Hyposmocoma kahamanoa* | Hkah_Bit_LysI_213 | AMP | 0.9998 |
| *Lamprigera yunnana* | Lyun_Bit_LysI_215 | AMP | 0.9854 |
| *Lamprigera yunnana* | Lyun_Bit_LysI_216 | AMP | 0.9962 |
| *Lamprigera yunnana* | Lyun_Bit_LysI_217 | AMP | 0.9996 |
| *Lamprigera yunnana* | Lyun_Bit_LysI_219 | AMP | 0.9991 |
| *Laodelphax striatellus* | Lstr_Bit_LysI_220 | AMP | 0.9996 |
| *Laodelphax striatellus* | Lstr_Bit_LysI_223 | AMP | 0.9944 |
| *Leguminivora glycinivorella* | Lgly_Bit_LysI_224 | AMP | 0.9999 |
| *Leptinotarsa decemlineata* | XP_023029680.1 | Non-AMP | 0.2444 |
| *Lucilia cuprina* | Lcup_Bit_LysI_227 | AMP | 0.9723 |
| *Lucilia sericata* | Lser_Bit_LysI_229 | AMP | 0.9813 |
| *Manduca sexta* | Msex_Bit_LysI_231 | AMP | 0.9999 |
| *Maniola hyperantus* | XP_034830033.1 | AMP | 0.9734 |
| *Maniola jurtina* | XP_045765838.1 | AMP | 0.9989 |
| *Melipona quadrifasciata* | KOX67703.1 | AMP | 0.999 |
| *Microplitis demolitor* | Mdem_Bit_LysI_235 | AMP | 0.9998 |
| *Microplitis mediator* | XP_057326862.1 | AMP | 0.9999 |
| *Musca domestica* | Mdom_Bit_LysI_237 | AMP | 0.6685 |
| *Musca domestica* | Mdom_Bit_LysI_238 | AMP | 0.9999 |
| *Musca domestica* | Mdom_Bit_LysI_239 | AMP | 0.9999 |
| *Musca vetustissima* | XP_061396450.1 | AMP | 0.9995 |
| *Musca vetustissima* | Mvet_Bit_LysI_240 | AMP | 0.8443 |
| *Neocloeon triangulifer* | Ntri_Bit_LysI_244 | AMP | 0.9991 |
| *Nicrophorus vespilloides* | XP_017775925.1 | AMP | 0.9999 |
| *Nilaparvata lugens* | XP_039275197.1 | AMP | 0.7983 |
| *Nilaparvata lugens* | Nlug_Bit_LysI_251 | AMP | 1 |
| *Osmia lignaria* | Olig_Bit_LysI_259 | AMP | 0.9972 |
| *Papilio polytes* | Ppol_Bit_LysI_266 | AMP | 0.9997 |
| *Papilio polytes* | Ppol_Bit_LysI_267 | AMP | 0.9998 |
| *Parnassius apollo* | CAG4944323.1 | AMP | 0.9995 |
| *Parnassius apollo* | CAG4944327.1 | AMP | 0.9995 |
| *Parnassius apollo* | Papo_Bit_LysI_272 | AMP | 0.9994 |
| *Pediculus humanus corporis* | XP_002424398.1 | AMP | 1 |
| *Pediculus humanus corporis* | XP_002432292.1 | AMP | 0.9999 |
| *Periplaneta americana* | Pame_Bit_LysI_276 | AMP | 0.9988 |
| *Periplaneta americana* | Pame_Bit_LysI_277 | AMP | 1 |
| *Photinus pyralis* | Ppyr_Bit_LysI_279 | AMP | 0.9585 |
| *Pieris brassicae* | Pbra_Bit_LysI_283 | AMP | 0.9998 |
| *Pieris brassicae* | Pbra_Bit_LysI_284 | AMP | 0.9998 |
| *Pieris napi* | Pnap_Bit_LysI_285 | AMP | 0.9998 |
| *Pieris rapae* | Prap_Bit_LysI_286 | AMP | 0.9998 |
| *Polypedilum vanderplanki* | Pvan_Bit_LysI_290 | AMP | 0.9992 |
| *Rhodnius prolixus* | RPRC011594 | AMP | 0.9997 |
| *Rhodnius prolixus* | Rpro_Bit_LysI_292 | AMP | 0.9997 |
| *Scaptodrosophila lebanonensis* | Sleb_Bit_LysI_296 | AMP | 0.9427 |
| *Scylla paramamosain* | Spar_Bit_LysI_298 | AMP | 0.9999 |
| *Sipha flava* | Sfla_Bit_LysI_300 | AMP | 0.9984 |
| *Sipha flava* | Sfla_Bit_LysI_301 | AMP | 0.9981 |
| *Sitophilus oryzae* | XP_030758604.1* | AMP | 1 |
| *Solenopsis invicta* | Sinv_Bit_LysI_303 | AMP | 0.9664 |
| *Stomoxys calcitrans* | Scal_Bit_LysI_309 | AMP | 0.9911 |
| *Stomoxys calcitrans* | Scal_Bit_LysI_310 | Non-AMP | 0.1318 |
| *Temnothorax longispinosus* | TGZ53120.1 | AMP | 0.5838 |
| *Temnothorax longispinosus* | Tlon_Bit_LysI_315 | AMP | 0.9975 |
| *Tenebrio molitor* | Tmol_Bit_LysI_317 | Non-AMP | 0.2276 |
| *Tenebrio molitor* | Tmol_Bit_LysI_318 | AMP | 0.9917 |
| *Thrips palmi* | Tpal_Bit_LysI_320 | AMP | 0.9987 |
| *Toxorhynchites rutilus septentrionalis* | XP_055619715.1* | AMP | 0.9998 |
| *Uranotaenia lowii* | XP_055589868.1* | AMP | 0.9998 |
| *Vanessa tameamea* | Vtam_Bit_LysI_331 | AMP | 0.9996 |
| *Vespa crabro* | XP_046821287.1 | AMP | 0.9997 |
| *Vespa mandarinia* | XP_035725596.1 | AMP | 0.9997 |
| *Zerene cesonia* | Zces_Bit_LysI_335 | AMP | 0.9986 |
| *Zeugodacus cucurbitae* | Zcuc_Bit_LysI_336 | AMP | 0.9989 |
| *Zootermopsis nevadensis* | XP_021941419.1 | AMP | 0.9999 |
| *Zophobas morio* | XP_063904777.1 | AMP | 0.9993 |
| *Zophobas morio* | Zmor_Bit_LysI_340 | AMP | 0.9953 |
| *Zophobas morio* | Zmor_Bit_LysI_345 | AMP | 1 |

**Table S8** - Comparison of evolutionary model fit and phylogenetic signal for C-type and I-type lysozymes. Model fit was evaluated using the corrected Akaike Information Criterion (AICc), where lower values indicate better support. Phylogenetic signal was assessed using Blomberg's K, Moran's I, and Pagel's λ.

| **Evolutionary model fit** | | | **Phylogenetic signal** | | |
| --- | --- | --- | --- | --- | --- |
| **Lysozyme type** | **Best model** | **AICc** | **Blomberg's K (p-value)** | **Lambda (p-value)** | **Moran's I (p-value)** |
| C-type | BMS | 1292.095 | 0.309 (0.001) | 0.984573 (7.67863E-135) | 0.1927614 (< 0.001) |
| I-type | OU1 | 1158.568 | 0.064 (0.003) | 0.964854 (8.37E-66) | 0.06887771 (< 0.001) |

**Table S9 -** PGLS results performed using both C-type and I-type lysozyme copies.

| C-type | | | | | | | | | | |
| --- | --- | --- | --- | --- | --- | --- | --- | --- | --- | --- |
| Adult | | | | | **Larvae** | | | | | |
| Category | **Value** | **Std.Error** | **t-value** | **p-value** | **Category** | **Value** | | **Std.Error** | **t-value** | **p-value** |
| (Intercept) | 1.569976 | 0.387144 | 4.055277 | 0.0001 | (Intercept) | 1.4142748 | | 0.4004214 | 3.531967 | 0.0005 |
| Blood | 0.1755791 | 0.2496738 | 0.703234 | 0.4825 | Blood | 0.1884594 | | 0.2832518 | 0.665342 | 0.5064 |
| Detritivore | 0.1423074 | 0.0811587 | 1.753447 | 0.0806 | Detritivore | 0.6730612 | | 0.3367738 | 1.998556 | 0.0466 |
| Invertebrates | -0.0811087 | 0.0966553 | -0.839154 | 0.4021 | Invertebrates | -0.0694374 | | 0.0968713 | -0.7168 | 0.4741 |
| Omnivore | -0.0788898 | 0.0835068 | -0.94471 | 0.3456 | Omnivore | -0.0703466 | | 0.0836101 | -0.841364 | 0.4009 |
| Parasitoid | -0.1540036 | 0.0915063 | -1.682984 | 0.0935 | Parasitoid | -0.1523356 | | 0.0913919 | -1.66684 | 0.0967 |
| AIC = 205.5069; BIC = 230.7732; logLik=-95.75347 | | |  |  | AIC=201.6935; BIC=226.9598; logLik=-93.84674 | | | |  |  |
|  |  |  |  |  |  | |  |  |  |  |
| I-type | | | | | | | | | | |
| Adult | | | | | **Larvae** | | | | | |
| Category | **Value** | **Std.Error** | **t-value** | **p-value** | **Category** | | **Value** | **Std.Error** | **t-value** | **p-value** |
| (Intercept) | 1.3385489 | 0.5000458 | 2.6768524 | 0.0079 | (Intercept) | | 1.260997 | 0.5175852 | 2.4363082 | 0.0155 |
| Blood | 0.0274474 | 0.3224856 | 0.0851119 | 0.9322 | Blood | | 0.0863661 | 0.3661317 | 0.2358882 | 0.8137 |
| Detritivore | 0.0160752 | 0.1048268 | 0.1533502 | 0.8782 | Detritivore | | 0.2500637 | 0.4353143 | 0.574444 | 0.5661 |
| Invertebrates | 0.1245722 | 0.1248427 | 0.9978332 | 0.3192 | Invertebrates | | 0.1307035 | 0.125216 | 1.0438243 | 0.2975 |
| Omnivore | 0.2729907 | 0.1078598 | 2.5309783 | 0.0119 | Omnivore | | 0.2774848 | 0.1080746 | 2.5675307 | 0.0108 |
| Parasitoid | 0.0482306 | 0.118192 | 0.40807 | 0.6835 | Parasitoid | | 0.0491425 | 0.1181333 | 0.415992 | 0.6777 |
| AIC=345.2301; BIC=370.4964; logLik=-165.615 | | |  |  | AIC=341.8281; BIC=367.0944; logLik=-163.914 | | | |  |  |

**Table S10 -** PGLS results performed for the number of cleavage sites.

| **Peptidase** | **C-type** | | | | | **I-type** | | | | |
| --- | --- | --- | --- | --- | --- | --- | --- | --- | --- | --- |
|  | **Category** | **Value** | **Std.Error** | **t-value** | **p-value** | **Category** | **Value** | **Std.Error** | **t-value** | **p-value** |
| Chymo-trypsin | (Intercept) | 12.517222 | 3.534367 | 3.541574 | 0.0005 | (Intercept) | 14.435381 | 3.149318 | 4.583653 | 0 |
|  | Blood | 1.952888 | 1.607028 | 1.215217 | 0.2253 | Blattodea | 0.943641 | 3.743491 | 0.252075 | 0.8012 |
|  | Detritivore | 1.03804 | 1.562667 | 0.664275 | 0.5071 | Blood | 0.040388 | 1.404289 | 0.02876 | 0.9771 |
|  | Invertebrates | 0.526627 | 1.586454 | 0.331952 | 0.7402 | Invertebrates | 0.843743 | 1.352849 | 0.623678 | 0.5334 |
|  | Omnivore | 0.341289 | 1.386315 | 0.246184 | 0.8057 | Omnivore | -0.625931 | 1.405109 | -0.445468 | 0.6563 |
|  | Parasitoid | -1.875361 | 1.944304 | -0.964541 | 0.3356 | Parasitoid | 0.76989 | 2.04569 | 0.376348 | 0.707 |
| Papain | (Intercept) | 4.674343 | 1.2748412 | 3.666608 | 0.0003 | (Intercept) | 3.812242 | 1.7332426 | 2.1994858 | 0.0287 |
|  | Blood | -0.147893 | 0.7047312 | -0.209858 | 0.8339 | Blattodea | 0.127086 | 2.0602487 | 0.0616846 | 0.9509 |
|  | Detritivore | -0.133282 | 0.7406886 | -0.179944 | 0.8573 | Blood | -0.146619 | 0.7728574 | -0.1897098 | 0.8497 |
|  | Invertebrates | 0.00812 | 0.6962521 | 0.011663 | 0.9907 | Invertebrates | -0.227771 | 0.7445469 | -0.3059192 | 0.7599 |
|  | Omnivore | -0.041901 | 0.5933126 | -0.070622 | 0.9438 | Omnivore | -0.70852 | 0.7733088 | -0.9162192 | 0.3604 |
|  | Parasitoid | -0.578676 | 0.9037272 | -0.640322 | 0.5225 | Parasitoid | -0.783787 | 1.1258553 | -0.6961707 | 0.4869 |
| Pepsin | (Intercept) | 25.890565 | 7.707953 | 3.358942 | 0.0009 | (Intercept) | 31.167239 | 6.219733 | 5.011025 | 0 |
|  | Blood | 4.985178 | 3.739241 | 1.333206 | 0.1836 | Blattodea | 2.572144 | 7.393193 | 0.347907 | 0.7282 |
|  | Detritivore | 2.701124 | 3.79029 | 0.712643 | 0.4767 | Blood | -0.072024 | 2.773395 | -0.02597 | 0.9793 |
|  | Invertebrates | 1.435519 | 3.816049 | 0.376179 | 0.7071 | Invertebrates | 2.117441 | 2.671803 | 0.792514 | 0.4288 |
|  | Omnivore | 1.513969 | 3.27912 | 0.4617 | 0.6447 | Omnivore | -1.067636 | 2.775015 | -0.384732 | 0.7007 |
|  | Parasitoid | -4.373028 | 5.011407 | -0.872615 | 0.3836 | Parasitoid | -2.802668 | 4.040127 | -0.693708 | 0.4885 |
| Trypsin | (Intercept) | 14.190585 | 3.565637 | 3.979818 | 0.0001 | (Intercept) | 11.312393 | 3.797036 | 2.9792698 | 0.0032 |
|  | Blood | -0.36075 | 1.207146 | -0.298845 | 0.7653 | Blattodea | 1.420869 | 4.513412 | 0.3148104 | 0.7531 |
|  | Detritivore | -1.275596 | 1.23017 | -1.036926 | 0.3007 | Blood | -0.486043 | 1.693108 | -0.2870715 | 0.7743 |
|  | Invertebrates | 0.519124 | 1.436343 | 0.361421 | 0.7181 | Invertebrates | 0.984003 | 1.631088 | 0.6032805 | 0.5468 |
|  | Omnivore | 0.222377 | 1.144362 | 0.194324 | 0.8461 | Omnivore | -0.889551 | 1.694097 | -0.5250887 | 0.5999 |
|  | Parasitoid | -2.067365 | 1.94924 | -1.0606 | 0.2898 | Parasitoid | -0.890935 | 2.466425 | -0.3612254 | 0.7182 |

**Table S11 -** Expression profile of C-type and I-type lysozyme genes in *Musca domestica* larvae

| **Lysozyme type** | **Transcript ID** | | **Protein ID** | | | **Expression (TPM)*** | | | | | | | | | | | | | | |
| --- | --- | --- | --- | --- | --- | --- | --- | --- | --- | --- | --- | --- | --- | --- | --- | --- | --- | --- | --- | --- |
|  |  |  |  |  |  | **AM1** | | **AM2** | | **MM1** | | **MM2** | | **PM1** | | **PM2** | | **PM3** | | **Car** |
| C-type | XM_020035063.2 | | XP_019890622.1 | | | 0.44 | | 61.60 | | 1896.14 | | 1182.31 | | 5.89 | | 0.17 | | 0.56 | | 0.13 |
|  | XM_020035061.2 | | XP_019890620.1 | | | 0.13 | | 22.81 | | 1187.45 | | 410.05 | | 2.07 | | 0.12 | | 0.53 | | 0.26 |
|  | XM_005178411.4 | | XP_005178468.1 | | | 0.00 | | 28.62 | | 1269.74 | | 216.63 | | 0.79 | | 0.00 | | 0.81 | | 0.14 |
|  | XM_059126266.1 | | XP_058982249.1 | | | 0.28 | | 410.46 | | 5116.05 | | 455.95 | | 3.66 | | 0.61 | | 1.75 | | 0.46 |
|  | XM_011292180.2 | | XP_011290482.1 | | | 0.29 | | 48.38 | | 2207.71 | | 452.55 | | 2.48 | | 0.12 | | 0.88 | | 0.34 |
|  | XM_005185318.2 | | XP_005185375.2 | | | 0.00 | | 6.68 | | 104.10 | | 34.53 | | 0.00 | | 0.00 | | 0.00 | | 0.00 |
|  | XM_020035060.2 | | XP_019890619.1 | | | 0.48 | | 74.75 | | 3450.14 | | 1551.96 | | 8.61 | | 0.43 | | 1.01 | | 0.50 |
|  | XM_005178415.4 | | XP_005178472.1 | | | 0.08 | | 20.41 | | 986.80 | | 526.78 | | 3.81 | | 1.21 | | 0.88 | | 0.00 |
|  | XM_005178432.1 | | XP_005178489.1 | | | 0.00 | | 0.00 | | 0.00 | | 0.00 | | 0.00 | | 0.00 | | 0.00 | | 0.00 |
|  | XM_020035064.2 | | XP_019890623.1 | | | 0.20 | | 59.02 | | 748.94 | | 90.17 | | 0.76 | | 0.24 | | 0.25 | | 0.00 |
|  | XM_005185770.4 | | XP_005185827.1 | | | 17300.46 | | 33194.41 | | 24809.29 | | 354.81 | | 116.16 | | 137.64 | | 195.15 | | 195.99 |
|  | XM_059128789.1 | | XP_058984772.1 | | | 0.00 | | 0.00 | | 0.00 | | 0.00 | | 0.00 | | 0.00 | | 0.00 | | 0.00 |
|  | XM_020037581.2 | | XP_019893140.1 | | | 13854.19 | | 22623.17 | | 15024.85 | | 198.50 | | 89.35 | | 113.10 | | 111.01 | | 171.25 |
|  | XM_005184849.3 | | XP_005184906.2 | | | 0.19 | | 0.00 | | 0.00 | | 0.00 | | 0.00 | | 0.00 | | 0.00 | | 0.10 |
|  | XM_020037580.2 | | XP_019893139.2 | | | 2891.31 | | 5274.13 | | 2583.03 | | 28.79 | | 16.22 | | 20.29 | | 27.81 | | 28.34 |
|  | XM_005185778.4 | | XP_005185835.2 | | | 19224.36 | | 32392.30 | | 15627.49 | | 163.72 | | 102.87 | | 133.28 | | 168.86 | | 229.46 |
|  | XM_020037574.2 | | XP_019893133.2 | | | 7784.66 | | 13497.18 | | 6869.24 | | 67.25 | | 43.58 | | 57.64 | | 59.25 | | 149.03 |
|  | XM_011293180.3 | | XP_011291482.2 | | | 68.09 | | 0.64 | | 1.30 | | 20.90 | | 0.65 | | 0.17 | | 0.36 | | 1919.98 |
|  | NM_001308972.2 | | NP_001295901 | | | 14821.51 | | 27585.19 | | 15456.02 | | 188.40 | | 98.01 | | 121.18 | | 98.85 | | 43.24 |
|  | XM_005185309.3 | | XP_005185366.1 | | | 0.30 | | 177.27 | | 2307.22 | | 554.98 | | 4.52 | | 0.41 | | 1.99 | | 0.30 |
|  | XM_011292181.3 | | XP_011290483.1 | | | 0.00 | | 13.71 | | 550.55 | | 180.41 | | 0.99 | | 0.00 | | 0.46 | | 0.14 |
|  | XM_020035062.2 | | XP_019890621.1 | | | 0.13 | | 22.25 | | 1113.51 | | 784.95 | | 4.39 | | 0.12 | | 0.72 | | 0.00 |
|  | XM_011292185.3 | | XP_011290487.2 | | | 1.05 | | 118.26 | | 4117.95 | | 2598.87 | | 12.39 | | 0.39 | | 2.57 | | 0.25 |
|  | XM_005185779.4 | | XP_005185836.1 | | | 9060.38 | | 9960.93 | | 8642.23 | | 170.52 | | 30.61 | | 42.21 | | 50.85 | | 68.60 |
|  | XM_059126267.1 | | XP_058982250.1 | | | 0.00 | | 61.27 | | 1109.81 | | 151.68 | | 1.02 | | 0.55 | | 0.98 | | 0.13 |
|  | XM_059127590.1 | | XP_058983573.1 | | | 13697.18 | | 32719.02 | | 18849.98 | | 220.75 | | 117.01 | | 116.96 | | 166.06 | | 52.09 |
|  | XM_059127591.1 | | XP_058983574.1 | | | 48196.64 | | 67300.61 | | 70171.41 | | 1144.78 | | 260.14 | | 320.67 | | 344.36 | | 947.16 |
|  | XM_059127592.1 | | XP_058983575.1 | | | 48196.64 | | 67300.61 | | 70171.41 | | 1144.78 | | 260.14 | | 320.67 | | 344.36 | | 947.16 |
|  | XM_059127594.1 | | XP_058983577.1 | | | 25221.58 | | 28510.31 | | 29050.87 | | 480.99 | | 94.16 | | 120.65 | | 151.86 | | 768.61 |
|  | XM_059127595.1 | | XP_058983578.1 | | | 15632.21 | | 24744.13 | | 13437.51 | | 145.61 | | 81.99 | | 110.40 | | 113.98 | | 182.68 |
|  | XM_059127596.1 | | XP_058983579.1 | | | 2970.94 | | 5393.30 | | 2783.69 | | 34.96 | | 15.49 | | 21.39 | | 31.29 | | 38.20 |
|  | XM_059127597.1 | | XP_058983580.1 | | | 7094.19 | | 10644.10 | | 7760.35 | | 106.88 | | 38.39 | | 46.69 | | 53.36 | | 132.11 |
|  | XM_059127599.1 | | XP_058983582.1 | | | 45429.56 | | 54291.57 | | 49719.53 | | 741.36 | | 204.54 | | 254.59 | | 281.67 | | 1327.79 |
|  | XM_059127601.1 | | XP_058983584.1 | | | 22.13 | | 37.41 | | 25.27 | | 0.38 | | 0.19 | | 0.12 | | 0.37 | | 0.42 |
|  | XM_059127602.1 | | XP_058983585.1 | | | 32289.54 | | 34232.51 | | 36967.68 | | 621.18 | | 123.02 | | 136.98 | | 168.09 | | 1140.59 |
|  | XM_059127603.1 | | XP_058983586.1 | | | 2492.63 | | 6306.26 | | 2680.23 | | 23.92 | | 22.02 | | 23.08 | | 36.14 | | 1.36 |
|  | XM_059128787.1 | | XP_058984770.1 | | | 0.05 | | 0.00 | | 0.00 | | 0.00 | | 0.00 | | 0.00 | | 0.00 | | 0.00 |
|  | Mdom_Bit_LysC_342 | | Mdom_Bit_LysC_342 | | | 0.00 | | 0.00 | | 0.00 | | 0.00 | | 0.00 | | 0.00 | | 0.00 | | 0.09 |
|  | Mdom_Bit_LysC_343 | | Mdom_Bit_LysC_343 | | | 0.11 | | 0.00 | | 0.00 | | 0.00 | | 0.00 | | 0.00 | | 0.00 | | 0.00 |
|  | Mdom_Bit_LysC_344 | | Mdom_Bit_LysC_344 | | | 6.66 | | 0.09 | | 0.12 | | 1.81 | | 0 | | 0 | | 0 | | 188.41 |
|  | Mdom_Bit_LysC_345 | | Mdom_Bit_LysC_345 | | | 0 | | 0 | | 0 | | 0 | | 0 | | 0 | | 0 | | 0 |
|  | Mdom_Bit_LysC_347 | | Mdom_Bit_LysC_347 | | | 0.05 | | 0 | | 0 | | 0 | | 0 | | 0 | | 0 | | 0.01 |
|  | XM_059127598.1 | | XP_058983581.1 | | | 3408.83 | | 4572.93 | | 3241.98 | | 42.94 | | 13.73 | | 16.88 | | 23.37 | | 75.02 |
| **Total** |  | |  | | | **329667.47** | | **481706.29** | | **420039.59** | | **15095.05** | | **1779.65** | | **2118.96** | | **2440.44** | | **8610.34** |
| I-type | Mdom_Bit_LysI_238 | | Mdom_Bit_LysI_238 | | | 0.30 | | 0.00 | | 0.00 | | 0.00 | | 0.00 | | 0.00 | | 0.00 | | 0.00 |
|  | Mdom_Bit_LysI_237 | | Mdom_Bit_LysI_237 | | | 0.06 | | 0 | | 0 | | 0.36 | | 0 | | 0 | | 0 | | 0 |
|  | Mdom_Bit_LysI_239 | | Mdom_Bit_LysI_239 | | | 0.61 | | 0.36 | | 1.94 | | 0.76 | | 0.2 | | 0.17 | | 1.68 | | 116.76 |
|  | XM_020037373.2 | | XP_019892932.2 | | | 4.12 | | 1.21 | | 6.04 | | 4.48 | | 2.01 | | 2.33 | | 5.3 | | 314.13 |
| **Total** |  | |  | | | **5.09** | | **1.57** | | **7.98** | | **5.60** | | **2.21** | | **2.50** | | **6.98** | | **430.89** |
|  | |  | |  |  | |  | |  | |  | |  | |  | |  | |  |  |

*Expression was assessed in AM1 and AM2 (first and second portions of the anterior midgut), MM1 and MM2 (first and second portions of the middle midgut), PM1, PM2, and PM3 (first, second, and third portions of the posterior midgut), and CAR (carcass, referring to the larva without the midgut).

**Table S12 -** Expression profile of C-type and I-type lysozyme genes in *Rhodnius prolixus* adults

| **Lysozyme type** | **Transcript ID** | **Protein ID** | **Expression (TPM)*** | | | |
| --- | --- | --- | --- | --- | --- | --- |
|  |  |  | **AM** | **MM** | **PM** | **Car** |
| C-type | RPRC015440-RA | RPRC015440-PA | 19000.43 | 1665.62 | 2237.51 | 471.59 |
|  | RPRC015441-RA | RPRC015441-PA | 248092.13 | 3292.53 | 270.33 | 65.87 |
|  | RPRC015442-RA | RPRC015442-PA | 10.13 | 12.52 | 14.17 | 325.29 |
| **Total** |  |  | **267102.68** | **4970.67** | **2522.01** | **862.74** |
| I-type | RPRC011594-RA | RPRC011594-PA | 1.40 | 0.27 | 0.36 | 196.99 |
|  | Rpro_Bit_LysI_292 | Rpro_Bit_LysI_292 | 0.00 | 0.00 | 0.00 | 0.00 |
| **Total** |  |  | **1.40** | **0.27** | **0.36** | **196.99** |

* Gene expression was evaluated in the anterior (AM), middle (MM), and posterior midgut (PM), as well as in the carcass (CAR), which corresponds to the adult body excluding the head, legs, wings, and digestive tissues.

**Table S13 -** Expression profile of C-type and I-type lysozyme genes in *Tenebrio molitor* larvae

| **Lysozyme type** | **Transcript ID** | **Protein ID** | **Expression (TPM)*** | | | |
| --- | --- | --- | --- | --- | --- | --- |
|  |  |  | **AM** | **MM** | **PM** | **Car** |
| C-type | Tmol_Bit_LysC_423 | Tmol_Bit_LysC_423 | 7322.15 | 2069.07 | 1015.80 | 364.84 |
|  | Tmol_Bit_LysC_424 | Tmol_Bit_LysC_424 | 0.00 | 0.00 | 0.00 | 0.00 |
| **Total** |  |  | **7322.15** | **2069.07** | **1015.80** | **364.84** |
| I-type | TMOL_7.1405.1 | CAH1377254.1 | 0.04 | 0.02 | 0.05 | 2.31 |
|  | TMOL_7.1406.1 | CAH1377255.1 | 0.00 | 0.00 | 0.00 | 0.00 |
|  | TMOL_7.1407.1 | CAH1377256.1 | 0.00 | 0.00 | 0.00 | 5.96 |
|  | TMOL_7.1409.1 | CAH1377258.1 | 5.87 | 1.50 | 2.69 | 324.08 |
|  | TMOL_7.56.1 | CAH1375994.1 | 0.29 | 0.00 | 0.00 | 55.81 |
|  | TMOL_7.57.1 | CAH1375995.1 | 0.92 | 0.00 | 0.00 | 64.25 |
|  | TMOL_7.58.1 | CAH1375996.1 | 6.97 | 0.24 | 1.85 | 560.33 |
|  | Tmol_Bit_LysI_317 | Tmol_Bit_LysI_317 | 0.00 | 0.00 | 0.00 | 83.65 |
|  | Tmol_Bit_LysI_318 | Tmol_Bit_LysI_318 | 0.00 | 0.00 | 0.00 | 0.87 |
|  | Tmol_Bit_LysI_319 | Tmol_Bit_LysI_319 | 8.11 | 10.09 | 12.92 | 1243.10 |
| **Total** |  |  | **22.19** | **11.85** | **17.52** | **2340.36** |

*Gene expression was evaluated in the anterior (AM), middle (MM), and posterior midgut (PM), as well as in the carcass (CAR), which corresponds to the body excluding the digestive tissues

**Table S14 -** Expression profile of C-type and I-type lysozyme genes in *Periplaneta americana* adults

| **Lysozyme type** | **Transcript ID** | **Protein ID** | **Expression (TPM)*** | | | | | | | |
| --- | --- | --- | --- | --- | --- | --- | --- | --- | --- | --- |
|  |  |  | **SG** | **GC** | **FG** | **AM** | **PM** | **HG** | **MT** | **Car** |
| C-type | XM_069817730.1 | XP_069673831.1 | 11.76 | 8.58 | 449.70 | 820.84 | 506.66 | 24.70 | 94.23 | 93.72 |
|  | XM_069817731.1 | XP_069673832.1 | 0.00 | 77.86 | 0.00 | 0.00 | 0.48 | 4.20 | 9.09 | 18.88 |
|  | XM_069825741.1 | XP_069681842.1 | 4585.86 | 228.38 | 14.77 | 19.76 | 14.46 | 3.93 | 3.87 | 1465.70 |
|  | XM_069844692.1 | XP_069700793.1 | 0.00 | 0.00 | 0.00 | 5.53 | 31.75 | 49.38 | 12.57 | 0.54 |
|  | XM_069825743.1 | XP_069681844.1 | 36113.83 | 1226.28 | 92.48 | 19.76 | 17.56 | 44.25 | 16.90 | 11962.62 |
|  | XM_069846431.1 | XP_069702532.1 | 0.42 | 2.57 | 0.26 | 6.32 | 6.77 | 0.25 | 0.77 | 1.99 |
|  | XM_069846432.1 | XP_069702533.1 | 6.05 | 34.01 | 16.39 | 21.21 | 18.99 | 119.16 | 240.74 | 15.76 |
|  | XM_069848274.1 | XP_069704375.1 | 9.74 | 26.31 | 6.53 | 4.92 | 6.62 | 3.95 | 3.27 | 13.56 |
| **Total** |  |  | **40727.67** | **1603.98** | **580.13** | **898.33** | **603.29** | **249.82** | **381.41** | **13572.77** |
| I-type | XM_069819816.1 | XP_069675917.1 | 20.70 | 6.85 | 0.84 | 0.22 | 0.00 | 2.23 | 0.00 | 11.13 |
|  | XM_069828481.1 | XP_069684582.1 | 101.76 | 166.83 | 144.56 | 9.52 | 38.65 | 80.02 | 15.21 | 64.29 |
|  | XM_069828485.1 | XP_069684586.1 | 4.96 | 6.22 | 11.84 | 1.46 | 3.54 | 19.90 | 35.03 | 2.66 |
|  | XM_069828488.1 | XP_069684589.1 | 89.88 | 19.06 | 136.46 | 2.78 | 8.43 | 19.32 | 8.43 | 7.32 |
|  | XM_069828489.1 | XP_069684590.1 | 1.21 | 12.92 | 4.89 | 21.37 | 17.21 | 6.91 | 5.26 | 5.44 |
|  | Pame_Bit_LysI_276 | Pame_Bit_LysI_276 | 0.00 | 0.00 | 0.00 | 0.00 | 0.00 | 0.00 | 0.00 | 0.00 |
|  | Pame_Bit_LysI_277 | Pame_Bit_LysI_277 | 0.00 | 0.00 | 0.00 | 0.00 | 0.00 | 5.23 | 0.00 | 0.00 |
|  | Pame_Bit_LysI_278 | Pame_Bit_LysI_278 | 0.00 | 0.00 | 0.38 | 2.02 | 2.02 | 0.00 | 0.17 | 0.00 |
| **Total** |  |  | **218.50** | **211.86** | **298.97** | **37.37** | **69.85** | **133.61** | **64.08** | **90.85** |
| *Tissues included the gastric cecum (GC), salivary glands (SG), foregut (FG), anterior and posterior midgut (AM and PM), hindgut (HG), Malpighian tubules (MT), and the carcass (CAR), representing the adult body without the head, legs, wings, and dissected gut parts. | | | | | | | | | | |

**Table S15 -** Expression profile of C-type and I-type lysozyme genes in *Spodoptera frugiperda* larvae

| **Lysozyme type** | **Transcript ID** | **Protein ID** | **Expression (TPM)*** | | |
| --- | --- | --- | --- | --- | --- |
|  |  |  | **AM** | **MM** | **PM** |
| C-type | Sfru_Bit_LysC_414 | Sfru_Bit_LysC_414 | 3.22 | 0.19 | 0.73 |
|  | XM_035578087.2 | XP_035433980.2 | 2.88 | 0.20 | 1.79 |
|  | XM_035578414.2 | XP_035434307.1 | 346.27 | 135.73 | 694.51 |
|  | XM_035585417.2 | XP_035441310.1 | 1.62 | 2.40 | 3.64 |
| **Total** |  |  | **353.98** | **138.51** | **700.67** |
| I-type | XM_050693850.1 | XP_050549807.1 | 0.00 | 0.00 | 0.00 |
|  | XM_035588210.2 | XP_035444103.1 | 1022.01 | 36.95 | 136.61 |
|  | XM_050693672.1 | XP_050549629.1 | 0.05 | 0.00 | 0.00 |
|  | Sfru_Bit_LysI_306 | Sfru_Bit_LysI_306 | 0.00 | 0.00 | 0.00 |
|  | Sfru_Bit_LysI_307 | Sfru_Bit_LysI_307 | 0.00 | 0.00 | 0.00 |
| **Total** |  |  | **1022.06** | **36.95** | **136.61** |

*The tissues analyzed included AM, MM, and PM, corresponding to the anterior, middle, and posterior midgut, respectively.

**Table S16 -** Accession numbers, BUSCO completeness scores, and taxonomic lineage information for the insect species and non-insect outgroups analyzed in this study.

| Organism | Accession | Source | BUSCO scores | | | | | | Lineage |
| --- | --- | --- | --- | --- | --- | --- | --- | --- | --- |
|  |  |  | C (%) | S (%) | D (%) | F (%) | M (%) | N |  |
| *Procambarus clarkii* | GCF_020424385.1 | NCBI | 98.2 | 96.2 | 2.0 | 0.9 | 0.9 | 1013 | Arthropoda; Mandibulata; Pancrustacea; Crustacea; Multicrustacea; Malacostraca; Eumalacostraca; Eucarida; Decapoda; Pleocyemata; Astacidea; Astacoidea; Cambaridae; Cambarinae; Procambarus |
| *Eriocheir sinensis* | GCF_024679095.1 | NCBI | 97.8 | 91.2 | 6.6 | 0.3 | 1.9 | 1013 | Arthropoda; Mandibulata; Pancrustacea; Crustacea; Multicrustacea; Malacostraca; Eumalacostraca; Eucarida; Decapoda; Pleocyemata; Brachyura; Eubrachyura; Thoracotremata; Grapsoidea; Varunidae; Eriocheir |
| *Scylla paramamosain* | GCF_035594125.1 | NCBI | 98.3 | 97.3 | 1.0 | 0.2 | 1.5 | 1013 | Arthropoda; Mandibulata; Pancrustacea; Crustacea; Multicrustacea; Malacostraca; Eumalacostraca; Eucarida; Decapoda; Pleocyemata; Brachyura; Eubrachyura; Heterotremata; Portunoidea; Portunidae; Portuninae; Scylla |
| *Folsomia candida* | GCF_002217175.1 | NCBI | 97.9 | 96.0 | 1.9 | 0.4 | 1.7 | 1013 | Arthropoda; Mandibulata; Pancrustacea; Hexapoda; Collembola; Entomobryomorpha; Isotomoidea; Isotomidae; Proisotominae; Folsomia |
| *Ischnura elegans* | GCF_921293095.1 | NCBI | 99.1 | 97.6 | 1.5 | 0.0 | 0.9 | 1367 | Arthropoda; Mandibulata; Pancrustacea; Hexapoda; Insecta; Dicondylia; Pterygota; Palaeoptera; Odonata; Zygoptera; Coenagrionoidea; Coenagrionidae; Ischnura; Ischnura elegans |
| *Hetaerina americana* | GCF_022747635.1 | NCBI | 98.9 | 97.0 | 1.9 | 0.2 | 0.9 | 1367 | Arthropoda; Mandibulata; Pancrustacea; Hexapoda; Insecta; Dicondylia; Pterygota; Palaeoptera; Odonata; Zygoptera; Calopterygoidea; Calopterygidae; Hetaerina |
| *Cloeon dipterum* | GCF_949628265.1 | NCBI | 98.0 | 96.3 | 1.7 | 0.4 | 1.6 | 1367 | Arthropoda; Mandibulata; Pancrustacea; Hexapoda; Insecta; Dicondylia; Pterygota; Palaeoptera; Ephemeroptera; Pisciforma; Baetidae; Cloeon |
| *Neocloeon triangulifer* | GCF_031216515.1 | NCBI | 97.8 | 96.0 | 1.8 | 0.4 | 1.8 | 1367 | Arthropoda; Mandibulata; Pancrustacea; Hexapoda; Insecta; Dicondylia; Pterygota; Palaeoptera; Ephemeroptera; Pisciforma; Baetidae; Neocloeon; Neocloeon triangulifer |
| *Gryllus longicercus* | GCA_038098605.1 | NCBI | 95.0 | 88.1 | :6.9 | 0.4 | 4.6 | 1367 | Arthropoda; Mandibulata; Pancrustacea; Hexapoda ; Insecta ; Dicondylia ; Pterygota; Neoptera; Polyneoptera; Orthoptera; Ensifera; Gryllidea; Grylloidea ; Gryllidae ; Gryllinae ; Gryllus |
| *Anabrus simplex* | GCF_040414725.1 | NCBI | 99.1 | 95.8 | 3.3 | 0.1 | 0.8 | 1367 | Arthropoda ; Mandibulata ; Pancrustacea ; Hexapoda ; Insecta ; Dicondylia ; Pterygota ; Neoptera ; Polyneoptera ; Orthoptera ; Ensifera ; Tettigoniidea ; Tettigonioidea ; Tettigoniidae ; Tettigoniinae ; Anabrus |
| *Bacillus rossius redtenbacheri* | GCF_032445375.1 | NCBI | 97.4 | 96.4 | 1.0 | 0.6 | 2.0 | 1367 | Arthropoda; Mandibulata; Pancrustacea; Hexapoda; Insecta; Dicondylia; Pterygota; Neoptera; Polyneoptera; Phasmatodea; Verophasmatodea; Areolatae; Bacilloidea; Bacillidae; Bacillinae; Bacillini; Bacillus; Bacillus rossius; Bacillus rossius redtenbacheri |
| *Blattella germanica* | GCA_003018175.1 | NCBI | 83.5 | 81.6 | 1.9 | 10.2 | 6.3 | 1367 | Arthropoda; Mandibulata; Pancrustacea; Hexapoda; Insecta; Dicondylia; Pterygota; Neoptera; Polyneoptera; Dictyoptera; Blattodea; Blaberoidea; Blattellidae; Blattella; Blattella germanica |
| *Periplaneta americana* | GCF_040183065.1 | NCBI | 97.9 | 96.6 | 1.3 | 0.2 | 1.9 | 1367 | Arthropoda ; Mandibulata ; Pancrustacea ; Hexapoda ; Insecta ; Dicondylia ; Pterygota ; Neoptera ; Polyneoptera ; Dictyoptera ; Blattodea ; Blattoidea ; Blattidae ; Blattinae ; Periplaneta |
| *Zootermopsis nevadensis* | GCF_000696155.1 | NCBI | 98.9 | 98.0 | 0.9 | 0.4 | 0.7 | 1367 | Arthropoda; Mandibulata; Pancrustacea; Hexapoda; Insecta; Dicondylia; Pterygota; Neoptera; Polyneoptera; Dictyoptera; Blattodea; Blattoidea; Termitoidae; Termopsidae; Termopsinae; Termopsini; Zootermopsis; Zootermopsis nevadensis |
| *Coptotermes formosanus* | GCA_013340265.1 | NCBI | 89.7 | 87.9 | 1.8 | 5.4 | 4.9 | 1367 | Arthropoda; Mandibulata; Pancrustacea; Hexapoda; Insecta; Dicondylia; Pterygota; Neoptera; Polyneoptera; Dictyoptera; Blattodea; Blattoidea; Termitoidae; Rhinotermitidae; Coptotermitinae; Coptotermes; Coptotermes formosanus |
| *Cryptotermes secundus* | GCF_002891405.2 | NCBI | 98.3 | 96.3 | 2.0 | 0.5 | 1.2 | 1367 | Arthropoda; Mandibulata; Pancrustacea; Hexapoda; Insecta; Dicondylia; Pterygota; Neoptera; Polyneoptera; Dictyoptera; Blattodea; Blattoidea; Termitoidae; Kalotermitidae; Cryptotermitinae; Cryptotermes; Cryptotermes secundus |
| *Frankliniella occidentalis* | GCF_000697945.3 | NCBI | 99.3 | 97.9 | 1.4 | 0.4 | 0.3 | 1367 | Arthropoda; Mandibulata; Pancrustacea; Hexapoda; Insecta; Dicondylia; Pterygota; Neoptera; Paraneoptera; Thysanoptera; Terebrantia; Thripoidea; Thripidae; Thripinae; Frankliniella; Frankliniella occidentalis |
| *Thrips palmi* | GCF_012932325.1 | NCBI | 97.6 | 96.6 | 1.0 | 0.3 | 2.1 | 1367 | Arthropoda; Mandibulata; Pancrustacea; Hexapoda; Insecta; Dicondylia; Pterygota; Neoptera; Paraneoptera; Thysanoptera; Terebrantia; Thripoidea; Thripidae; Thripinae; Thrips; Thrips palmi |
| *Homalodisca vitripennis* | GCF_021130785.1 | NCBI | 91.3 | 80.4 | 10.9 | 2.3 | 6.4 | 1367 | Arthropoda; Mandibulata; Pancrustacea; Hexapoda; Insecta; Dicondylia; Pterygota; Neoptera; Paraneoptera; Hemiptera; Auchenorrhyncha; Cicadomorpha; Membracoidea; Cicadellidae; Cicadellinae; Proconiini; Homalodisca; Homalodisca vitripennis |
| *Laodelphax striatellus* | GCA_003335185.2 | NCBI | 93.6 | 90.2 | 3.4 | 2.6 | 3.8 | 1367 | Arthropoda; Mandibulata; Pancrustacea; Hexapoda; Insecta; Dicondylia; Pterygota; Neoptera; Paraneoptera; Hemiptera; Auchenorrhyncha; Fulgoromorpha; Fulgoroidea; Delphacidae; Criomorphinae; Laodelphax; Laodelphax striatellus |
| *Nilaparvata lugens* | GCF_014356525.2 | NCBI | 96.2 | 89.5 | 6.7 | 1.4 | 2.4 | 1367 | Arthropoda; Mandibulata; Pancrustacea; Hexapoda; Insecta; Dicondylia; Pterygota; Neoptera; Paraneoptera; Hemiptera; Auchenorrhyncha; Fulgoromorpha; Fulgoroidea; Delphacidae; Delphacinae; Nilaparvata; Nilaparvata lugens |
| *Halyomorpha halys* | GCF_000696795.2 | NCBI | 99.3 | 97.3 | 2.0 | 0.2 | 0.5 | 1367 | Arthropoda; Mandibulata; Pancrustacea; Hexapoda; Insecta; Dicondylia; Pterygota; Neoptera; Paraneoptera; Hemiptera; Prosorrhyncha; Heteroptera; Euheteroptera; Neoheteroptera; Panheteroptera; Pentatomomorpha; Pentatomoidea; Pentatomidae; Pentatominae; Halyomorpha; Halyomorpha halys |
| *Rhodnius prolixus* | RprolixusCDC | VectorBase | 88.3 | 87.2 | 1.1 | 5.9 | 5.8 | 1367 | Arthropoda; Mandibulata; Pancrustacea; Hexapoda; Insecta; Dicondylia; Pterygota; Neoptera; Paraneoptera; Hemiptera; Prosorrhyncha; Heteroptera; Euheteroptera; Neoheteroptera; Panheteroptera; Cimicomorpha; Reduvioidea; Reduviidae; Triatominae; Rhodnius; Rhodnius prolixus |
| *Apolygus lucorum* | GCA_009739505.2 | NCBI | 93.5 | 88.3 | 5.2 | 2.3 | 4.2 | 1367 | Arthropoda; Mandibulata; Pancrustacea; Hexapoda; Insecta; Dicondylia; Pterygota; Neoptera; Paraneoptera; Hemiptera; Prosorrhyncha; Heteroptera; Euheteroptera; Neoheteroptera; Panheteroptera; Cimicomorpha; Cimicoidea; Miridae; Mirinae; Mirini; Apolygus; Apolygus lucorum |
| *Cimex lectularius* | GCF_000648675.2 | NCBI | 99.7 | 98.5 | 1.2 | 0.3 | 0.0 | 1367 | Arthropoda; Mandibulata; Pancrustacea; Hexapoda; Insecta; Dicondylia; Pterygota; Neoptera; Paraneoptera; Hemiptera; Prosorrhyncha; Heteroptera; Euheteroptera; Neoheteroptera; Panheteroptera; Cimicomorpha; Cimicoidea; Cimicidae; Cimex; Cimex lectularius |
| *Bemisia tabaci* | GCF_001854935.1 | NCBI | 98.6 | 95.9 | 2.7 | 0.2 | 1.2 | 1367 | Arthropoda; Mandibulata; Pancrustacea; Hexapoda; Insecta; Dicondylia; Pterygota; Neoptera; Paraneoptera; Hemiptera; Sternorrhyncha; Aleyrodoidea; Aleyrodidae; Aleyrodinae; Bemisia; Bemisia tabaci |
| *Planococcus citri* | GCF_950023065.1 | NCBI | 96.5 | 88.4 | 8.1 | 0.4 | 3.1 | 1367 | Arthropoda; Mandibulata; Pancrustacea; Hexapoda; Insecta; Dicondylia; Pterygota; Neoptera; Paraneoptera; Hemiptera; Sternorrhyncha; Coccoidea; Pseudococcidae; Planococcus; Planococcus citri |
| *Adelges cooleyi* | GCF_023614345.1 | NCBI | 96.8 | 92.6 | 4.2 | 0.3 | 2.9 | 1367 | Arthropoda; Mandibulata; Pancrustacea; Hexapoda; Insecta; Dicondylia; Pterygota; Neoptera; Paraneoptera; Hemiptera; Sternorrhyncha; Aphidomorpha; Phylloxeroidea; Adelgidae; Adelges; Gilletteella; Adelges cooleyi |
| *Daktulosphaira vitifoliae* | GCF_025091365.1 | NCBI | 97.3 | 93.9 | 3.4 | 0.3 | 2.4 | 1367 | Arthropoda; Mandibulata; Pancrustacea; Hexapoda; Insecta; Dicondylia; Pterygota; Neoptera; Paraneoptera; Hemiptera; Sternorrhyncha; Aphidomorpha; Phylloxeroidea; Phylloxeridae; Daktulosphaira; Daktulosphaira vitifoliae |
| *Sipha flava* | GCF_003268045.1 | NCBI | 94.6 | 92.0 | 2.6 | 0.8 | 4.6 | 1367 | Arthropoda; Mandibulata; Pancrustacea; Hexapoda; Insecta; Dicondylia; Pterygota; Neoptera; Paraneoptera; Hemiptera; Sternorrhyncha; Aphidomorpha; Aphidoidea; Aphididae; Chaitophorinae; Sipha; Sipha flava |
| *Acyrthosiphon pisum* | GCF_005508785.2 | NCBI | 96.2 | 89.8 | 6.4 | 0.8 | 3.0 | 1367 | Arthropoda; Mandibulata; Pancrustacea; Hexapoda; Insecta; Dicondylia; Pterygota; Neoptera; Paraneoptera; Hemiptera; Sternorrhyncha; Aphidomorpha; Aphidoidea; Aphididae; Aphidinae; Macrosiphini; Acyrthosiphon; Acyrthosiphon pisum |
| *Myzus persicae* | GCF_001856785.1 | NCBI | 96.7 | 92.9 | 3.8 | 0.6 | 2.7 | 1367 | Arthropoda; Mandibulata; Pancrustacea; Hexapoda; Insecta; Dicondylia; Pterygota; Neoptera; Paraneoptera; Hemiptera; Sternorrhyncha; Aphidomorpha; Aphidoidea; Aphididae; Aphidinae; Macrosiphini; Myzus; Myzus persicae |
| *Melanaphis sacchari* | GCF_002803265.2 | NCBI | 97.1 | 94.7 | 2.4 | 0.5 | 2.4 | 1367 | Arthropoda; Mandibulata; Pancrustacea; Hexapoda; Insecta; Dicondylia; Pterygota; Neoptera; Paraneoptera; Hemiptera; Sternorrhyncha; Aphidomorpha; Aphidoidea; Aphididae; Aphidinae; Aphidini; Melanaphis; Melanaphis sacchari |
| *Aphis craccivora* | GCA_009835225.1 | NCBI | 94.2 | 90.6 | 3.6 | 2.0 | 3.8 | 1367 | Arthropoda; Mandibulata; Pancrustacea; Hexapoda; Insecta; Dicondylia; Pterygota; Neoptera; Paraneoptera; Hemiptera; Sternorrhyncha; Aphidomorpha; Aphidoidea; Aphididae; Aphidinae; Aphidini; Aphis; Aphis; Aphis craccivora |
| *Aphis gossypii* | GCF_020184175.1 | NCBI | 96.7 | 92.2 | 4.5 | 0.5 | 2.8 | 1367 | Arthropoda; Mandibulata; Pancrustacea; Hexapoda; Insecta; Dicondylia; Pterygota; Neoptera; Paraneoptera; Hemiptera; Sternorrhyncha; Aphidomorpha; Aphidoidea; Aphididae; Aphidinae; Aphidini; Aphis; Aphis; Aphis gossypii |
| *Rhopalosiphum maidis* | GCF_003676215.2 | NCBI | 97.2 | 91.9 | 5.3 | 0.4 | 2.4 | 1367 | Arthropoda; Mandibulata; Pancrustacea; Hexapoda; Insecta; Dicondylia; Pterygota; Neoptera; Paraneoptera; Hemiptera; Sternorrhyncha; Aphidomorpha; Aphidoidea; Aphididae; Aphidinae; Aphidini; Rhopalosiphum; Rhopalosiphum maidis |
| *Rhopalosiphum padi* | GCF_020882245.1 | NCBI | 96.9 | 93.6 | 3.3 | 0.4 | 2.7 | 1367 | Arthropoda; Mandibulata; Pancrustacea; Hexapoda; Insecta; Dicondylia; Pterygota; Neoptera; Paraneoptera; Hemiptera; Sternorrhyncha; Aphidomorpha; Aphidoidea; Aphididae; Aphidinae; Aphidini; Rhopalosiphum; Rhopalosiphum padi complex; Rhopalosiphum padi |
| *Pediculus humanus corporis* | GCF_000006295.1 | NCBI | 95.7 | 95.3 | 0.4 | 2.1 | 2.2 | 1367 | Arthropoda; Mandibulata; Pancrustacea; Hexapoda; Insecta; Dicondylia; Pterygota; Neoptera; Paraneoptera; Psocodea; Phthiraptera; Anoplura; Pediculidae; Pediculus; Pediculus humanus; Pediculus humanus corporis |
| *Athalia rosae* | GCF_917208135.1 | NCBI | 99.7 | 99.6 | 0.1 | 0.1 | 0.2 | 1367 | Arthropoda; Mandibulata; Pancrustacea; Hexapoda; Insecta; Dicondylia; Pterygota; Neoptera; Endopterygota; Hymenoptera; Tenthredinoidea; Athaliidae; Athalia; Athalia rosae |
| *Diprion similis* | GCF_021155765.1 | NCBI | 95.4 | 94.7 | 0.7 | 0.3 | 4.3 | 1367 | Arthropoda; Mandibulata; Pancrustacea; Hexapoda; Insecta; Dicondylia; Pterygota; Neoptera; Endopterygota; Hymenoptera; Tenthredinoidea; Diprionidae; Diprioninae; Diprion |
| *Neodiprion fabricii* | GCF_021155785.1 | NCBI | 99.5 | 98.8 | 0.7 | 0.1 | 0.4 | 1367 | Arthropoda; Mandibulata; Pancrustacea; Hexapoda; Insecta; Dicondylia; Pterygota; Neoptera; Endopterygota; Hymenoptera; Tenthredinoidea; Diprionidae; Diprioninae; Neodiprion; Neodiprion fabricii |
| *Neodiprion virginianus* | GCF_021901495.1 | NCBI | 99.5 | 98.8 | 0.7 | 0.1 | 0.4 | 1367 | Arthropoda; Mandibulata; Pancrustacea; Hexapoda; Insecta; Dicondylia; Pterygota; Neoptera; Endopterygota; Hymenoptera; Tenthredinoidea; Diprionidae; Diprioninae; Neodiprion; Neodiprion virginianus |
| *Neodiprion lecontei* | GCF_021901455.1 | NCBI | 99.7 | 99.0 | 0.7 | 0.1 | 0.2 | 1367 | Arthropoda; Mandibulata; Pancrustacea; Hexapoda; Insecta; Dicondylia; Pterygota; Neoptera; Endopterygota; Hymenoptera; Tenthredinoidea; Diprionidae; Diprioninae; Neodiprion; Neodiprion |
| *Neodiprion pinetum* | GCF_021155775.1 | NCBI | 99.5 | 98.8 | 0.7 | 0.1 | 0.4 | 1367 | Arthropoda; Mandibulata; Pancrustacea; Hexapoda; Insecta; Dicondylia; Pterygota; Neoptera; Endopterygota; Hymenoptera; Tenthredinoidea; Diprionidae; Diprioninae; Neodiprion; Neodiprion pinetum |
| *Cephus cinctus* | GCF_000341935.2 | NCBI | 99.6 | 98.8 | 0.8 | 0.1 | 0.3 | 1367 | Arthropoda; Mandibulata; Pancrustacea; Hexapoda; Insecta; Dicondylia; Pterygota; Neoptera; Endopterygota; Hymenoptera; Cephoidea; Cephidae; Cephus |
| *Orussus abietinus* | GCF_000612105.2 | NCBI | 98.5 | 97.7 | 0.8 | 0.4 | 1.1 | 1367 | Arthropoda; Mandibulata; Pancrustacea; Hexapoda; Insecta; Dicondylia; Pterygota; Neoptera; Endopterygota; Hymenoptera; Orussoidea; Orussidae; Orussus |
| *Belonocnema kinseyi* | GCF_010883055.1 | NCBI | 97.0 | 95.9 | 1.1 | 1.5 | 1.5 | 1367 | Arthropoda; Mandibulata; Pancrustacea; Hexapoda; Insecta; Dicondylia; Pterygota; Neoptera; Endopterygota; Hymenoptera; Apocrita; Proctotrupomorpha; Cynipoidea; Cynipidae; Cynipinae; Cynipini; Belonocnema |
| *Leptopilina boulardi* | GCF_019393585.1 | NCBI | 99.0 | 98.5 | 0.5 | 0.1 | 0.9 | 1367 | Arthropoda; Mandibulata; Pancrustacea; Hexapoda; Insecta; Dicondylia; Pterygota; Neoptera; Endopterygota; Hymenoptera; Apocrita; Proctotrupomorpha; Cynipoidea; Figitidae; Eucoilinae; Leptopilina; Leptopilina boulardi |
| *Leptopilina heterotoma* | GCF_015476425.1 | NCBI | 99.0 | 98.1 | 0.9 | 0.3 | 0.7 | 1367 | Arthropoda; Mandibulata; Pancrustacea; Hexapoda; Insecta; Dicondylia; Pterygota; Neoptera; Endopterygota; Hymenoptera; Apocrita; Proctotrupomorpha; Cynipoidea; Figitidae; Eucoilinae; Leptopilina; Leptopilina heterotoma |
| *Trichogramma pretiosum* | GCF_000599845.2 | NCBI | 98.7 | 95.4 | 3.3 | 0.1 | 1.2 | 1367 | Arthropoda; Mandibulata; Pancrustacea; Hexapoda; Insecta; Dicondylia; Pterygota; Neoptera; Endopterygota; Hymenoptera; Apocrita; Proctotrupomorpha; Chalcidoidea; Trichogrammatidae; Trichogramma; Trichogramma pretiosum |
| *Copidosoma floridanum* | GCF_000648655.2 | NCBI | 97.8 | 94.7 | 3.1 | 0.6 | 1.6 | 1367 | Arthropoda; Mandibulata; Pancrustacea; Hexapoda; Insecta; Dicondylia; Pterygota; Neoptera; Endopterygota; Hymenoptera; Apocrita; Proctotrupomorpha; Chalcidoidea; Encyrtidae; Encyrtinae; Copidosoma; Copidosoma floridanum |
| *Phymastichus coffea* | GCF_024137745.1 | NCBI | 97.5 | 95.4 | 2.1 | 0.5 | 2.0 | 1367 | Arthropoda; Mandibulata; Pancrustacea; Hexapoda; Insecta; Dicondylia; Pterygota; Neoptera; Endopterygota; Hymenoptera; Apocrita; Proctotrupomorpha; Chalcidoidea; Eulophidae; Tetrastichinae; Phymastichus; Phymastichus coffea |
| *Ceratosolen solmsi marchali* | GCF_000503995.2 | NCBI | 97.9 | 97.4 | 0.5 | 0.8 | 1.3 | 1367 | Arthropoda; Mandibulata; Pancrustacea; Hexapoda; Insecta; Dicondylia; Pterygota; Neoptera; Endopterygota; Hymenoptera; Apocrita; Proctotrupomorpha; Chalcidoidea; Agaonidae; Agaoninae; Ceratosolen; Ceratosolen solmsi; Ceratosolen solmsi marchali |
| *Nasonia vitripennis* | GCF_009193385.2 | NCBI | 98.1 | 96.6 | 1.5 | 0.3 | 1.6 | 1367 | Arthropoda; Mandibulata; Pancrustacea; Hexapoda; Insecta; Dicondylia; Pterygota; Neoptera; Endopterygota; Hymenoptera; Apocrita; Proctotrupomorpha; Chalcidoidea; Pteromalidae; Pteromalinae; Nasonia; Nasonia vitripennis |
| *Venturia canescens* | GCF_019457755.1 | NCBI | 99.5 | 98.3 | 1.2 | 0.2 | 0.3 | 1367 | Arthropoda; Mandibulata; Pancrustacea; Hexapoda; Insecta; Dicondylia; Pterygota; Neoptera; Endopterygota; Hymenoptera; Apocrita; Ichneumonoidea; Ichneumonidae; Campopleginae; Dusona group; Venturia; Venturia canescens |
| *Aphidius gifuensis* | GCF_014905175.1 | NCBI | 98.6 | 92.8 | 5.8 | 0.1 | 1.3 | 1367 | Arthropoda; Mandibulata; Pancrustacea; Hexapoda; Insecta; Dicondylia; Pterygota; Neoptera; Endopterygota; Hymenoptera; Apocrita; Ichneumonoidea; Braconidae; Aphidiinae; Aphidius |
| *Fopius arisanus* | GCF_000806365.1 | NCBI | 98.4 | 97.8 | 0.6 | 0.4 | 1.2 | 1367 | Arthropoda; Mandibulata; Pancrustacea; Hexapoda; Insecta; Dicondylia; Pterygota; Neoptera; Endopterygota; Hymenoptera; Apocrita; Ichneumonoidea; Braconidae; Opiinae; Fopius; Fopius arisanus |
| *Diachasma alloeum* | GCF_001412515.2 | NCBI | 99.0 | 98.3 | 0.7 | 0.1 | 0.9 | 1367 | Arthropoda; Mandibulata; Pancrustacea; Hexapoda; Insecta; Dicondylia; Pterygota; Neoptera; Endopterygota; Hymenoptera; Apocrita; Ichneumonoidea; Braconidae; Opiinae; Diachasma; Diachasma alloeum |
| *Diachasmimorpha longicaudata* | GCF_034640455.1 | NCBI | 99.4 | 98.2 | 1.2 | 0.1 | 0.5 | 1367 | Arthropoda; Mandibulata; Pancrustacea; Hexapoda; Insecta; Dicondylia; Pterygota; Neoptera; Endopterygota; Hymenoptera; Apocrita; Ichneumonoidea; Braconidae; Opiinae; Diachasmimorpha |
| *Chelonus insularis* | GCF_013357705.1 | NCBI | 99.2 | 98.3 | 0.9 | 0.1 | 0.7 | 1367 | Arthropoda; Mandibulata; Pancrustacea; Hexapoda; Insecta; Dicondylia; Pterygota; Neoptera; Endopterygota; Hymenoptera; Apocrita; Ichneumonoidea; Braconidae; Cheloninae; Chelonus; Chelonus insularis |
| *Cotesia glomerata* | GCF_020080835.1 | NCBI | 99.2 | 97.4 | 1.8 | 0.0 | 0.8 | 1367 | Arthropoda; Mandibulata; Pancrustacea; Hexapoda; Insecta; Dicondylia; Pterygota; Neoptera; Endopterygota; Hymenoptera; Apocrita; Ichneumonoidea; Braconidae; Microgastrinae; Cotesia; Cotesia glomerata |
| *Microplitis demolitor* | GCF_026212275.2 | NCBI | 99.6 | 98.1 | 1.5 | 0.0 | 0.4 | 1367 | Arthropoda; Mandibulata; Pancrustacea; Hexapoda; Insecta; Dicondylia; Pterygota; Neoptera; Endopterygota; Hymenoptera; Apocrita; Ichneumonoidea; Braconidae; Microgastrinae; Microplitis; |
| *Microplitis mediator* | GCF_029852145.1 | NCBI | 99.5 | 97.5 | 2.0 | 0.0 | 0.5 | 1367 | Arthropoda; Mandibulata; Pancrustacea; Hexapoda; Insecta; Dicondylia; Pterygota; Neoptera; Endopterygota; Hymenoptera; Apocrita; Ichneumonoidea; Braconidae; Microgastrinae; Microplitis; Microplitis mediator |
| *Polistes dominula* | GCF_001465965.1 | NCBI | 99.6 | 98.8 | 0.8 | 0.0 | 0.4 | 1367 | Arthropoda; Mandibulata; Pancrustacea; Hexapoda; Insecta; Dicondylia; Pterygota; Neoptera; Endopterygota; Hymenoptera; Apocrita; Aculeata; Vespoidea; Vespidae; Polistinae; Polistini; Polistes; Polistes dominula |
| *Polistes canadensis* | GCF_001313835.1 | NCBI | 99.2 | 98.5 | 0.7 | 0.1 | 0.7 | 1367 | Arthropoda; Mandibulata; Pancrustacea; Hexapoda; Insecta; Dicondylia; Pterygota; Neoptera; Endopterygota; Hymenoptera; Apocrita; Aculeata; Vespoidea; Vespidae; Polistinae; Polistini; Polistes; Polistes canadensis |
| *Polistes fuscatus* | GCF_010416935.1 | NCBI | 99.0 | 97.8 | 1.2 | 0.1 | 0.9 | 1367 | Arthropoda; Mandibulata; Pancrustacea; Hexapoda; Insecta; Dicondylia; Pterygota; Neoptera; Endopterygota; Hymenoptera; Apocrita; Aculeata; Vespoidea; Vespidae; Polistinae; Polistini; Polistes; Polistes fuscatus |
| *Vespula pensylvanica* | GCF_014466175.1 | NCBI | 99.2 | 98.6 | 0.6 | 0.2 | 0.6 | 1367 | Arthropoda; Mandibulata; Pancrustacea; Hexapoda; Insecta; Dicondylia; Pterygota; Neoptera; Endopterygota; Hymenoptera; Apocrita; Aculeata; Vespoidea; Vespidae; Vespinae; Vespula; Vespula pensylvanica |
| *Vespula vulgaris* | GCF_905475345.1 | NCBI | 99.1 | 98.3 | 0.8 | 0.0 | 0.9 | 1367 | Arthropoda; Mandibulata; Pancrustacea; Hexapoda; Insecta; Dicondylia; Pterygota; Neoptera; Endopterygota; Hymenoptera; Apocrita; Aculeata; Vespoidea; Vespidae; Vespinae; Vespula; Vespula vulgaris |
| *Vespa velutina* | GCF_912470025.1 | NCBI | 98.2 | 97.2 | 1.0 | 0.1 | 1.7 | 1367 | Arthropoda; Mandibulata; Pancrustacea; Hexapoda; Insecta; Dicondylia; Pterygota; Neoptera; Endopterygota; Hymenoptera; Apocrita; Aculeata; Vespoidea; Vespidae; Vespinae; Vespa; Vespa velutina |
| *Vespa crabro* | GCF_910589235.1 | NCBI | 99.5 | 98.5 | 1.0 | 0.1 | 0.4 | 1367 | Arthropoda; Mandibulata; Pancrustacea; Hexapoda; Insecta; Dicondylia; Pterygota; Neoptera; Endopterygota; Hymenoptera; Apocrita; Aculeata; Vespoidea; Vespidae; Vespinae; Vespa; Vespa crabro |
| *Vespa mandarinia* | GCF_014083535.2 | NCBI | 99.1 | 98.1 | 1.0 | 0.1 | 0.8 | 1367 | Arthropoda; Mandibulata; Pancrustacea; Hexapoda; Insecta; Dicondylia; Pterygota; Neoptera; Endopterygota; Hymenoptera; Apocrita; Aculeata; Vespoidea; Vespidae; Vespinae; Vespa; Vespa mandarinia |
| *Ampulex compressa* | GCA_038496175.1 | NCBI | 96.5 | 96.1 | 0.4 | 1.4 | 2.1 | 1367 | Arthropoda; Mandibulata; Pancrustacea; Hexapoda; Insecta; Dicondylia; Pterygota; Neoptera; Endopterygota; Hymenoptera; Apocrita; Aculeata; Apoidea; Ampulicidae; Ampulicini; Ampulex; Ampulex compressa |
| *Colletes gigas* | GCF_013123115.1 | NCBI | 99.2 | 97.7 | 1.5 | 0.2 | 0.6 | 1367 | Arthropoda; Mandibulata; Pancrustacea; Hexapoda; Insecta; Dicondylia; Pterygota; Neoptera; Endopterygota; Hymenoptera; Apocrita; Aculeata; Apoidea; Anthophila; Colletidae; Colletinae; Colletes; Colletes gigas |
| *Hylaeus anthracinus* | GCF_026225885.1 | NCBI | 99.1 | 97.7 | 1.4 | 0.1 | 0.8 | 1367 | Arthropoda; Mandibulata; Pancrustacea; Hexapoda; Insecta; Dicondylia; Pterygota; Neoptera; Endopterygota; Hymenoptera; Apocrita; Aculeata; Apoidea; Anthophila; Colletidae; Hylaeinae; Hylaeus; Nesoprosopis; Hylaeus anthracinus |
| *Hylaeus volcanicus* | GCF_026283585.1 | NCBI | 99.2 | 98.9 | 0.3 | 0.2 | 0.6 | 1367 | Arthropoda; Mandibulata; Pancrustacea; Hexapoda; Insecta; Dicondylia; Pterygota; Neoptera; Endopterygota; Hymenoptera; Apocrita; Aculeata; Apoidea; Anthophila; Colletidae; Hylaeinae; Hylaeus; Nesoprosopis; Hylaeus volcanicus |
| *Dufourea novaeangliae* | GCF_001272555.1 | NCBI | 99.3 | 98.8 | 0.5 | 0.1 | 0.6 | 1367 | Arthropoda; Mandibulata; Pancrustacea; Hexapoda; Insecta; Dicondylia; Pterygota; Neoptera; Endopterygota; Hymenoptera; Apocrita; Aculeata; Apoidea; Anthophila; Halictidae; Rophitinae; Dufourea; Dufourea novaeangliae |
| *Megalopta genalis* | GCF_011865705.1 | NCBI | 97.8 | 94.9 | 2.9 | 0.1 | 2.1 | 1367 | Arthropoda; Mandibulata; Pancrustacea; Hexapoda; Insecta; Dicondylia; Pterygota; Neoptera; Endopterygota; Hymenoptera; Apocrita; Aculeata; Apoidea; Anthophila; Halictidae; Halictinae; Augochlorini; Megalopta; Megalopta genalis |
| *Nomia melanderi* | GCF_003710045.2 | NCBI | 98.5 | 98.0 | 0.5 | 0.4 | 1.1 | 1367 | Arthropoda; Mandibulata; Pancrustacea; Hexapoda; Insecta; Dicondylia; Pterygota; Neoptera; Endopterygota; Hymenoptera; Apocrita; Aculeata; Apoidea; Anthophila; Halictidae; Nomiinae; Nomia; Acunomia; Nomia melanderi |
| *Megachile rotundata* | GCF_000220905.1 | NCBI | 98.9 | 98.5 | 0.4 | 0.1 | 1.0 | 1367 | Arthropoda; Mandibulata; Pancrustacea; Hexapoda; Insecta; Dicondylia; Pterygota; Neoptera; Endopterygota; Hymenoptera; Apocrita; Aculeata; Apoidea; Anthophila; Megachilidae; Megachilinae; Megachilini; Megachile; Megachile rotundata |
| *Osmia bicornis bicornis* | GCF_907164935.1 | NCBI | 99.2 | 98.8 | 0.4 | 0.3 | 0.5 | 1367 | Arthropoda; Mandibulata; Pancrustacea; Hexapoda; Insecta; Dicondylia; Pterygota; Neoptera; Endopterygota; Hymenoptera; Apocrita; Aculeata; Apoidea; Anthophila; Megachilidae; Megachilinae; Osmiini; Osmia; Osmia bicornis; Osmia bicornis bicornis |
| *Osmia lignaria* | GCF_012274295.1 | NCBI | 98.8 | 98.2 | 0.6 | 0.1 | 1.1 | 1367 | Arthropoda; Mandibulata; Pancrustacea; Hexapoda; Insecta; Dicondylia; Pterygota; Neoptera; Endopterygota; Hymenoptera; Apocrita; Aculeata; Apoidea; Anthophila; Megachilidae; Megachilinae; Osmiini; Osmia; Osmia lignaria |
| *Ceratina calcarata* | GCF_001652005.2 | NCBI | 98.0 | 95.5 | 2.5 | 1.0 | 1.0 | 1367 | Arthropoda; Mandibulata; Pancrustacea; Hexapoda; Insecta; Dicondylia; Pterygota; Neoptera; Endopterygota; Hymenoptera; Apocrita; Aculeata; Apoidea; Anthophila; Apidae; Xylocopinae; Ceratinini; Ceratina; Zadontomerus; Ceratina calcarata |
| *Eufriesea mexicana* | GCF_001483705.2 | NCBI | 98.8 | 96.7 | 2.1 | 0.2 | 1.0 | 1367 | Arthropoda; Mandibulata; Pancrustacea; Hexapoda; Insecta; Dicondylia; Pterygota; Neoptera; Endopterygota; Hymenoptera; Apocrita; Aculeata; Apoidea; Anthophila; Apidae; Apinae; Euglossini; Eufriesea; Eufriesea mexicana |
| *Bombus affinis* | GCF_024516045.1 | NCBI | 99.6 | 99.0 | 0.6 | 0.1 | 0.3 | 1367 | Arthropoda; Mandibulata; Pancrustacea; Hexapoda; Insecta; Dicondylia; Pterygota; Neoptera; Endopterygota; Hymenoptera; Apocrita; Aculeata; Apoidea; Anthophila; Apidae; Apinae; Bombini; Bombus; Bombus; Bombus affinis |
| *Bombus terrestris* | GCF_910591885.1 | NCBI | 99.4 | 98.7 | 0.7 | 0.1 | 0.5 | 1367 | Arthropoda; Mandibulata; Pancrustacea; Hexapoda; Insecta; Dicondylia; Pterygota; Neoptera; Endopterygota; Hymenoptera; Apocrita; Aculeata; Apoidea; Anthophila; Apidae; Apinae; Bombini; Bombus; Bombus; Bombus terrestris |
| *Frieseomelitta varia* | GCF_011392965.1 | NCBI | 99.0 | 98.3 | 0.7 | 0.4 | 0.6 | 1367 | Arthropoda; Mandibulata; Pancrustacea; Hexapoda; Insecta; Dicondylia; Pterygota; Neoptera; Endopterygota; Hymenoptera; Apocrita; Aculeata; Apoidea; Anthophila; Apidae; Apinae; Meliponini; Frieseomelitta; Frieseomelitta varia |
| *Melipona quadrifasciata* | GCA_001276565.1 | NCBI | 91.0 | 90.3 | 0.7 | 1.8 | 7.2 | 1367 | Arthropoda; Mandibulata; Pancrustacea; Hexapoda; Insecta; Dicondylia; Pterygota; Neoptera; Endopterygota; Hymenoptera; Apocrita; Aculeata; Apoidea; Anthophila; Apidae; Apinae; Meliponini; Melipona; Melipona quadrifasciata |
| *Apis florea* | GCF_000184785.3 | NCBI | 98.9 | 98.5 | 0.4 | 0.3 | 0.8 | 1367 | Arthropoda; Mandibulata; Pancrustacea; Hexapoda; Insecta; Dicondylia; Pterygota; Neoptera; Endopterygota; Hymenoptera; Apocrita; Aculeata; Apoidea; Anthophila; Apidae; Apinae; Apini; Apis; Apis florea |
| *Apis cerana* | GCF_029169275.1 | NCBI | 99.6 | 98.9 | 0.7 | 0.1 | 0.3 | 1367 | Arthropoda; Mandibulata; Pancrustacea; Hexapoda; Insecta; Dicondylia; Pterygota; Neoptera; Endopterygota; Hymenoptera; Apocrita; Aculeata; Apoidea; Anthophila; Apidae; Apinae; Apini; Apis; Apis cerana |
| *Apis mellifera* | GCF_003254395.2 | NCBI | 99.2 | 98.8 | 0.4 | 0.1 | 0.7 | 1367 | Arthropoda; Mandibulata; Pancrustacea; Hexapoda; Insecta; Dicondylia; Pterygota; Neoptera; Endopterygota; Hymenoptera; Apocrita; Aculeata; Apoidea; Anthophila; Apidae; Apinae; Apini; Apis; Apis mellifera |
| *Apis dorsata* | GCF_000469605.1 | NCBI | 98.5 | 98.1 | 0.4 | 0.7 | 0.8 | 1367 | Arthropoda; Mandibulata; Pancrustacea; Hexapoda; Insecta; Dicondylia; Pterygota; Neoptera; Endopterygota; Hymenoptera; Apocrita; Aculeata; Apoidea; Anthophila; Apidae; Apinae; Apini; Apis; Apis dorsata |
| *Apis laboriosa* | GCF_014066325.1 | NCBI | 99.4 | 99.0 | 0.4 | 0.1 | 0.5 | 1367 | Arthropoda; Mandibulata; Pancrustacea; Hexapoda; Insecta; Dicondylia; Pterygota; Neoptera; Endopterygota; Hymenoptera; Apocrita; Aculeata; Apoidea; Anthophila; Apidae; Apinae; Apini; Apis; Apis laboriosa |
| *Dinoponera quadriceps* | GCF_001313825.1 | NCBI | 99.1 | 98.3 | 0.8 | 0.4 | 0.5 | 1367 | Arthropoda; Mandibulata; Pancrustacea; Hexapoda; Insecta; Dicondylia; Pterygota; Neoptera; Endopterygota; Hymenoptera; Apocrita; Aculeata; Formicoidea; Formicidae; Ponerinae; Ponerini; Dinoponera; Dinoponera quadriceps |
| *Harpegnathos saltator* | GCF_003227715.2 | NCBI | 99.7 | 98.2 | 1.5 | 0.1 | 0.2 | 1367 | Arthropoda; Mandibulata; Pancrustacea; Hexapoda; Insecta; Dicondylia; Pterygota; Neoptera; Endopterygota; Hymenoptera; Apocrita; Aculeata; Formicoidea; Formicidae; Ponerinae; Ponerini; Harpegnathos; Harpegnathos saltator |
| *Odontomachus brunneus* | GCF_010583005.1 | NCBI | 99.5 | 97.4 | 2.1 | 0.0 | 0.5 | 1367 | Arthropoda; Mandibulata; Pancrustacea; Hexapoda; Insecta; Dicondylia; Pterygota; Neoptera; Endopterygota; Hymenoptera; Apocrita; Aculeata; Formicoidea; Formicidae; Ponerinae; Ponerini; Odontomachus; Odontomachus brunneus |
| *Eciton burchellii* | GCA_020341155.1 | NCBI | 98.7 | 97.5 | 1.2 | 0.3 | 1.0 | 1367 | Arthropoda; Mandibulata; Pancrustacea; Hexapoda; Insecta; Dicondylia; Pterygota; Neoptera; Endopterygota; Hymenoptera; Apocrita; Aculeata; Formicoidea; Formicidae; Dorylinae; Eciton; Eciton burchellii |
| *Ooceraea biroi* | GCF_003672135.1 | NCBI | 99.3 | 97.0 | 2.3 | 0.1 | 0.6 | 1367 | Arthropoda; Mandibulata; Pancrustacea; Hexapoda; Insecta; Dicondylia; Pterygota; Neoptera; Endopterygota; Hymenoptera; Apocrita; Aculeata; Formicoidea; Formicidae; Dorylinae; Ooceraea; Ooceraea biroi |
| *Linepithema humile* | GCF_000217595.1 | NCBI | 99.2 | 98.2 | 1.0 | 0.2 | 0.6 | 1367 | Arthropoda; Mandibulata; Pancrustacea; Hexapoda; Insecta; Dicondylia; Pterygota; Neoptera; Endopterygota; Hymenoptera; Apocrita; Aculeata; Formicoidea; Formicidae; Dolichoderinae; Linepithema; Linepithema humile |
| *Pseudomyrmex gracilis* | GCF_002006095.1 | NCBI | 99.2 | 98.0 | 1.2 | 0.1 | 0.7 | 1367 | Arthropoda; Mandibulata; Pancrustacea; Hexapoda; Insecta; Dicondylia; Pterygota; Neoptera; Endopterygota; Hymenoptera; Apocrita; Aculeata; Formicoidea; Formicidae; Pseudomyrmecinae; Pseudomyrmex |
| *Nylanderia fulva* | GCF_005281655.2 | NCBI | 98.4 | 94.7 | 3.7 | 0.1 | 1.5 | 1367 | Arthropoda; Mandibulata; Pancrustacea; Hexapoda; Insecta; Dicondylia; Pterygota; Neoptera; Endopterygota; Hymenoptera; Apocrita; Aculeata; Formicoidea; Formicidae; Formicinae; Lasiini; Nylanderia; Nylanderia fulva |
| *Camponotus floridanus* | GCF_003227725.1 | NCBI | 99.3 | 98.3 | 1.0 | 0.2 | 0.5 | 1367 | Arthropoda; Mandibulata; Pancrustacea; Hexapoda; Insecta; Dicondylia; Pterygota; Neoptera; Endopterygota; Hymenoptera; Apocrita; Aculeata; Formicoidea; Formicidae; Formicinae; Camponotini; Camponotus |
| *Cataglyphis hispanica* | GCF_021464435.1 | NCBI | 99.6 | 98.4 | 1.2 | 0.1 | 0.3 | 1367 | Arthropoda; Mandibulata; Pancrustacea; Hexapoda; Insecta; Dicondylia; Pterygota; Neoptera; Endopterygota; Hymenoptera; Apocrita; Aculeata; Formicoidea; Formicidae; Formicinae; Formicini; Cataglyphis |
| *Formica exsecta* | GCF_003651465.1 | NCBI | 99.0 | 95.0 | 4.0 | 0.8 | 0.2 | 1367 | Arthropoda; Mandibulata; Pancrustacea; Hexapoda; Insecta; Dicondylia; Pterygota; Neoptera; Endopterygota; Hymenoptera; Apocrita; Aculeata; Formicoidea; Formicidae; Formicinae; Formicini; Formica; Formica exsecta |
| *Pogonomyrmex barbatus* | GCF_000187915.1 | NCBI | 97.8 | 97.3 | 0.5 | 1.7 | 0.5 | 1367 | Arthropoda; Mandibulata; Pancrustacea; Hexapoda; Insecta; Dicondylia; Pterygota; Neoptera; Endopterygota; Hymenoptera; Apocrita; Aculeata; Formicoidea; Formicidae; Myrmicinae; Pogonomyrmecini; Pogonomyrmex; Pogonomyrmex barbatus |
| *Vollenhovia emeryi* | GCF_000949405.1 | NCBI | 99.6 | 98.7 | 0.9 | 0.2 | 0.2 | 1367 | Arthropoda; Mandibulata; Pancrustacea; Hexapoda; Insecta; Dicondylia; Pterygota; Neoptera; Endopterygota; Hymenoptera; Apocrita; Aculeata; Formicoidea; Formicidae; Myrmicinae; Stenammini; Vollenhovia; Vollenhovia emeryi |
| *Temnothorax curvispinosus* | GCF_003070985.1 | NCBI | 95.1 | 88.0 | 7.1 | 1.8 | 3.1 | 1367 | Arthropoda; Mandibulata; Pancrustacea; Hexapoda; Insecta; Dicondylia; Pterygota; Neoptera; Endopterygota; Hymenoptera; Apocrita; Aculeata; Formicoidea; Formicidae; Myrmicinae; Crematogastrini; Temnothorax; Temnothorax curvispinosus |
| *Temnothorax longispinosus* | GCA_004794745.1 | NCBI | 90.6 | 89.8 | 0.8 | 2.0 | 7.4 | 1367 | Arthropoda; Mandibulata; Pancrustacea; Hexapoda; Insecta; Dicondylia; Pterygota; Neoptera; Endopterygota; Hymenoptera; Apocrita; Aculeata; Formicoidea; Formicidae; Myrmicinae; Crematogastrini; Temnothorax; Temnothorax longispinosus |
| *Monomorium pharaonis* | GCF_013373865.1 | NCBI | 99.6 | 95.6 | 4.0 | 0.2 | 0.2 | 1367 | Arthropoda; Mandibulata; Pancrustacea; Hexapoda; Insecta; Dicondylia; Pterygota; Neoptera; Endopterygota; Hymenoptera; Apocrita; Aculeata; Formicoidea; Formicidae; Myrmicinae; Solenopsidini; Monomorium; Monomorium pharaonis |
| *Solenopsis invicta* | GCF_016802725.1 | NCBI | 99.5 | 97.8 | 1.7 | 0.1 | 0.4 | 1367 | Arthropoda; Mandibulata; Pancrustacea; Hexapoda; Insecta; Dicondylia; Pterygota; Neoptera; Endopterygota; Hymenoptera; Apocrita; Aculeata; Formicoidea; Formicidae; Myrmicinae; Solenopsidini; Solenopsis; Solenopsis invicta |
| *Wasmannia auropunctata* | GCF_000956235.1 | NCBI | 99.1 | 98.0 | 1.1 | 0.6 | 0.3 | 1367 | Arthropoda; Mandibulata; Pancrustacea; Hexapoda; Insecta; Dicondylia; Pterygota; Neoptera; Endopterygota; Hymenoptera; Apocrita; Aculeata; Formicoidea; Formicidae; Myrmicinae; Blepharidattini; Wasmannia; Wasmannia auropunctata |
| *Cyphomyrmex costatus* | GCF_001594065.2 | NCBI | 99.1 | 97.7 | 1.4 | 0.3 | 0.6 | 1367 | Arthropoda; Mandibulata; Pancrustacea; Hexapoda; Insecta; Dicondylia; Pterygota; Neoptera; Endopterygota; Hymenoptera; Apocrita; Aculeata; Formicoidea; Formicidae; Myrmicinae; Attini; Cyphomyrmex; Cyphomyrmex costatus |
| *Trachymyrmex septentrionalis* | GCF_001594115.1 | NCBI | 99.4 | 99.0 | 0.4 | 0.1 | 0.5 | 1367 | Arthropoda; Mandibulata; Pancrustacea; Hexapoda; Insecta; Dicondylia; Pterygota; Neoptera; Endopterygota; Hymenoptera; Apocrita; Aculeata; Formicoidea; Formicidae; Myrmicinae; Attini; Trachymyrmex; Trachymyrmex septentrionalis |
| *Acromyrmex echinatior* | GCF_000204515.1 | NCBI | 99.4 | 98.5 | 0.9 | 0.3 | 0.3 | 1367 | Arthropoda; Mandibulata; Pancrustacea; Hexapoda; Insecta; Dicondylia; Pterygota; Neoptera; Endopterygota; Hymenoptera; Apocrita; Aculeata; Formicoidea; Formicidae; Myrmicinae; Attini; Acromyrmex; Acromyrmex echinatior |
| *Atta cephalotes* | GCF_000143395.1 | NCBI | 98.1 | 97.3 | 0.8 | 0.7 | 1.2 | 1367 | Arthropoda; Mandibulata; Pancrustacea; Hexapoda; Insecta; Dicondylia; Pterygota; Neoptera; Endopterygota; Hymenoptera; Apocrita; Aculeata; Formicoidea; Formicidae; Myrmicinae; Attini; Atta |
| *Atta colombica* | GCF_001594045.1 | NCBI | 99.5 | 98.7 | 0.8 | 0.1 | 0.4 | 1367 | Arthropoda; Mandibulata; Pancrustacea; Hexapoda; Insecta; Dicondylia; Pterygota; Neoptera; Endopterygota; Hymenoptera; Apocrita; Aculeata; Formicoidea; Formicidae; Myrmicinae; Attini; Atta; Atta colombica |
| *Chrysoperla carnea* | GCF_905475395.1 | NCBI | 98.2 | 97.1 | 1.1 | 0.1 | 1.7 | 1367 | Arthropoda; Mandibulata; Pancrustacea; Hexapoda; Insecta; Dicondylia; Pterygota; Neoptera; Endopterygota; Neuropterida; Neuroptera; Hemerobiiformia; Chrysopidae; Chrysopinae; Chrysoperla; Chrysoperla carnea |
| *Agrilus planipennis* | GCF_000699045.2 | NCBI | 97.5 | 90.8 | 6.7 | 1.2 | 1.3 | 1367 | Arthropoda; Mandibulata; Pancrustacea; Hexapoda; Insecta; Dicondylia; Pterygota; Neoptera; Endopterygota; Coleoptera; Polyphaga; Elateriformia; Buprestoidea; Buprestidae; Agrilinae; Agrilus; Agrilus planipennis |
| *Ignelater luminosus* | GCA_011009095.1 | NCBI | 94.1 | 91.3 | 2.8 | 2.5 | 3.4 | 1367 | Arthropoda; Mandibulata; Pancrustacea; Hexapoda; Insecta; Dicondylia; Pterygota; Neoptera; Endopterygota; Coleoptera; Polyphaga; Elateriformia; Elateroidea; Elateridae; Agrypninae; Pyrophorini; Ignelater; Ignelater luminosus |
| *Abscondita terminalis* | GCA_013368085.1 | NCBI | 94.8 | 91.4 | 3.4 | 1.4 | 3.8 | 1367 | Arthropoda; Mandibulata; Pancrustacea; Hexapoda; Insecta; Dicondylia; Pterygota; Neoptera; Endopterygota; Coleoptera; Polyphaga; Elateriformia; Elateroidea; Lampyridae; Luciolinae; Abscondita; Abscondita terminalis |
| *Lamprigera yunnana* | GCA_013368075.1 | NCBI | 94.5 | 92.7 | 1.8 | 1.6 | 3.9 | 1367 | Arthropoda; Mandibulata; Pancrustacea; Hexapoda; Insecta; Dicondylia; Pterygota; Neoptera; Endopterygota; Coleoptera; Polyphaga; Elateriformia; Elateroidea; Lampyridae; Lampyrinae; Lamprigera; Lamprigera yunnana |
| *Photinus pyralis* | GCF_008802855.1 | NCBI | 99.0 | 85.2 | 13.8 | 0.1 | 0.9 | 1367 | Arthropoda; Mandibulata; Pancrustacea; Hexapoda; Insecta; Dicondylia; Pterygota; Neoptera; Endopterygota; Coleoptera; Polyphaga; Elateriformia; Elateroidea; Lampyridae; Lampyrinae; Photinus; Photinus pyralis |
| *Onthophagus taurus* | GCF_000648695.1 | NCBI | 99.7 | 96.5 | 3.2 | 0.1 | 0.2 | 1367 | Arthropoda; Mandibulata; Pancrustacea; Hexapoda; Insecta; Dicondylia; Pterygota; Neoptera; Endopterygota; Coleoptera; Polyphaga; Scarabaeiformia; Scarabaeoidea; Scarabaeidae; Scarabaeinae; Onthophagini; Onthophagus; Onthophagus taurus |
| *Dalotia coriaria* | GCF_025399875.1 | NCBI | 97.9 | 87.6 | 10.3 | 0.2 | 1.9 | 1367 | Arthropoda; Mandibulata; Pancrustacea; Hexapoda; Insecta; Dicondylia; Pterygota; Neoptera; Endopterygota; Coleoptera; Polyphaga; Staphyliniformia; Staphylinoidea; Staphylinidae; Tachyporinae group; Aleocharinae; Athetini; Dalotia; Dalotia coriaria |
| *Nicrophorus vespilloides* | GCF_001412225.1 | NCBI | 99.8 | 98.2 | 1.6 | 0.1 | 0.1 | 1367 | Arthropoda; Mandibulata; Pancrustacea; Hexapoda; Insecta; Dicondylia; Pterygota; Neoptera; Endopterygota; Coleoptera; Polyphaga; Staphyliniformia; Staphylinoidea; Silphidae; Nicrophorinae; Nicrophorus; Nicrophorus vespilloides |
| *Tenebrio molitor* | GCF_036711695.1 | NCBI | 96.5 | 95.4 | 1.1 | 0.9 | 2.6 | 1367 | Arthropoda; Mandibulata; Pancrustacea; Hexapoda; Insecta; Dicondylia; Pterygota; Neoptera; Endopterygota; Coleoptera; Polyphaga; Cucujiformia; Tenebrionoidea; Tenebrionidae; Tenebrioninae; Tenebrio; Tenebrio molitor |
| *Zophobas morio* | GCF_031307605.1 | NCBI | 99.7 | 96.3 | 3.4 | 0.1 | 0.2 | 1367 | Arthropoda; Mandibulata; Pancrustacea; Hexapoda; Insecta; Dicondylia; Pterygota; Neoptera; Endopterygota; Coleoptera; Polyphaga; Cucujiformia; Tenebrionoidea; Tenebrionidae; Tenebrioninae; Zophobas; Zophobas morio |
| *Tribolium castaneum* | GCF_015345945.1 | NCBI | 99.2 | 98.4 | 0.8 | 0.1 | 0.7 | 1367 | Arthropoda; Mandibulata; Pancrustacea; Hexapoda; Insecta; Dicondylia; Pterygota; Neoptera; Endopterygota; Coleoptera; Polyphaga; Cucujiformia; Tenebrionoidea; Tenebrionidae; Tenebrionidae incertae sedis; Tribolium |
| *Tribolium madens* | GCF_907165205.1 | NCBI | 99.4 | 98.6 | 0.8 | 0.1 | 0.5 | 1367 | Arthropoda; Mandibulata; Pancrustacea; Hexapoda; Insecta; Dicondylia; Pterygota; Neoptera; Endopterygota; Coleoptera; Polyphaga; Cucujiformia; Tenebrionoidea; Tenebrionidae; Tenebrionidae incertae sedis; Tribolium; Tribolium madens |
| *Coccinella septempunctata* | GCF_914767665.1 | NCBI | 99.0 | 97.1 | 1.9 | 0.1 | 0.9 | 1367 | Arthropoda; Mandibulata; Pancrustacea; Hexapoda; Insecta; Dicondylia; Pterygota; Neoptera; Endopterygota; Coleoptera; Polyphaga; Cucujiformia; Coccinelloidea; Coccinellidae; Coccinellinae; Coccinellini; Coccinella; Coccinella septempunctata |
| *Harmonia axyridis* | GCF_024364675.1 | NCBI | 97.9 | 95.2 | 2.7 | 0.1 | 2.0 | 1367 | Arthropoda; Mandibulata; Pancrustacea; Hexapoda; Insecta; Dicondylia; Pterygota; Neoptera; Endopterygota; Coleoptera; Polyphaga; Cucujiformia; Coccinelloidea; Coccinellidae; Coccinellinae; Coccinellini; Harmonia; Harmonia axyridis |
| *Aethina tumida* | GCA_921294245.1 | NCBI | 99.5 | 96.9 | 2.6 | 0.0 | 0.5 | 1367 | Arthropoda; Mandibulata; Pancrustacea; Hexapoda; Insecta; Dicondylia; Pterygota; Neoptera; Endopterygota; Coleoptera; Polyphaga; Cucujiformia; Cucujoidea; Nitidulidae; Nitidulinae; Aethina; Aethina tumida |
| *Brassicogethes aeneus* | GCF_029955315.1 | NCBI | 95.6 | 93.8 | 1.8 | 0.5 | 3.9 | 1367 | Arthropoda; Mandibulata; Pancrustacea; Hexapoda; Insecta; Dicondylia; Pterygota; Neoptera; Endopterygota; Coleoptera; Polyphaga; Cucujiformia; Cucujoidea; Nitidulidae; Meligethinae; Brassicogethes; Brassicogethes aeneus |
| *Cylas formicarius* | GCF_022605725.1 | NCBI | 98.7 | 97.4 | 1.3 | 0.1 | 1.2 | 1367 | Arthropoda; Mandibulata; Pancrustacea; Hexapoda; Insecta; Dicondylia; Pterygota; Neoptera; Endopterygota; Coleoptera; Polyphaga; Cucujiformia; Curculionoidea; Brentidae; Cyladinae; Cylas; Cylas formicarius |
| *Anthonomus grandis grandis* | GCF_020466585.1 | NCBI | 99.3 | 96.6 | 2.7 | 0.0 | 0.7 | 1367 | Arthropoda; Mandibulata; Pancrustacea; Hexapoda; Insecta; Dicondylia; Pterygota; Neoptera; Endopterygota; Coleoptera; Polyphaga; Cucujiformia; Curculionoidea; Curculionidae; Curculioninae; Anthonomini; Anthonomus; Anthonomus grandis; Anthonomus grandis grandis |
| *Dendroctonus ponderosae* | GCA_014462685.1 | NCBI | 99.2 | 93.1 | 6.1 | 0.1 | 0.7 | 1367 | Arthropoda; Mandibulata; Pancrustacea; Hexapoda; Insecta; Dicondylia; Pterygota; Neoptera; Endopterygota; Coleoptera; Polyphaga; Cucujiformia; Curculionoidea; Curculionidae; Scolytinae; Dendroctonus; Dendroctonus ponderosae |
| *Rhynchophorus ferrugineus* | GCF_002938485.1 | NCBI | 96.6 | 93.7 | 2.9 | 1.7 | 1.7 | 1367 | Arthropoda; Mandibulata; Pancrustacea; Hexapoda; Insecta; Dicondylia; Pterygota; Neoptera; Endopterygota; Coleoptera; Polyphaga; Cucujiformia; Curculionoidea; Curculionidae; Dryophthorinae; Rhynchophorus; Rhynchophorus ferrugineus |
| *Sitophilus oryzae* | GCF_000390285.2 | NCBI | 98.7 | 95.2 | 3.5 | 0.1 | 1.2 | 1367 | Arthropoda; Mandibulata; Pancrustacea; Hexapoda; Insecta; Dicondylia; Pterygota; Neoptera; Endopterygota; Coleoptera; Polyphaga; Cucujiformia; Curculionoidea; Curculionidae; Dryophthorinae; Sitophilus; Sitophilus oryzae |
| *Anoplophora glabripennis* | GCA_018342105.1 | NCBI | 99.3 | 97.0 | 2.3 | 0.4 | 0.3 | 1367 | Arthropoda; Mandibulata; Pancrustacea; Hexapoda; Insecta; Dicondylia; Pterygota; Neoptera; Endopterygota; Coleoptera; Polyphaga; Cucujiformia; Chrysomeloidea; Cerambycidae; Lamiinae; Lamiini; Anoplophora; Anoplophora glabripennis |
| *Gonioctena quinquepunctata* | GCF_000500325.1 | NCBI | 91.3 | 89.3 | 2.0 | 4.3 | 4.4 | 1367 | Arthropoda; Mandibulata; Pancrustacea; Hexapoda; Insecta; Dicondylia; Pterygota; Neoptera; Endopterygota; Coleoptera; Polyphaga; Cucujiformia; Chrysomeloidea; Chrysomelidae; Chrysomelinae; Chrysomelini; Gonioctena; Gonioctena quinquepunctata |
| *Leptinotarsa decemlineata* | GCF_917563875.1 | NCBI | 95.0 | 93.6 | 1.4 | 2.6 | 2.4 | 1367 | Arthropoda; Mandibulata; Pancrustacea; Hexapoda; Insecta; Dicondylia; Pterygota; Neoptera; Endopterygota; Coleoptera; Polyphaga; Cucujiformia; Chrysomeloidea; Chrysomelidae; Chrysomelinae; Doryphorini; Leptinotarsa; Leptinotarsa decemlineata |
| *Diabrotica virgifera virgifera* | GCF_026250575.1 | NCBI | 98.1 | 96.6 | 1.5 | 0.2 | 1.7 | 1367 | Arthropoda; Mandibulata; Pancrustacea; Hexapoda; Insecta; Dicondylia; Pterygota; Neoptera; Endopterygota; Coleoptera; Polyphaga; Cucujiformia; Chrysomeloidea; Chrysomelidae; Galerucinae; Luperini; Diabroticina; Diabroticites; Diabrotica; Diabrotica virgifera; Diabrotica virgifera virgifera |
| *Diorhabda carinulata* | GCF_026230105.1 | NCBI | 99.5 | 98.4 | 1.1 | 0.0 | 0.5 | 1367 | Arthropoda; Mandibulata; Pancrustacea; Hexapoda; Insecta; Dicondylia; Pterygota; Neoptera; Endopterygota; Coleoptera; Polyphaga; Cucujiformia; Chrysomeloidea; Chrysomelidae; Galerucinae; Galerucini; Atysites; Diorhabda; Diorhabda carinulata |
| *Diorhabda sublineata* | GCF_932276165.1 | NCBI | 99.6 | 98.3 | 1.3 | 0.0 | 0.4 | 1367 | Arthropoda; Mandibulata; Pancrustacea; Hexapoda; Insecta; Dicondylia; Pterygota; Neoptera; Endopterygota; Coleoptera; Polyphaga; Cucujiformia; Chrysomeloidea; Chrysomelidae; Galerucinae; Galerucini; Atysites; Diorhabda; Diorhabda sublineata |
| *Plutella xylostella* | GCF_023078275.1 | NCBI | 99.6 | 98.9 | 0.7 | 0.1 | 0.3 | 1367 | Arthropoda; Mandibulata; Pancrustacea; Hexapoda; Insecta; Dicondylia; Pterygota; Neoptera; Endopterygota; Amphiesmenoptera; Lepidoptera; Glossata; Neolepidoptera; Heteroneura; Ditrysia; Yponomeutoidea; Plutellidae; Plutella; Plutella xylostella |
| *Leguminivora glycinivorella* | GCF_033807575.1 | NCBI | 93.8 | 92.0 | 1.8 | 0.2 | 6.0 | 1367 | Arthropoda; Mandibulata; Pancrustacea; Hexapoda; Insecta; Dicondylia; Pterygota; Neoptera; Endopterygota; Amphiesmenoptera; Lepidoptera; Glossata; Neolepidoptera; Heteroneura; Ditrysia; Apoditrysia; Tortricoidea; Tortricidae; Olethreutinae; Grapholitini; Leguminivora; Leguminivora glycinivorella |
| *Cydia pomonella* | GCF_910591565.1 | NCBI | 99.2 | 94.7 | 4.5 | 0.2 | 0.6 | 1367 | Arthropoda; Mandibulata; Pancrustacea; Hexapoda; Insecta; Dicondylia; Pterygota; Neoptera; Endopterygota; Amphiesmenoptera; Lepidoptera; Glossata; Neolepidoptera; Heteroneura; Ditrysia; Apoditrysia; Tortricoidea; Tortricidae; Olethreutinae; Grapholitini; Cydia; Cydia pomonella |
| *Cydia splendana* | GCF_003589595.1 | NCBI | 99.7 | 98.7 | 1.0 | 0.1 | 0.2 | 1367 | Arthropoda; Mandibulata; Pancrustacea; Hexapoda; Insecta; Dicondylia; Pterygota; Neoptera; Endopterygota; Amphiesmenoptera; Lepidoptera; Glossata; Neolepidoptera; Heteroneura; Ditrysia; Apoditrysia; Tortricoidea; Tortricidae; Olethreutinae; Grapholitini; Cydia; Cydia splendana |
| *Hyposmocoma kahamanoa* | GCF_024362695.1 | NCBI | 94.7 | 93.7 | 1.0 | 1.8 | 3.5 | 1367 | Arthropoda; Mandibulata; Pancrustacea; Hexapoda; Insecta; Dicondylia; Pterygota; Neoptera; Endopterygota; Amphiesmenoptera; Lepidoptera; Glossata; Neolepidoptera; Heteroneura; Ditrysia; Gelechioidea; Cosmopterigidae; Cosmopteriginae; Hyposmocoma; Hyposmocoma kahamanoa |
| *Pectinophora gossypiella* | GCA_902850365.2 | NCBI | 99.4 | 97.5 | 1.9 | 0.2 | 0.4 | 1367 | Arthropoda; Mandibulata; Pancrustacea; Hexapoda; Insecta; Dicondylia; Pterygota; Neoptera; Endopterygota; Amphiesmenoptera; Lepidoptera; Glossata; Neolepidoptera; Heteroneura; Ditrysia; Gelechioidea; Gelechiidae; Apatetrinae; Pectinophora; Pectinophora gossypiella |
| *Chilo suppressalis* | GCA_004000445.1 | NCBI | 92.1 | 89.5 | 2.6 | 2.3 | 5.6 | 1367 | Arthropoda; Mandibulata; Pancrustacea; Hexapoda; Insecta; Dicondylia; Pterygota; Neoptera; Endopterygota; Amphiesmenoptera; Lepidoptera; Glossata; Neolepidoptera; Heteroneura; Ditrysia; Obtectomera; Pyraloidea; Crambidae; Crambinae; Chilo; Chilo suppressalis |
| *Ostrinia furnacalis* | GCF_004193835.3 | NCBI | 98.0 | 96.6 | 1.4 | 0.9 | 1.1 | 1367 | Arthropoda; Mandibulata; Pancrustacea; Hexapoda; Insecta; Dicondylia; Pterygota; Neoptera; Endopterygota; Amphiesmenoptera; Lepidoptera; Glossata; Neolepidoptera; Heteroneura; Ditrysia; Obtectomera; Pyraloidea; Crambidae; Pyraustinae; Ostrinia; Ostrinia furnacalis |
| *Ostrinia nubilalis* | GCF_963855985.1 | NCBI | 98.0 | 97.3 | 0.7 | 0.5 | 1.5 | 1367 | Arthropoda; Mandibulata; Pancrustacea; Hexapoda; Insecta; Dicondylia; Pterygota; Neoptera; Endopterygota; Amphiesmenoptera; Lepidoptera; Glossata; Neolepidoptera; Heteroneura; Ditrysia; Obtectomera; Pyraloidea; Crambidae; Pyraustinae; Ostrinia; Ostrinia nubilalis |
| *Achroia grisella* | GCF_030625045.1 | NCBI | 99.9 | 97.4 | 2.5 | 0.0 | 0.1 | 1367 | Arthropoda; Mandibulata; Pancrustacea; Hexapoda; Insecta; Dicondylia; Pterygota; Neoptera; Endopterygota; Amphiesmenoptera; Lepidoptera; Glossata; Neolepidoptera; Heteroneura; Ditrysia; Obtectomera; Pyraloidea; Pyralidae; Galleriinae; Achroia; Achroia grisella |
| *Galleria mellonella* | GCF_026898425.1 | NCBI | 99.8 | 98.9 | 0.9 | 0.0 | 0.2 | 1367 | Arthropoda; Mandibulata; Pancrustacea; Hexapoda; Insecta; Dicondylia; Pterygota; Neoptera; Endopterygota; Amphiesmenoptera; Lepidoptera; Glossata; Neolepidoptera; Heteroneura; Ditrysia; Obtectomera; Pyraloidea; Pyralidae; Galleriinae; Galleria; Galleria mellonella |
| *Amyelois transitella* | GCF_032362555.1 | NCBI | 99.7 | 98.9 | 0.8 | 0.1 | 0.2 | 1367 | Arthropoda; Mandibulata; Pancrustacea; Hexapoda; Insecta; Dicondylia; Pterygota; Neoptera; Endopterygota; Amphiesmenoptera; Lepidoptera; Glossata; Neolepidoptera; Heteroneura; Ditrysia; Obtectomera; Pyraloidea; Pyralidae; Phycitinae; Amyelois; Amyelois transitella |
| *Plodia interpunctella* | GCF_027563975.2 | NCBI | 99.8 | 99.0 | 0.8 | 0.0 | 0.2 | 1367 | Arthropoda; Mandibulata; Pancrustacea; Hexapoda; Insecta; Dicondylia; Pterygota; Neoptera; Endopterygota; Amphiesmenoptera; Lepidoptera; Glossata; Neolepidoptera; Heteroneura; Ditrysia; Obtectomera; Pyraloidea; Pyralidae; Phycitinae; Plodia; Plodia interpunctella |
| *Manduca sexta* | GCF_014839805.1 | NCBI | 99.2 | 89.5 | 9.7 | 0.1 | 0.7 | 1367 | Arthropoda; Mandibulata; Pancrustacea; Hexapoda; Insecta; Dicondylia; Pterygota; Neoptera; Endopterygota; Amphiesmenoptera; Lepidoptera; Glossata; Neolepidoptera; Heteroneura; Ditrysia; Obtectomera; Bombycoidea; Sphingidae; Sphinginae; Sphingini; Manduca; Manduca sexta |
| *Bombyx mandarina* | GCF_003987935.1 | NCBI | 95.3 | 94.6 | 0.7 | 0.4 | 4.3 | 1367 | Arthropoda; Mandibulata; Pancrustacea; Hexapoda; Insecta; Dicondylia; Pterygota; Neoptera; Endopterygota; Amphiesmenoptera; Lepidoptera; Glossata; Neolepidoptera; Heteroneura; Ditrysia; Obtectomera; Bombycoidea; Bombycidae; Bombycinae; Bombyx; Bombyx mori |
| *Bombyx mori* | GCF_030269925.1 | NCBI | 99.5 | 98.8 | 0.7 | 0.1 | 0.4 | 1367 | Arthropoda; Mandibulata; Pancrustacea; Hexapoda; Insecta; Dicondylia; Pterygota; Neoptera; Endopterygota; Amphiesmenoptera; Lepidoptera; Glossata; Neolepidoptera; Heteroneura; Ditrysia; Obtectomera; Bombycoidea; Bombycidae; Bombycinae; Bombyx; Bombyx mori |
| *Arctia plantaginis* | GCA_902825455.1 | NCBI | 95.9 | 94.7 | 1.2 | 1.1 | 3.0 | 1367 | Arthropoda; Mandibulata; Pancrustacea; Hexapoda; Insecta; Dicondylia; Pterygota; Neoptera; Endopterygota; Amphiesmenoptera; Lepidoptera; Glossata; Neolepidoptera; Heteroneura; Ditrysia; Obtectomera; Noctuoidea; Erebidae; Arctiinae; Arctiini; Arctia; Arctia plantaginis |
| *Trichoplusia ni* | GCF_003590095.1 | NCBI | 98.8 | 91.3 | 7.5 | 0.1 | 1.1 | 1367 | Arthropoda; Mandibulata; Pancrustacea; Hexapoda; Insecta; Dicondylia; Pterygota; Neoptera; Endopterygota; Amphiesmenoptera; Lepidoptera; Glossata; Neolepidoptera; Heteroneura; Ditrysia; Obtectomera; Noctuoidea; Noctuidae; Plusiinae; Trichoplusia; Trichoplusia ni |
| *Helicoverpa armigera* | GCF_030705265.1 | NCBI | 99.3 | 94.5 | 4.8 | 0.1 | 0.6 | 1367 | Arthropoda; Mandibulata; Pancrustacea; Hexapoda; Insecta; Dicondylia; Pterygota; Neoptera; Endopterygota; Amphiesmenoptera; Lepidoptera; Glossata; Neolepidoptera; Heteroneura; Ditrysia; Obtectomera; Noctuoidea; Noctuidae; Heliothinae; Helicoverpa; Helicoverpa armigera |
| *Helicoverpa zea* | GCF_022581195.2 | NCBI | 99.9 | 99.3 | 0.6 | 0.0 | 0.1 | 1367 | Arthropoda; Mandibulata; Pancrustacea; Hexapoda; Insecta; Dicondylia; Pterygota; Neoptera; Endopterygota; Amphiesmenoptera; Lepidoptera; Glossata; Neolepidoptera; Heteroneura; Ditrysia; Obtectomera; Noctuoidea; Noctuidae; Heliothinae; Helicoverpa; Helicoverpa zea |
| *Spodoptera frugiperda* | GCF_023101765.2 | NCBI | 99.6 | 98.2 | 1.4 | 0.0 | 0.4 | 1367 | Arthropoda; Mandibulata; Pancrustacea; Hexapoda; Insecta; Dicondylia; Pterygota; Neoptera; Endopterygota; Amphiesmenoptera; Lepidoptera; Glossata; Neolepidoptera; Heteroneura; Ditrysia; Obtectomera; Noctuoidea; Noctuidae; Amphipyrinae; Spodoptera; Spodoptera frugiperda |
| *Spodoptera litura* | GCF_002706865.2 | NCBI | 99.4 | 98.0 | 1.4 | 0.3 | 0.3 | 1367 | Arthropoda; Mandibulata; Pancrustacea; Hexapoda; Insecta; Dicondylia; Pterygota; Neoptera; Endopterygota; Amphiesmenoptera; Lepidoptera; Glossata; Neolepidoptera; Heteroneura; Ditrysia; Obtectomera; Noctuoidea; Noctuidae; Amphipyrinae; Spodoptera; Spodoptera litura |
| *Parnassius apollo* | GCA_907164705.1 | NCBI | 96.2 | 95.0 | 1.2 | 1.4 | 2.4 | 1367 | Arthropoda; Mandibulata; Pancrustacea; Hexapoda; Insecta; Dicondylia; Pterygota; Neoptera; Endopterygota; Amphiesmenoptera; Lepidoptera; Glossata; Neolepidoptera; Heteroneura; Ditrysia; Obtectomera; Papilionoidea; Papilionidae; Parnassiinae; Parnassini; Parnassius; Parnassius; Parnassius apollo |
| *Papilio polytes* | GCF_000836215.1 | NCBI | 94.6 | 94.2 | 0.4 | 2.4 | 3.0 | 1367 | Arthropoda; Mandibulata; Pancrustacea; Hexapoda; Insecta; Dicondylia; Pterygota; Neoptera; Endopterygota; Amphiesmenoptera; Lepidoptera; Glossata; Neolepidoptera; Heteroneura; Ditrysia; Obtectomera; Papilionoidea; Papilionidae; Papilioninae; Papilionini; Papilio; Papilio polytes |
| *Papilio machaon* | GCF_912999745.1 | NCBI | 99.7 | 98.5 | 1.2 | 0.1 | 0.2 | 1367 | Arthropoda; Mandibulata; Pancrustacea; Hexapoda; Insecta; Dicondylia; Pterygota; Neoptera; Endopterygota; Amphiesmenoptera; Lepidoptera; Glossata; Neolepidoptera; Heteroneura; Ditrysia; Obtectomera; Papilionoidea; Papilionidae; Papilioninae; Papilionini; Papilio; Papilio machaon |
| *Papilio xuthus* | GCF_000836235.2 | NCBI | 99.4 | 99.1 | 0.3 | 0.1 | 0.5 | 1367 | Arthropoda; Mandibulata; Pancrustacea; Hexapoda; Insecta; Dicondylia; Pterygota; Neoptera; Endopterygota; Amphiesmenoptera; Lepidoptera; Glossata; Neolepidoptera; Heteroneura; Ditrysia; Obtectomera; Papilionoidea; Papilionidae; Papilioninae; Papilionini; Papilio; Papilio xuthus |
| *Leptidea sinapis* | GCF_905404315.1 | NCBI | 99.1 | 98.0 | 1.1 | 0.2 | 0.7 | 1367 | Arthropoda; Mandibulata; Pancrustacea; Hexapoda; Insecta; Dicondylia; Pterygota; Neoptera; Endopterygota; Amphiesmenoptera; Lepidoptera; Glossata; Neolepidoptera; Heteroneura; Ditrysia; Obtectomera; Papilionoidea; Pieridae; Dismorphiinae; Leptidea; Leptidea sinapis |
| *Colias croceus* | GCF_905220415.1 | NCBI | 99.7 | 99.0 | 0.7 | 0.0 | 0.3 | 1367 | Arthropoda; Mandibulata; Pancrustacea; Hexapoda; Insecta; Dicondylia; Pterygota; Neoptera; Endopterygota; Amphiesmenoptera; Lepidoptera; Glossata; Neolepidoptera; Heteroneura; Ditrysia; Obtectomera; Papilionoidea; Pieridae; Coliadinae; Colias; Colias croceus |
| *Zerene cesonia* | GCF_012273895.1 | NCBI | 97.1 | 93.7 | 3.4 | 0.8 | 2.1 | 1367 | Arthropoda; Mandibulata; Pancrustacea; Hexapoda; Insecta; Dicondylia; Pterygota; Neoptera; Endopterygota; Amphiesmenoptera; Lepidoptera; Glossata; Neolepidoptera; Heteroneura; Ditrysia; Obtectomera; Papilionoidea; Pieridae; Coliadinae; Zerene; Zerene cesonia |
| *Pieris rapae* | GCF_905147795.1 | NCBI | 99.2 | 98.5 | 0.7 | 0.1 | 0.7 | 1367 | Arthropoda; Mandibulata; Pancrustacea; Hexapoda; Insecta; Dicondylia; Pterygota; Neoptera; Endopterygota; Amphiesmenoptera; Lepidoptera; Glossata; Neolepidoptera; Heteroneura; Ditrysia; Obtectomera; Papilionoidea; Pieridae; Pierinae; Pierini; Pieris; Pieris rapae |
| *Pieris brassicae* | GCF_905147105.1 | NCBI | 99.5 | 98.8 | 0.7 | 0.1 | 0.4 | 1367 | Arthropoda; Mandibulata; Pancrustacea; Hexapoda; Insecta; Dicondylia; Pterygota; Neoptera; Endopterygota; Amphiesmenoptera; Lepidoptera; Glossata; Neolepidoptera; Heteroneura; Ditrysia; Obtectomera; Papilionoidea; Pieridae; Pierinae; Pierini; Pieris; Pieris brassicae |
| *Pieris napi* | GCF_905475465.1 | NCBI | 99.4 | 98.2 | 1.2 | 0.1 | 0.5 | 1367 | Arthropoda; Mandibulata; Pancrustacea; Hexapoda; Insecta; Dicondylia; Pterygota; Neoptera; Endopterygota; Amphiesmenoptera; Lepidoptera; Glossata; Neolepidoptera; Heteroneura; Ditrysia; Obtectomera; Papilionoidea; Pieridae; Pierinae; Pierini; Pieris; Pieris napi |
| *Aricia agestis* | GCF_905147365.1 | NCBI | 99.6 | 98.8 | 0.8 | 0.0 | 0.4 | 1367 | Arthropoda; Mandibulata; Pancrustacea; Hexapoda; Insecta; Dicondylia; Pterygota; Neoptera; Endopterygota; Amphiesmenoptera; Lepidoptera; Glossata; Neolepidoptera; Heteroneura; Ditrysia; Obtectomera; Papilionoidea; Lycaenidae; Polyommatinae; Aricia; Aricia agestis |
| *Danaus plexippus* | GCF_018135715.1 | NCBI | 99.7 | 96.9 | 2.8 | 0.1 | 0.2 | 1367 | Arthropoda; Mandibulata; Pancrustacea; Hexapoda; Insecta; Dicondylia; Pterygota; Neoptera; Endopterygota; Amphiesmenoptera; Lepidoptera; Glossata; Neolepidoptera; Heteroneura; Ditrysia; Obtectomera; Papilionoidea; Nymphalidae; Danainae; Danaini; Danaina; Danaus; Danaus; Danaus plexippus |
| *Bicyclus anynana* | GCF_947172395.1 | NCBI | 99.7 | 98.5 | 1.2 | 0.0 | 0.3 | 1367 | Arthropoda; Mandibulata; Pancrustacea; Hexapoda; Insecta; Dicondylia; Pterygota; Neoptera; Endopterygota; Amphiesmenoptera; Lepidoptera; Glossata; Neolepidoptera; Heteroneura; Ditrysia; Obtectomera; Papilionoidea; Nymphalidae; Satyrinae; Satyrini; Mycalesina; Bicyclus; Bicyclus anynana |
| *Pararge aegeria* | GCF_905163445.1 | NCBI | 99.6 | 98.9 | 0.7 | 0.0 | 0.4 | 1367 | Arthropoda; Mandibulata; Pancrustacea; Hexapoda; Insecta; Dicondylia; Pterygota; Neoptera; Endopterygota; Amphiesmenoptera; Lepidoptera; Glossata; Neolepidoptera; Heteroneura; Ditrysia; Obtectomera; Papilionoidea; Nymphalidae; Satyrinae; Satyrini; Parargina; Pararge; Pararge aegeria |
| *Maniola hyperantus* | GCF_902806685.1 | NCBI | 99.7 | 98.2 | 1.5 | 0.1 | 0.2 | 1367 | Arthropoda; Mandibulata; Pancrustacea; Hexapoda; Insecta; Dicondylia; Pterygota; Neoptera; Endopterygota; Amphiesmenoptera; Lepidoptera; Glossata; Neolepidoptera; Heteroneura; Ditrysia; Obtectomera; Papilionoidea; Nymphalidae; Satyrinae; Satyrini; Maniolina; Maniola; Aphantopus; Maniola hyperantus |
| *Maniola jurtina* | GCF_905333055.1 | NCBI | 99.6 | 98.6 | 1.0 | 0.0 | 0.4 | 1367 | Arthropoda; Mandibulata; Pancrustacea; Hexapoda; Insecta; Dicondylia; Pterygota; Neoptera; Endopterygota; Amphiesmenoptera; Lepidoptera; Glossata; Neolepidoptera; Heteroneura; Ditrysia; Obtectomera; Papilionoidea; Nymphalidae; Satyrinae; Satyrini; Maniolina; Maniola; jurtina species complex; Maniola jurtina |
| *Brenthis ino* | GCA_921882275.1 | NCBI | 98.8 | 98.4 | 0.4 | 0.6 | 0.6 | 1367 | Arthropoda; Mandibulata; Pancrustacea; Hexapoda; Insecta; Dicondylia; Pterygota; Neoptera; Endopterygota; Amphiesmenoptera; Lepidoptera; Glossata; Neolepidoptera; Heteroneura; Ditrysia; Obtectomera; Papilionoidea; Nymphalidae; Heliconiinae; Argynnini; Brenthis; Brenthis ino |
| *Melitaea cinxia* | GCF_905220565.1 | NCBI | 99.3 | 98.5 | 0.8 | 0.1 | 0.6 | 1367 | Arthropoda; Mandibulata; Pancrustacea; Hexapoda; Insecta; Dicondylia; Pterygota; Neoptera; Endopterygota; Amphiesmenoptera; Lepidoptera; Glossata; Neolepidoptera; Heteroneura; Ditrysia; Obtectomera; Papilionoidea; Nymphalidae; Nymphalinae; Melitaeini; Melitaeina; Melitaea; Melitaea cinxia |
| *Nymphalis io* | GCF_905147045.1 | NCBI | 99.7 | 99.2 | 0.5 | 0.0 | 0.3 | 1367 | Arthropoda; Mandibulata; Pancrustacea; Hexapoda; Insecta; Dicondylia; Pterygota; Neoptera; Endopterygota; Amphiesmenoptera; Lepidoptera; Glossata; Neolepidoptera; Heteroneura; Ditrysia; Obtectomera; Papilionoidea; Nymphalidae; Nymphalinae; Nymphalini; Nymphalis; Aglais; Nymphalis io |
| *Vanessa cardui* | GCF_905220365.1 | NCBI | 99.5 | 99.1 | 0.4 | 0.1 | 0.4 | 1367 | Arthropoda; Mandibulata; Pancrustacea; Hexapoda; Insecta; Dicondylia; Pterygota; Neoptera; Endopterygota; Amphiesmenoptera; Lepidoptera; Glossata; Neolepidoptera; Heteroneura; Ditrysia; Obtectomera; Papilionoidea; Nymphalidae; Nymphalinae; Nymphalini; Vanessa; Vanessa atalanta |
| *Vanessa atalanta* | GCF_905147765.1 | NCBI | 99.5 | 99.3 | 0.2 | 0.1 | 0.4 | 1367 | Arthropoda; Mandibulata; Pancrustacea; Hexapoda; Insecta; Dicondylia; Pterygota; Neoptera; Endopterygota; Amphiesmenoptera; Lepidoptera; Glossata; Neolepidoptera; Heteroneura; Ditrysia; Obtectomera; Papilionoidea; Nymphalidae; Nymphalinae; Nymphalini; Vanessa; Vanessa atalanta |
| *Vanessa tameamea* | GCF_037043105.1 | NCBI | 99.9 | 99.5 | 0.4 | 0.0 | 0.1 | 1367 | Arthropoda; Mandibulata; Pancrustacea; Hexapoda; Insecta; Dicondylia; Pterygota; Neoptera; Endopterygota; Amphiesmenoptera; Lepidoptera; Glossata; Neolepidoptera; Heteroneura; Ditrysia; Obtectomera; Papilionoidea; Nymphalidae; Nymphalinae; Nymphalini; Vanessa; Vanessa atalanta |
| *Culicoides brevitarsis* | GCF_036172545.1 | NCBI | 97.0 | 94.3 | 2.7 | 0.7 | 2.3 | 1367 | Arthropoda; Mandibulata; Pancrustacea; Hexapoda; Insecta; Dicondylia; Pterygota; Neoptera; Endopterygota; Diptera; Nematocera; Culicomorpha; Chironomoidea; Ceratopogonidae; Ceratopogoninae; Culicoidini; Culicoides; Avaritia; Culicoides brevitarsis |
| *Clunio marinus* | GCA_900005825.1 | NCBI | 94.1 | 92.4 | 1.7 | 0.7 | 5.2 | 1367 | Arthropoda; Mandibulata; Pancrustacea; Hexapoda; Insecta; Dicondylia; Pterygota; Neoptera; Endopterygota; Diptera; Nematocera; Culicomorpha; Chironomoidea; Chironomidae; Orthocladiinae; Clunio; Clunio marinus |
| *Polypedilum vanderplanki* | GCA_018290095.1 | NCBI | 98.3 | 95.7 | 2.6 | 0.5 | 1.2 | 1367 | Arthropoda; Mandibulata; Pancrustacea; Hexapoda; Insecta; Dicondylia; Pterygota; Neoptera; Endopterygota; Diptera; Nematocera; Culicomorpha; Chironomoidea; Chironomidae; Chironominae; Chironomini; Polypedilum; Polypedilum; Polypedilum vanderplanki |
| *Toxorhynchites rutilus septentrionalis* | GCF_029784135.1 | NCBI | 99.3 | 97.9 | 1.4 | 0.1 | 0.6 | 1367 | Arthropoda; Mandibulata; Pancrustacea; Hexapoda; Insecta; Dicondylia; Pterygota; Neoptera; Endopterygota; Diptera; Nematocera; Culicomorpha; Culicoidea; Culicidae; Toxorhynchitinae; Toxorhynchites; Toxorhynchites rutilus; Toxorhynchites rutilus septentrionalis |
| *Uranotaenia lowii* | GCF_029784155.1 | NCBI | 96.8 | 87.1 | 9.7 | 0.6 | 2.6 | 1367 | Arthropoda; Mandibulata; Pancrustacea; Hexapoda; Insecta; Dicondylia; Pterygota; Neoptera; Endopterygota; Diptera; Nematocera; Culicomorpha; Culicoidea; Culicidae; Culicinae; Uranotaeniini; Uranotaenia; Uranotaenia; Uranotaenia lowii |
| *Malaya genurostris* | GCF_030247185.1 | NCBI | 99.1 | 96.1 | 3.0 | 0.0 | 0.9 | 1367 | Arthropoda; Mandibulata; Pancrustacea; Hexapoda; Insecta; Dicondylia; Pterygota; Neoptera; Endopterygota; Diptera; Nematocera; Culicomorpha; Culicoidea; Culicidae; Culicinae; Sabethini; Malaya; Malaya genurostris |
| *Topomyia yanbarensis* | GCF_030247195.1 | NCBI | 98.8 | 93.1 | 5.7 | 0.3 | 0.9 | 1367 | Arthropoda; Mandibulata; Pancrustacea; Hexapoda; Insecta; Dicondylia; Pterygota; Neoptera; Endopterygota; Diptera; Nematocera; Culicomorpha; Culicoidea; Culicidae; Culicinae; Sabethini; Topomyia; Topomyia yanbarensis |
| *Sabethes cyaneus* | GCF_943734655.1 | NCBI | 98.8 | 97.0 | 1.8 | 0.1 | 1.1 | 1367 | Arthropoda; Mandibulata; Pancrustacea; Hexapoda; Insecta; Dicondylia; Pterygota; Neoptera; Endopterygota; Diptera; Nematocera; Culicomorpha; Culicoidea; Culicidae; Culicinae; Sabethini; Sabethes; Sabethes cyaneus |
| *Wyeomyia smithii* | GCF_029784165.1 | NCBI | 98.3 | 96.8 | 1.5 | 0.1 | 1.6 | 1367 | Arthropoda; Mandibulata; Pancrustacea; Hexapoda; Insecta; Dicondylia; Pterygota; Neoptera; Endopterygota; Diptera; Nematocera; Culicomorpha; Culicoidea; Culicidae; Culicinae; Sabethini; Wyeomyia; Wyeomyia smithii |
| *Culex pipiens pallens* | GCF_016801865.2 | NCBI | 95.9 | 90.3 | 5.6 | 0.6 | 3.5 | 1367 | Arthropoda; Mandibulata; Pancrustacea; Hexapoda; Insecta; Dicondylia; Pterygota; Neoptera; Endopterygota; Diptera; Nematocera; Culicomorpha; Culicoidea; Culicidae; Culicinae; Culicini; Culex; Culex; Culex pipiens complex; Culex pipiens; Culex pipiens pallens |
| *Culex quinquefasciatus* | GCF_015732765.1 | NCBI | 97.0 | 95.0 | 2.0 | 0.3 | 2.7 | 1367 | Arthropoda; Mandibulata; Pancrustacea; Hexapoda; Insecta; Dicondylia; Pterygota; Neoptera; Endopterygota; Diptera; Nematocera; Culicomorpha; Culicoidea; Culicidae; Culicinae; Culicini; Culex; Culex; Culex pipiens complex; Culex quinquefasciatus |
| *Ochlerotatus camptorhynchus* | GCF_037179485.1 | NCBI | 99.4 | 93.2 | 6.2 | 0.1 | 0.5 | 1367 | Arthropoda; Mandibulata; Pancrustacea; Hexapoda; Insecta; Dicondylia; Pterygota; Neoptera; Endopterygota; Diptera; Nematocera; Culicomorpha; Culicoidea; Culicidae; Culicinae; Aedini; Ochlerotatus; Ochlerotatus; Ochlerotatus camptorhynchus |
| *Armigeres subalbatus* | GCF_024139115.2 | NCBI | 99.4 | 91.6 | 7.8 | 0.1 | 0.5 | 1367 | Arthropoda; Mandibulata; Pancrustacea; Hexapoda; Insecta; Dicondylia; Pterygota; Neoptera; Endopterygota; Diptera; Nematocera; Culicomorpha; Culicoidea; Culicidae; Culicinae; Aedini; Armigeres; Armigeres; Armigeres subalbatus |
| *Aedes aegypti* | GCF_002204515.2 | NCBI | 99.5 | 93.4 | 6.1 | 0.1 | 0.4 | 1367 | Arthropoda; Mandibulata; Pancrustacea; Hexapoda; Insecta; Dicondylia; Pterygota; Neoptera; Endopterygota; Diptera; Nematocera; Culicomorpha; Culicoidea; Culicidae; Culicinae; Aedini; Aedes; Stegomyia; Aedes aegypti |
| *Aedes albopictus* | GCF_035046485.1 | NCBI | 98.4 | 89.8 | 8.6 | 0.3 | 1.3 | 1367 | Arthropoda; Mandibulata; Pancrustacea; Hexapoda; Insecta; Dicondylia; Pterygota; Neoptera; Endopterygota; Diptera; Nematocera; Culicomorpha; Culicoidea; Culicidae; Culicinae; Aedini; Aedes; Stegomyia; Aedes albopictus |
| *Anopheles coustani* | GCF_943734705.1 | NCBI | 99.4 | 97.5 | 1.9 | 0.1 | 0.5 | 1367 | Arthropoda; Mandibulata; Pancrustacea; Hexapoda; Insecta; Dicondylia; Pterygota; Neoptera; Endopterygota; Diptera; Nematocera; Culicomorpha; Culicoidea; Culicidae; Anophelinae; Anopheles; Anopheles; Laticorn; Myzorhynchus; coustani group; Anopheles coustani |
| *Anopheles ziemanni* | GCF_943734765.1 | NCBI | 99.4 | 97.4 | 2.0 | 0.0 | 0.6 | 1367 | Arthropoda; Mandibulata; Pancrustacea; Hexapoda; Insecta; Dicondylia; Pterygota; Neoptera; Endopterygota; Diptera; Nematocera; Culicomorpha; Culicoidea; Culicidae; Anophelinae; Anopheles; Anopheles; Laticorn; Myzorhynchus; coustani group; Anopheles ziemanni |
| *Anopheles bellator* | GCF_943735745.2 | NCBI | 99.0 | 97.0 | 2.0 | 0.1 | 0.9 | 1367 | Arthropoda; Mandibulata; Pancrustacea; Hexapoda; Insecta; Dicondylia; Pterygota; Neoptera; Endopterygota; Diptera; Nematocera; Culicomorpha; Culicoidea; Culicidae; Anophelinae; Anopheles; Anopheles |
| *Anopheles cruzii* | GCF_943734635.1 | NCBI | 99.0 | 95.7 | 3.3 | 0.2 | 0.8 | 1367 | Arthropoda; Mandibulata; Pancrustacea; Hexapoda; Insecta; Dicondylia; Pterygota; Neoptera; Endopterygota; Diptera; Nematocera; Culicomorpha; Culicoidea; Culicidae; Anophelinae; Anopheles; Anopheles |
| *Anopheles darlingi* | GCF_943734745.1 | NCBI | 99.1 | 97.1 | 2.0 | 0.1 | 0.8 | 1367 | Arthropoda; Mandibulata; Pancrustacea; Hexapoda; Insecta; Dicondylia; Pterygota; Neoptera; Endopterygota; Diptera; Nematocera; Culicomorpha; Culicoidea; Culicidae; Anophelinae; Anopheles; Nyssorhynchus; argyritarsis section; argyritarsis series; darlingi group; Anopheles darlingi |
| *Anopheles albimanus* | GCF_013758885.1 | NCBI | 99.2 | 97.7 | 1.5 | 0.1 | 0.7 | 1367 | Arthropoda; Mandibulata; Pancrustacea; Hexapoda; Insecta; Dicondylia; Pterygota; Neoptera; Endopterygota; Diptera; Nematocera; Culicomorpha; Culicoidea; Culicidae; Anophelinae; Anopheles; Nyssorhynchus; albimanus section; albimanus series; Anopheles albimanus |
| *Anopheles aquasalis* | GCF_943734665.1 | NCBI | 99.3 | 97.8 | 1.5 | 0.2 | 0.5 | 1367 | Arthropoda; Mandibulata; Pancrustacea; Hexapoda; Insecta; Dicondylia; Pterygota; Neoptera; Endopterygota; Diptera; Nematocera; Culicomorpha; Culicoidea; Culicidae; Anophelinae; Anopheles; Nyssorhynchus; albimanus section; oswaldoi series; oswaldoi group; oswaldoi subgroup; Anopheles aquasalis |
| *Anopheles maculipalpis* | GCF_943734695.1 | NCBI | 99.3 | 98.1 | 1.2 | 0.1 | 0.6 | 1367 | Arthropoda; Mandibulata; Pancrustacea; Hexapoda; Insecta; Dicondylia; Pterygota; Neoptera; Endopterygota; Diptera; Nematocera; Culicomorpha; Culicoidea; Culicidae; Anophelinae; Anopheles; Cellia; Neocellia; Anopheles maculipalpis |
| *Anopheles stephensi* | GCF_013141755.1 | NCBI | 99.2 | 94.4 | 4.8 | 0.1 | 0.7 | 1367 | Arthropoda; Mandibulata; Pancrustacea; Hexapoda; Insecta; Dicondylia; Pterygota; Neoptera; Endopterygota; Diptera; Nematocera; Culicomorpha; Culicoidea; Culicidae; Anophelinae; Anopheles; Anopheles |
| *Anopheles nili* | GCF_943737925.1 | NCBI | 98.9 | 97.7 | 1.2 | 0.1 | 1.0 | 1367 | Arthropoda; Mandibulata; Pancrustacea; Hexapoda; Insecta; Dicondylia; Pterygota; Neoptera; Endopterygota; Diptera; Nematocera; Culicomorpha; Culicoidea; Culicidae; Anophelinae; Anopheles; Cellia; Neomyzomyia; ardensis group; nili species complex; Anopheles nili |
| *Anopheles funestus* | GCF_943734845.2 | NCBI | 99.4 | 98.1 | 1.3 | 0.0 | 0.6 | 1367 | Arthropoda; Mandibulata; Pancrustacea; Hexapoda; Insecta; Dicondylia; Pterygota; Neoptera; Endopterygota; Diptera; Nematocera; Culicomorpha; Culicoidea; Culicidae; Anophelinae; Anopheles; Cellia; Myzomyia; funestus group; Anopheles funestus |
| *Anopheles moucheti* | GCF_943734755.1 | NCBI | 98.9 | 97.4 | 1.5 | 0.0 | 1.1 | 1367 | Arthropoda; Mandibulata; Pancrustacea; Hexapoda; Insecta; Dicondylia; Pterygota; Neoptera; Endopterygota; Diptera; Nematocera; Culicomorpha; Culicoidea; Culicidae; Anophelinae; Anopheles; Cellia; Myzomyia; Anopheles moucheti |
| *Anopheles merus* | GCF_017562075.2 | NCBI | 99.1 | 91.6 | 7.5 | 0.1 | 0.8 | 1367 | Arthropoda; Mandibulata; Pancrustacea; Hexapoda; Insecta; Dicondylia; Pterygota; Neoptera; Endopterygota; Diptera; Nematocera; Culicomorpha; Culicoidea; Culicidae; Anophelinae; Anopheles; Cellia; Pyretophorus; gambiae species complex; Anopheles merus |
| *Anopheles arabiensis* | GCF_016920715.1 | NCBI | 99.3 | 97.8 | 1.5 | 0.1 | 0.6 | 1367 | Arthropoda; Mandibulata; Pancrustacea; Hexapoda; Insecta; Dicondylia; Pterygota; Neoptera; Endopterygota; Diptera; Nematocera; Culicomorpha; Culicoidea; Culicidae; Anophelinae; Anopheles; Cellia; Pyretophorus; gambiae species complex; Anopheles arabiensis |
| *Anopheles coluzzii* | GCF_943734685.1 | NCBI | 99.2 | 98.2 | 1.0 | 0.1 | 0.7 | 1367 | Arthropoda; Mandibulata; Pancrustacea; Hexapoda; Insecta; Dicondylia; Pterygota; Neoptera; Endopterygota; Diptera; Nematocera; Culicomorpha; Culicoidea; Culicidae; Anophelinae; Anopheles; Cellia; Pyretophorus; gambiae species complex; Anopheles coluzzii |
| *Anopheles gambiae* | GCF_943734735.2 | NCBI | 99.0 | 97.8 | 1.2 | 0.1 | 0.9 | 1367 | Arthropoda; Mandibulata; Pancrustacea; Hexapoda; Insecta; Dicondylia; Pterygota; Neoptera; Endopterygota; Diptera; Nematocera; Culicomorpha; Culicoidea; Culicidae; Anophelinae; Anopheles; Cellia; Pyretophorus; gambiae species complex; Anopheles gambiae |
| *Lutzomyia longipalpis* | GCF_024334085.1 | NCBI | 98.6 | 97.7 | 0.9 | 0.1 | 1.3 | 1367 | Arthropoda; Mandibulata; Pancrustacea; Hexapoda; Insecta; Dicondylia; Pterygota; Neoptera; Endopterygota; Diptera; Nematocera; Psychodomorpha; Psychodoidea; Psychodidae; Phlebotominae; Lutzomyia; Lutzomyia; Lutzomyia longipalpis |
| *Phlebotomus argentipes* | GCF_947086385.1 | NCBI | 98.7 | 96.4 | 2.3 | 0.1 | 1.2 | 1367 | Arthropoda; Mandibulata; Pancrustacea; Hexapoda; Insecta; Dicondylia; Pterygota; Neoptera; Endopterygota; Diptera; Nematocera; Psychodomorpha; Psychodoidea; Psychodidae; Phlebotominae; Phlebotomus; Euphlebotomus; Phlebotomus argentipes |
| *Phlebotomus papatasi* | GCF_024763615.1 | NCBI | 97.2 | 95.2 | 2.0 | 0.4 | 2.4 | 1367 | Arthropoda; Mandibulata; Pancrustacea; Hexapoda; Insecta; Dicondylia; Pterygota; Neoptera; Endopterygota; Diptera; Nematocera; Psychodomorpha; Psychodoidea; Psychodidae; Phlebotominae; Phlebotomus; Phlebotomus; Phlebotomus papatasi |
| *Bradysia coprophila* | GCF_014529535.1 | NCBI | 99.6 | 95.5 | 4.1 | 0.1 | 0.3 | 1367 | Arthropoda; Mandibulata; Pancrustacea; Hexapoda; Insecta; Dicondylia; Pterygota; Neoptera; Endopterygota; Diptera; Nematocera; Bibionomorpha; Sciaroidea; Sciaridae; Bradysia |
| *Contarinia nasturtii* | GCF_009176525.2 | NCBI | 98.6 | 96.8 | 1.8 | 0.3 | 1.1 | 1367 | Arthropoda; Mandibulata; Pancrustacea; Hexapoda; Insecta; Dicondylia; Pterygota; Neoptera; Endopterygota; Diptera; Nematocera; Bibionomorpha; Sciaroidea; Cecidomyiidae; Cecidomyiinae; Cecidomyiidi; Cecidomyiini; Contarinia; Contarinia nasturtii |
| *Sitodiplosis mosellana* | GCF_009176505.1 | NCBI | 98.7 | 95.9 | 2.8 | 0.1 | 1.2 | 1367 | Arthropoda; Mandibulata; Pancrustacea; Hexapoda; Insecta; Dicondylia; Pterygota; Neoptera; Endopterygota; Diptera; Nematocera; Bibionomorpha; Sciaroidea; Cecidomyiidae; Cecidomyiinae; Cecidomyiidi; Cecidomyiini; Sitodiplosis |
| *Hermetia illucens* | GCF_905115235.1 | NCBI | 99.4 | 98.8 | 0.6 | 0.0 | 0.6 | 1367 | Arthropoda; Mandibulata; Pancrustacea; Hexapoda; Insecta; Dicondylia; Pterygota; Neoptera; Endopterygota; Diptera; Brachycera; Stratiomyomorpha; Stratiomyidae; Hermetiinae; Hermetia; Hermetia illucens |
| *Condylostylus longicornis* | GCF_029603195.1 | NCBI | 98.7 | 97.4 | 1.3 | 0.1 | 1.2 | 1367 | Arthropoda; Mandibulata; Pancrustacea; Hexapoda; Insecta; Dicondylia; Pterygota; Neoptera; Endopterygota; Diptera; Brachycera; Muscomorpha; Eremoneura; Empidoidea; Dolichopodidae; Sciapodinae; Condylostylus; Condylostylus longicornis |
| *Episyrphus balteatus* | GCF_945859705.1 | NCBI | 99.1 | 98.2 | 0.9 | 0.0 | 0.9 | 1367 | Arthropoda; Mandibulata; Pancrustacea; Hexapoda; Insecta; Dicondylia; Pterygota; Neoptera; Endopterygota; Diptera; Brachycera; Muscomorpha; Eremoneura; Cyclorrhapha; Aschiza; Syrphoidea; Syrphidae; Syrphinae; Syrphini; Episyrphus; Episyrphus balteatus |
| *Eupeodes corollae* | GCF_945859685.1 | NCBI | 99.4 | 98.3 | 1.1 | 0.0 | 0.6 | 1367 | Arthropoda; Mandibulata; Pancrustacea; Hexapoda; Insecta; Dicondylia; Pterygota; Neoptera; Endopterygota; Diptera; Brachycera; Muscomorpha; Eremoneura; Cyclorrhapha; Aschiza; Syrphoidea; Syrphidae; Syrphinae; Syrphini; Eupeodes; Eupeodes; Eupeodes corollae |
| *Anastrepha ludens* | GCF_028408465.1 | NCBI | 99.8 | 98.8 | 1.0 | 0.0 | 0.2 | 1367 | Arthropoda; Mandibulata; Pancrustacea; Hexapoda; Insecta; Dicondylia; Pterygota; Neoptera; Endopterygota; Diptera; Brachycera; Muscomorpha; Eremoneura; Cyclorrhapha; Schizophora; Acalyptratae; Tephritoidea; Tephritidae; Trypetinae; Toxotrypanini; Anastrepha; Anastrepha ludens |
| *Anastrepha obliqua* | GCF_027943255.1 | NCBI | 99.6 | 97.5 | 2.1 | 0.0 | 0.4 | 1367 | Arthropoda; Mandibulata; Pancrustacea; Hexapoda; Insecta; Dicondylia; Pterygota; Neoptera; Endopterygota; Diptera; Brachycera; Muscomorpha; Eremoneura; Cyclorrhapha; Schizophora; Acalyptratae; Tephritoidea; Tephritidae; Trypetinae; Toxotrypanini; Anastrepha; Anastrepha obliqua |
| *Ceratitis capitata* | GCF_000347755.3 | NCBI | 99.7 | 99.3 | 0.4 | 0.1 | 0.2 | 1367 | Arthropoda; Mandibulata; Pancrustacea; Hexapoda; Insecta; Dicondylia; Pterygota; Neoptera; Endopterygota; Diptera; Brachycera; Muscomorpha; Eremoneura; Cyclorrhapha; Schizophora; Acalyptratae; Tephritoidea; Tephritidae; Dacinae; Ceratitidini; Ceratitis; Ceratitis; Ceratitis capitata |
| *Zeugodacus cucurbitae* | GCF_028554725.1 | NCBI | 99.3 | 98.5 | 0.8 | 0.1 | 0.6 | 1367 | Arthropoda; Mandibulata; Pancrustacea; Hexapoda; Insecta; Dicondylia; Pterygota; Neoptera; Endopterygota; Diptera; Brachycera; Muscomorpha; Eremoneura; Cyclorrhapha; Schizophora; Acalyptratae; Tephritoidea; Tephritidae; Dacinae; Dacini; Zeugodacus; Zeugodacus; Zeugodacus cucurbitae |
| *Bactrocera tryoni* | GCF_016617805.1 | NCBI | 99.2 | 98.0 | 1.2 | 0.2 | 0.6 | 1367 | Arthropoda; Mandibulata; Pancrustacea; Hexapoda; Insecta; Dicondylia; Pterygota; Neoptera; Endopterygota; Diptera; Brachycera; Muscomorpha; Eremoneura; Cyclorrhapha; Schizophora; Acalyptratae; Tephritoidea; Tephritidae; Dacinae; Dacini; Bactrocera; Bactrocera; Bactrocera tyroni species complex; Bactrocera tryoni |
| *Bactrocera dorsalis* | GCF_023373825.1 | NCBI | 99.2 | 98.3 | 0.9 | 0.0 | 0.8 | 1367 | Arthropoda; Mandibulata; Pancrustacea; Hexapoda; Insecta; Dicondylia; Pterygota; Neoptera; Endopterygota; Diptera; Brachycera; Muscomorpha; Eremoneura; Cyclorrhapha; Schizophora; Acalyptratae; Tephritoidea; Tephritidae; Dacinae; Dacini; Bactrocera; Bactrocera; Bactrocera dorsalis complex; Bactrocera dorsalis |
| *Bactrocera latifrons* | GCF_001853355.1 | NCBI | 99.5 | 98.5 | 1.0 | 0.0 | 0.5 | 1367 | Arthropoda; Mandibulata; Pancrustacea; Hexapoda; Insecta; Dicondylia; Pterygota; Neoptera; Endopterygota; Diptera; Brachycera; Muscomorpha; Eremoneura; Cyclorrhapha; Schizophora; Acalyptratae; Tephritoidea; Tephritidae; Dacinae; Dacini; Bactrocera; Bactrocera; Bactrocera latifrons |
| *Glossina fuscipes* | GCF_014805625.2 | NCBI | 99.4 | 95.5 | 3.9 | 0.2 | 0.4 | 1367 | Arthropoda; Mandibulata; Pancrustacea; Hexapoda; Insecta; Dicondylia; Pterygota; Neoptera; Endopterygota; Diptera; Brachycera; Muscomorpha; Eremoneura; Cyclorrhapha; Schizophora; Calyptratae; Hippoboscoidea; Glossinidae; Glossina; Nemorhina; Glossina fuscipes |
| *Calliphora vicina* | GCF_958450345.1 | NCBI | 99.7 | 98.8 | 0.9 | 0.0 | :0.3 | 1367 | Arthropoda; Mandibulata; Pancrustacea; Hexapoda; Insecta; Dicondylia; Pterygota; Neoptera; Endopterygota; Diptera; Brachycera; Muscomorpha; Eremoneura; Cyclorrhapha; Schizophora; Calyptratae; Oestroidea; Calliphoridae; Calliphorinae; Calliphora; Calliphora vicina |
| *Lucilia cuprina* | GCF_022045245.1 | NCBI | 98.6 | 97.4 | 1.2 | 0.8 | 0.6 | 1367 | Arthropoda; Mandibulata; Pancrustacea; Hexapoda; Insecta; Dicondylia; Pterygota; Neoptera; Endopterygota; Diptera; Brachycera; Muscomorpha; Eremoneura; Cyclorrhapha; Schizophora; Calyptratae; Oestroidea; Calliphoridae; Luciliinae; Lucilia; Lucilia cuprina |
| *Lucilia sericata* | GCF_015586225.1 | NCBI | 97.5 | 90.8 | 6.7 | 0.6 | 1.9 | 1367 | Arthropoda; Mandibulata; Pancrustacea; Hexapoda; Insecta; Dicondylia; Pterygota; Neoptera; Endopterygota; Diptera; Brachycera; Muscomorpha; Eremoneura; Cyclorrhapha; Schizophora; Calyptratae; Oestroidea; Calliphoridae; Luciliinae; Lucilia; Lucilia sericata |
| *Stomoxys calcitrans* | GCF_963082655.1 | NCBI | 99.5 | 98.5 | 1.0 | 0.1 | 0.4 | 1367 | Arthropoda; Mandibulata; Pancrustacea; Hexapoda; Insecta; Dicondylia; Pterygota; Neoptera; Endopterygota; Diptera; Brachycera; Muscomorpha; Eremoneura; Cyclorrhapha; Schizophora; Calyptratae; Muscoidea; Muscidae; Muscinae; Stomoxyini; Stomoxys; Stomoxys calcitrans |
| *Musca domestica* | GCF_030504385.1 | NCBI | 98.8 | 77.5 | 21.3 | 0.1 | 1.1 | 1367 | Arthropoda; Mandibulata; Pancrustacea; Hexapoda; Insecta; Dicondylia; Pterygota; Neoptera; Endopterygota; Diptera; Brachycera; Muscomorpha; Eremoneura; Cyclorrhapha; Schizophora; Calyptratae; Muscoidea; Muscidae; Muscinae; Muscini; Musca; Musca; Musca domestica |
| *Musca vetustissima* | GCF_032173495.1 | NCBI | 99.2 | 83.4 | 15.8 | 0.2 | 0.6 | 1367 | Arthropoda; Mandibulata; Pancrustacea; Hexapoda; Insecta; Dicondylia; Pterygota; Neoptera; Endopterygota; Diptera; Brachycera; Muscomorpha; Eremoneura; Cyclorrhapha; Schizophora; Calyptratae; Muscoidea; Muscidae; Muscinae; Muscini; Musca; Byomya; Musca vetustissima |
| *Scaptodrosophila lebanonensis* | GCF_003285725.1 | NCBI | 99.7 | 97.8 | 1.9 | 0.1 | 0.2 | 1367 | Arthropoda; Mandibulata; Pancrustacea; Hexapoda; Insecta; Dicondylia; Pterygota; Neoptera; Endopterygota; Diptera; Brachycera; Muscomorpha; Eremoneura; Cyclorrhapha; Schizophora; Acalyptratae; Ephydroidea; Drosophilidae; Drosophilinae; Colocasiomyini; Scaptodrosophila; victoria group; Scaptodrosophila lebanonensis |
| *Drosophila sulfurigaster albostrigata* | GCF_023558435.1 | NCBI | 99.9 | 98.2 | 1.7 | 0.0 | 0.1 | 1367 | Arthropoda; Mandibulata; Pancrustacea; Hexapoda; Insecta; Dicondylia; Pterygota; Neoptera; Endopterygota; Diptera; Brachycera; Muscomorpha; Eremoneura; Cyclorrhapha; Schizophora; Acalyptratae; Ephydroidea; Drosophilidae; Drosophilinae; Drosophilini; Drosophila; Drosophila; immigrans group; nasuta subgroup; Drosophila sulfurigaster; Drosophila sulfurigaster albostrigata |
| *Drosophila albomicans* | GCF_009650485.2 | NCBI | 100.0 | 98.2 | 1.8 | 0.0 | 0.0 | 1367 | Arthropoda; Mandibulata; Pancrustacea; Hexapoda; Insecta; Dicondylia; Pterygota; Neoptera; Endopterygota; Diptera; Brachycera; Muscomorpha; Eremoneura; Cyclorrhapha; Schizophora; Acalyptratae; Ephydroidea; Drosophilidae; Drosophilinae; Drosophilini; Drosophila; Drosophila; immigrans group; nasuta subgroup; Drosophila albomicans |
| *Drosophila nasuta* | GCF_023558535.2 | NCBI | 100.0 | 98.5 | 1.5 | 0.0 | 0.0 | 1367 | Arthropoda; Mandibulata; Pancrustacea; Hexapoda; Insecta; Dicondylia; Pterygota; Neoptera; Endopterygota; Diptera; Brachycera; Muscomorpha; Eremoneura; Cyclorrhapha; Schizophora; Acalyptratae; Ephydroidea; Drosophilidae; Drosophilinae; Drosophilini; Drosophila; Drosophila; immigrans group; nasuta subgroup; Drosophila nasuta |
| *Drosophila grimshawi* | GCF_018153295.1 | NCBI | 99.9 | 98.3 | 1.6 | 0.0 | 0.1 | 1367 | Arthropoda; Mandibulata; Pancrustacea; Hexapoda; Insecta; Dicondylia; Pterygota; Neoptera; Endopterygota; Diptera; Brachycera; Muscomorpha; Eremoneura; Cyclorrhapha; Schizophora; Acalyptratae; Ephydroidea; Drosophilidae; Drosophilinae; Drosophilini; Drosophila; Hawaiian Drosophila; picture wing clade; grimshawi clade; grimshawi group; grimshawi subgroup; Drosophila grimshawi |
| *Drosophila montana* | GCF_035044405.1 | NCBI | 99.9 | 97.7 | 2.2 | 0.0 | 0.1 | 1367 | Arthropoda; Mandibulata; Pancrustacea; Hexapoda; Insecta; Dicondylia; Pterygota; Neoptera; Endopterygota; Diptera; Brachycera; Muscomorpha; Eremoneura; Cyclorrhapha; Schizophora; Acalyptratae; Ephydroidea; Drosophilidae; Drosophilinae; Drosophilini; Drosophila; Drosophila; virilis group; Drosophila montana |
| *Drosophila novamexicana* | GCF_003285875.2 | NCBI | 99.1 | 97.3 | 1.8 | 0.1 | 0.8 | 1367 | Arthropoda; Mandibulata; Pancrustacea; Hexapoda; Insecta; Dicondylia; Pterygota; Neoptera; Endopterygota; Diptera; Brachycera; Muscomorpha; Eremoneura; Cyclorrhapha; Schizophora; Acalyptratae; Ephydroidea; Drosophilidae; Drosophilinae; Drosophilini; Drosophila; Drosophila; virilis group; Drosophila novamexicana |
| *Drosophila virilis* | GCF_003285735.1 | NCBI | 99.9 | 96.3 | 3.6 | 0.0 | 0.1 | 1367 | Arthropoda; Mandibulata; Pancrustacea; Hexapoda; Insecta; Dicondylia; Pterygota; Neoptera; Endopterygota; Diptera; Brachycera; Muscomorpha; Eremoneura; Cyclorrhapha; Schizophora; Acalyptratae; Ephydroidea; Drosophilidae; Drosophilinae; Drosophilini; Drosophila |
| *Drosophila hydei* | GCF_003285905.1 | NCBI | 99.7 | 96.7 | 3.0 | 0.1 | 0.2 | 1367 | Arthropoda; Mandibulata; Pancrustacea; Hexapoda; Insecta; Dicondylia; Pterygota; Neoptera; Endopterygota; Diptera; Brachycera; Muscomorpha; Eremoneura; Cyclorrhapha; Schizophora; Acalyptratae; Ephydroidea; Drosophilidae; Drosophilinae; Drosophilini; Drosophila; Drosophila; repleta group; hydei subgroup; Drosophila hydei |
| *Drosophila navojoa* | GCF_001654015.2 | NCBI | 99.1 | 98.0 | 1.1 | 0.4 | 0.5 | 1367 | Arthropoda; Mandibulata; Pancrustacea; Hexapoda; Insecta; Dicondylia; Pterygota; Neoptera; Endopterygota; Diptera; Brachycera; Muscomorpha; Eremoneura; Cyclorrhapha; Schizophora; Acalyptratae; Ephydroidea; Drosophilidae; Drosophilinae; Drosophilini; Drosophila; Drosophila; repleta group; mulleri subgroup; mojavensis species complex; Drosophila navojoa |
| *Drosophila arizonae* | GCF_001654025.1 | NCBI | 96.1 | 94.9 | 1.2 | 0.9 | 3.0 | 1367 | Arthropoda; Mandibulata; Pancrustacea; Hexapoda; Insecta; Dicondylia; Pterygota; Neoptera; Endopterygota; Diptera; Brachycera; Muscomorpha; Eremoneura; Cyclorrhapha; Schizophora; Acalyptratae; Ephydroidea; Drosophilidae; Drosophilinae; Drosophilini; Drosophila; Drosophila; repleta group; mulleri subgroup; mojavensis species complex; Drosophila arizonae |
| *Drosophila mojavensis* | GCF_018153725.1 | NCBI | 100.0 | 98.5 | 1.5 | 0.0 | 0.0 | 1367 | Arthropoda; Mandibulata; Pancrustacea; Hexapoda; Insecta; Dicondylia; Pterygota; Neoptera; Endopterygota; Diptera; Brachycera; Muscomorpha; Eremoneura; Cyclorrhapha; Schizophora; Acalyptratae; Ephydroidea; Drosophilidae; Drosophilinae; Drosophilini; Drosophila; Drosophila; repleta group; mulleri subgroup; mojavensis species complex; Drosophila mojavensis |
| *Drosophila obscura* | GCF_018151105.1 | NCBI | 99.8 | 98.0 | 1.8 | 0.1 | 0.1 | 1367 | Arthropoda; Mandibulata; Pancrustacea; Hexapoda; Insecta; Dicondylia; Pterygota; Neoptera; Endopterygota; Diptera; Brachycera; Muscomorpha; Eremoneura; Cyclorrhapha; Schizophora; Acalyptratae; Ephydroidea; Drosophilidae; Drosophilinae; Drosophilini; Drosophila; Sophophora; obscura group; obscura subgroup; Drosophila obscura |
| *Drosophila guanche* | GCF_900245975.1 | NCBI | 99.5 | 98.4 | 1.1 | 0.1 | 0.4 | 1367 | Arthropoda; Mandibulata; Pancrustacea; Hexapoda; Insecta; Dicondylia; Pterygota; Neoptera; Endopterygota; Diptera; Brachycera; Muscomorpha; Eremoneura; Cyclorrhapha; Schizophora; Acalyptratae; Ephydroidea; Drosophilidae; Drosophilinae; Drosophilini; Drosophila; Sophophora; obscura group; obscura subgroup; Drosophila guanche |
| *Drosophila subobscura* | GCF_008121235.1 | NCBI | 99.5 | 98.2 | 1.3 | 0.1 | 0.4 | 1367 | Arthropoda; Mandibulata; Pancrustacea; Hexapoda; Insecta; Dicondylia; Pterygota; Neoptera; Endopterygota; Diptera; Brachycera; Muscomorpha; Eremoneura; Cyclorrhapha; Schizophora; Acalyptratae; Ephydroidea; Drosophilidae; Drosophilinae; Drosophilini; Drosophila; Sophophora; obscura group; obscura subgroup; Drosophila subobscura |
| *Drosophila miranda* | GCF_003369915.1 | NCBI | 99.9 | 85.3 | 14.6 | 0.0 | 0.1 | 1367 | Arthropoda; Mandibulata; Pancrustacea; Hexapoda; Insecta; Dicondylia; Pterygota; Neoptera; Endopterygota; Diptera; Brachycera; Muscomorpha; Eremoneura; Cyclorrhapha; Schizophora; Acalyptratae; Ephydroidea; Drosophilidae; Drosophilinae; Drosophilini; Drosophila; Sophophora; obscura group; pseudoobscura subgroup; Drosophila miranda |
| *Drosophila persimilis* | GCF_003286085.1 | NCBI | 99.6 | 97.3 | 2.3 | 0.1 | 0.3 | 1367 | Arthropoda; Mandibulata; Pancrustacea; Hexapoda; Insecta; Dicondylia; Pterygota; Neoptera; Endopterygota; Diptera; Brachycera; Muscomorpha; Eremoneura; Cyclorrhapha; Schizophora; Acalyptratae; Ephydroidea; Drosophilidae; Drosophilinae; Drosophilini; Drosophila; Sophophora; obscura group; pseudoobscura subgroup; Drosophila persimilis |
| *Drosophila pseudoobscura* | GCF_009870125.1 | NCBI | 100.0 | 98.0 | 2.0 | 0.0 | 0.0 | 1367 | Arthropoda; Mandibulata; Pancrustacea; Hexapoda; Insecta; Dicondylia; Pterygota; Neoptera; Endopterygota; Diptera; Brachycera; Muscomorpha; Eremoneura; Cyclorrhapha; Schizophora; Acalyptratae; Ephydroidea; Drosophilidae; Drosophilinae; Drosophilini; Drosophila; Sophophora; obscura group; pseudoobscura subgroup; Drosophila pseudoobscura |
| *Drosophila ananassae* | GCF_017639315.1 | NCBI | 99.7 | 98.6 | 1.1 | 0.0 | 0.3 | 1367 | Arthropoda; Mandibulata; Pancrustacea; Hexapoda; Insecta; Dicondylia; Pterygota; Neoptera; Endopterygota; Diptera; Brachycera; Muscomorpha; Eremoneura; Cyclorrhapha; Schizophora; Acalyptratae; Ephydroidea; Drosophilidae; Drosophilinae; Drosophilini; Drosophila; Sophophora; melanogaster group; ananassae subgroup; ananassae species complex; Drosophila ananassae |
| *Drosophila bipectinata* | GCF_018153845.1 | NCBI | 99.8 | 98.2 | 1.6 | 0.0 | 0.2 | 1367 | Arthropoda; Mandibulata; Pancrustacea; Hexapoda; Insecta; Dicondylia; Pterygota; Neoptera; Endopterygota; Diptera; Brachycera; Muscomorpha; Eremoneura; Cyclorrhapha; Schizophora; Acalyptratae; Ephydroidea; Drosophilidae; Drosophilinae; Drosophilini; Drosophila; Sophophora; melanogaster group; ananassae subgroup; bipectinata species complex; Drosophila bipectinata |
| *Drosophila kikkawai* | GCF_018152535.1 | NCBI | 99.9 | 98.3 | 1.6 | 0.0 | 0.1 | 1367 | Arthropoda; Mandibulata; Pancrustacea; Hexapoda; Insecta; Dicondylia; Pterygota; Neoptera; Endopterygota; Diptera; Brachycera; Muscomorpha; Eremoneura; Cyclorrhapha; Schizophora; Acalyptratae; Ephydroidea; Drosophilidae; Drosophilinae; Drosophilini; Drosophila; Sophophora; melanogaster group; montium subgroup; Drosophila kikkawai |
| *Drosophila serrata* | GCF_002093755.2 | NCBI | 99.7 | 96.3 | 3.4 | 0.0 | 0.3 | 1367 | Arthropoda; Mandibulata; Pancrustacea; Hexapoda; Insecta; Dicondylia; Pterygota; Neoptera; Endopterygota; Diptera; Brachycera; Muscomorpha; Eremoneura; Cyclorrhapha; Schizophora; Acalyptratae; Ephydroidea; Drosophilidae; Drosophilinae; Drosophilini; Drosophila; Sophophora; melanogaster group; montium subgroup; Drosophila serrata species complex; Drosophila serrata |
| *Drosophila ficusphila* | GCF_018152265.1 | NCBI | 99.7 | 98.5 | 1.2 | 0.1 | 0.2 | 1367 | Arthropoda; Mandibulata; Pancrustacea; Hexapoda; Insecta; Dicondylia; Pterygota; Neoptera; Endopterygota; Diptera; Brachycera; Muscomorpha; Eremoneura; Cyclorrhapha; Schizophora; Acalyptratae; Ephydroidea; Drosophilidae; Drosophilinae; Drosophilini; Drosophila; Sophophora; melanogaster group; ficusphila subgroup; Drosophila ficusphila |
| *Drosophila elegans* | GCF_018152505.1 | NCBI | 99.8 | 99.1 | 0.7 | 0.1 | 0.1 | 1367 | Arthropoda; Mandibulata; Pancrustacea; Hexapoda; Insecta; Dicondylia; Pterygota; Neoptera; Endopterygota; Diptera; Brachycera; Muscomorpha; Eremoneura; Cyclorrhapha; Schizophora; Acalyptratae; Ephydroidea; Drosophilidae; Drosophilinae; Drosophilini; Drosophila; Sophophora; melanogaster group; elegans subgroup (in: flies); Drosophila elegans |
| *Drosophila rhopaloa* | GCF_018152115.1 | NCBI | 99.1 | 97.9 | 1.2 | 0.0 | 0.9 | 1367 | Arthropoda; Mandibulata; Pancrustacea; Hexapoda; Insecta; Dicondylia; Pterygota; Neoptera; Endopterygota; Diptera; Brachycera; Muscomorpha; Eremoneura; Cyclorrhapha; Schizophora; Acalyptratae; Ephydroidea; Drosophilidae; Drosophilinae; Drosophilini; Drosophila; Sophophora; melanogaster group; rhopaloa subgroup; Drosophila rhopaloa |
| *Drosophila takahashii* | GCF_018152695.1 | NCBI | 98.7 | 97.5 | 1.2 | 0.1 | 1.2 | 1367 | Arthropoda; Mandibulata; Pancrustacea; Hexapoda; Insecta; Dicondylia; Pterygota; Neoptera; Endopterygota; Diptera; Brachycera; Muscomorpha; Eremoneura; Cyclorrhapha; Schizophora; Acalyptratae; Ephydroidea; Drosophilidae; Drosophilinae; Drosophilini; Drosophila; Sophophora; melanogaster group; takahashii subgroup; Drosophila takahashii |
| *Drosophila biarmipes* | GCF_025231255.1 | NCBI | 99.8 | 99.1 | 0.7 | 0.0 | 0.2 | 1367 | Arthropoda; Mandibulata; Pancrustacea; Hexapoda; Insecta; Dicondylia; Pterygota; Neoptera; Endopterygota; Diptera; Brachycera; Muscomorpha; Eremoneura; Cyclorrhapha; Schizophora; Acalyptratae; Ephydroidea; Drosophilidae; Drosophilinae; Drosophilini; Drosophila |
| *Drosophila subpulchrella* | GCF_014743375.2 | NCBI | 99.9 | 97.9 | 2.0 | 0.1 | 0.0 | 1367 | Arthropoda; Mandibulata; Pancrustacea; Hexapoda; Insecta; Dicondylia; Pterygota; Neoptera; Endopterygota; Diptera; Brachycera; Muscomorpha; Eremoneura; Cyclorrhapha; Schizophora; Acalyptratae; Ephydroidea; Drosophilidae; Drosophilinae; Drosophilini; Drosophila |
| *Drosophila suzukii* | GCF_013340165.1 | NCBI | 99.7 | 95.5 | 4.2 | 0.1 | 0.2 | 1367 | Arthropoda; Mandibulata; Pancrustacea; Hexapoda; Insecta; Dicondylia; Pterygota; Neoptera; Endopterygota; Diptera; Brachycera; Muscomorpha; Eremoneura; Cyclorrhapha; Schizophora; Acalyptratae; Ephydroidea; Drosophilidae; Drosophilinae; Drosophilini; Drosophila; Sophophora; melanogaster group; suzukii subgroup; Drosophila suzukii |
| *Drosophila eugracilis* | GCF_018153835.1 | NCBI | 99.9 | 98.8 | 1.1 | 0.0 | 0.1 | 1367 | Arthropoda; Mandibulata; Pancrustacea; Hexapoda; Insecta; Dicondylia; Pterygota; Neoptera; Endopterygota; Diptera; Brachycera; Muscomorpha; Eremoneura; Cyclorrhapha; Schizophora; Acalyptratae; Ephydroidea; Drosophilidae; Drosophilinae; Drosophilini; Drosophila; Sophophora; melanogaster group; eugracilis subgroup; Drosophila eugracilis |
| *Drosophila melanogaster* | GCF_000001215.4 | NCBI | 99.9 | 98.8 | 1.1 | 0.0 | 0.1 | 1367 | Arthropoda; Mandibulata; Pancrustacea; Hexapoda; Insecta; Dicondylia; Pterygota; Neoptera; Endopterygota; Diptera; Brachycera; Muscomorpha; Eremoneura; Cyclorrhapha; Schizophora; Acalyptratae; Ephydroidea; Drosophilidae; Drosophilinae; Drosophilini; Drosophila; Sophophora; melanogaster group; melanogaster subgroup; Drosophila melanogaster |
| *Drosophila erecta* | GCF_003286155.1 | NCBI | 99.9 | 98.8 | 1.1 | 0.1 | 0.0 | 1367 | Arthropoda; Mandibulata; Pancrustacea; Hexapoda; Insecta; Dicondylia; Pterygota; Neoptera; Endopterygota; Diptera; Brachycera; Muscomorpha; Eremoneura; Cyclorrhapha; Schizophora; Acalyptratae; Ephydroidea; Drosophilidae; Drosophilinae; Drosophilini; Drosophila; Sophophora; melanogaster group; melanogaster subgroup; Drosophila erecta |
| *Drosophila teissieri* | GCF_016746235.2 | NCBI | 99.8 | 98.0 | 1.8 | 0.0 | 0.2 | 1367 | Arthropoda; Mandibulata; Pancrustacea; Hexapoda; Insecta; Dicondylia; Pterygota; Neoptera; Endopterygota; Diptera; Brachycera; Muscomorpha; Eremoneura; Cyclorrhapha; Schizophora; Acalyptratae; Ephydroidea; Drosophilidae; Drosophilinae; Drosophilini; Drosophila; Sophophora; melanogaster group; melanogaster subgroup; Drosophila teissieri |
| *Drosophila yakuba* | GCF_016746365.2 | NCBI | 99.9 | 99.2 | 0.7 | 0.0 | 0.1 | 1367 | Arthropoda; Mandibulata; Pancrustacea; Hexapoda; Insecta; Dicondylia; Pterygota; Neoptera; Endopterygota; Diptera; Brachycera; Muscomorpha; Eremoneura; Cyclorrhapha; Schizophora; Acalyptratae; Ephydroidea; Drosophilidae; Drosophilinae; Drosophilini; Drosophila; Sophophora; melanogaster group; melanogaster subgroup; Drosophila yakuba |
| *Drosophila sechellia* | GCF_004382195.2 | NCBI | 100.0 | 98.8 | 1.2 | 0.0 | 0.0 | 1367 | Arthropoda; Mandibulata; Pancrustacea; Hexapoda; Insecta; Dicondylia; Pterygota; Neoptera; Endopterygota; Diptera; Brachycera; Muscomorpha; Eremoneura; Cyclorrhapha; Schizophora; Acalyptratae; Ephydroidea; Drosophilidae; Drosophilinae; Drosophilini; Drosophila; Sophophora; melanogaster group; melanogaster subgroup; Drosophila sechellia |
| *Drosophila mauritiana* | GCF_004382145.1 | NCBI | 99.8 | 98.8 | 1.0 | 0.0 | 0.2 | 1367 | Arthropoda; Mandibulata; Pancrustacea; Hexapoda; Insecta; Dicondylia; Pterygota; Neoptera; Endopterygota; Diptera; Brachycera; Muscomorpha; Eremoneura; Cyclorrhapha; Schizophora; Acalyptratae; Ephydroidea; Drosophilidae; Drosophilinae; Drosophilini; Drosophila |
| *Drosophila simulans* | GCF_016746395.2 | NCBI | 100.0 | 98.8 | 1.2 | 0.0 | 0.0 | 1367 | Arthropoda; Mandibulata; Pancrustacea; Hexapoda; Insecta; Dicondylia; Pterygota; Neoptera; Endopterygota; Diptera; Brachycera; Muscomorpha; Eremoneura; Cyclorrhapha; Schizophora; Acalyptratae; Ephydroidea; Drosophilidae; Drosophilinae; Drosophilini; Drosophila; Sophophora; melanogaster group; melanogaster subgroup; Drosophila simulans |

**Table S17** - Uniprot accession numbers for sequences used to filter and classify the dataset utilized

| Uniprot Accession ID | Species | Phylum | Lysozyme type |
| --- | --- | --- | --- |
| O76357 | *Caenorhabditis elegans* | Nematoda | I |
| Q1XG90 | *Crassostrea virginica* | Mollusca | I |
| Q8IU26 | *Ruditapes philippinarum* | Mollusca | I |
| P83972 | *Drosophila melanogaster* | Arthropoda | I |
| C0HLB7 | *Lucilia sericata* | Arthropoda | I |
| O02119 | *Caenorhabditis elegans* | Nematoda | I |
| Q7YT16 | *Musca domestica* | Arthropoda | I |
| B3A003 | *Crassostrea virginica* | Mollusca | C |
| O75951 | *Homo sapiens* | Chordata | C |
| P00697 | *Rattus norvegicus* | Chordata | C |
| P00698 | *Gallus gallus* | Chordata | C |
| P00701 | *Coturnix japonica* | Chordata | C |
| P00702 | *Phasianus colchicus colchicus* | Chordata | C |
| P00703 | *Meleagris gallopavo* | Chordata | C |
| P04421 | *Bos taurus* | Chordata | C |
| P08905 | *Mus musculus* | Chordata | C |
| P11941 | *Oncorhynchus mykiss* | Chordata | C |
| P17607 | *Ovis aries* | Chordata | C |
| P17897 | *Mus musculus* | Chordata | C |
| P61626 | *Homo sapiens* | Chordata | C |
| P83673 | *Crassostrea virginica* | Mollusca | C |
| Q9DA11 | *Mus musculus* | Chordata | C |
| C1IIX1 | *Penaeus merguiensis* | Arthropoda | C |
| G3XDT7 | *Dromaius novaehollandiae* | Chordata | C |
| P00704 | *Numida meleagris* | Chordata | C |
| P00705 | *Anas platyrhynchos* | Chordata | C |
| P05105 | *Hyalophora cecropia* | Arthropoda | C |
| P12067 | *Sus scrofa* | Chordata | C |
| P12068 | *Sus scrofa* | Chordata | C |
| P12069 | *Sus scrofa* | Chordata | C |
| P29615 | *Drosophila melanogaster* | Arthropoda | C |
| P30201 | *Macaca mulatta* | Chordata | C |
| P37159 | *Drosophila melanogaster* | Arthropoda | C |
| P37161 | *Drosophila melanogaster* | Arthropoda | C |
| P51782 | *Trichosurus vulpecula* | Chordata | C |
| P61627 | *Pan paniscus* | Chordata | C |
| P61628 | *Pan troglodytes* | Chordata | C |
| P61629 | *Papio anubis* | Chordata | C |
| P61630 | *Cercocebus atys* | Chordata | C |
| P61631 | *Colobus angolensis* | Chordata | C |
| P61632 | *Colobus guereza* | Chordata | C |
| P61634 | *Erythrocebus patas* | Chordata | C |
| P67977 | *Semnopithecus entellus* | Chordata | C |
| P67978 | *Trachypithecus vetulus* | Chordata | C |
| P67979 | *Trachypithecus obscurus* | Chordata | C |
| P67980 | *Trachypithecus francoisi* | Chordata | C |
| P79158 | *Callithrix jacchus* | Chordata | C |
| P79179 | *Gorilla gorilla gorilla* | Chordata | C |
| P79180 | *Hylobates lar* | Chordata | C |
| P79239 | *Pongo pygmaeus* | Chordata | C |
| P79268 | *Saguinus oedipus* | Chordata | C |
| P79294 | *Saimiri sciureus* | Chordata | C |
| P79687 | *Allenopithecus nigroviridis* | Chordata | C |
| P79806 | *Miopithecus talapoin* | Chordata | C |
| P79811 | *Nasalis larvatus* | Chordata | C |
| P79847 | *Pygathrix nemaeus* | Chordata | C |
| P85045 | *Bufo gargarizans andrewsi* | Chordata | C |
| Q05820 | *Rattus norvegicus* | Chordata | C |
| Q06283 | *Bos taurus* | Chordata | C |
| Q06284 | *Bos taurus* | Chordata | C |
| Q06285 | *Bos taurus* | Chordata | C |
| Q27996 | *Bos taurus* | Chordata | C |
| Q29RT1 | *Bos taurus* | Chordata | C |
| Q91159 | *Opisthocomus hoazin* | Chordata | C |
| Q9TUN1 | *Ovis aries* | Chordata | C |
| A0JNM6 | *Bos taurus* | Chordata | C |
| D9J143 | *Lucilia sericata* | Arthropoda | C |
| P37160 | *Drosophila melanogaster* | Arthropoda | C |
| P48816 | *Bombyx mori* | Arthropoda | C |
| P50717 | *Hyphantria cunea* | Arthropoda | C |
| P50718 | *Trichoplusia ni* | Arthropoda | C |
| P61633 | *Chlorocebus aethiops* | Chordata | C |
| P61944 | *Takifugu rubripes* | Chordata | C |
| P80189 | *Bos taurus* | Chordata | C |
| Q08694 | *Drosophila melanogaster* | Arthropoda | C |
| Q17005 | *Anopheles gambiae* | Arthropoda | C |
| Q659U0 | *Leptonychotes weddellii* | Chordata | C |
| Q659U1 | *Phoca vitulina* | Chordata | C |
| Q659U5 | *Halichoerus grypus* | Chordata | C |
| Q6B410 | *Bos taurus* | Chordata | C |
| Q6B411 | *Bos taurus* | Chordata | C |
| Q6L6Q5 | *Ostrea edulis* | Mollusca | C |
| Q6L6Q6 | *Crassostrea gigas* | Mollusca | C |
| Q6UWQ5 | *Homo sapiens* | Chordata | C |
| Q7Z4W2 | *Homo sapiens* | Chordata | C |
| Q9DD65 | *Paralichthys olivaceus* | Chordata | C |
| Q9PU28 | *Scophthalmus maximus* | Chordata | C |

**Table S18 -** Scientific articles used to guide the construction of the constraint tree and to assess the topological accuracy of the phylogenetic tree inferred in the present study

| Order | Author | Publication year | Title | DOI/Pages |
| --- | --- | --- | --- | --- |
| Decapoda | Misof et al. | 2014 | Phylogenomics resolves the timing and pattern of insect evolution | https://doi.org/10.1126/science.1257570 |
| Entomobryomorpha | Misof et al. | 2014 | Phylogenomics resolves the timing and pattern of insect evolution | https://doi.org/10.1126/science.1257570 |
| Odonata | Misof et al. | 2014 | Phylogenomics resolves the timing and pattern of insect evolution | https://doi.org/10.1126/science.1257570 |
| Ephemeroptera | Misof et al. | 2014 | Phylogenomics resolves the timing and pattern of insect evolution | https://doi.org/10.1126/science.1257570 |
| Phasmatodea | Misof et al. | 2014 | Phylogenomics resolves the timing and pattern of insect evolution | https://doi.org/10.1126/science.1257570 |
| Blattodea | Evangelista D.A et al. | 2019 | An integrative phylogenomic approach illuminates the evolutionary history of cockroaches and termites (Blattodea) | https://doi.org/10.1098/rspb.2018.2076 |
| Blattodea | Kambhampati and Eggleton | 2000 | Taxonomy and Phylogeny of Termites | Termites: Evolution, Sociality, Symbioses, Ecology, 1-23. |
| Blattodea | Cameron et al. | 2012 | A mitochondrial genome phylogeny of termites (Blattodea: Termitoidae): Robust support for interfamilial relationships and molecular synapomorphies define major clades | 10.1016/j.ympev.2012.05.034 |
| Blattodea | Wang et al. | 2017 | Reconstructing the phylogeny of Blattodea: Robust support for interfamilial relationships and major clades | 10.1038/s41598-017-04243-1 |
| Hemiptera | Cryan and Urban | 2011 | Higher-level phylogeny of the insect order Hemiptera: is Auchenorrhyncha really paraphyletic? | https://doi.org/10.1111/j.1365-3113.2011.00611.x |
| Hemiptera | Schuh, Weirauch and Wheeler | 2009 | Phylogenetic relationships within the Cimicomorpha (Hemiptera: Heteroptera): a total-evidence analysis | https://doi.org/10.1111/j.1365-3113.2008.00436.x |
| Hemiptera | Drohojowska, J. et al. | 2020 | Fossils reshape the Sternorrhyncha evolutionary tree (Insecta, Hemiptera) | https://doi.org/10.1038/s41598-020-68220-x |
| Hemiptera | Rivas and Torres | 2010 | Combination of molecular data support the existence of three main lineages in the phylogeny of aphids (Hemiptera: Aphididae) and the basal position of the subfamily Lachninae | https://doi.org/10.1016/j.ympev.2009.12.005 |
| Hemiptera | Rebijith et al | 2017 | Reconstructing the macroevolutionary patterns of aphids (Hemiptera: Aphididae) using nuclear and mitochondrial DNA sequences | https://doi.org/10.1093/biolinnean/blx020 |
| Hemiptera | Song and Zhang | 2022 | A comprehensive analysis of higher-level phylogenetic relationships of Hemiptera based on transcriptome data | https://doi.org/10.1111/jse.12855 |
| Hemiptera | Song et al | 2024 | Phylogeny and evolution of hemipteran insects based on expanded genomic and transcriptomic data | https://doi.org/10.1186/s12915-024-01991-1 |
| Hemiptera | Johnson et al | 2018 | Phylogenomics and the evolution of hemipteroid insects | https://doi.org/10.1073/pnas.1815820115 |
| Phthiraptera | Misof et al. | 2014 | Phylogenomics resolves the timing and pattern of insect evolution | https://doi.org/10.1126/science.1257570 |
| Hymenoptera | Niu et al. | 2022 | Phylogenomic Analyses of the Tenthredinoidea Support the Familial Rank of Athaliidae (Insecta, Tenthredinoidea) | https://doi.org/10.3390/insects13100858 |
| Hymenoptera | Linnen and Farrell | 2008 | Comparison of Methods for Species-Tree Inference in the Sawfly Genus Neodiprion (Hymenoptera: Diprionidae) | https://doi.org/10.1080/10635150802580949 |
| Hymenoptera | Zhang, Bossert and Spasovejic | 2024 | Evolving perspectives in Hymenoptera systematics: Bridging fossils and genomes across time | https://doi.org/10.1111/syen.12645 |
| Hymenoptera | Munro et al. | 2011 | A Molecular Phylogeny of the Chalcidoidea (Hymenoptera) | https://doi.org/10.1371/journal.pone.0027023 |
| Hymenoptera | Wharton et al. | 1992 | Phylogeny of the Subfamiles of the Family Braconidae (Hymenoptera: Icheneumonoidea): A Reassessment | https://doi.org/10.1111/j.1096-0031.1992.tb00068.x |
| Hymenoptera | Arévalo et al. | 2004 | The phylogeny of the social wasp subfamily Polistinae: evidence from microsatellite flanking sequences, mitochondrial COI sequence, and morphological characters | https://doi.org/10.1186/1471-2148-4-8 |
| Hymenoptera | Schmitz and Moritz | 1998 | Molecular Phylogeny of Vespidae (Hymenoptera) and the Evolution of Sociality in Wasps | https://doi.org/10.1006/mpev.1997.0460 |
| Hymenoptera | Perrard et al | 2013 | Phylogeny of hornets: a total evidence approach (Hymenoptera, Vespidae, Vespinae, Vespa ) | https://doi.org/10.3897/jhr.32.4685 |
| Hymenoptera | Schimidt | 2013 | Molecular phylogenetics of ponerine ants (Hymenoptera: Formicidae: Ponerinae) | https://doi.org/10.11646/zootaxa.3647.2.1 |
| Hymenoptera | Ward, Blaimer and Fisher | 2016 | A revised phylogenetic classification of the ant subfamily Formicinae (Hymenoptera: Formicidae), with resurrection of the genera Colobopsis and Dinomyrmex | https://doi.org/10.11646/zootaxa.4072.3.4 |
| Hymenoptera | Ward et al. | 2014 | The evolution of myrmicine ants: phylogeny and biogeography of a hyperdiverse ant clade (Hymenoptera: Formicidae) | https://doi.org/10.1111/syen.12090 |
| Hymenoptera | Debevec, Cardinal and Danforth | 2012 | Identifying the sister group to the bees: a molecular phylogeny of Aculeata with an emphasis on the superfamily Apoidea | https://doi.org/10.1111/j.1463-6409.2012.00549.x |
| Hymenoptera | Bossert et al. | 2019 | Combining transcriptomes and ultraconserved elements to illuminate the phylogeny of Apidae | https://doi.org/10.1016/j.ympev.2018.10.012 |
| Hymenoptera | Jasso-Martínez et al | 2022 | Phylogenomics of braconid wasps (Hymenoptera, Braconidae) sheds light on classification and the evolution of parasitoid life history traits | doi:10.1016/j.ympev.2022.107452 |
| Hymenoptera | Cameron, Hines and Williams | 2007 | A comprehensive phylogeny of the bumble bees (Bombus) | https://doi.org/10.1111/j.1095-8312.2007.00784.x |
| Neuroptera | Misof et al. | 2014 | Phylogenomics resolves the timing and pattern of insect evolution | https://doi.org/10.1126/science.1257570 |
| Coleoptera | Kundrata, Bocakova and Bocak | 2014 | The comprehensive phylogeny of the superfamily Elateroidea (Coleoptera: Elateriformia) | https://doi.org/10.1016/j.ympev.2014.03.012 |
| Coleoptera | Kergoat et al. | 2014 | Higher level molecular phylogeny of darkling beetles (Coleoptera: Tenebrionidae) | https://doi.org/10.1111/syen.12065 |
| Coleoptera | Cai et al. | 2022 | Integrated phylogenomics and fossil data illuminate the evolution of beetles | https://doi.org/10.1098/rsos.211771 |
| Lepidoptera | Kawahara et al. | 2019 | Phylogenomics reveals the evolutionary timing and pattern of butterflies and moths | https://doi.org/10.1073/pnas.1907847116 |
| Lepidoptera | Pashley | 1983 | Biosystematic Study in Tortricidae (Lepidoptera), with a Note on Evolutionary Rates of Allozymes | https://doi.org/10.1093/aesa/76.2.139 |
| Lepidoptera | Riyaz et al. | 2021 | Comparative mitochondrial genome analysis of Eudocima salaminia (Cramer, 1777) (Lepidoptera: Noctuoidea), novel gene rearrangement and phylogenetic relationship within the superfamily Noctuoidea | https://doi.org/10.1007/s11033-021-06465-z |
| Lepidoptera | Condamine et al. | 2023 | A comprehensive phylogeny and revised taxonomy illuminate the origin and diversification of the global radiation of Papilio (Lepidoptera: Papilionidae) | https://doi.org/10.1016/j.ympev.2023.107758 |
| Lepidoptera | Wahlberg et al. | 2014 | Revised systematics and higher classification of pierid butterflies (Lepidoptera: Pieridae) based on molecular data | https://doi.org/10.1111/zsc.12075 |
| Lepidoptera | Okamura et al. | 2019 | Molecular signatures of selection associated with host plant differences in Pieris butterflies | https://doi.org/10.1111/mec.15268 |
| Lepidoptera | Espeland et al. | 2018 | A Comprehensive and Dated Phylogenomic Analysis of Butterflies | https://doi.org/10.1016/j.cub.2018.01.061 |
| Lepidoptera | Shi QH et al. | 2015 | Morphological Characters Are Compatible with Mitogenomic Data in Resolving the Phylogeny of Nymphalid Butterflies (Lepidoptera: Papilionoidea: Nymphalidae) | https://doi.org/10.1371/journal.pone.0124349 |
| Diptera | Suvorov et al. | 2022 | Widespread introgression across a phylogeny of 155 Drosophila genomes | https://doi.org/10.1016/j.cub.2021.10.052 |
| Diptera | Lambkin et al. | 2012 | The phylogenetic relationships among infraorders and superfamilies of Diptera based on morphological evidence | https://doi.org/10.1111/j.1365-3113.2012.00652.x |
| Diptera | Judd D. | 1996 | Review of the systematics and phylogenetic relationships of the Sabethini (Diptera: Culicidae) | https://doi.org/10.1046/j.1365-3113.1996.d01-1.x |
| Diptera | Harbach R.E | 2007 | The Culicidae (Diptera): a review of taxonomy, classification and phylogeny | https://doi.org/10.11646/zootaxa.1668.1.288 |
| Diptera | Zadra, Rizzoli and Rota-Stabelli | 2021 | Chronological Incongruences between Mitochondrial and Nuclear Phylogenies of Aedes Mosquitoes | https://doi.org/10.3390/life11030181 |
| Diptera | Freitas et al. | 2015 | Diversification of the Genus Anopheles and a Neotropical Clade from the Late Cretaceous | https://doi.org/10.1371/journal.pone.0134462 |
| Diptera | Wiegmann et al | 2011 | Episodic radiations in the fly tree of life | https://doi.org/10.1073/pnas.1012675108 |
| Diptera | Yuan et al | 2025 | Study of Mitogenomes Provides Implications for the Phylogenetics and Evolution of the Infraorder Muscomorpha in Diptera | https://doi.org/10.1002/ece3.70832 |
| Diptera | Bayless et al | 2021 | Beyond Drosophila: resolving the rapid radiation of schizophoran flies with phylotranscriptomics | https://doi.org/10.1186/s12915-020-00944-8 |
| Diptera | Silva et al | 2020 | Culicidae evolutionary history focusing on the Culicinae subfamily based on mitochondrial phylogenomics | https://doi.org/10.1038/s41598-020-74883-3 |

**Table S19 -** Dietary habits of the species used in the present study

| Species | Diet - Adult | Diet - Larvae | Order |
| --- | --- | --- | --- |
| *Eriocheir sinensis* | Omnivore | Omnivore | Decapoda |
| *Procambarus clarkii* | Omnivore | Omnivore | Decapoda |
| *Scylla paramamosain* | Detritivore | Detritivore | Decapoda |
| *Folsomia candida* | Detritivore | Detritivore | Entomobryomorpha |
| *Ischnura elegans* | Invertebrates | Invertebrates | Odonata |
| *Hetaerina americana* | Invertebrates | Invertebrates | Odonata |
| *Cloeon dipterum* | Plants | Plants | Ephemeroptera |
| *Neocloeon triangulifer* | Plants | Plants | Ephemeroptera |
| *Anabrus simplex* | Plants | Plants | Orthoptera |
| *Gryllus longicercus* | Plants | Plants | Orthoptera |
| *Bacillus rossius redtenbacheri* | Plants | Plants | Phasmatodea |
| *Blattella germanica* | Detritivore | Detritivore | Blattodea |
| *Periplaneta americana* | Detritivore | Detritivore | Blattodea |
| *Zootermopsis nevadensis* | Plants | Plants | Blattodea |
| *Coptotermes formosanus* | Plants | Plants | Blattodea |
| *Cryptotermes secundus* | Plants | Plants | Blattodea |
| *Frankliniella occidentalis* | Plants | Plants | Thysanoptera |
| *Thrips palmi* | Plants | Plants | Thysanoptera |
| *Homalodisca vitripennis* | Plants | Plants | Hemiptera |
| *Laodelphax striatellus* | Plants | Plants | Hemiptera |
| *Nilaparvata lugens* | Plants | Plants | Hemiptera |
| *Halyomorpha halys* | Plants | Plants | Hemiptera |
| *Rhodnius prolixus* | Blood | Blood | Hemiptera |
| *Apolygus lucorum* | Plants | Plants | Hemiptera |
| *Cimex lectularius* | Blood | Blood | Hemiptera |
| *Bemisia tabaci* | Plants | Plants | Hemiptera |
| *Planococcus citri* | Plants | Plants | Hemiptera |
| *Adelges cooleyi* | Plants | Plants | Hemiptera |
| *Daktulosphaira vitifoliae* | Plants | Plants | Hemiptera |
| *Sipha flava* | Plants | Plants | Hemiptera |
| *Acyrthosiphon pisum* | Plants | Plants | Hemiptera |
| *Myzus persicae* | Plants | Plants | Hemiptera |
| *Melanaphis sacchari* | Plants | Plants | Hemiptera |
| *Aphis craccivora* | Plants | Plants | Hemiptera |
| *Aphis gossypii* | Plants | Plants | Hemiptera |
| *Rhopalosiphum maidis* | Plants | Plants | Hemiptera |
| *Rhopalosiphum padi* | Plants | Plants | Hemiptera |
| *Pediculus humanus corporis* | Blood | Blood | Phthiraptera |
| *Athalia rosae* | Plants | Plants | Hymenoptera |
| *Diprion similis* | Plants | Plants | Hymenoptera |
| *Neodiprion fabricii* | Plants | Plants | Hymenoptera |
| *Neodiprion virginianus* | Plants | Plants | Hymenoptera |
| *Neodiprion lecontei* | Plants | Plants | Hymenoptera |
| *Neodiprion pinetum* | Plants | Plants | Hymenoptera |
| *Cephus cinctus* | Plants | Plants | Hymenoptera |
| *Orussus abietinus* | Parasitoid | Parasitoid | Hymenoptera |
| *Belonocnema kinseyi* | Plants | Plants | Hymenoptera |
| *Leptopilina boulardi* | Parasitoid | Parasitoid | Hymenoptera |
| *Leptopilina heterotoma* | Parasitoid | Parasitoid | Hymenoptera |
| *Trichogramma pretiosum* | Parasitoid | Parasitoid | Hymenoptera |
| *Copidosoma floridanum* | Parasitoid | Parasitoid | Hymenoptera |
| *Phymastichus coffea* | Parasitoid | Parasitoid | Hymenoptera |
| *Ceratosolen solmsi marchali* | Plants | Plants | Hymenoptera |
| *Nasonia vitripennis* | Parasitoid | Parasitoid | Hymenoptera |
| *Venturia canescens* | Parasitoid | Parasitoid | Hymenoptera |
| *Aphidius gifuensis* | Parasitoid | Parasitoid | Hymenoptera |
| *Fopius arisanus* | Parasitoid | Parasitoid | Hymenoptera |
| *Diachasma alloeum* | Parasitoid | Parasitoid | Hymenoptera |
| *Diachasmimorpha longicaudata* | Parasitoid | Parasitoid | Hymenoptera |
| *Chelonus insularis* | Parasitoid | Parasitoid | Hymenoptera |
| *Cotesia glomerata* | Parasitoid | Parasitoid | Hymenoptera |
| *Microplitis demolitor* | Parasitoid | Parasitoid | Hymenoptera |
| *Microplitis mediator* | Parasitoid | Parasitoid | Hymenoptera |
| *Polistes dominula* | Omnivore | Omnivore | Hymenoptera |
| *Polistes canadensis* | Omnivore | Omnivore | Hymenoptera |
| *Polistes fuscatus* | Omnivore | Omnivore | Hymenoptera |
| *Vespula pensylvanica* | Omnivore | Omnivore | Hymenoptera |
| *Vespula vulgaris* | Omnivore | Omnivore | Hymenoptera |
| *Vespa velutina* | Omnivore | Omnivore | Hymenoptera |
| *Vespa crabro* | Omnivore | Omnivore | Hymenoptera |
| *Vespa mandarinia* | Omnivore | Omnivore | Hymenoptera |
| *Ampulex compressa* | Omnivore | Omnivore | Hymenoptera |
| *Colletes gigas* | Plants | Plants | Hymenoptera |
| *Hylaeus anthracinus* | Plants | Plants | Hymenoptera |
| *Hylaeus volcanicus* | Plants | Plants | Hymenoptera |
| *Dufourea novaeangliae* | Plants | Plants | Hymenoptera |
| *Megalopta genalis* | Plants | Plants | Hymenoptera |
| *Nomia melanderi* | Plants | Plants | Hymenoptera |
| *Megachile rotundata* | Plants | Plants | Hymenoptera |
| *Osmia bicornis bicornis* | Plants | Plants | Hymenoptera |
| *Osmia lignaria* | Plants | Plants | Hymenoptera |
| *Ceratina calcarata* | Plants | Plants | Hymenoptera |
| *Eufriesea mexicana* | Plants | Plants | Hymenoptera |
| *Bombus affinis* | Plants | Plants | Hymenoptera |
| *Bombus terrestris* | Plants | Plants | Hymenoptera |
| *Frieseomelitta varia* | Plants | Plants | Hymenoptera |
| *Melipona quadrifasciata* | Plants | Plants | Hymenoptera |
| *Apis florea* | Plants | Plants | Hymenoptera |
| *Apis cerana* | Plants | Plants | Hymenoptera |
| *Apis mellifera* | Plants | Plants | Hymenoptera |
| *Apis dorsata* | Plants | Plants | Hymenoptera |
| *Apis laboriosa* | Plants | Plants | Hymenoptera |
| *Dinoponera quadriceps* | Invertebrates | Invertebrates | Hymenoptera |
| *Harpegnathos saltator* | Invertebrates | Invertebrates | Hymenoptera |
| *Odontomachus brunneus* | Invertebrates | Invertebrates | Hymenoptera |
| *Eciton burchellii* | Invertebrates | Invertebrates | Hymenoptera |
| *Ooceraea biroi* | Invertebrates | Invertebrates | Hymenoptera |
| *Linepithema humile* | Omnivore | Omnivore | Hymenoptera |
| *Pseudomyrmex gracilis* | Omnivore | Omnivore | Hymenoptera |
| *Nylanderia fulva* | Omnivore | Omnivore | Hymenoptera |
| *Camponotus floridanus* | Omnivore | Omnivore | Hymenoptera |
| *Cataglyphis hispanica* | Invertebrates | Invertebrates | Hymenoptera |
| *Formica exsecta* | Omnivore | Omnivore | Hymenoptera |
| *Pogonomyrmex barbatus* | Plants | Plants | Hymenoptera |
| *Vollenhovia emeryi* | Omnivore | Omnivore | Hymenoptera |
| *Temnothorax curvispinosus* | Omnivore | Omnivore | Hymenoptera |
| *Temnothorax longispinosus* | Omnivore | Omnivore | Hymenoptera |
| *Monomorium pharaonis* | Omnivore | Omnivore | Hymenoptera |
| *Solenopsis invicta* | Omnivore | Omnivore | Hymenoptera |
| *Wasmannia auropunctata* | Omnivore | Omnivore | Hymenoptera |
| *Cyphomyrmex costatus* | Omnivore | Omnivore | Hymenoptera |
| *Trachymyrmex septentrionalis* | Omnivore | Omnivore | Hymenoptera |
| *Acromyrmex echinatior* | Omnivore | Omnivore | Hymenoptera |
| *Atta cephalotes* | Omnivore | Omnivore | Hymenoptera |
| *Atta colombica* | Omnivore | Omnivore | Hymenoptera |
| *Chrysoperla carnea* | Invertebrates | Invertebrates | Neuroptera |
| *Agrilus planipennis* | Plants | Plants | Coleoptera |
| *Ignelater luminosus* | Invertebrates | Invertebrates | Coleoptera |
| *Abscondita terminalis* | Invertebrates | Invertebrates | Coleoptera |
| *Lamprigera yunnana* | Invertebrates | Invertebrates | Coleoptera |
| *Photinus pyralis* | Invertebrates | Invertebrates | Coleoptera |
| *Onthophagus taurus* | Detritivore | Detritivore | Coleoptera |
| *Dalotia coriaria* | Invertebrates | Invertebrates | Coleoptera |
| *Nicrophorus vespilloides* | Detritivore | Detritivore | Coleoptera |
| *Tenebrio molitor* | Omnivore | Omnivore | Coleoptera |
| *Zophobas morio* | Omnivore | Omnivore | Coleoptera |
| *Tribolium castaneum* | Omnivore | Omnivore | Coleoptera |
| *Tribolium madens* | Omnivore | Omnivore | Coleoptera |
| *Coccinella septempunctata* | Invertebrates | Invertebrates | Coleoptera |
| *Harmonia axyridis* | Invertebrates | Invertebrates | Coleoptera |
| *Aethina tumida* | Invertebrates | Invertebrates | Coleoptera |
| *Brassicogethes aeneus* | Plants | Plants | Coleoptera |
| *Cylas formicarius* | Plants | Plants | Coleoptera |
| *Anthonomus grandis grandis* | Plants | Plants | Coleoptera |
| *Dendroctonus ponderosae* | Plants | Plants | Coleoptera |
| *Rhynchophorus ferrugineus* | Plants | Plants | Coleoptera |
| *Sitophilus oryzae* | Plants | Plants | Coleoptera |
| *Anoplophora glabripennis* | Plants | Plants | Coleoptera |
| *Gonioctena quinquepunctata* | Plants | Plants | Coleoptera |
| *Leptinotarsa decemlineata* | Plants | Plants | Coleoptera |
| *Diabrotica virgifera virgifera* | Plants | Plants | Coleoptera |
| *Diorhabda carinulata* | Plants | Plants | Coleoptera |
| *Diorhabda sublineata* | Plants | Plants | Coleoptera |
| *Plutella xylostella* | Plants | Plants | Lepidoptera |
| *Leguminivora glycinivorella* | Plants | Plants | Lepidoptera |
| *Cydia pomonella* | Plants | Plants | Lepidoptera |
| *Cydia splendana* | Plants | Plants | Lepidoptera |
| *Hyposmocoma kahamanoa* | Detritivore | Detritivore | Lepidoptera |
| *Pectinophora gossypiella* | Plants | Plants | Lepidoptera |
| *Chilo suppressalis* | Plants | Plants | Lepidoptera |
| *Ostrinia furnacalis* | Plants | Plants | Lepidoptera |
| *Ostrinia nubilalis* | Plants | Plants | Lepidoptera |
| *Achroia grisella* | Plants | Plants | Lepidoptera |
| *Galleria mellonella* | Plants | Plants | Lepidoptera |
| *Amyelois transitella* | Plants | Plants | Lepidoptera |
| *Plodia interpunctella* | Plants | Plants | Lepidoptera |
| *Manduca sexta* | Plants | Plants | Lepidoptera |
| *Bombyx mandarina* | Plants | Plants | Lepidoptera |
| *Bombyx mori* | Plants | Plants | Lepidoptera |
| *Arctia plantaginis* | Plants | Plants | Lepidoptera |
| *Trichoplusia ni* | Plants | Plants | Lepidoptera |
| *Helicoverpa armigera* | Plants | Plants | Lepidoptera |
| *Helicoverpa zea* | Plants | Plants | Lepidoptera |
| *Spodoptera frugiperda* | Plants | Plants | Lepidoptera |
| *Spodoptera litura* | Plants | Plants | Lepidoptera |
| *Parnassius apollo* | Plants | Plants | Lepidoptera |
| *Papilio polytes* | Plants | Plants | Lepidoptera |
| *Papilio machaon* | Plants | Plants | Lepidoptera |
| *Papilio xuthus* | Plants | Plants | Lepidoptera |
| *Leptidea sinapis* | Plants | Plants | Lepidoptera |
| *Colias croceus* | Plants | Plants | Lepidoptera |
| *Zerene cesonia* | Plants | Plants | Lepidoptera |
| *Pieris rapae* | Plants | Plants | Lepidoptera |
| *Pieris brassicae* | Plants | Plants | Lepidoptera |
| *Pieris napi* | Plants | Plants | Lepidoptera |
| *Aricia agestis* | Plants | Plants | Lepidoptera |
| *Danaus plexippus* | Plants | Plants | Lepidoptera |
| *Bicyclus anynana* | Plants | Plants | Lepidoptera |
| *Pararge aegeria* | Plants | Plants | Lepidoptera |
| *Maniola hyperantus* | Plants | Plants | Lepidoptera |
| *Maniola jurtina* | Plants | Plants | Lepidoptera |
| *Brenthis ino* | Plants | Plants | Lepidoptera |
| *Melitaea cinxia* | Plants | Plants | Lepidoptera |
| *Nymphalis io* | Plants | Plants | Lepidoptera |
| *Vanessa cardui* | Plants | Plants | Lepidoptera |
| *Vanessa atalanta* | Plants | Plants | Lepidoptera |
| *Vanessa tameamea* | Plants | Plants | Lepidoptera |
| *Culicoides brevitarsis* | Blood | Blood | Diptera |
| *Clunio marinus* | Omnivore | Omnivore | Diptera |
| *Polypedilum vanderplanki* | Omnivore | Omnivore | Diptera |
| *Toxorhynchites rutilus septentrionalis* | Invertebrates | Invertebrates | Diptera |
| *Uranotaenia lowii* | Blood | Blood | Diptera |
| *Malaya genurostris* | Blood | Blood | Diptera |
| *Topomyia yanbarensis* | Blood | Blood | Diptera |
| *Sabethes cyaneus* | Blood | Blood | Diptera |
| *Wyeomyia smithii* | Blood | Blood | Diptera |
| *Culex pipiens pallens* | Blood | Blood | Diptera |
| *Culex quinquefasciatus* | Blood | Blood | Diptera |
| *Ochlerotatus camptorhynchus* | Blood | Blood | Diptera |
| *Armigeres subalbatus* | Blood | Blood | Diptera |
| *Aedes aegypti* | Blood | Blood | Diptera |
| *Aedes albopictus* | Blood | Blood | Diptera |
| *Anopheles coustani* | Blood | Blood | Diptera |
| *Anopheles ziemanni* | Blood | Blood | Diptera |
| *Anopheles bellator* | Blood | Blood | Diptera |
| *Anopheles cruzii* | Blood | Blood | Diptera |
| *Anopheles darlingi* | Blood | Blood | Diptera |
| *Anopheles albimanus* | Blood | Blood | Diptera |
| *Anopheles aquasalis* | Blood | Blood | Diptera |
| *Anopheles maculipalpis* | Blood | Blood | Diptera |
| *Anopheles stephensi* | Blood | Blood | Diptera |
| *Anopheles nili* | Blood | Blood | Diptera |
| *Anopheles funestus* | Blood | Blood | Diptera |
| *Anopheles moucheti* | Blood | Blood | Diptera |
| *Anopheles merus* | Blood | Blood | Diptera |
| *Anopheles arabiensis* | Blood | Blood | Diptera |
| *Anopheles coluzzii* | Blood | Blood | Diptera |
| *Anopheles gambiae* | Blood | Blood | Diptera |
| *Lutzomyia longipalpis* | Blood | Blood | Diptera |
| *Phlebotomus argentipes* | Blood | Blood | Diptera |
| *Phlebotomus papatasi* | Blood | Blood | Diptera |
| *Bradysia coprophila* | Detritivore | Detritivore | Diptera |
| *Contarinia nasturtii* | Plants | Plants | Diptera |
| *Sitodiplosis mosellana* | Plants | Plants | Diptera |
| *Hermetia illucens* | Detritivore | Detritivore | Diptera |
| *Condylostylus longicornis* | Invertebrates | Invertebrates | Diptera |
| *Episyrphus balteatus* | Invertebrates | Invertebrates | Diptera |
| *Eupeodes corollae* | Invertebrates | Invertebrates | Diptera |
| *Anastrepha ludens* | Plants | Plants | Diptera |
| *Anastrepha obliqua* | Plants | Plants | Diptera |
| *Ceratitis capitata* | Plants | Plants | Diptera |
| *Zeugodacus cucurbitae* | Plants | Plants | Diptera |
| *Bactrocera tryoni* | Plants | Plants | Diptera |
| *Bactrocera dorsalis* | Plants | Plants | Diptera |
| *Bactrocera latifrons* | Plants | Plants | Diptera |
| *Glossina fuscipes* | Blood | Blood | Diptera |
| *Calliphora vicina* | Detritivore | Detritivore | Diptera |
| *Lucilia cuprina* | Detritivore | Detritivore | Diptera |
| *Lucilia sericata* | Detritivore | Detritivore | Diptera |
| *Stomoxys calcitrans* | Blood | Detritivore | Diptera |
| *Musca domestica* | Detritivore | Detritivore | Diptera |
| *Musca vetustissima* | Detritivore | Detritivore | Diptera |
| *Scaptodrosophila lebanonensis* | Detritivore | Detritivore | Diptera |
| *Drosophila sulfurigaster albostrigata* | Detritivore | Detritivore | Diptera |
| *Drosophila albomicans* | Detritivore | Detritivore | Diptera |
| *Drosophila nasuta* | Detritivore | Detritivore | Diptera |
| *Drosophila grimshawi* | Detritivore | Detritivore | Diptera |
| *Drosophila montana* | Detritivore | Detritivore | Diptera |
| *Drosophila novamexicana* | Detritivore | Detritivore | Diptera |
| *Drosophila virilis* | Detritivore | Detritivore | Diptera |
| *Drosophila hydei* | Detritivore | Detritivore | Diptera |
| *Drosophila navojoa* | Detritivore | Detritivore | Diptera |
| *Drosophila arizonae* | Detritivore | Detritivore | Diptera |
| *Drosophila mojavensis* | Detritivore | Detritivore | Diptera |
| *Drosophila obscura* | Detritivore | Detritivore | Diptera |
| *Drosophila guanche* | Detritivore | Detritivore | Diptera |
| *Drosophila subobscura* | Detritivore | Detritivore | Diptera |
| *Drosophila miranda* | Detritivore | Detritivore | Diptera |
| *Drosophila persimilis* | Detritivore | Detritivore | Diptera |
| *Drosophila pseudoobscura* | Detritivore | Detritivore | Diptera |
| *Drosophila ananassae* | Detritivore | Detritivore | Diptera |
| *Drosophila bipectinata* | Detritivore | Detritivore | Diptera |
| *Drosophila kikkawai* | Detritivore | Detritivore | Diptera |
| *Drosophila serrata* | Detritivore | Detritivore | Diptera |
| *Drosophila ficusphila* | Plants | Detritivore | Diptera |
| *Drosophila elegans* | Plants | Detritivore | Diptera |
| *Drosophila rhopaloa* | Plants | Detritivore | Diptera |
| *Drosophila takahashii* | Plants | Detritivore | Diptera |
| *Drosophila biarmipes* | Plants | Detritivore | Diptera |
| *Drosophila subpulchrella* | Plants | Detritivore | Diptera |
| *Drosophila suzukii* | Plants | Detritivore | Diptera |
| *Drosophila eugracilis* | Plants | Detritivore | Diptera |
| *Drosophila melanogaster* | Detritivore | Detritivore | Diptera |
| *Drosophila erecta* | Plants | Detritivore | Diptera |
| *Drosophila teissieri* | Plants | Detritivore | Diptera |
| *Drosophila yakuba* | Plants | Detritivore | Diptera |
| *Drosophila sechellia* | Plants | Detritivore | Diptera |
| *Drosophila mauritiana* | Plants | Detritivore | Diptera |
| *Drosophila simulans* | Detritivore | Detritivore | Diptera |
